# Synthesis and Comparison of the Flame-Retardant Properties of Phosphorylated-Coumarins and Phosphorylated-Isophosphinolines

**DOI:** 10.3390/molecules30183717

**Published:** 2025-09-12

**Authors:** Issaka Ibrahim Abdou Rachid, Karen-Pacelye Mengue Me Ndong, Abdoul Razak Halidou Dougourikoye, Mina Hariri, Gabin Mwande-Maguene, Jacques Lebibi, Fatemeh Darvish, Ilagouma Amadou Tidjani, David Virieux, Jean-Luc Pirat, Tahar Ayad, Loic Dumazert, Arie van der Lee, Claire Negrell, Rodolphe Sonnier

**Affiliations:** 1 ICGM, Univ Montpellier, ENSCM, CNRS, 34090 Montpellier, France; abdrachid2i@gmail.com (I.I.A.R.); guemenkaren@gmail.com (K.-P.M.M.N.); pirat@enscm.fr (J.-L.P.);; 2 University Abdou Moumouni of Niamey, Niamey 10896, Niger; 3 Université des Sciences et Techniques de Masuku, Franceville 942, Gabon; gabin.maguene@gmail.com (G.M.-M.);; 4 Department of Chemistry, K. N. Toosi University of Technology, Tehran 19991-43344, Iran; 5 Polymers Composites and Hybrids (PCH), IMT Mines Ales, 30319 Ales, France; 6 IEM, University Montpellier, ENSCM, CNRS, 34095 Montpellier, France

**Keywords:** coumarin, isophosphinoline, phosphorus, pyrolysis–combustion flow calorimetry, flame retardancy, fire behavior

## Abstract

This study focuses on the synthesis, properties, and comparative analysis of new flame-retardant compounds: coumarins and isophosphinolines. These compounds feature a diarylphosphine oxide (DAPO) substituent at the β-position relative to both the carbonyl and the phosphoryl groups. Various derivatives with halogens, phosphorus, and/or aromatics substituents were synthetized and their thermal stability and flammability were evaluated at the microscale by thermogravimetric analysis (TGA) and pyrolysis–combustion flow calorimetry (PCFC) in order to identify the most promising molecules for use as flame-retardant (FR) additives or comonomers. FTIR-coupled PCFC analysis was also carried out to study the combustion profiles of the molecules. Beyond the confirmation of some expected trends, such as the char promotion of phosphorus and flame inhibition of halogens, the study revealed some unexpected findings that warrant further investigation. These include the prominent role of the chlorine substitution position on the aromatic ring, as well as significant differences in FR performance between diastereoisomers.

## 1. Introduction

Polymers are generally highly flammable and, in the event of a fire, they tend to release a large amount of heat. This promotes flame propagation and increases the hazard of a fire. To mitigate this risk, flame retardancy is typically achieved by incorporating FR additives or by integrating FR building blocks directly into the polymer backbone. While some elements, such as halogens and phosphorus, are well known for their FR properties, research on new FR polymeric structures or additives still remains a hot topic. However, this process often relies on an expensive and time-consuming trial-and-error approach, since the flame-retardant performance of a specific additive or structural unit cannot be accurately predicted prior to its incorporation into the host polymer. For example, the thermal stability of a small molecule is usually much lower than that of the polymer: for the former, evaporation can occur before pyrolysis. Similarly, the ability of a FR group to promote charring or to inhibit combustion needs to be assessed within the context of the whole macromolecular structure.

However, methods have been developed at the microscale to predict the flammability of polymers based on their chemical composition [1,2,3]. The modes-of-action of certain chemical groups can also be evaluated using specific procedures, which provide insight into their intrinsic FR efficiency [4]. It can be particularly valuable to characterize the intrinsic flammability of small FR molecules under well-controlled conditions, especially as part of a preliminary screening approach to select the most promising FR structures.

It is well recognized that microscale tests (i.e., thermogravimetric analysis and pyrolysis–combustion flow calorimetry) do not accurately correlate with performances at bench scale (i.e., cone calorimetry, limiting oxygen index, or UL94 tests, among others) [5]. The fire behavior of a material must be assessed according to a specific standard, depending on its application field, using a couple of different fire tests. Despite active research on this topic, no general correlation exists between these different tests. Nevertheless, determining flammability properties at the microscale is useful for better predicting fire behavior at the bench scale, and remains a promising approach. Some valuable examples can be found elsewhere [6].

**Figure 1:**
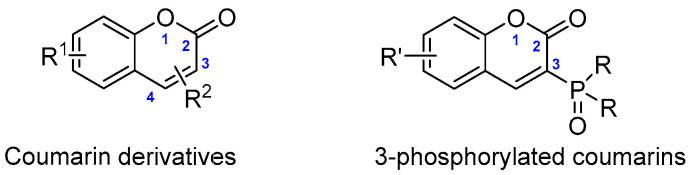
Coumarin derivatives and 3-phosphorylated coumarins.

Among the wide variety of molecular structures available for synthesizing new FR compounds, biobased building blocks are particularly attractive in light of growing environmental concerns. Coumarin, also known as 2H-1-benzopyran-2-one, is a naturally occurring biobased molecule found in many plants (Figure 1). Since its first synthesis by Perkin in 1868, coumarin and its derivatives have been widely used as fragrance ingredients in perfumes, soaps, and household products. In addition to these properties, coumarins also possess significant therapeutic potential, including anti-breast cancer, anti-HIV, anti-Alzheimer’s, vasorelaxant, and platelet anti-aggregatory effects [7,8]. Substituted coumarins, such as 3-phosphorylated coumarins, have shown notable cytotoxicity against leukemia cells and possess alkylating activity, further highlighting their versatility and potential for functional material design [9,10].

**Scheme 1:**
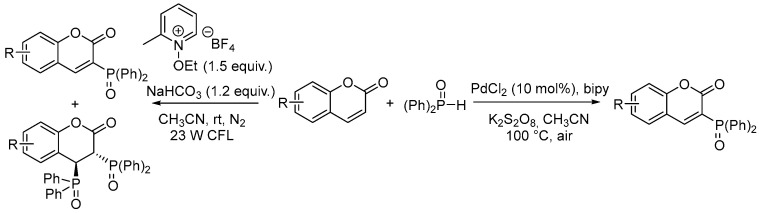
Direct 3-phosphonation of coumarins.

The phosphorylation of the coumarin ring at the C-3 position has been extensively studied. Notable examples of diethylcoumarin-3-phosphonate synthesis were reported by Singh et al. (1985) and Janecki et al. (2010) [11,12]. More recent advancements have introduced innovative approaches such as the photochemical phosphorylation of coumarins, developed by Mi et al. [13] and later by Xu in 2017 [14]. Additionally, C–H functionalization strategies have been explored, including a palladium-catalyzed phosphorylation method described by Wu et al. in 2013, as illustrated in Scheme 1 [15].

**Scheme 2:**
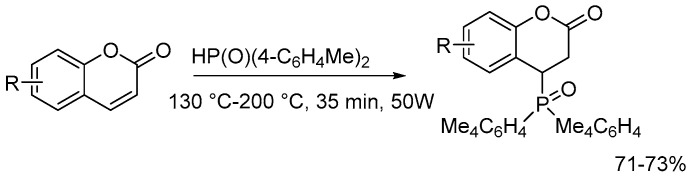
Direct 4-phosphonation of coumarins.

Fewer syntheses have been reported for 4-phosphonated or 4-phosphinylated coumarins. A notable example is the work by Lenker et al. (2011) [16], who successfully added di-tolylphosphine oxide to coumarins under microwave irradiation, without the need for solvents or catalysts (Scheme 2).

**Scheme 3:**
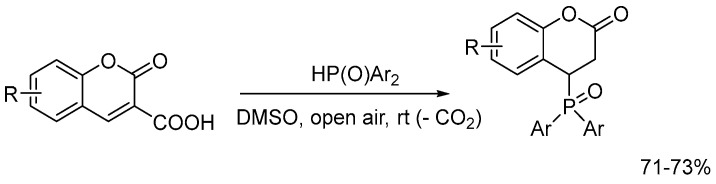
Access to a new series of 4-(diarylphosphoryl)chroman-2-ones via decarboxylative C-4 phosphorylation of coumarin-3-carboxylic acids.

Additionally, Brahmachari et al. (2020) [17] reported the green synthesis of 4-(diarylphosphoryl)chroman-2-ones under ambient conditions, further expanding the scope of environmentally friendly approaches to 4-phosphorylated coumarin derivatives (Scheme 3).

**Scheme 4:**
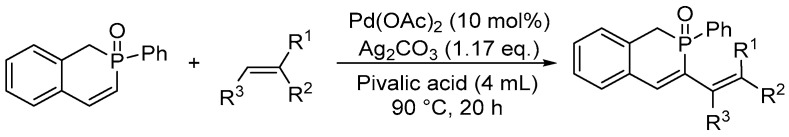
Regioselective C2 functionalization of 2-phenyl-1H-isophosphinoline 2-oxide.

Structures based on such aromatic moieties and heteroatoms have high flame-retardant potential [18]. We recently demonstrated that the association of a phosphine oxide (1H-isophosphinoline 2-oxide) with another heteroatom-carrying group has positive synergistic effects, enabling action in the condensed phase that is not seen in pure phosphine oxide FRs (Scheme 4) [19].

There have been few studies focusing on the flame-retardant properties of coumarin or isophosphinoline derivatives. In a recent paper, Roncucci et al. prepared a new bioFR combining coumarin and diphenylphosphine oxide (DPPO) [20]. When this was incorporated into polylactic acid (PLA) at 10 or 20 wt%, the authors measured the fire behavior of the composite. The Limiting Oxygen Index increased significantly (from 23.2 to 29.5 with 10 wt% of the new bioFR) and the UL94 test yielded a V0 rating. However, the improvement observed in the cone calorimeter test was considerably more modest, highlighting the limitations of the additive under more stringent fire scenarios.

Built on previous studies involving phosphoryl-containing natural products, and in continuation of our recent work devoted to the development of compounds bearing phosphoryl groups as substituents incorporated into natural products or isophosphinolines as substituents, this study focuses on the regioselective phosphorylation of coumarins at the C-3 position and of isophosphinoline at the β-position relative to the phosphoryl group. The flame-retardant potential of these new molecules was assessed by evaluating their thermal stability and heat release properties, using thermogravimetric analysis and pyrolysis–combustion flow calorimetry. The goal of this work is to examine the influence of different substituents, such as fluorine, chlorine, bromine, phosphorus, and aromatic moieties, when linked to these two building blocks (i.e., isophosphinoline and coumarin). By analyzing their thermal and flammability behavior, we aim to identify the most promising molecules for future application as efficient flame retardant additives or as key components in the design of innovative, intrinsically flame-retardant polymers.

## 2. Materials and Methods

### 2.1. Synthesis of Diarylphosphine Oxide

Various phosphine oxides were synthesized to demonstrate the Michael addition at either the C-3 position of the coumarin ring or at the β-position relative to the phosphoryl group. The crude products were isolated and compared with those described in the literature.

**Scheme 5:**
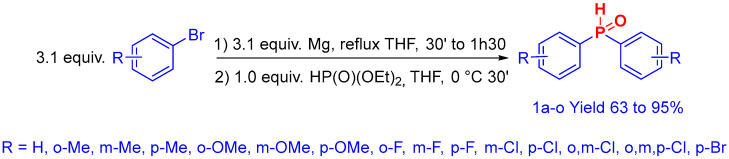
Synthesis of the diarylphosphine oxide.

Diarylphosphine oxides were obtained in good yields through a two-step reaction sequence (Scheme 5). In the first step, aryl halides were added dropwise to ground magnesium in anhydrous THF under reflux, within a nitrogen atmosphere. Upon completion of the reaction, the mixture was cooled to 0 °C, and diethyl phosphite was introduced slowly. The reaction was then quenched by hydrolysis with 1 M hydrochloric acid. The resulting mixture was filtered, and after extraction and separation via column chromatography, the diarylphosphines were isolated in yields ranging from 63% to 95% (Table 1).

Electron-withdrawing groups (EWGs) and electron-donating groups (EDGs) were introduced on the aryl halide precursors to assess the impact of these substituents on the flame-retardant properties of the resulting coumarin and isophosphinoline derivatives.

### 2.2. Synthesis of 4-Phosphorylated Coumarins COUM-R and 3-Phosphorylated Isophosphinolines ISOP-R

**Scheme 6:**
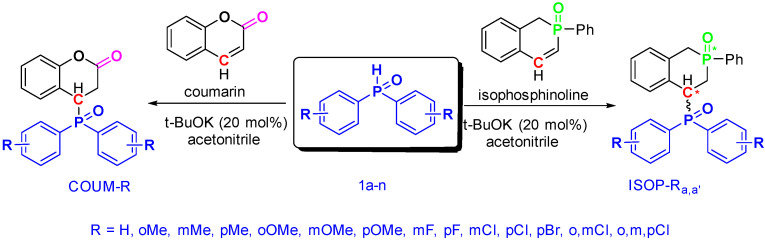
Synthesis of the coumarin COUM-R and isophosphinoline derivatives ISOP-Ra,a′.

We employed a similar reaction strategy to synthesize the target compounds COUM-R and ISOP-R, which were first described by Knochel in 2002 [21] and Zhang in 2017 [22]. Alkenyl phosphorus compounds were reacted with diphenylphosphine oxide in the presence of a catalytic amount of potassium t-butoxide as a base, in dioxane at 80 °C (Scheme 6 and Table 2).

Under optimized reaction conditions developed by one of us, acetonitrile was used as the solvent instead of dioxane. This enabled the Michael reaction to afford the corresponding coumarin derivatives in yields ranging from 58% to 97% and isophosphinoline derivatives in yields ranging from 70% to 88%.

Post-reaction analysis of the isophosphinoline derivatives by ^31^P NMR consistently revealed the presence of two diastereomers: an intracyclic phosphorus–phenyl isomer (P-intracyclic) and an extracyclic carbon–phosphorus isomer (C–P extracyclic). These exist in cis and trans configurations in different proportions. In some cases, these stereoisomers could be isolated individually, allowing us to obtain crystals of ISOP-Ha, ISOP-Hb, and ISOP-2oMea, which were subsequently characterized by X-ray crystallography. At this stage, we have not attempted to determine whether the observed isomers represent thermodynamic or kinetic products. Attempts to isomerize one stereoisomer using a catalytic amount of base did not yield conclusive results.

More details, including synthesis conditions and NMR data, can be found in the Supporting Information S1 and S2 for each molecule. References [23,24,25,26,27,28,29,30,31,32] are cited in the Supplementary Materials.

**Scheme 7:**
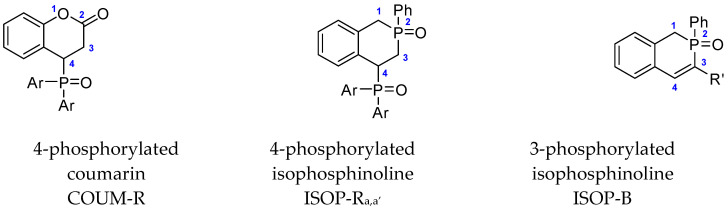
The general structure of the modified coumarin and isophosphinolines.

All the molecules synthetized from coumarin and isophosphinoline studied in this paper are divided into two groups (named group 1 and 2) and compared with a third group (group 3) of compounds that have already been studied in detail in a previous article [19] (Scheme 7). This extended comparison will allow for the identification of the key structural parameters that control flame retardancy.

Group 1 gathers 4-phosphorylated coumarins, where the substituent group is linked to carbon 4 (called modified coumarins COUM-R, with R referring to the substituent group). Group 2 gathers 4-phosphorylated isophosphinolines grafted with a group linked to carbon 4 that is not directly bound to the phosphorus atom (called modified isophosphinolines ISOP-Ra,a′, with R referring to the substituting group). Note that some molecules in this group are diastereomers or a mixture of diastereomers. Group 3 gathers isophosphinolines that are modified by grafting of a group linked to carbon 3. This carbon atom is directly bound to the phosphorus atom (called ISOB-R, with R referring to the substituting group). These molecules have already been studied in detail in a previous article [19].

All the molecules are listed in Table 3, alongside the main data obtained from TGA and PCFC analyses.

### 2.3. Characterizations

^1^H NMR, ^31^P NMR, and ^13^C NMR spectra were recorded on a BRUKER (Wissembourg, France) Ultra shield 400 plus (400.13 MHz) instrument at 25 °C. NMR samples were prepared with DMSO-d6 or CDCl3 as the solvent. The ^1^H NMR spectra were recorded with a spectral width of 8 kHz, a transmitter frequency offset of 3 kHz, and an acquisition time of 4 s, and 8 scans were performed. The ^31^P NMR spectra were recorded with a spectral width of 64 kHz, a transmitter frequency offset of 8000 Hz, and an acquisition time of 1 s, and 4 scans were performed. External references were trimethylsilane (TMS) for ^1^H and phosphoric acid (H_3_PO_4_) for ^31^P NMR. Shifts were given in ppm.

High-resolution mass spectroscopic (HRMS) analysis was performed on a Xevo G2 Q TOF spectrometer using the electrospray method by the Laboratoire de Mesures Physiques of the University of Montpellier (Montpellier, France).

Flame retardancy was assessed at the microscale using pyrolysis–combustion flow calorimetry (PCFC) and thermogravimetric analysis.

Thermogravimetric analyses (TGAs) were carried out using a TGA Q50 (TA instrument, New Castle, DE, USA) at a heating rate of 20 °C·min^−1^. A 2–8 mg sample was placed on a platinum pan and heated from room temperature to 800 °C under a nitrogen flow (60 mL·min^−1^).

Pyrolysis–combustion flow calorimetry tests were performed using a device provided by Fire Testing Technologies (FTT, UK), according to method A proposed by the ASTM D7309 standard. Briefly, a 2–5 mg sample is heated in a first chamber under a nitrogen flow (100 mL/min) at 1 K/s up to 750 °C. Pyrolytic gases are sent to a second chamber (called a combustor) at 900 °C in the presence of an excess of oxygen (N_2_/O_2_ ratio 80/20) to be fully oxidized. Heat release is calculated using the well-known Huggett relation: the consumption of 1 g of oxygen during combustion corresponds to an energy release of 13.1 kJ, whatever the material under pyrolysis [33]. The heat release rate (HRR) is plotted versus the pyrolysis temperature. The residue fraction $f_{residue}$ is measured at 750 °C, and the heat of complete combustion (∆h in kJ·g^−1^) is calculated as follows:

(1) $$∆h=\frac{THR}{{1-f}_{residue}}\;\mathrm{with}\;THR\;\mathrm{is}\;\mathrm{the}\;\mathrm{total}\;\mathrm{heat}\;\mathrm{release}\;(\mathrm{in}\;\mathrm{kJ}\cdot g^{-1})$$

Standard deviations were assessed to equal 54 W/g, 4 °C, 0.8 kJ/g, 0.01, and 1 kJ/g, respectively, for the pHRR, TpHRR, THR, residue fraction, and ∆h.

Thermogravimetric analysis and pyrolysis–combustion flow calorimetry are two useful techniques for studying decomposition behavior at a very small scale (a few mg). In most cases, both techniques may provide similar information (see Supporting Information S3, reference [34] is cited in the Supplementary Materials.) because pyrolysis occurs under the same conditions (i.e., in a nitrogen atmosphere at a constant heating rate—even if the heating rate is usually higher in PCFC at 1 K/s). This is evidenced when comparing residue contents or the temperature at the pHRR (peak of heat release rate) and at the pMLR (peak of mass loss rate) from PCFC and TGA, respectively. Nevertheless, the combination of both techniques can also provide additional information.

Since some molecules contain halogen atoms, which are well known as flame inhibitors (i.e., they disturb oxidation in the gas phase), additional analyses were conducted using PCFC-FTIR coupling [35]. Indeed, under standard conditions, combustion in PCFC is complete. Nevertheless, reducing the combustor temperature makes the combustion incomplete and allows for assessment of the ability of gases to undergo combustion under degraded conditions [4]. FTIR analysis of gases gives useful insight into the gases released under specific conditions, i.e., CO_2_, hydrogen fluoride, or chloride, but also CO, methane, and some other partially oxidized gases, revealing incomplete combustion.

In most cases, carbon released in the gas phase is fully transformed into CO_2_ when the combustor temperature is 900 °C. At 800 °C, only CO_2_ and CO are observed. Therefore, knowing the carbon content in the initial molecule and assuming the carbon content in the residue (if any), the carbon content in the gas phase can be assessed and a proportionality factor can be obtained between carbon oxidized in CO_2_ (at 900 °C) and the area of the CO_2_ band (2405–2273 cm^−1^). Indeed, according to the Beer–Lambert law, the absorbance of a species is proportional to its concentration. Knowing this factor, it is possible to calculate a similar proportionality factor for CO using data at 800 °C (CO band is observed between 1991 and 2257 cm^−1^). Therefore, CO and CO_2_ contents and their ratio can be approximately calculated using such a procedure. The proportionality factors for CO_2_ (respectively, CO) were calculated from the data for which only CO_2_ (respectively, CO and CO_2_) was observed in FTIR spectra (corresponding to 18 independent analyses). The carbon fraction in the residue was fixed arbitrarily to 0.9. This value is close to that expected from the typical composition of char as a polyaromatic compound (i.e., C_5_H_2_) [36]. In most cases, the residue content was almost negligible or very small, and this value did not significantly impact the calculation.

## 3. Results

In the first part, data from TGA and PCFC (according to method A, i.e., anaerobic pyrolysis and complete combustion) are discussed for the three groups of molecules. In the second part, some selected molecules are tested by PCFC-FTIR coupling under incomplete combustion conditions in order to assess the so-called “flame inhibition” mode-of-action.

**Figure 2:**
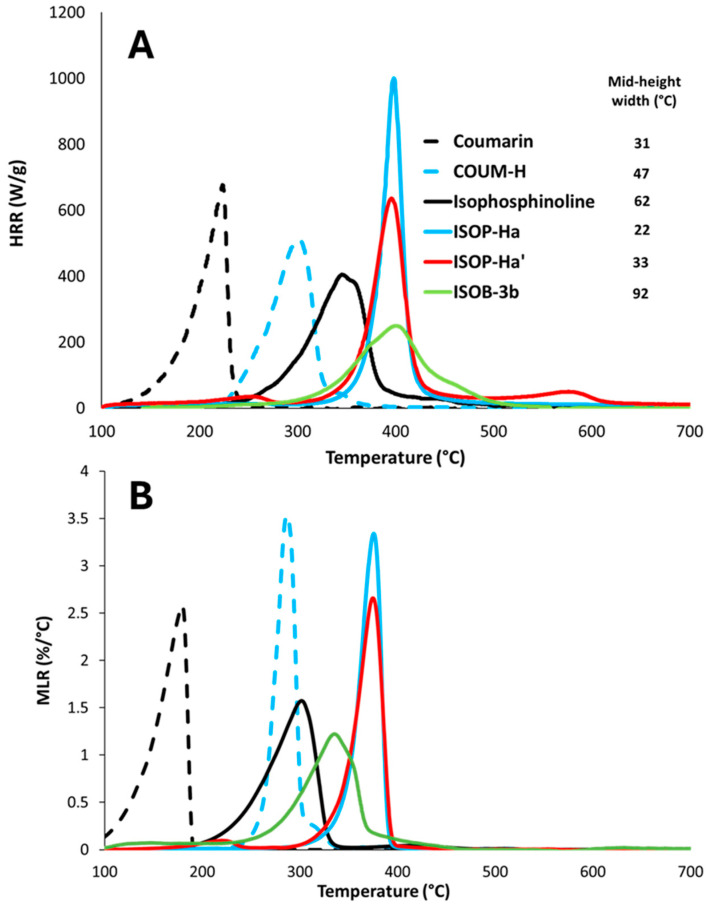
HRR curves (**A**) and DTG curves (**B**) for pure coumarin and isophosphinoline, as well as for some molecules from groups 1, 2, and 3.

Figure 2A,B show the HRR and MLR curves for pure coumarin and isophosphinoline, as well as for selected molecules from groups 1, 2, and 3 (all TGA curves can be found in the Supporting Information). These molecules are substituted by a substituent mainly containing aromatic rings, with no heteroatoms (F, Cl, Br, or P). None of them is able to produce a significant amount of char (char content < 5 wt%). The change in the curves after modification is typical for each group.

**Figure 3:**
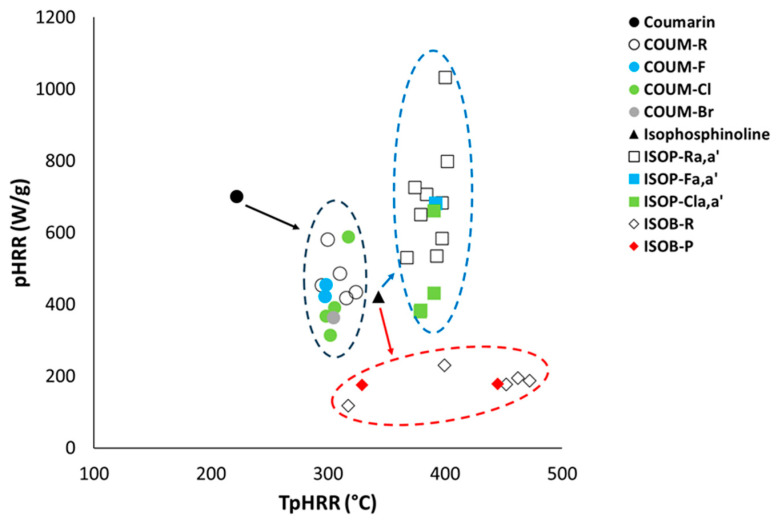
pHRR vs. TpHRR for all molecules studied (TpHRR corresponds to the temperature of the highest pHRR).

Figure 3 plots the pHRR versus the temperature at pHRR for all molecules from the three groups. For group 1, the addition of a substituting group (in all cases, aromatic groups sometimes substituted by halogenated atoms) significantly improves thermal stability. Indeed, the temperature of the peak increases from 222 °C for pure coumarin to 300 °C for COUM-H (295–324 °C depending on the substituting group). In all cases, only one apparent peak is observed. The pHRR tends to decrease slightly. Halogenated atoms (especially chlorine and bromine) reduce it to a larger extent. Even if combustion is complete under standard PCFC conditions (no flame inhibition occurs), heavy atoms like Cl and Br consume only one hydrogen atom to produce HCl or HBr. Therefore, they reduce the overall heat of combustion, and therefore the heat release rate.

For group 2, for which all substituting groups are aromatic groups, sometimes substituted with methyl or methoxy groups, fluorine atoms, or chlorine atoms, thermal stability is also strongly improved. The temperature at the main peak increases from 343 °C for pure isophosphinoline to 400 °C and 397 °C for ISOP-Ha and ISOP-Ha′, respectively (367–402 °C depending on the molecule from group 2). Decomposition occurs in one main apparent step, but very small peaks can be observed for some molecules at low or high temperature (ISOP-pCla or ISOP-o,mCla, for example). The pHRR tends to strongly increase (up to 1033 W/g for ISOP-Ha), or, at best, remains close to the value for pure isophosphinoline (422 W/g), when aromatic rings are substituted by chlorine atoms. Another observation is the width at mid-height, which indicates the temperature range in which decomposition occurs. This width is around 60 °C for pure isophosphinoline, but decreases strongly for the modified molecules of group 2 (up to 22 °C for ISOP-Ha, for example). In other words, the decomposition starts later, but when it starts, it is very fast. Aromatic groups usually improve the thermal stability of molecules. But when the temperature is high enough to allow the release of aromatic compounds, the destabilization of the molecule probably leads to its fast decomposition.

Group 3 contains molecules substituted with different chemical groups, including one bringing aromatic rings (ISOB-3b). The HRR curve is very different for all of these molecules in comparison to group 2. Molecules from group 3 show a large decomposition range, with most often two apparent pHRRs. For molecules with only one pHRR, such as ISOB-3b, the width at mid-height also increases strongly (92 °C). As the decomposition occurs over a wider range of temperatures, the pHRRs are much less intense (105–230 W/g).

Figure 2B plots the MLR versus temperature curves measured in TGA for the same molecules as shown in Figure 2A. The curve profile is the same, but the temperatures at peak are lower in TGA, because the heating rate is only 20 °C/min, versus 60 °C/min in PCFC. Nevertheless, it can be noted that the shift in temperatures at peak is different according to the molecule (compare, for example, ISOB-3b and ISOP-Ha). This may reflect some differences in the activation energy of pyrolysis. The relative intensity of peaks is usually the same in TGA and in PCFC. In other words, a high pHRR corresponds to a high pMLR. This is not surprising, as the heat of combustion ∆h of these molecules is relatively similar (remind that pHRR=pMLR×∆h). Nevertheless, this is not true for COUM-H. For this molecule, the pMLR is the most intense, while the pHRR is lower than the pHRR of coumarin, ISOP-Ha, and ISOP-Ha′. This may be attributable to a change in the pyrolysis pathway depending on the heating rate, or, alternatively, to a change in the heat of combustion during decomposition (in this case, the heat of combustion at peak may be different to the mean value).

**Figure 4:**
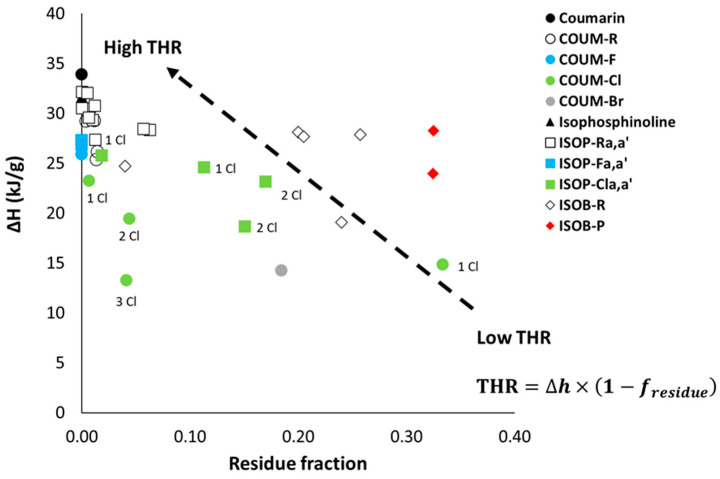
Heat of complete combustion versus residue fraction in PCFC for all molecules studied.

The heats of complete combustion of pure coumarin and isophosphinoline are high (around 30 kJ/g), while their residue fractions are null (Figure 4). Therefore, the total heat release (THR), which is a product of the mass loss fraction and heat of combustion, is also high.

The presence of halogenated atoms reduces the heat of combustion. Indeed, these atoms have a negligible contribution to heat release (in the models based on molar group contributions, their contributions can be even negative [2,3]. They lead to the release of HF, HCl, or HBr gases during pyrolysis. Moreover, some molecules are able to char. While char contains a great amount of carbon, it usually stores a huge amount of energy (typically 37.2 kJ/g with a composition close to that of C_5_H_2_, corresponding to polyaromatic compounds [36]). The gases released are poorer in carbon, and their oxidation releases less heat.

The THR can be reduced for both reasons. In the case of group 1, a decrease in the heat of combustion is observed mainly for halogen-containing molecules. This decrease is more significant for bromine (Mw = 81 g/mol) than for chlorine (Mw = 35 g/mol), and very limited for fluorine (Mw = 19 g/mol). It can also be observed that molecules containing three chlorine atoms on each aromatic ring release less heat than those containing two chlorine atoms, which also release less heat than those containing only one chlorine atom. Interestingly, COUM-2mCl releases little heat, despite the presence of only one chlorine atom. This can be ascribed to its very strong and unusual ability to produce char (residue fraction 0.33), which stores a high amount of carbon.

Under standard PCFC conditions, combustion is complete. Nevertheless, for some molecules, combustion can be incomplete even when the combustor temperature is as high as 900 °C [4]. It is noteworthy that two molecules exhibit incomplete combustion (COUM-2pBr, bearing a bromine atom, and COUM-2m,m,pCl, bearing three chlorine atoms on aromatic rings), as evidenced by FTIR-PCFC coupling (see below). For these two molecules, flame inhibition also contributed to the low total heat release.

For group 2, the ability to char is reduced (residue fraction < 0.1 in all cases). Therefore, a decrease in the heat of combustion is observed only for molecules containing chlorine atoms.

On the contrary, the heat of combustion is only slightly reduced (in the range 24–28 kJ/g) for most molecules from group 3. But these molecules often have a high ability to char, especially phosphonate-containing molecules, allowing for a significant reduction in the THR. On the contrary, ISOB-3b is the only molecule from group 3 with a negligible char content. It can be assumed that the presence of aromatic rings (as pendant groups) has a detrimental effect on char promotion.

**Figure 5:**
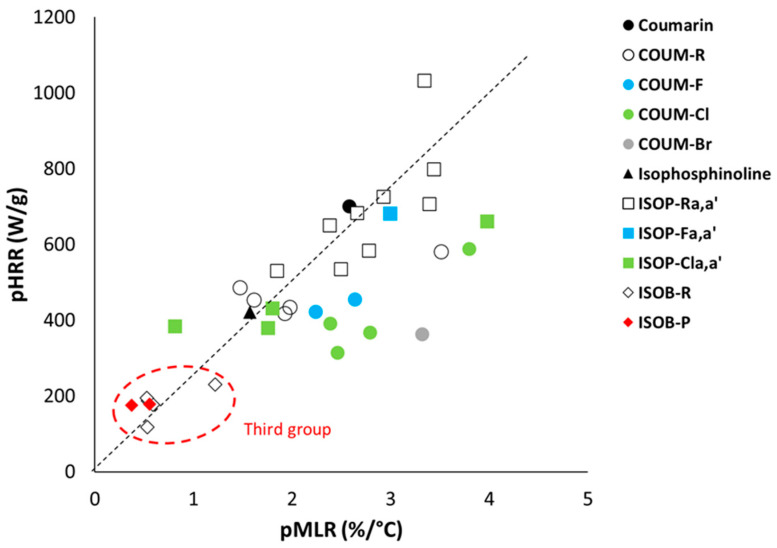
Peak of heat release rate in PCFC versus peak of mass loss rate in TGA for all molecules studied.

The ratio between the heat release rate and mass loss rate corresponds to the heat of combustion. Comparing the pHRR and pMLR, measured respectively using PCFC and TGA, is another way to assess the heat of combustion (at peak decomposition). Figure 4 plots the pHRR and the pMLR for all the molecules studied. A rather good correlation can be found for most molecules. These molecules have more or less the same heat of combustion (around 25–30 kJ/g). Nevertheless, for molecules with a lower heat of combustion, the pHRR is lower than expected from the dotted line drawn in Figure 5. This is especially true in the case of chlorine- and bromine-containing molecules, which reduce the heat of combustion due to their own low contribution, as discussed above. Among halogen-free molecules, only the molecule COUM-H from group 1 exhibits a relatively low pHRR for a quite high pMLR, as already discussed above.

Beyond the general trends discussed above, some specific features can be drawn. In the following, we highlight three points concerning, respectively, diastereomers, the role of phosphorus atoms, and the impact of halogens on combustion efficiency.

- Diastereomers

Some molecules from group 2 are diastereomers. ISOP-Ra,a′ molecules correspond to a mixture of two diastereomers. ISOP-Ra and ISOP-Ra′ constitute, after purification, only one diastereomer. Interestingly, most diastereomers exhibit different fire performances.

**Figure 6:**
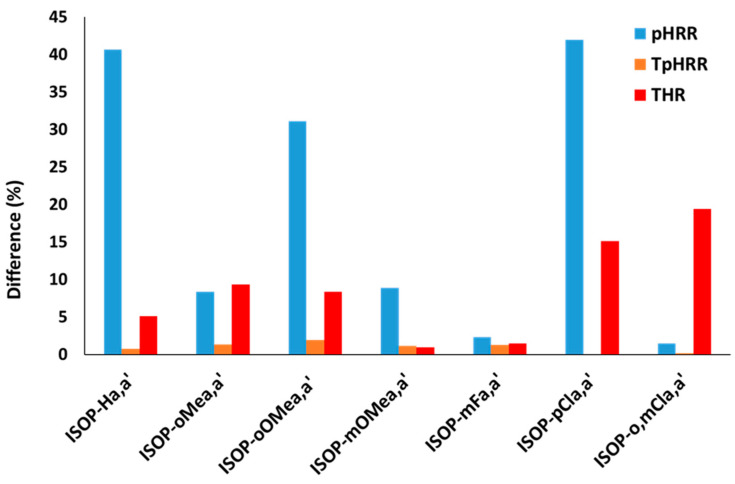
The relative difference (in %) between the pHRR, TpHRR, and THR of the two diastereomers (or mix of diastereomers).

Figure 6 shows the relative difference in the pHRR, TpHRR, and THR. The relative difference was calculated between the two diasteromers or between one diastereomer and the non-purified mixture of both (see Table 3) as follows:

$$Relative\;difference\;\left(\%\right)=100\times \frac{2\times \left|P_{a}-P_{a^{\prime }}\right|}{\left(P_{a}+P_{a^{\prime }}\right)}$$

With $P_{a}$ and $P_{a^{\prime }}$ being the properties considered for diastereomers a and a′ (or a mix of both diastereomers).

The temperature at pHRR, which corresponds to thermal stability, is similar for both diastereomers for all couples. But a huge difference is observed in the pHRR (up to 40%). For example, ISOP-Ha and ISOP-Ha′ exhibit a pHRR equal to 1033 W/g and 684 W/g, respectively (the same observation can be made for pMLR). The variation in total heat release is intermediate. Some diastereomers have the same THR, but some other ones show some differences (up to 19%). Other differences are noticeable: in particular, the char content is significantly different for the two diastereomers of ISOP-pCla,a′: 0.11 versus 0.02 in PCFC, and, similarly, 0.14 versus 0.04 in TGA.

Detailing the decomposition pathway for each molecule is beyond the scope of the present paper. Nevertheless, these results provide evidence that the precise structure of a molecule (including stereochemistry) may impact the pyrolysis, and therefore the fire behavior, of the molecule. Computational modeling may be useful in future work to address this point.

- Phosphorus

Phosphorus is a well-known flame-retardant element. Its effect depends mainly on its oxidation level [37,38]. Indeed, its low oxidation level (phosphine oxide, phosphinate) leads to its mode-of-action as a flame inhibitor, while its char promotion is poorly effective. On the contrary, char promotion is favored with phosphate or phosphonate groups. In this paper, three phosphorus groups are present. In pure isophosphinoline, phosphorus is present with a low oxidation level. In all modified isophosphinolines (from groups 2 and 3), a second phosphorus atom, once again with a low oxidation level, is present in the substituting group. In all the molecules from group 2 and one molecule from group 3, it can be observed that the phosphorus is linked to aromatic rings. Finally, in two molecules from group 3 (namely ISOB-P3d and ISOB-P3e), a phosphonate group is also present.

From the overall results, it can be concluded that the phosphorus atom with a low oxidation level is not able to improve char formation. Most molecules from group 2 and pure isophosphinoline have a residue fraction close to 0. While flame inhibition could not be properly assessed in these experiments, it can be assumed that this phosphorus atom is not effective in improving flame retardancy. The better thermal stability of isophosphinoline in comparison to coumarin may be related not to the presence of phosphorus, but rather to the presence of an additional aromatic ring.

On the contrary, if most molecules from group 3 exhibit a high char content, ISOB-P3d and ISOB-P3e have the highest value. Therefore, phosphonate groups have an influence on flame retardancy by promoting char formation.

- Halogens

Standard test conditions in PCFC do not allow for the assessment of some flame-retardant modes-of-action. In particular, flame inhibition—when a FR releases, in the gas phase, some molecules that are able to disturb the oxidation of carbon-based gases—is usually not effective, because the combustor temperature and residence time (900 °C and 10 s, respectively) are high enough to ensure complete combustion [4]. Only a few chlorine- or bromine-rich molecules can prevent combustion under such conditions. In order to study this specific mode-of-action, additional tests were performed on some molecules by reducing the combustor temperature (at 600, 700, and 800 °C) and by coupling PCFC with a FTIR spectrophotometer.

**Figure 7:**
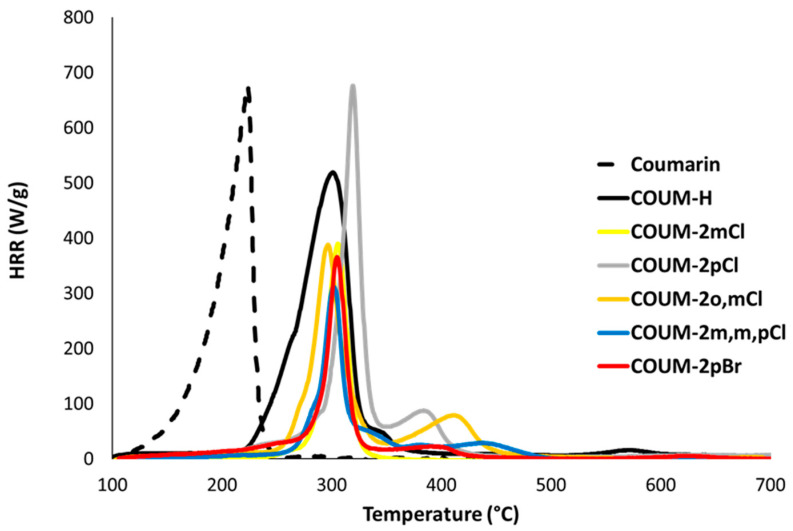
HRR curves under standard conditions (combustor temperature 900 °C) for some molecules from group 1.

**Figure 8:**
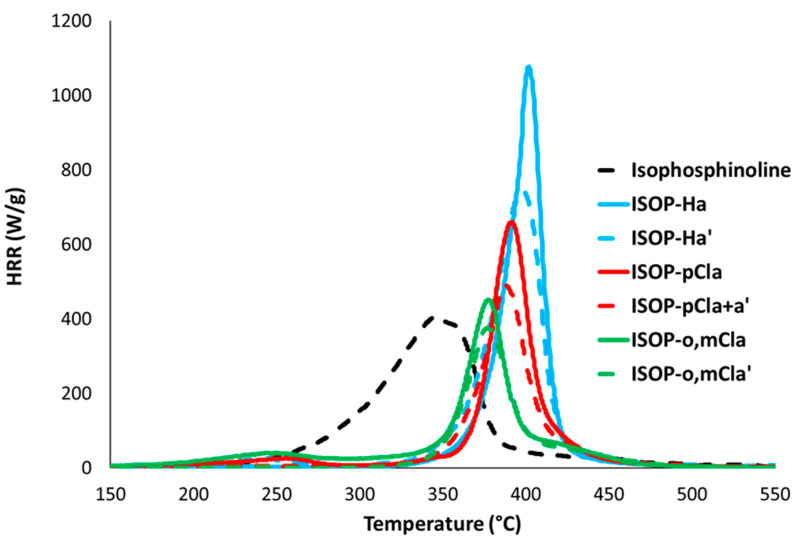
HRR curves under standard conditions (combustor temperature 900 °C) for some molecules from group 2.

**Figure 9:**
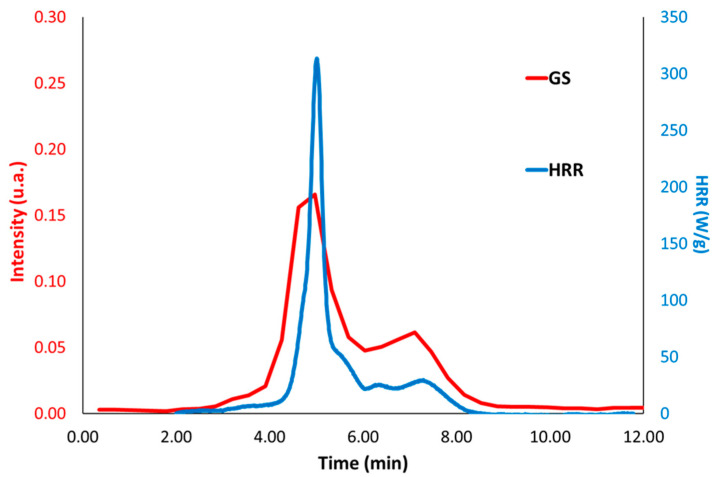
HRR and Gram–Schmidt (GS) curves for COUM-2m,m,pCl at a combustor temperature of 900 °C.

Figure 7 and Figure 8 show the HRR curves under standard conditions (i.e., combustor temperature of 900 °C) for these molecules. In most cases, decomposition occurs in one main step. Nevertheless, two peaks can be observed for COUM-2o,mCl, COUM-2pCl, COUM-2m,m,pCl, and COUM-2pBr (i.e., molecules from group 1 bearing chlorine or bromine atoms). For COUM-2o,mCl, COUM-2pCl, and COUM-2m,m,pCl, the Gram–Schmidt curve highlights the second peak, which is much more intense than in HRR curves (Figure 9). This two-step decomposition is observed neither for non-halogen and fluorine-based molecules, nor for chlorine-based molecules from group 2 (in this case, an almost negligible peak is observed at a very low temperature, close to 250 °C). Finally, for COUM-2mCl from group 1, which contains chlorine atoms, the decomposition also occurs in one step. But this molecule exhibits a specific decomposition pathway with a very high ability to char (residue fraction 0.33).

**Figure 10:**
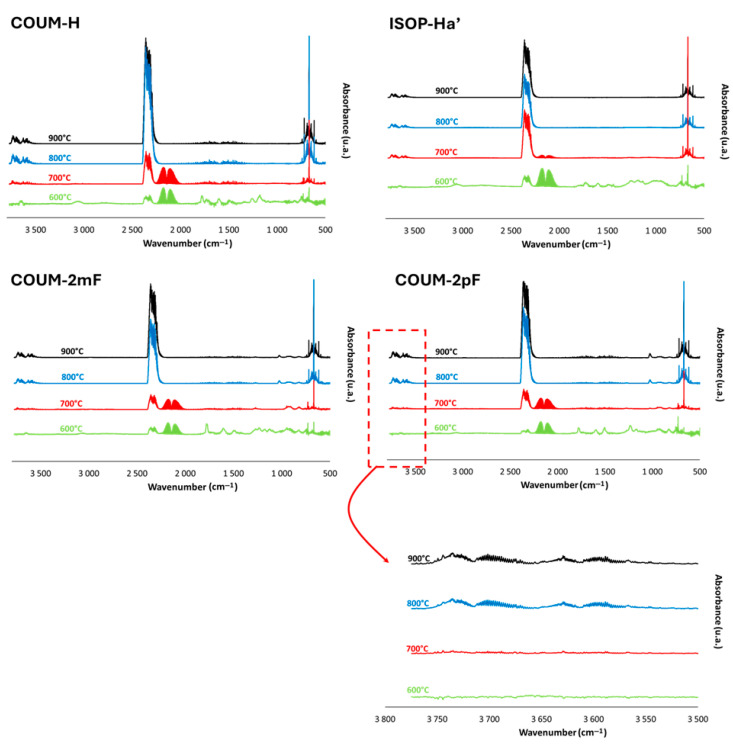
FTIR spectra at peak of Gram–Schmidt curve for COUM-H, ISOP-Ha′, COUM-2mF, and COUM-2pF at four temperatures (600, 700, 800, and 900 °C).

For non-halogen-based molecules (namely COUM-H and ISOP-Ha′), the combustion remains complete at 900 and 800 °C (Figure 10). A CO band is observed only at 700 °C, which agrees well with some previous findings [35]. At this temperature, the CO band is still limited, especially for ISOP-Ha′. At 600 °C, the CO_2_ band is strongly reduced. CO release is enhanced, and some other bands corresponding to partially oxidized or unoxidized gases can also be found, for example, methane at 3000 cm^−1^.

For fluorine-based molecules (namely COUM-2mF and COUM-2pF), the same characteristics can be highlighted (Figure 10). Combustion is complete at 900 and 800 °C and decreases at lower temperatures. The presence of other carbon-based gases can be detected at 600 °C, especially a narrow band at 1770 cm^−1^ related to C=O, which may be indicative of aromatic acid halides [39]. Interestingly, fluoride hydrogen is detected (bands around 3700 cm^−1^), but only at a high temperature. This point needs further investigation.

**Figure 11:**
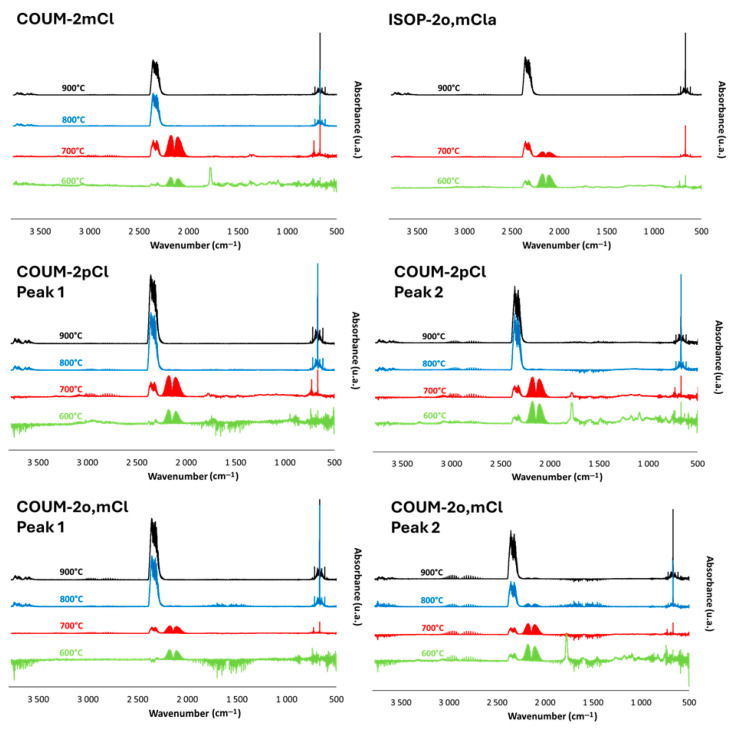
FTIR spectra at peak of Gram–Schmidt curve for COUM-2mCl, ISOP-2o,mCla, COUM-2pCl, and COUM-2o,mCl at four temperatures (600, 700, 800, and 900 °C).

**Figure 12:**
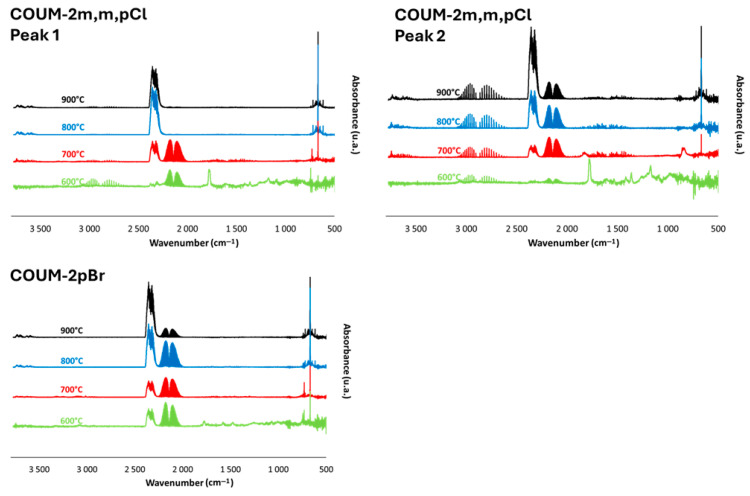
FTIR spectra at peak of Gram–Schmidt curve for COUM-2m,m,pCl and COUM-2pBr at four temperatures (600, 700, 800, and 900 °C).

FTIR spectra at the peak(s) of Gram–Schmidt curves for chlorine- and bromine-based molecules can be found in the following figures (Figure 11 and Figure 12). Combustion remains almost complete at 800 °C (a very small—negligible—CO band can be found) for molecules containing one chlorine atom on aromatic rings. On the contrary, the CO band is clearly visible at 800 °C for the other molecules. For COUM-2m,m,pCl (three chlorine on aromatic rings) and COUM-2pBr (containing a bromine atom), CO is even observed at 900 °C, evidencing that the combustion is not complete even at this high temperature.

Interestingly, at 700 °C, the CO band is the lowest (in comparison to the CO_2_ band) for ISOP-o,mCla (in comparison to COUM-2pCl or COUM-2mCl, which have only one chlorine atom on the aromatic ring). It seems that combustion decreases faster with temperature for molecules from group 1 than from group 2.

Hydrogen chloride is clearly identifiable from bands at 2700–3000 cm^−1^. Nevertheless, hydrogen bromide is not observed, maybe because its characteristic band appears in the same range as CO_2_ at 700 or 600 °C, depending on the molecule; other gases can be highlighted as methane. The band as 1770 cm^−1^ is also well visible (except for ISOP-o,mCla—for COUM-2pBr, this band appears to be reduced). It is also noteworthy that there are some differences between the spectra corresponding to the two peaks of the Gram–Schmidt curves (i.e., also to the two HRR curves) for COUM-2pCl, COUM-2o,mCl, and COUM-2m,m,pCl. In particular, at 600 °C, the band at 1770 cm^−1^ is observed only for the second step of decomposition for COUM-2pCl and COUM-2o,mCl. The relative intensity of the halogen chloride band (versus CO or CO_2_) is also enhanced during the second step. This is especially clear for COUM-2m,m,pCl.

**Figure 13:**
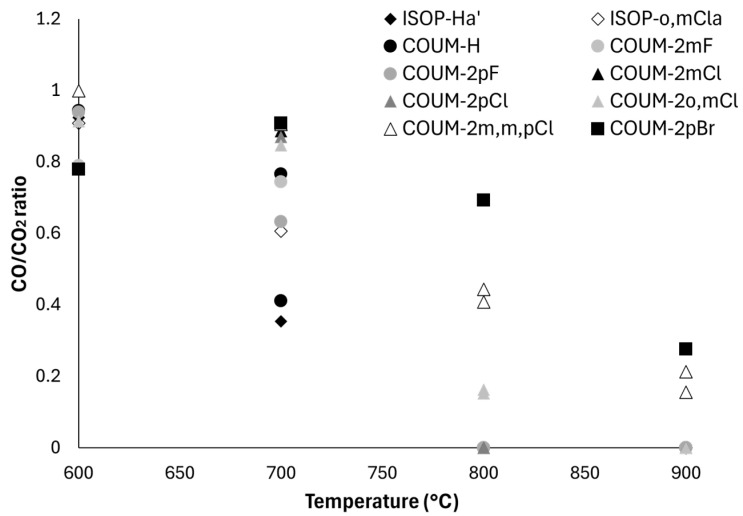
CO/CO_2_ ratio versus temperature for all molecules studied using PCFC-FTIR coupling.

Figure 13 shows the CO/CO_2_ ratio versus temperature for the studied molecules. At 900 °C, the ratio is null (no CO) for all molecules except bromine-containing COUM-2pBr and COUM-2m,m,pCl (three chlorine atoms on aromatic rings). At 800 °C, the ratio is the highest, in the following order: COUM-2pBr > COUM-2m,m,pCl > COUM-2o,mCl (two chlorine atoms). It remains null for all other molecules, even those with one chlorine atom. At 700 °C, all bromine- and chlorine-based molecules from group 1 exhibit a ratio close to 1. The ratio is lower for other molecules from group 1 (even with fluorine atoms) and even lower for molecules from group 2 (even with one chlorine atom-ISOP-o,mCla). Finally, at 600 °C, the ratio is very high. It slightly decreases for COUM-2pBr, certainly because the amount of low-oxidized gases (or soots) may strongly increase at the expense of CO and CO_2_ production. Both CO and CO_2_ production seem to be reduced. It is noteworthy that two molecules, namely COUM-H and ISOP-Ha′, contain low-oxidation-state phosphorus, but no halogen atoms. Low-oxidation-state phosphorus is known to act as a radical scavenger like halogen atoms [37,38]. Nevertheless, in the present study, the CO/CO_2_ ratio remains among the lowest for these two molecules, providing evidence that the radical scavenging seems not to be efficient in this case. Nevertheless, the possible condensation of phosphorus-based gases (between pyrolyzer and combustor) should also be considered.

The temperatures of 700 and 800 °C seem to be the best conditions for discriminating the ability of the different molecules to disturb combustion.

## 4. Conclusions

The influence of different parameters on the thermal stability and flammability of molecules is highlighted in this work. Some of the parameters are rather well known, namely the following:

- Substituting groups most often tend to increase the thermal stability of both building blocks, namely, coumarin and isophosphinoline. This effect is more prominent for coumarin. In isophosphinoline, it depends on the location of the substituting group.
- The oxidation level of phosphorus has an impact on char promotion. Phosphonates are highly efficient groups for enhancing charring, while low-oxidation-level phosphorus is unable to promote charring.
- Aromatic rings as pendant groups (and without the presence of heteroatoms) increase the peak of heat release rate and do not promote charring, especially when they are not substituted by halogen atoms. This is not surprising, and previous work has shown that these aromatic rings have a high contribution to the pHRR and THR and a negligible contribution to charring [3,18].
- Fluorine atoms have very little effect on flammability, while chlorine and bromine groups strongly reduce heat release through a decrease in the heat of combustion and char promotion (to a certain extent). Moreover, the combustion efficiency is highly reduced when the combustion temperature is low, and in some cases, even when the temperature is as high as 900 °C. Moreover, the greater the quantity of halogen atoms, the more significant this effect is.

Other parameters are new findings, or are unexpected and have not been proved yet to be influential, and need further study:

- The influence of halogen atoms as flame inhibitors is strongly dependent on the building block structure. In particular, the combustion is much more disturbed at a low temperature for coumarin than for isophosphinoline, despite the presence of a phosphorus atom in the structure of the latter.
- The location of the substituting group on isophosphinoline (on carbon 3 or 4) completely changes the decomposition pathway. In particular, the grafting of the substituting group on carbon 3 (group 3) leads to decomposition over a wide temperature range, and, consequently, a low pHRR.
- The location of halogen atoms on the aromatic ring has a huge effect on the decomposition pathway. In one case, only one chlorine atom in meta position leads to a strong charring. Such an effect is not observed when the chlorine atom is in para position or when several chlorine atoms are present.
- In some cases, diastereomers exhibit different flammability properties, mainly in terms of their pHRR.

Next steps will consist of selecting some of these molecules as FR additives or monomers in order to prepare polymeric materials and to test their fire-retardant behavior. The objective will then be to check whether the findings listed above remain suitable.

## Figures and Tables

**Table 1:** Diarylphosphine oxide synthesized compounds **1a**–**n**.

** 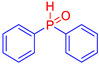 **	** 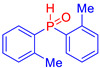 **	** 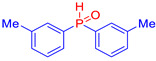 **	** 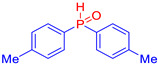 **
**1a**, 95%	**1b**, 68%	**1c**, 65%	**1d**, 76%
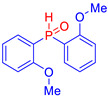	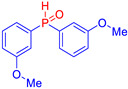	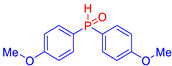	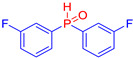
**1e**, 78%	**1f**, 82%	**1g**, Sigma-Aldrich	**1h**, 96%
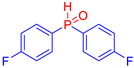	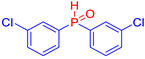	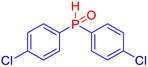	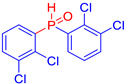
**1i**, 63%	**1j**, 85%	**1k**, 65%	**1l**, 82%
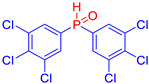	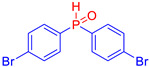		
**1m**, 54%	**1n**, 66%		

**Table 2:** Coumarin and isophosphinoline derivative synthesized compounds.

** 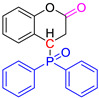 **	** 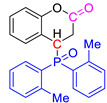 **	** 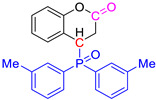 **	** 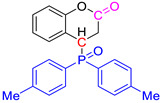 **
COUM-H, 84%	COUM-2oMe, 65%	COUM-2mMe, 80%	COUM-2pMe, 58%
** 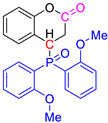 **	** 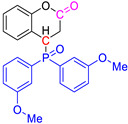 **	** 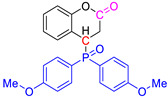 **	
COUM-2oOMe, 86%	COUM-2mOMe, 69%	COUM-2pOMe, 77%	
** 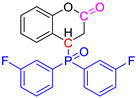 **	** 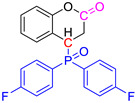 **	** 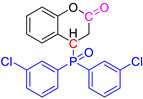 **	** 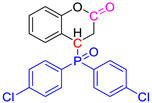 **
COUM-2mF, 95%	COUM-2pF, 97%	COUM-2mCl, 81%	COUM-2pCl, 89%
** 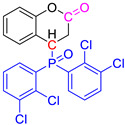 **	** 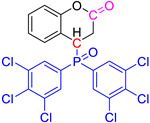 **	** 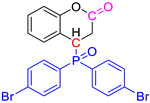 **	
COUM-2o,mCl, 96%	COUM-2m,m,pCl, 87%	COUM-2pBr, 72%	
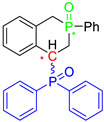	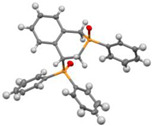	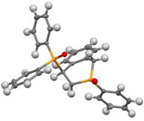	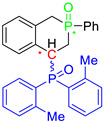
ISOP-H_a,a′_: 84%	ISOP-H_a_ (cis)	ISOP-H_a′_ (trans)	ISOP-oMe_a,a′_, 86%,
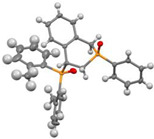	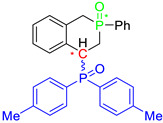	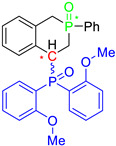	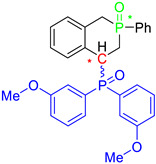
ISOP-oMe_a_ (cis)	ISOP-pMe_a,a′_, 80%	ISOP-oOMe_a,a′_, 85%	ISOP-mMe_a,a′_, 88%
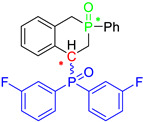	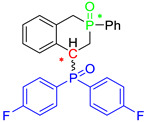	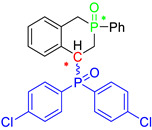	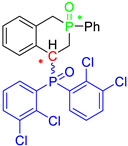
ISOP-mF_a,a′_, 80%	ISOP-pF_a,a′_, 84%	ISOP-pCl_a,a′_, 70%	ISOP-o,mCl_a,a′_, 79%

**Table 3:** Molecules studied in this work, with main data from TGA and PCFC.

Acronym	Structure	PCFC					TGA		
		PHRR (W/g)	TpHRR * (°C)	THR (kJ/g)	Residue Fraction at 750 °C (-)	Δh (kJ/g)	pMLR (%/°C)	Tdmax ** (°C)	Residue Fraction at 750 °C (-)
Coumarin	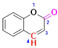	711	222	33.9	0	33.9	2.58	179	0
Isophosphinoline	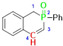	422	343	31.7	0	31.7	1.57	302	0
Group 1									
COUM-H	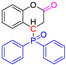	580	300	28.9	0.01	29.2	3.51	288	0.03
COUM-2oMe	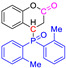	453	295	30.2	0.01	30.4	1.61	262	0.01
COUM-2pMe	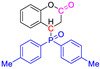	485	311	29.1	0	29.2	1.48	274	0.02
COUM-2oOMe	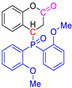	434	324	25.0	0.01	25.3	1.98	283	0.04
COUM-2mOMe	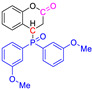	417	316	25.8	0.01	26.1	1.93	288	0.06
COUM-2mF	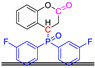	454	299	25.9	0	25.9	2.64	286	0.01
COUM-2pF	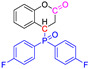	421	298	26.5	0	26.5	2.24	290	0.02
COUM-2mCl	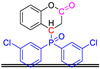	390	306	9.9	0.33	14.9	2.39	297	0.28
COUM-2pCl	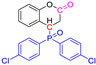	588	318	23.1	0.01	23.3	3.8	305	0.03
COUM-2o,m,Cl	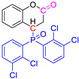	367	299	18.6	0.04	19.5	2.79	286	0.03
COUM-2m,mpCl	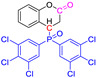	313	302	12.7	0.04	13.2	2.46	291	0.03
COUM-2pBr	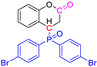	363	305	11.6	0.18	14.2	3.32	300	0.12
Group 2									
ISOP-H_a_ (cis)	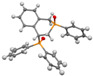	1033	400	30.6	0	30.6	3.34	376	0.02
ISOP-H_a′_ (trans)	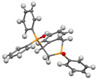	684	397	32.2	0	32.2	2.66	375	0.02
ISOP-oMe_a_ (cis)	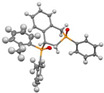	707	384	29.1	0.01	29.4	3.39	362	0.01
ISOP-oMe_a′_ (trans)	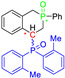	651	379	31.9	0.01	32.1	2.38	356	0.01
ISOP-pMe_a,a′_	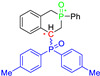	800	402	30.5	0.01	30.8	3.44	381	0.01
ISOP-oOMe_a_	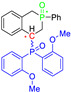	726	374	29.4	0.01	29.6	2.92	356	0.01
ISOP-oOMe_a′_	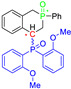	531	367	27.1	0.01	27.4	1.85	343	0.02
ISOP-mOMe_a_	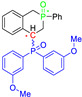	585	397	26.6	0.06	28.4	2.77	372	0.04
ISOP-mOMe_a′_	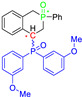	535	393	26.9	0.06	28.4	2.49	372	0.06
Mixture ISOP-mF_a_ + ISOP-mF_a′_	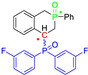	697	387	27.7	0.02	28.2			
ISOP-mF_a′_	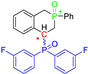	681	392	27.3	0	27.3	3.0	396	0.03
Mixture ISOP-pF_a_ + ISOP-pF_a′_	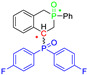	749	398	26.5	0.02	27.0			
ISOP-pCl_a_	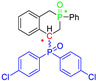	660	391	25.3	0.02	25.8	3.98	373	0.04
Mixture ISOP-pCl_a_ + ISOP-pCl_a′_	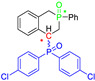	431	391	21.8	0.11	24.6	1.81	390	0.14
ISOP-o,mCl_a_	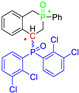	384	380	19.2	0.17	23.1	0.82	377	0.23
ISOP-o,mCl_a′_	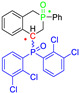	378	379	15.8	0.15	18.6	1.76	363	0.12
Group 3									
ISOB-3b	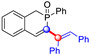	230	400	23.8	0.04	24.7	1.22	334	0.04
ISOB-3f	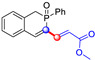	188	472	20.7	0.26	27.9	0.54	451	0.22
ISOB-3ja	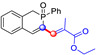	118	317	14.5	0.24	19.1	0.53	299	0.23
ISOB-3h	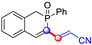	177	452	22.5	0.20	28.1	0.59	420	0.14
ISOB-3g	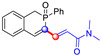	195	462	22.0	0.21	27.7	0.53	358	0.21
ISOB-P3e	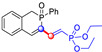	175	329	19.1	0.32	28.3	0.37	407	0.28
ISOB-P3d	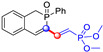	178	445	16.2	0.32	24.0	0.56	410	0.33

* TpHRR = Temperature at pHRR. ** Tdmax = Temperature at pMLR.

## Data Availability

Data are available upon request.

## Supplementary Materials

The following supporting information can be downloaded at https://www.mdpi.com/article/10.3390/molecules30183717/s1, Figure S1a: ORTEP-style plot of the molecular structure of ISOP-Ha (cis) with atomic displacement parameters ellipsoids at the 50% probability level; Figure S1b: ORTEP-style plot of the molecular structure of ISOP-Ha′ (trans) with atomic displacement parameters ellipsoids at the 50% probability level; Figure S1c: ORTEP-style plot of the molecular structure of ISOP-oMea (cis) with atomic displacement parameters ellipsoids at the 50% probability level; Figure S2: 31P{1H}, 1H and 13C{1H} NMR and HRMS spectra of the synthesized compounds; Figure S3a: Residue fraction in TGA versus residue fraction in PCFC for all molecules studied; Figure S3b: Temperature at maximum decomposition rate in TGA versus temperature at pHRR in PCFC for all molecules studied; Table S1: Crystal structure data.
