# Supplementary material for: Synthesis and Comparison of the Flame-Retardant Properties of Phosphorylated-Coumarins and Phosphorylated-Isophosphinolines
Source: Molecules. 2025 Sep 12;30(18):3717. doi: 10.3390/molecules30183717 (PMC12472271; doi:10.3390/molecules30183717)
Supplement: Supplementary file 1 [file molecules-30-03717-s001.zip › molecules-3810346-supplementary.pdf]

Synthesis and Comparison of the flame-retardant properties of phosphorylated-coumarins and phosphorylated-isophosphinolines

Issaka Ibrahim Abdou Rachid[1,2,†], Karen-Pacelye Mengue Me Ndong [1,3,†], Abdoul Razak Halidou Dougourikoye [2], Mina Hariri [1,4], Gabin Mwande-Maguene [3], Jacques Lebibi [3], Fatemeh Darvish [4], Ilagouma Amadou Tidjani [2], David Virieux [1], Jean-Luc Pirat [1], Tahar Ayad [1,*], Loic Dumazert [5], Arie van der Lee [6], Claire Negrell [1] and Rodolphe Sonnier [5,*]

[1] ICGM, Univ Montpellier, ENSCM, CNRS, 34090 Montpellier, France; abdrachid2i@gmail.com (I.I.A.R.); guemenkaren@gmail.com (K.-P.M.M.N.); pirat@enscm.fr (J.-L.P.)
[2] University Abdou Moumouni of Niamey, Niamey 10896, Niger
[3] Université des Sciences et Techniques de Masuku, Franceville 942, Gabon; gabin.maguene@gmail.com (G.M.-M.)
[4] Department of Chemistry, K. N. Toosi University of Technology, Tehran 19991-43344, Iran
[5] Polymers Composites and Hybrids (PCH), IMT Mines Ales, 30319 Ales, France
[6] IEM, University Montpellier, ENSCM, CNRS, 34095 Montpellier, France
* Correspondence: authors: tahar.ayad@enscm.fr (T.A.); rodolphe.sonnier@mines-ales.fr (R.S.)
† These authors contributed equally to this work.

Supporting information

S1 - Experimental Data

Characterizations

NMR analyses

^{1}H NMR spectra were recorded on a BRUKER Ultra shield 400 plus (400.13 MHz) instrument. The chemical shifts are expressed in parts per million (ppm) referenced to residual $CDCl_3$ (7.27 ppm), DMSO-d_6 (2.50 ppm), D_2O (4.79 ppm), acetone-d_6 (2.05 ppm). Coupling constants are expressed in Hertz (Hz). These abbreviations are used to express the multiplicity: s (singulet), d (doublet), t (triplet), q (quadruplet), quint (quintuplet), m (multiplet).

^{31}P{1H} NMR spectra were recorded on the same instrument at 161.99 MHz. The chemical shifts are expressed in parts per million (ppm). These abbreviations are used to express the multiplicity: s (singulet), d (doublet).

^{13}C{H} NMR spectra were recorded on the same instrument at 100.6 MHz. The chemical shifts are expressed in parts per million (ppm), reported from the central of $CDCl_3$ ($CDCl_3$, 77.16 ppm), DMSO-d_6 ($(CD_3)_2SO$, 39.52 ppm). Coupling constants are expressed in Hertz (Hz). These abbreviations are used to express the multiplicity: s (singulet), d (doublet).

X-ray data collection and structure refinement

Crystal screening and intensity measurements for the three compounds ISOP-H_a (cis), ISOP-$H_{a'}$ (trans), and ISOP-oMe$_a$ (cis) were performed at 173°K on a Bruker Venture diffractometer equipped with a Cu-$K\alpha$ microsource and a Photon-II detector. The APEX3 program was used for data collection, and the SAINT, XPREP, and SADABS routines were respectively utilized for integration of the data using default parameters, empirical absorption correction using spherical harmonics employing symmetry-equivalent and redundant data, and correction for Lorentz and polarization effects [1].

All three crystal structures were solved with the *ab initio* iterative charge-flipping method with parameters described elsewhere [2], by using the SUPERFLIP program [3]. All structural models were refined against $|F|^2$ using full-matrix non-linear least-squares procedures as implemented in CRYSTALS on all independent reflections with $I > -3\sigma(I)$ [4]. Hydrogen atoms were refined with riding constraints in all three structures. One of the rings in ISOP-oMe$_a$ (cis) was found to contain unresolved disorder. Geometrical restraints were used to force the ring geometry to have generally accepted bond distances and angles.

Crystallographic parameters and basic information pertaining to data collection and structure refinement are summarized in Table S1.

CCDC 2393228 contains the supplementary crystallographic data for ISOP-H_a (cis), 2393229 those for ISOP-$H_{a'}$ (trans), and 2393230 those for ISOP-oMe$_a$ (cis). These data can be obtained free of charge from The Cambridge Crystallographic Data Centre via www.ccdc.cam.ac.uk/structures.

Table S1: Crystal structure data

	ISOP-H_a (cis)	ISOP-$H_{a'}$ (trans)	ISOP-oMe$_a$ (cis)
formula	$C_{27}H_{24}O_2P_2$	$C_{27}H_{24}O_2P_2$	$C_{29}H_{28}O_2P_2$
moiety	$C_{27}H_{24}O_2P_2$	$C_{27}H_{24}O_2P_2$	$C_{29}H_{28}O_2P_2$
T (K)	173	173	173

spacegroup	*P*-1	$P2_1/c$	$Pna2_1$
crystal system	triclinic	monoclinic	orthorhombic
a (Å)	8.8933(2)	11.5226(3)	14.1592(7)
b (Å)	10.1097(3)	11.1426(3)	19.9385(10)
c (Å)	12.9373(3)	17.8897(5)	8.5717(4)
α (°)	90.1061(8)	90	90
β (°)	105.6226(7)	103.4397(7)	90
γ (°)	95.5920(8)	90	90
V (Å^3)	1114.39(5)	2233.99(10)	2419.9(2)
Z	2	4	4
ρ (gcm^{-3})	1.318	1.315	1.291
M_r (gmol^{-1})	442.40	442.40	470.45
μ (mm^{-1})	1.940	1.936	1.817
R_{int}	0.047	0.051	0.000
Θ_{max} (°)	72.357	72.566	72.589
resolution (Å)	0.81	0.81	0.81
N_{tot} (measured)	32269	41240	4794
N_{ref} (unique)	4392	4419	4794
N_{ref} (I>2$\sigma(I)$)	4173	4218	4689
N_{ref} (least-squares)	4392	4418	4793
N_{par}	280	281	299
$<\sigma(I)/I>$	0.0302	0.0288	0.031
R_1 (I>2$\sigma(I)$)	0.0335	0.0370	0.0510
wR_2 (I>2$\sigma(I)$)	0.0848	0.1006	0.1339
R_1 (all)	0.0360	0.0379	0.0518
wR_2 (all)	0.0868	0.0868	0.1014
GOF	1.0026	0.8195	1.0061
$\Delta\rho$ (eÅ^{-3})	-0.38/0.45	-0.35/0.43	-0.38/0.99
crystal size (mm^3)	0.05x0.10x0.15	0.05x0.12x0.15	0.06x0.11x0.15

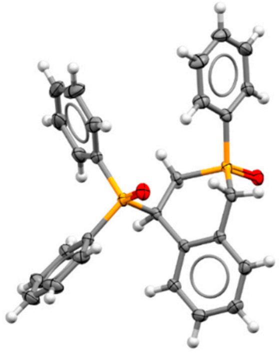

Figure S1a: ORTEP-style plot of the molecular structure of ISOP-H_a (cis) with atomic displacement parameters ellipsoids at the 50% probability level.

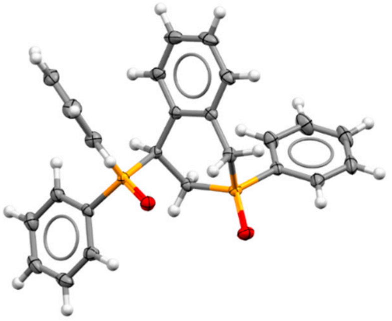

Figure S1b: ORTEP-style plot of the molecular structure of ISOP-$H_{a'}$ (trans) with atomic displacement parameters ellipsoids at the 50% probability level.

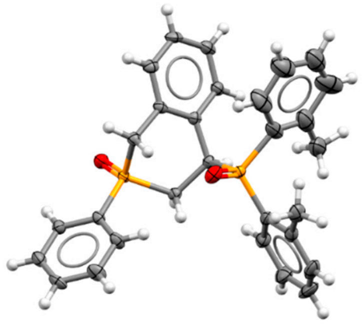

Figure S1c: ORTEP-style plot of the molecular structure of ISOP-oMe$_a$ (cis) with atomic displacement parameters ellipsoids at the 50% probability level.

General procedures

Synthesis of secondary diarylphosphine oxide

The first section focuses on the preparation of diversely substituted secondary diarylphosphine oxides, which bear either electron-withdrawing groups (EWGs) or electron-donating groups (EDGs) on the aryl halides. The modified diarylphosphine oxides 1a-n were efficiently synthesized with good yields through a two-step reaction sequence following established literature methods [5].

General Procedure for the Synthesis of Diarylphosphine oxide 1a-n [6]

In a three-neck round-bottom flask**,** equipped with a reflux condenser, was charged 1 equivalent of crushed magnesium scraps (31 mmol, 753 mg) and 3 mL of anhydrous tetrahydrofuran (THF) to immerse the magnesium scraps. Then, 1 equivalent of haloarene (31 mmol) diluted in 17 mL of anhydrous THF was added dropwise via dropping funnel under nitrogen atmosphere. After the completion of dropping, the reaction was warmed at 45°C for 0.5−1.5 h.

Then, the flask was cooled to 0°C in an ice-water bath, and 1 equivalent of diethyl phosphite (10 mmol, 1.29 mL) was slowly added. After the completion of dropping, the mixture was stirred at room temperature (monitored by TLC). Then, diluted 1M HCl (20 mL) was added, at 0°C and the reaction was stirred for 30 min. The mixture was extracted three times with ethyl

acetate (3 x 20 mL), the organic phase was washed with brine (10 mL), dried over Na_2SO_4, filtered, then concentrated in vacuo. The crude product was isolated by column chromatography (silica gel 40-63 μm, eluent; petroleum ether/ethyl acetate 10:90), to give the desired product 1. Only the ^{31}P and ^{1}H NMR spectra of diarylphosphine oxide were carried out and compared with the corresponding products already described in the literature.

Diphenylphosphine oxide 1a. [7]

Magnesium turnings (31 mmol, 753 mg), bromobenzene (31 mmol, 3,25 mL), diethyl phosphite (10 mmol, 1.29 mL) THF 20 mL. Diphenylphosphine oxide was isolated as a white powder with a yield of 95% (1.92 g).

$^{31}P\{1H\}$ NMR (162 MHz, Chloroform-*d*) δ 21.47. ^{1}H NMR (400 MHz, Chloroform-*d*) δ 8.08 (d, *J* = 480.7 Hz, 1H), 7.75 – 7.67 (m, 4H), 7.61 – 7.55 (m, 2H), 7.54 – 7.49 (m, 4H).

Di-o-tolylphosphine oxide 1b. [8]

Magnesium turnings (31 mmol, 753 mg), 2-bromotoluene (31 mmol, 3.7 mL), diethyl phosphite (10 mmol, 1.29 mL), THF 20 mL. Di-o-tolylphosphine oxide was isolated as a white powder with a yield of 68% (1.26 g).

$^{31}P\{1H\}$ NMR (162 MHz, Chloroform-*d*) δ 17.64. ^{1}H NMR (400 MHz, Chloroform-*d*) δ 8.20 (d, *J* = 476.2 Hz, 1H), 7.71 (ddd, *J* = 15.2, 7.6, 1.5 Hz, 2H), 7.46 (tt, *J* = 7.6, 1.5 Hz, 2H), 7.32 (tt, *J* = 7.6, 1.8 Hz, 2H), 7.28-7.17 (m, 3H), 2.37 (s, 6H).

Di-m-tolylphosphine oxide 1c.

Magnesium turnings (31 mmol, 753 mg), 3-bromotoluene (31 mmol, 3.7 mL), diethyl phosphite (10 mmol, 1.29 mL), THF 20 mL. Di-o-tolylphosphine oxide was isolated as a white powder with a yield of 65% (1.56 g).

$^{31}P\{1H\}$ NMR (162 MHz, Chloroform-*d*) δ 22.08. ^{1}H NMR (400 MHz, Chloroform-*d*) δ 8.00 (d, *J* = 479.0 Hz, 1H), 7.57 – 7.53 (m, 2H), 7.53 – 7.49 (m, 2H), 7.46 (dd, *J* = 5.7, 1.0 Hz, 1H), 7.45 – 7.40 (m, 1H), 7.36 (ddd, *J* = 5.4, 2.8, 0.9 Hz, 2H), 2.37 (s, 6H).

Di-p-tolylphosphine oxide 1d.

Magnesium turnings (3.1 eq, 753 mg), 4-bromotoluene (31 mmol, 3.8 mL), diethyl phosphite (10 mmol, 1.29 mL), THF 20 mL. Di-p-tolylphosphine oxide was isolated as a white powder with a yield of 76% (1.75 g).

$^{31}P\{1H\}$ NMR (162 MHz, Chloroform-*d*) δ 21.56. ^{1}H NMR (400 MHz, Chloroform-*d*) δ 8.03 (d, *J* = 477.0 Hz, 1H), 7.68-7.48 (m, 4H), 7.33-7.16 (m, 4H), 2.40 (d, *J* = 0.9 Hz, 6H).

Bis(2-methoxyphenyl)phosphine oxide 1e. [9]

Magnesium turnings (3.1 eq, 753 mg), 2-bromoanisole (31 mmol, 3.86 mL), diethyl phosphite (10 mmol, 1.28 mL), THF 20 mL. Bis(2-methoxyphenyl)phosphine oxide was isolated as a white powder with a yield of 78% (2.04 g).

$^{31}P\{1H\}$ NMR (162 MHz, Chloroform-*d*) δ 18.29 (d, *J* = 1.7 Hz). ^{1}H NMR (400 MHz, Chloroform-*d*) δ 8.24 (d, *J* = 514.3 Hz, 1H), 7.67 (ddd, *J* = 15.0, 7.5, 1.8 Hz, 2H), 7.49 (dddd, *J* = 8.3, 7.2, 1.8, 0.9 Hz, 2H), 7.05 (tdd, *J* = 7.4, 2.1, 0.8 Hz, 2H), 6.89 (ddd, *J* = 8.4, 5.7, 0.9 Hz, 2H), 3.75 (s, 6H).

Bis(3-metoxyphenyl) phosphine oxide 1f.

Magnesium turnings (3.1 eq, 753 mg), 3- bromoanisole (31 mmol, 3.86 mL), diethyl phosphite (10 mmol, 1.28 mL), THF 20 mL. Bis(3-methoxyphenyl)phosphine oxide was isolated as a white powder with a yield of 82% (2.16 g).

^{31}P{1H} NMR (162 MHz, Chloroform-*d*) δ 21.51. ^{1}H NMR (400 MHz, Chloroform-*d*) δ 7.98 (d, *J* = 482.7 Hz, 1H), 7.37 – 7.30 (m, 2H), 7.25 – 7.12 (m, 4H), 7.06 – 7.01 (m, 1H), 3.77 (s, 6H).

Bis(4-methoxyphenyl)phosphine oxide 1g.

Magnesium turnings (3.1 eq, 753 mg), 4-bromoanisole (3.1 eq, 4.2 mL), diethyl phosphite (10 mmol, 1.28 mL), THF 20 mL. Bis(4-methoxyphenyl)phosphine oxide was isolated as a white powder with a yield of 88% (2.3 g).

^{31}P{H} NMR (162 MHz, $CDCl_3$) δ 20.61 (s). ^{1}H NMR (400 MHz, $CDCl_3$) δ 8.59 (s, 1H), 7.63 – 7.47 (m, 4H), 6.96 (dd, *J* = 8.8, 2.1 Hz, 4H), 3.81 (s, 6H).

Bis(3-fluorophenyl)phosphine oxide 1h.

Magnesium turnings (3.1 eq, 753 mg), 1-bromo-3-fluorobenzene (3.1 eq, 3.3 mL), diethyl phosphite (10 mmol, 1.28 mL), THF 20 mL. Bis(3-fluorophenyl)phosphine oxide was isolated as a white powder with a yield of 96% (2.3 g).

^{31}P{H} NMR (162 MHz, Chloroform-*d*) δ 18.35. RMN ^{1}H (400 MHz, Chloroform-*d*) δ 8.07 (d, *J* = 491.1 Hz, 1H), 7.51 (m, 4H), 7.39 (m, 2H), 7.29 (m, 2H).

Bis(4-fluorophenyl)phosphine oxide 1i.

Magnesium turnings (3.1 eq, 753 mg), 1-bromo-4-fluorobenzene (3.1 eq, 3.3 mL), diethyl phosphite (10 mmol, 1.28 mL), THF 20 mL. Bis(4-fluorophenyl)phosphine oxide was isolated as a white powder with a yield of 63% (1.5 g).

^{31}P{H} NMR (162 MHz, Chloroform-*d*) δ 18.75

δ 18.74. ^{1}H NMR (400 MHz, Chloroform-*d*) δ 8.06 (d, *J* = 485.1 Hz, 1H), 7.68 (ddd, *J* = 13.2, 8.8, 5.4 Hz, 4H), 7.19 (td, *J* = 8.7, 2.1 Hz, 4H).

Bis(3-chlorophenyl)phosphine oxide 1j.

Magnesium turnings (3.1 eq, 753 mg), 1-bromo-3-chlorobenzene (3.1 eq, 3.3 mL), diethyl phosphite (10 mmol, 1.28 mL), THF 20 mL. Bis(3-chlorophenyl)phosphine oxide was isolated as a white powder with a yield of 85% (2.3 g).

^{31}P{H} NMR (162 MHz, Chloroform-*d*) δ 18.31. ^{1}H NMR (400 MHz, Chloroform-*d*) δ 8.03 (d, *J* = 490.5 Hz, 1H), 7.70 – 7.67 (m, 1H), 7.66 – 7.64 (m, 1H), 7.59 (dt, *J* = 7.4, 1.3 Hz, 1H), 7.56 (q, *J* = 1.6 Hz, 1H), 7.54 (t, *J* = 1.6 Hz, 2H), 7.49 – 7.42 (m, 2H).

Bis(4-chlorophenyl)phosphine oxide 1k.

Magnesium turnings (3.1 eq, 753 mg), 1-bromo-4-chlorobenzene (3.1 eq, 3.8 mL), diethyl phosphite (10 mmol, 1.28 mL), THF 20 mL. Bis(4-chlorophenyl)phosphine oxide was isolated as a white powder with a yield of 65% (1.75 g).

^{31}P{1H} NMR (162 MHz, Chloroform-*d*) δ 18.80. ^{1}H NMR (400 MHz, Chloroform-*d*) δ 8.06 (d, *J* = 486.6 Hz, 1H), 7.71-7.56 (m, 4H), 7.50 (dq, *J* = 8.6, 2.1 Hz, 4H).

Bis(2,3-dichlorophenyl)phosphine oxide 1l.

Magnesium turnings (3.1 eq, 753 mg), 1-bromo-2,3-dichlorobenzene (3.1 eq, 3.97 mL), diethyl phosphite (10 mmol, 1.28 mL), THF 20 mL. Bis(2,3-dichlorophenyl)phosphine oxide was isolated as a white powder with a yield of 82% (2.8 g).

^{31}P{1H} NMR (162 MHz, Chloroform-*d*) δ 16.01. RMN ^{1}H (400 MHz, Chloroform-*d*) δ 8.06 (d, *J* = 497.0 Hz, 1H), 7.77 (dd, *J* = 13.7, 1.7 Hz, 2H), 7.62 (dd, *J* = 8.1, 3.0 Hz, 2H), 7.50 (ddd, *J* = 13.0, 8.1, 1.8 Hz, 2H).

Bis(3,4,5-trichlorophenyl)phosphine oxide 1m. [10]

In a three-neck round-bottom flask under nitrogen, equipped with a reflux condenser, at -20 °C to 3,4,5 trichlorobromobenzene (1.56 g, 60 mmol) in anhydrous THF solution (40 mL) was added iPrMgCl*LiCl (56 mL of THF solution) dropwise. After 3 hours, to the solution was slowly added dropwise at 0°C a solution of diethyl phosphite (10 mmol, 1.8 mL) in anhydrous THF (6mL). The reaction was quenched with ammonium chloride solution. The solid precipitate was removed by filtration and the filtrate was extracted with ethyl acetate, washed with saturated sodium chloride solution and dried with $MgSO_4$. The filtrate was extracted with ethyl acetate, washed with saturated sodium chloride solution and dried with $MgSO_4$. All solvents were removed by evaporation and the pure product was obtained by rapid column chromatography on silica gel (PE/EA = 1:1) with a yield of 54% (1.3 g).

^{31}P{1H} NMR (162 MHz, Chloroform-*d*) δ 14.23. ^{1}H NMR (400 MHz, Chloroform-*d*) δ 8.01 (d, *J* = 503.1 Hz, 1H), 7.70 (s, 2H), 7.67 (s, 2H).

Bis(4-bromophenyl)phosphine oxide 1n.

Magnesium turnings (3.1 eq, 753 mg), 1-bromo-4-chlorobenzene (3.1 eq, 3.8 mL), diethyl phosphite (10 mmol, 1.28 mL), THF 20 mL. Bis(4-bromophenyl)phosphine oxide was isolated as a white powder with a yield of 66% (2.4 g).

^{31}P{1H} NMR (162 MHz, Chloroform-*d*) δ 19.15. ^{1}H NMR (400 MHz, Chloroform-*d*) δ 8.03 (d, *J* = 487.1 Hz, 1H), 7.66 (dd, *J* = 8.5, 2.3 Hz, 4H), 7.55 (dd, *J* = 13.3, 8.5 Hz, 4H).

Synthesis of 4-phosphorylated coumarins COUM-R derivatives and 3-phosphorylated isophosphinolines ISOP-Ra,a' derivatives.

General procedure for preparation of functionalized 4-(diphenylphosphoryl)chroman-2-one COUM-R

4-(diphenylphosphoryl)chroman-2-one COUM-H

Product COUM-H was prepared following the general protocol, using coumarin (200 mg, 1.37 mmol, 1 eq), diphenylphosphine oxide (1a, 305 mg, 1.51 mmol, 1.1 eq), and potassium tert-butoxide (31 mg, 20 mol %). Product COUM-H was isolated as a white powder with a yield of 84% (402 mg).

^{31}P{1H} NMR (162 MHz, DMSO-*d*$_6$) δ 32.13. ^{1}H NMR (400 MHz, DMSO-*d*$_6$) δ 8.14 – 7.95 (m, 2H), 7.80 – 7.42 (m, 8H), 7.32 – 7.17 (m, 1H), 7.05 (d, *J* = 8.0 Hz, 1H), 6.82 (t, *J* = 7.5 Hz, 1H), 6.69 – 6.58 (m, 1H), 4.64 (t, *J* = 7.0 Hz, 1H), 3.35 (ddd, *J* = 32.6, 16.6, 7.9 Hz, 1H), 2.59 – 2.44 (m, 1H). ^{13}C{H} NMR (101 MHz, Chloroform-*d*) δ 211.7 (d, *J* = 71.6 Hz), 132.8, 131.9 (d, *J* = 8.8 Hz), 131.7 (d, *J* = 8.9 Hz), 129.5, 129.2 (d, *J* = 11.6 Hz), 128.7 (d, *J* = 11.7 Hz),

124.0, 117.8, 38.0 (d, J = 65.8 Hz), 29.0 (d, J = 2.1 Hz). HRMS (ESI, M + H^+): m/z calcd for $C_{21}H_{18}O_3P$: 349.098612, found: 349.098807.

4-(di-o-tolylphosphoryl)chroman-2-one COUM-2oMe

Product COUM-2oMe was prepared following the general protocol, using coumarin (200 mg, 1.37 mmol, 1 eq), bis(2-methylphenyl)phosphine oxide (1b, 348 mg, 1.51 mmol, 1.1 eq), and potassium tert-butoxide (31 mg, 20 mol %). Product COUM-2oMe was isolated as a white powder with a yield of 65% (336 mg).

$^{31}P\{1H\}$ NMR (162 MHz, DMSO-d_6) δ 37.96. ^1H NMR (400 MHz, Chloroform-d) δ 7.58 (dd, J = 7.7, 1.4 Hz, 1H), 7.47 (td, J = 7.6, 1.4 Hz, 1H), 7.38 – 7.31 (m, 2H), 7.30 – 7.25 (m, 1H), 7.22 (ddd, J = 8.3, 7.4, 1.6 Hz, 2H), 7.15 – 7.08 (m, 1H), 7.05 (ddd, J = 8.2, 6.5, 1.0 Hz, 2H), 6.75 (td, J = 7.5, 1.3 Hz, 1H), 6.32 (d, J = 7.6 Hz, 1H), 4.04 (dd, J = 7.6, 1.8 Hz, 1H), 3.41 (dd, J = 16.6, 1.8 Hz, 1H), 3.18 (dd, J = 16.6, 7.5 Hz, 1H), 2.33 (s, 3H), 2.05 (s, 3H). $^{13}C\{H\}$ NMR (101 MHz, Chloroform-d) δ 165.9 (d, J = 3.1 Hz), 153.3 (d, J = 4.6 Hz), 144.1 (dd, J = 7.4, 4.8 Hz), 133.1 (d, J = 10.2 Hz), 132.4 (d, J = 2.6 Hz), 132.1 (d, J = 2.8 Hz), 131.8 (t, J = 11.3 Hz), 130.5 (d, J = 10.2 Hz), 129.7 (d, J = 4.0 Hz), 129.3 (d, J = 3.1 Hz), 128.7 (d, J = 46.3 Hz), 126.1 – 125.4 (m), 123.7 (d, J = 2.7 Hz), 117.5 (d, J = 2.6 Hz), 117.1 (d, J = 5.0 Hz), 36.7 (d, J = 66.3 Hz), 30.0 (d, J = 3.2 Hz), 21.3 (d, J = 4.6 Hz), 20.7 (d, J = 3.3 Hz). HRMS (ESI, M + H^+): m/z calcd for $C_{23}H_{22}O_3P$: 377.1301, found: 377.1305

4-(di-m-tolylphosphoryl)chroman-2-one COUM-2mMe

Product COUM-2mMe was prepared following the general protocol, using coumarin (200 mg, 1.37 mmol, 1 eq), bis(3-methylphenyl)phosphine oxide (1c, 348 mg, 1.51 mmol, 1.1 eq), and potassium tert-butoxide (31 mg, 20 mol %). Product COUM-2mMe was isolated as a white powder with a yield of 80 % (410 mg).

$^{31}P\{1H\}$ NMR (162 MHz, DMSO-d_6) δ 31.61. ^1H NMR (400 MHz, Chloroform-*d*) δ ^1H NMR (400 MHz, Chloroform-*d*) δ 7.68 (dt, J = 11.2, 0.9 Hz, 1H), 7.62 – 7.54 (m, 4H), 7.48 – 7.38 (m, 2H), 7.36 – 7.30 (m, 1H), 7.26 – 7.20 (m, 1H), 7.00 (dt, J = 8.2, 1.0 Hz, 1H), 6.87 (tt, J = 7.5, 1.0 Hz, 1H), 6.64 (dt, J = 7.7, 1.9 Hz, 1H), 3.90 (ddd, J = 10.3, 8.0, 1.9 Hz, 1H), 3.19 (ddd, J = 16.8, 9.6, 1.9 Hz, 1H), 2.97 (ddd, J = 29.7, 16.8, 8.0 Hz, 1H), 2.41 (s, 3H), 2.29 (s, 3H). $^{13}C\{H\}$ NMR (101 MHz, Chloroform-*d*) δ 165.7 (d, J = 3.0 Hz), 152.7 (d, J = 4.6 Hz), 139.2 (d, J = 11.4 Hz), 138.6 (d, J = 11.4 Hz), 133.5 (dd, J = 7.3, 2.9 Hz), 132.5 (dd, J = 24.2, 8.5 Hz), 129.6 (d, J = 96.7 Hz), 129.6 (d, J = 4.0 Hz), 129.4 (d, J = 3.0 Hz), 128.9 (d, J = 12.4 Hz), 128.8 (d, J = 97.8 Hz), 128.7 (d, J = 9.3 Hz), 128.5, 128.4 (d, J = 3.0 Hz), 123.9 (d, J = 2.6 Hz), 117.7 (d, J = 2.5 Hz), 117.5 (d, J = 5.0 Hz), 37.8 (d, J = 65.6 Hz), 28.9 (d, J = 2.2 Hz), 21.5 (d, J = 16.6 Hz). HRMS (ESI, M + H^+): m/z calcd for $C_{23}H_{22}O_3P$: 377. 377.1301, found : 377.1304

4-(di-p-tolylphosphoryl)chroman-2-one COUM-2pMe

Product COUM-2pMe was prepared following the general protocol, using coumarin (200 mg, 1.37 mmol, 1 eq), bis(4-methylphenyl)phosphine oxide (1d, 348 mg, 1.51 mmol, 1.1 eq), and potassium tert-butoxide (31 mg, 20 mol %). Product COUM-2pMe was isolated as a white powder with a yield of 58% (300 mg).

$^{31}P\{1H\}$ NMR (162 MHz, DMSO-d_6) δ 32.45. ^1H NMR (400 MHz, Chloroform-d) δ 7.61 (dd, J = 7.7, 1.4 Hz, 1H), 7.49 (td, J = 7.5, 1.3 Hz, 1H), 7.40 – 7.34 (m, 2H), 7.32 – 7.28 (m, 1H), 7.24 (ddd, J = 8.3, 7.5, 1.6 Hz, 2H), 7.17 – 7.11 (m, 1H), 7.08 (ddd, J = 8.2, 6.5, 1.0 Hz, 2H), 6.77 (td, J = 7.5, 1.3 Hz, 1H), 6.35 (d, J = 7.6 Hz, 1H), 4.07 (dd, J = 7.6, 1.8 Hz, 1H), 3.43 (dd, J = 16.6, 1.8 Hz, 1H), 3.21 (dd, J = 16.6, 7.5 Hz, 1H), 2.35 (s, 3H), 2.07 (s, 3H). $^{13}C\{H\}$ NMR (101 MHz, DMSO-d_6) δ 166.0, 152.4 (d, J = 4.4 Hz), 146.0 – 139.9 (m), 131.0 (d, J = 10.3 Hz), 130.8, 129.6 (d, J = 12.0 Hz), 129.0 (d, J = 11.9 Hz), 128.7 (d, J = 3.0 Hz), 128.3 (d, J = 31.8

Hz), 127.3 (d, $J = 36.1$ Hz), 118.2 (d, $J = 5.7$ Hz), 116.7 (d, $J = 2.6$ Hz), 35.4 (d, $J = 65.4$ Hz), 28.4, 21.1. HRMS (ESI, M + H^+) : m/z calcd for $C_{23}H_{22}O_3P$: 377.1301, found : 377.1300

4-(bis(2-methoxyphenyl)phosphoryl)chroman-2-one COUM-2oOMe

Product COUM-2oOMe was prepared following the general protocol, using coumarin (200 mg, 1.37 mmol, 1eq), bis(2-methoxyphenyl)phosphine oxide (1e, 398 mg, 1.51 mmol, 1.1 eq), and potassium tert-butoxide (31 mg, 20 mol %). Product COUM-2oOMe was isolated as a white powder with a yield of 86% (480 mg).

$^{31}P\{1H\}$ NMR (162 MHz, DMSO-d_6) δ 36.11. ^1H NMR (400 MHz, Chloroform-d) δ 7.62 (dd, $J = 7.6$, 1.8 Hz, 1H), 7.55 – 7.39 (m, 3H), 7.15 – 7.04 (m, 2H), 7.01 – 6.79 (m, 6H), 4.55 (dd, $J = 9.1$, 2.1 Hz, 1H), 3.77 (s, 3H), 3.67 (s, 3H), 3.26 (dd, $J = 17.6$, 2.1 Hz, 1H), 3.06 (dd, $J = 17.5$, 9.0 Hz, 1H). $^{13}C\{H\}$ NMR (101 MHz, Chloroform-d) δ 166.7, 160.1 (dd, $J = 32.1$, 3.7 Hz), 152.0 (d, $J = 5.3$ Hz), 135.3, 134.6 (d, $J = 6.8$ Hz), 134.2, 134.0, 129.0 (dd, $J = 73.1$, 3.8 Hz), 123.9 (d, $J = 2.9$ Hz), 121.1 (dd, $J = 11.4$, 2.3 Hz), 117.0 (d, $J = 2.8$ Hz), 110.8 (dd, $J = 6.7$, 2.9 Hz), 55.4 (d, $J = 3.6$ Hz), 38.2 (d, $J = 69.2$ Hz), 29.4. HRMS (ESI, M + H^+): m/z calcd for $C_{23}H_{22}O_5P$: 409.1199, found: 409.1195

4-(bis(3-methoxyphenyl)phosphoryl)chroman-2-one COUM-2mOMe

Product COUM-2mOMe was prepared following the general protocol, using coumarin (200 mg, 1.37 mmol, 1eq), bis(2-methoxyphenyl)phosphine oxide (1f, 396 mg, 1.51 mmol, 1.1 eq), and potassium tert-butoxide (31 mg, 20 mol %). Product COUM-2mOMe was isolated as a white powder with a yield of 69% (387 mg).

$^{31}P\{1H\}$ NMR (162 MHz, DMSO-d_6) δ 31.60. ^1H NMR (400 MHz, Chloroform-*d*) δ 7.71 – 7.38 (m, 8H), 7.20 (d, $J = 8.6$ Hz, 2H), 7.05 (t, $J = 7.5$ Hz, 1H), 6.81 (d, $J = 7.6$ Hz, 1H), 4.03 (d, $J = 11.4$ Hz, 4H), 3.87 (s, 3H), 3.34 (d, $J = 16.7$ Hz, 1H), 3.15 (dd, $J = 16.8$, 7.9 Hz, 1H). $^{13}C\{H\}$ NMR (101 MHz, Chloroform-d) δ 165.7, 160.2, 152.8, 131.2 (d, $J = 62.4$ Hz), 130.3 (d, $J = 13.9$ Hz), 129.8 (d, $J = 14.1$ Hz), 129.6, 129.4, 124.0, 123.6 (d, $J = 9.2$ Hz), 123.4 (d, $J = 9.1$ Hz), 119.3, 118.9, 117.8, 117.4 (d, $J = 5.2$ Hz), 116.8 (d, $J = 9.6$ Hz), 116.5 (d, $J = 9.3$ Hz), 55.6 (d, $J = 12.9$ Hz), 37.9 (d, $J = 66.1$ Hz), 29.0. HRMS (ESI, M + H^+): m/z calcd for $C_{23}H_{22}O_5P$: 409.119890, found: 409.19937

4-(bis(4-methoxyphenyl)phosphoryl)chroman-2-one COUM-2pOMe

Product COUM-2pOMe was prepared following the general protocol, using coumarin (200 mg, 1.37 mmol, 1 eq), bis(4-methoxyphenyl)phosphine oxide (1g, 396 mg, 1.51 mmol, 1.1 eq), and potassium tert-butoxide (31 mg, 20 mol %). Product COUM-2pOMe was isolated as a white powder with a yield of 77 % (430 mg).

$^{31}P\{1H\}$ NMR (162 MHz, DMSO-d_6) δ 31.14. ^1H NMR (400 MHz, Chloroform-d) δ 7.71 (d, $J = 8.9$ Hz, 2H), 7.41 (d, $J = 8.7$ Hz, 2H), 7.23 (ddd, $J = 8.2$, 7.4, 1.6 Hz, 1H), 7.01 (d, $J = 8.8$ Hz, 3H), 6.89 (d, $J = 8.8$ Hz, 3H), 6.68 (dd, $J = 7.7$, 1.7 Hz, 1H), 3.87 (s, 3H), 3.83 (m, 4H), 3.25 (dd, $J = 16.8$, 1.7 Hz, 1H), 2.95 (dd, $J = 16.8$, 8.1 Hz, 1H). $^{13}C\{H\}$ NMR (101 MHz, Chloroform-d) δ 165.8 (d, $J = 2.9$ Hz), 163.0 (dd, $J = 6.8$, 2.9 Hz), 152.6 (d, $J = 4.6$ Hz), 133.9 (d, $J = 10.1$ Hz), 133.6 (d, $J = 10.3$ Hz), 129.7 (d, $J = 4.0$ Hz), 129.3 (d, $J = 3.0$ Hz), 124.0 (d, $J = 2.7$ Hz), 121.1 (d, $J = 104.3$ Hz), 119.6 (d, $J = 104.6$ Hz), 117.7, 114.7, 114.2 (d, $J = 12.6$ Hz), 55.5 (d, $J = 8.3$ Hz), 38.4 (d, $J = 66.7$ Hz), 28.9 (d, $J = 2.1$ Hz). HRMS (ESI, M + H^+): m/z calcd for $C_{23}H_{22}O_5P$: 409.119937, found: 409.120414.

4-(bis(3-fluorophenyl)phosphoryl)chroman-2-one COUM-2mF

Product COUM-2mF was prepared following the general protocol, using coumarin (200 mg, 1.37 mmol, 1eq), bis(2-fluorophenylphosphine oxide (1h, 360 mg, 1.51 mmol, 1.1 eq), and

potassium tert-butoxide (31 mg, 20 mol %). Product COUM-2mF was isolated as a white powder with a yield of 95% (502 mg).

^{31}P{1H} NMR (162 MHz, DMSO-d_6) δ 30.77 (d, J = 24.0 Hz). ^{1}H NMR (400 MHz, DMSO-d_6) δ 7.99 – 7.84 (m, 3H), 7.82 – 7.68 (m, 1H), 7.59 (dddd, J = 16.7, 14.6, 7.6, 4.0 Hz, 3H), 7.45 (ddd, J = 9.7, 7.4, 2.3 Hz, 1H), 7.31 – 7.21 (m, 1H), 7.09 (dd, J = 8.3, 1.2 Hz, 1H), 6.90 (t, J = 7.5 Hz, 1H), 6.76 – 6.64 (m, 1H), 4.77 (t, J = 7.0 Hz, 1H), 3.45 (ddd, J = 33.9, 16.8, 7.9 Hz, 1H), 2.64 – 2.53 (m, 1H). ^{13}C{H} NMR (101 MHz, DMSO-d_6) δ 165.8 (d, J = 2.8 Hz), 163.2 (dd, J = 46.4, 16.4 Hz), 160.8 (dd, J = 45.9, 16.5 Hz), 152.5 (d, J = 4.6 Hz), 134.1 – 133.35 (m), 132.6 (dd, J = 13.4, 5.6 Hz), 131.8 (dd, J = 13.0, 7.7 Hz), 131.2 (dd, J = 13.5, 7.6 Hz), 129.2 (dd, J = 22.1, 3.7 Hz), 127.3 (dd, J = 8.0, 3.8 Hz), 123.6 (d, J = 2.9 Hz), 120.5 – 119.1 (m), 117.6 (dd, J = 10.4, 2.7 Hz), 117.3, 116.9 (d, J = 2.8 Hz), 35.1 (d, J = 66.6 Hz), 28.3 (d, J = 3.2 Hz). HRMS (ESI, M + H$^+$): m/z calcd for $C_{21}H_{16}F_2O_3P$: 385.0800, found: 385.0793

4-(bis(4-fluorophenyl)phosphoryl)chroman-2-one COUM-2pF

Product COUM-2pF was prepared following the general protocol, using coumarin (200 mg, 1.37 mmol, 1eq), bis(2-fluorophenylphosphine oxide (1i, 360 mg, 1.51 mmol, 1.1 eq), and potassium tert-butoxide (31 mg, 20 mol %). Product COUM-2pF was isolated as a white powder with a yield of 97% (514 mg).

^{31}P{1H} NMR (162 MHz, DMSO-d_6) δ 31.52. ^{1}H NMR (400 MHz, DMSO-d_6) δ 8.08 (d, J = 5.9 Hz, 2H), 7.77 (d, J = 6.0 Hz, 2H), 7.49 (t, J = 8.6 Hz, 2H), 7.33 (t, J = 8.6 Hz, 2H), 7.23 (d, J = 7.8 Hz, 1H), 7.06 (d, J = 8.2 Hz, 1H), 6.87 (t, J = 7.5 Hz, 1H), 6.64 (d, J = 7.6 Hz, 1H), 4.67 (s, 1H), 3.62 – 3.38 (m, 1H), 2.75 – 2.52 (m, 1H). ^{13}C{H} NMR (101 MHz, DMSO-d_6) δ 165.9, 167.9 – 162.6 (m), 152.5 (d, J = 4.5 Hz), 134.5 – 133.6 (m), 129.4 (d, J = 4.2 Hz), 128.9 (d, J = 3.1 Hz), 127.7 – 125.9 (m), 123.5 (d, J = 2.8 Hz), 117.7, 116.8 (d, J = 2.7 Hz), 116.5 (dd, J = 21.5, 12.6 Hz), 115.9 (dd, J = 21.4, 12.8 Hz), 35.5 (d, J = 66.5 Hz), 28.3 (d, J = 3.0 Hz). HRMS (ESI, M + H$^+$): m/z calcd for $C_{21}H_{16}F_2O_3P$: 385.0800, found: 385.0799

4-(bis(3-chlorophenyl)phosphoryl)chroman-2-one COUM-2mCl

Product COUM-2mCl was prepared following the general protocol, using coumarin (200 mg, 1.37 mmol, 1eq), bis(3-chlorophenylphosphine oxide (1j, 409 mg, 1.51 mmol, 1.1 eq), and potassium tert-butoxide (31 mg, 20 mol %). Product COUM-2mCl was isolated as a white powder with a yield of 81% (460 mg).

^{31}P{1H} NMR (162 MHz, DMSO-d_6) δ 30.92. ^{1}H NMR (400 MHz, DMSO-d_6) δ 8.08 (dt, J = 11.1, 1.7 Hz, 1H), 8.03 – 7.93 (m, 1H), 7.81 – 7.63 (m, 5H), 7.54 (td, J = 7.8, 3.4 Hz, 1H), 7.33 – 7.21 (m, 1H), 7.08 (dd, J = 8.3, 1.2 Hz, 1H), 6.90 (t, J = 7.5 Hz, 1H), 6.69 (dt, J = 7.8, 2.0 Hz, 1H), 4.80 (t, J = 6.9 Hz, 1H), 3.44 (ddd, J = 34.1, 16.8, 7.9 Hz, 1H), 2.59 – 2.52 (m, 1H). ^{13}C{H} NMR (101 MHz, DMSO-d_6) δ 165.8, 152.6, 134.4 (d, J = 14.7 Hz), 133.7 (d, J = 15.4 Hz), 133.3, 132.7, 132.4, 131.4 (d, J = 11.9 Hz), 130.8 (d, J = 12.5 Hz), 130.3 (t, J = 9.9 Hz), 129.9 – 129.5 (m), 129.4, 129.2, 123.7, 117.3 (d, J = 6.1 Hz), 117.0, 35.0 (d, J = 66.2 Hz), 28.3. HRMS (ESI, M + H$^+$): m/z calcd for $C_{21}H_{16}Cl_2O_3P$: 417.0209, found: 417.0200.

4-(bis(4-chlorophenyl)phosphoryl)chroman-2-one COUM-2pCl

Product COUM-2pCl was prepared following the general protocol, using coumarin (200 mg, 1.37 mmol, 1eq), bis(2-fluorophenylphosphine oxide (1k, 409 mg, 1.51 mmol, 1.1 eq), and potassium tert-butoxide (31 mg, 20 mol %). Product COUM-2pCl was isolated as a white powder with a yield of 89% (511 mg).

^{31}P{1H} NMR (162 MHz, DMSO-d_6) δ 32.58. ^{1}H NMR (400 MHz, DMSO-d_6) δ 7.17 (dd, J = 10.5, 8.5 Hz, 2H), 7.00 – 6.82 (m, 4H), 6.72 (dd, J = 8.5, 2.1 Hz, 2H), 6.40 (t, J = 7.8 Hz, 1H),

6.22 (d, J = 8.0 Hz, 1H), 6.04 (t, J = 7.4 Hz, 1H), 5.83 (d, J = 7.6 Hz, 1H), 3.86 (t, J = 6.8 Hz, 1H), 2.67 – 2.53 (m, 1H), 1.71 (dd, J = 16.8, 9.6 Hz, 1H). $^{13}C\{H\}$ NMR (101 MHz, DMSO-d_6) δ 165.8 (d, J = 2.6 Hz), 137.6 (dd, J = 26.6, 3.3 Hz), 132.9 (d, J = 10.2 Hz), 129.8, 129.4 (d, J = 12.2 Hz), 129.1, 128.7 (d, J = 12.3 Hz), 117.5 (d, J = 5.9 Hz), 116.9, 35.2 (d, J = 66.4 Hz), 28.3. HRMS (ESI, M + H^+): m/z calcd for $C_{21}H_{16}Cl_2O_3P$: 417.0200, found: 417.0209.

4-(bis(2,3-dichlorophenyl)phosphoryl)chroman-2-one COUM-2o,mCl

Product COUM-2o,mCl was prepared following the general protocol, using coumarin (200 mg, 1.37 mmol, 1eq), bis(2,3-dichlorophenylphosphine oxide (1l, 513 mg, 1.51 mmol, 1.1 eq), and potassium tert-butoxide (31 mg, 20 mol %). Product COUM-2o,mCl was isolated as a white powder with a yield of 96 % (641 mg).

$^{31}P\{1H\}$ NMR (162 MHz, DMSO-d_6) δ 30.57. ^{1}H NMR (400 MHz, DMSO-d_6) δ 8.26 (dd, J = 10.8, 1.7 Hz, 1H), 8.11 – 7.88 (m, 3H), 7.79 (dd, J = 8.2, 2.9 Hz, 1H), 7.70 (ddd, J = 10.2, 8.2, 1.8 Hz, 1H), 7.30 – 7.23 (m, 1H), 7.09 (dd, J = 8.2, 1.2 Hz, 1H), 6.93 (t, J = 7.5 Hz, 1H), 6.77 – 6.68 (m, 1H), 4.83 (t, J = 7.0 Hz, 1H), 3.46 (ddd, J = 34.8, 16.9, 7.9 Hz, 1H), 2.72 – 2.54 (m, 1H). $^{13}C\{H\}$ NMR (101 MHz, DMSO-d_6) δ 165.7 (d, J = 2.7 Hz), 152.5 (d, J = 4.6 Hz), 136.0 (dd, J = 38.9, 2.9 Hz), 132.6, 132.5 (d, J = 3.4 Hz), 132.1, 131.9, 131.8, 131.5, 131.3, 131.2 (d, J = 9.4 Hz), 131.0, 130.5 (d, J = 5.8 Hz), 129.6 – 129.1 (m), 123.8, 35.0 (d, J = 67.0 Hz), 28.1. HRMS (ESI, M + H^+): m/z calcd for $C_{21}H_{14}Cl_4O_3P$: 484.9429, found: 484.9413

4-(bis(3,4,5-trichlorophenyl)phosphoryl)chroman-2-one COUM-2m,m,pCl

Product COUM-2m,m,pCl was prepared following the general protocol, using coumarin (100 mg, 0;68 mmol, 1eq), bis(3,4,5-trichlorophenylphosphine oxide (1m, 306,57 mg, 0;75 mmol, 1.1 eq), and potassium tert-butoxide (15.35 mg, 20 mol %). Product COUM-2m,m,pCl was isolated as a white powder with a yield of 87 % (330 mg).

$^{31}P\{1H\}$ NMR (162 MHz, Chloroform-d) δ 27.44. ^{1}H NMR (400 MHz, Chloroform-d) δ 7.80 (d, J = 10.8 Hz, 2H), 7.56 – 7.31 (m, 3H), 7.12 (d, J = 8.3 Hz, 1H), 7.01 (t, J = 7.6 Hz, 1H), 6.65 (d, J = 7.7 Hz, 1H), 3.91 (d, J = 8.3 Hz, 1H), 3.40 – 3.02 (m, 2H). $^{13}C\{H\}$ NMR (101 MHz, Chloroform-d) δ 164.9 (d, J = 69.2 Hz), 131.1 (d, J = 9.7 Hz), 130.9 (d, J = 9.9 Hz), 130.7 (d, J = 3.4 Hz), 129.2, 124.6, 118.5, 37.8 (d, J = 68.5 Hz), 28.8. HRMS (ESI, M + H^+): m/z calcd for $C_{21}H_{12}Cl_6O_3P$: 552.8650, found: 552.8648.

4-(bis(4-bromophenyl)phosphoryl)chroman-2-one COUM-2pBr

The title product COUM-2pBr was prepared according to the general protocol using coumarin (200 mg, 1.37 mmol, 1eq), bis-(4-bromophenyl)phosphine oxide (1n, 513 mg, 1.51 mmol, 1.1eq) and Potassium tert-butoxide (31 mg, 20 mol %). The product COUM-2pBr was isolated as a white powder in 72 % (250 mg).

$^{31}P\{1H\}$ NMR (162 MHz, Chloroform-d) δ 30.53. ^{1}H NMR (400 MHz, Chloroform-d) δ 7.76 – 7.61 (m, 4H), 7.56 (dd, J = 8.5, 2.5 Hz, 2H), 7.36 (dd, J = 10.8, 8.5 Hz, 2H), 7.27 (d, J = 5.8 Hz, 1H), 7.04 (dd, J = 8.2, 1.1 Hz, 1H), 6.98 – 6.86 (m, 1H), 6.62 (dt, J = 7.8, 1.9 Hz, 1H), 3.88 (dd, J = 10.3, 7.9 Hz, 1H), 3.16 (ddd, J = 16.7, 9.8, 1.7 Hz, 1H), 3.01 (ddd, J = 31.1, 16.8, 7.9 Hz, 1H). $^{13}C\{H\}$ NMR (101 MHz, Chloroform-d) δ 165.3, 133.3 (d, J = 9.6 Hz), 133.0 (d, J = 9.8 Hz), 132.6 (d, J = 11.9 Hz), 132.2 (d, J = 12.1 Hz), 129.7 (d, J = 49.6 Hz), 128.4, 124.3, 118.0, 37.94 (d, J = 67.0 Hz), 28.9. HRMS (ESI, M + H^+): m/z calcd for $C_{21}H_{16}Br_2O_3P$: 504.9198, found: 504.9195.

General procedure for preparation of 4-(diarylphosphoryl)-2-phenyl-1,3,4-trihydroisophosphinoline 2-oxide ISOPa-i, ISOPa'-i'.

Under nitrogen, in a microwave tube was added isophosphinoline (0.75 mmol, 1 eq.), the phosphorus compound 1 (0.825 mmol, 1.1 eq.), potassium tert-butoxide (0,15 mmol, 20 mol%) and 2.5 mL of acetonitrile.

The reaction mixture was heated under microwave irradiation at 80°C during 15 minutes. Then, after evaporation of the solvent, a diluted solution of 1M HCl (5 mL) was added to the mixture, the aqueous layer was separated and extracted with 5 mL of DCM three times. The combined organic layers were washed with water, dried over anhydrous magnesium sulfate, filtered, and then concentrated under reduced pressure.

4-(diphenylphosphoryl)-2-phenyl-1,3,4-trihydroisophosphinoline 2-oxide ISOP-Ha,a'

Products ISOP-$H_{a,a'}$ were prepared following the general protocol, using isophosphinoline (120 mg, 0.5mmol, 1 eq), diphenylphosphine oxide 1a (111 mg, 0,55 mmol, 1,1 eq) and potassium tert-butoxide (11 mg, 0,1 mmol, 20 mol%) in 2.5 mL of acetonitrile. Products ISOP-H_a, ISOP-$H_{a'}$ were isolated as a white powder with a yield of 84% (185 mg, ISOP-H_a 20 mg, ISOP-$H_{a'}$ 165 mg).

ISOP-Ha

$^{31}P\{1H\}$ NMR (162 MHz, Chloroform-*d*) δ 36.73 (d, *J* = 14.4 Hz), 35.79 (d, *J* = 14.4 Hz). ^{1}H NMR (400 MHz, Chloroform-*d*) δ 7.97 (dddd, *J* = 14.6, 10.6, 7.9, 1.9 Hz, 4H), 7.68 – 7.39 (m, 9H), 7.39 – 7.28 (m, 2H), 7.22 – 7.10 (m, 2H), 6.92 (t, *J* = 7.4 Hz, 1H), 6.77 (dt, *J* = 7.8, 1.6 Hz, 1H), 4.34 (dd, *J* = 15.1, 8.7 Hz, 1H), 4.22 – 4.03 (m, 1H), 3.19 (ddd, *J* = 18.2, 15.0, 1.6 Hz, 1H), 2.84 – 2.60 (m, 1H), 2.59 – 2.32 (m, 1H). ^{13}C NMR (101 MHz, Chloroform-*d*) δ 133.2 (d, *J* = 97.8 Hz), 133.0, 132.93 (d, *J* = 5.4 Hz), 132.5 (d, *J* = 2.7 Hz), 132.0 (d, *J* = 2.8 Hz), 132 (d, *J* = 2.9 Hz), 131.7, 131.6, 131.5 (d, *J* = 8.3 Hz), 131.4, 131.3, 131.1, 131.0, 130.6 (d, *J* = 49.7 Hz), 130.2 (d, *J* = 8.9 Hz), 129.4, 129.3, 128.7, 128.6 (d, *J* = 3.5 Hz), 128.5, 128.4 (d, *J* = 2.7 Hz), 127.0, 40.6 (dd, *J* = 67.3, 4.4 Hz), 35.4 (d, *J* = 61.7 Hz), 27.5, 27.1 (d, *J* = 68.6 Hz). HRMS (ESI, M + H+): m/z calcd for $C_{27}H_{25}O_2P_2$: 443.1324, found: 443.1333.

ISOP-Ha'

$^{31}P\{1H\}$ NMR (162 MHz, Chloroform-*d*) δ 35.37 (d, *J* = 10.9 Hz), 33.72 (d, *J* = 10.9 Hz). ^{1}H NMR (400 MHz, Chloroform-*d*) δ 8.04 (ddd, *J* = 10.9, 7.6, 1.9 Hz, 2H), 7.71 – 7.55 (m, 5H), 7.47 (dd, *J* = 7.5, 1.6 Hz, 1H), 7.41 – 7.33 (m, 3H), 7.33 – 7.13 (m, 4H), 7.13 (dd, *J* = 7.4, 1.6 Hz, 1H), 7.05 (d, *J* = 7.5 Hz, 1H), 6.95 (t, *J* = 7.5 Hz, 1H), 6.70 (t, *J* = 1.7 Hz, 1H), 4.55 (dd, *J* = 22.6, 15.1 Hz, 1H), 4.10 (dtd, *J* = 25.5, 9.7, 3.1 Hz, 1H), 3.19 (t, *J* = 15.0 Hz, 1H), 2.94 – 2.55 (m, 2H). ^{13}C NMR (101 MHz, Chloroform-*d*) δ 135.1, 134.1, 134.1, 134.0, 132.4 (d, *J* = 2.8 Hz), 132.1, 132.0 (d, *J* = 2.7 Hz), 131.8 (d, *J* = 8.4 Hz), 131.5 (d, *J* = 8.8 Hz), 130.8 (dd, *J* = 5.2, 2.6 Hz), 130.8 (d, *J* = 58.3 Hz), 130.1 (d, *J* = 8.7 Hz), 129.2 (d, *J* = 11.3 Hz), 128.4 (dd, *J* = 11.7, 9.8 Hz), 127.0, 43.1 (dd, *J* = 65.7, 4.6 Hz), 35.2 (d, *J* = 61.8 Hz), 26.6 (d, *J* = 69.2 Hz). HRMS (ESI, M + H+): m/z calcd for $C_{27}H_{25}O_2P_2$: 443.1324, found: 443.1320.

4-(di-o-tolyphosphoryl)-2-phenyl-1,3,4-trihydroisophosphinoline 2-oxide ISOP-oMea,a'

Products ISOP-$oMe_{a,a'}$were prepared following the general protocol, using isophosphinoline (180 mg, 0.825mmol, 1 eq), di-o-tolyphosphine oxide 1b (111 mg, 0,55 mmol, 1,1 eq) and potassium tert-butoxide (11 mg, 0,1 mmol, 20 mol%) in 2.5 mL of acetonitrile. Products ISOP-$oMe_{a,}$, ISOP-$oMe_{a'}$ were isolated as a white powder with a yield of 86% (304 mg, ISOP-oMe_a 132 mg, ISOP-$oMe_{a'}$ 172 mg).

ISOP-oMea

$^{31}P\{H\}$ NMR (162 MHz, Chloroform-*d*) δ 44.20 (d, *J* = 15.7 Hz), 34.63 (d, *J* = 15.7 Hz). ^{1}H NMR (400 MHz, Chloroform-*d*) δ 8.01 (ddd, *J* = 11.8, 8.2, 1.5 Hz, 2H), 7.82 – 7.73 (m, 1H), 7.63 – 7.48 (m, 4H), 7.46 – 7.33 (m, 3H), 7.33 – 7.22 (m, 3H), 7.14 (ddd, *J* = 15.5, 7.7, 3.3 Hz, 2H), 7.02 – 6.90 (m, 1H), 6.75 (d, *J* = 7.7 Hz, 2H), 4.49 – 4.26 (m, 1H), 3.29 (ddd, *J* = 18.3, 15.0, 1.9 Hz, 1H), 3.10 (tdd, *J* = 15.6, 7.4, 4.3 Hz, 1H), 2.86 – 2.65 (m, 1H), 2.50 (s, 3H), 2.16 (s, 3H). ^{13}C NMR (101 MHz, Chloroform-*d*) δ 143.8 (d, *J* = 7.4 Hz), 143.5 (d, *J* = 7.2 Hz), 133.2, 133.1 (d, *J* = 2.8 Hz), 133.0 (d, *J* = 98.4 Hz), 133.0 (d, *J* = 10.0 Hz), 132.2 (d, *J* = 2.7 Hz), 131.8 (d, *J* = 2.7 Hz), 131.5 (d, *J* = 5.4 Hz), 131.3 (d, *J* = 9.6 Hz), 130.4 (d, *J* = 40.4 Hz), 129.5 (d, *J* = 43.4 Hz), 128.6 (d, *J* = 12.0 Hz), 128.3 (d, *J* = 2.6 Hz), 127.0 125.7 (d, *J* = 11.3 Hz), 125.4 (d, *J* = 12.7 Hz), 39.7 (d, *J* = 5.1 Hz), 39.0 (d, *J* = 5.1 Hz), 35.2 (d, *J* = 62.1 Hz), 27.8 (dd, *J* = 69.4, 2.7 Hz), 21.6 (d, *J* = 4.2 Hz), 20.6 (d, *J* = 3.2 Hz). (ESI, M + H+): m/z calcd for $C_{29}H_{29}O_2P_2$: 471.1637, found: 471.1630.

ISOP-oMea'

$^{31}P\{H\}$ NMR (162 MHz, Chloroform-*d*) δ 42.80 (d, *J* = 11.7 Hz), 33.48 (d, *J* = 11.7 Hz). ^{1}H NMR (400 MHz, Chloroform-*d*) δ 7.67 (ddd, *J* = 11.2, 7.7, 1.4 Hz, 1H), 7.55 – 7.44 (m, 1H), 7.38 – 7.16 (m, 9H), 7.13 – 6.91 (m, 4H), 6.86 (t, *J* = 7.6 Hz, 1H), 6.59 – 6.39 (m, 1H), 4.69 – 4.45 (m, 1H), 4.25 – 4.03 (m, 1H), 3.32 – 3.00 (m, 2H), 3.00 – 2.65 (m, 1H), 2.43 (s, 3H), 2.07 (s, 3H). ^{13}C NMR (101 MHz, Chloroform-*d*) δ 144.2 (d, *J* = 7.0 Hz), 143.9 (d, *J* = 7.5 Hz), 134.6 (d, *J* = 97.1 Hz), 134.3 (t, *J* = 5.6 Hz), 133.0 (d, *J* = 10.1 Hz), 132.3 (d, *J* = 5.3 Hz), 132.2 (d, *J* = 2.4 Hz), 132.0 (d, *J* = 12.5 Hz), 131.8, 131.7 (d, *J* = 7.7 Hz), 131.5 (d, *J* = 2.7 Hz), 131.4 (dd, *J* = 8.7, 2.4 Hz), 131.0 (d, *J* = 10.0 Hz), 130.8 (dd, *J* = 5.0, 2.6 Hz), 130.6, 130.20 (d, *J* = 8.6 Hz), 129.5 (d, *J* = 46.0 Hz), 128.4, 128.3 (d, *J* = 7.5 Hz), 127.0 (d, *J* = 2.3 Hz), 125.5 (d, *J* = 3.6 Hz), 125.4 (d, *J* = 4.8 Hz), 42.0 (d, *J* = 5.0 Hz), 41.4 (d, *J* = 5.0 Hz), 35.0 (d, *J* = 62.3 Hz), 26.7 (d, *J* = 69.1 Hz), 21.6 (d, *J* = 4.2 Hz), 20.7 (d, *J* = 3.1 Hz). HRMS (ESI, M + H^+): m/z calcd for $C_{29}H_{29}O_2P_2$ 471.1637, found: 471.1636.

4-(di-p-tolyphosphoryl)-2-phenyl-1,3,4-trihydroisophosphinoline 2-oxide ISOP-pMea,a'

Products ISOP-$pMe_{a,a'}$ were prepared following the general protocol, using isophosphinoline (180 mg, 0.825mmol, 1 eq), di-p-tolylphosphine oxide 1d (190 mg, 0,825 mmol, 1,1 eq) and potassium tert-butoxide (17 mg, 0,15 mmol, 20 mol%) in 2.5 mL of acetonitrile. Products ISOP-pMe_a and mixture ISOP-$pMe_{a,}$ + ISOP-$pMe_{a'}$ were isolated as a white powder with a yield of 80% (284 mg, ISOP-pMe_a 236 mg, mixture ISOP-$pMe_{a,}$ + ISOP-$pMe_{a'}$ 48 mg).

ISOP-pMea

$^{31}P\{H\}$ NMR (162 MHz, Chloroform-*d*) δ 37.10 (d, *J* = 14.9 Hz), 36.10 (d, *J* = 14.9 Hz). ^{1}H NMR (400 MHz, Chloroform-*d*) δ 8.07 – 7.94 (m, 2H), 7.81 (dd, *J* = 10.4, 7.9 Hz, 2H), 7.60 – 7.28 (m, 7H), 7.21 – 7.07 (m, 4H), 6.94 (s, 1H), 6.81 (d, *J* = 7.7 Hz, 1H), 4.32 (dd, *J* = 15.0, 8.8 Hz, 1H), 4.07 (dtd, *J* = 21.8, 8.7, 3.8 Hz, 1H), 3.18 (dd, *J* = 18.1, 15.3 Hz, 1H), 2.65 (dddd, *J* = 15.3, 10.7, 6.7, 3.3 Hz, 1H), 2.54 – 2.42 (m, 1H), 2.40 (s, 3H), 2.32 (s, 3H). ^{13}C NMR (101 MHz, Chloroform-*d*) δ 142.7 (dd, *J* = 50.2, 2.8 Hz), 133.4 (d, *J* = 76.2 Hz), 133.0 (d, *J* = 2.8 Hz), 132.9 (d, *J* = 7.3 Hz), 131.9 (d, *J* = 2.9 Hz), 131.7 (d, *J* = 5.4 Hz), 131.6, 131.4 (d, *J* = 5.9 Hz), 131.3 (d, *J* = 7.0 Hz), 131.1 (d, *J* = 9.3 Hz), 130.0 (d, *J* = 11.5 Hz), 129.2 (d, *J* = 12.1 Hz), 128.6 (d, *J* = 12.0 Hz), 128.4 (d, *J* = 60.4 Hz), 128.3 (d, *J* = 2.6 Hz), 127.5 (d, *J* = 56.0 Hz), 127.0, 40.7 (dd, *J* = 67.5, 4.5 Hz), 35.4 (d, *J* = 61.7 Hz), 27.5 (d, *J* = 69.7 Hz), 21.7 (d, *J* = 2.6 Hz). HRMS (ESI, M + H^+): m/z calcd for $C_{29}H_{29}O_2P_2$ 471.1637, found: 471.1645.

ISOP-pMea, + ISOP-pMea'

^{31}P{H} NMR (162 MHz, Chloroform-*d*) δ 37.21 (d, *J* = 14.9 Hz), 36.41 (d, *J* = 14.9 Hz), 36.00 (d, *J* = 8.9 Hz), 34.29 (d, *J* = 8.9 Hz). HRMS (ESI, M + H$^+$): m/z calcd for $C_{29}H_{29}O_2P_2$ 471.1637, found: 471.1633.

(Bis(2-methoxyphenyl) phosphoryl)-2-phenyl-1,3,4-trihydroisophosphinoline 2-oxide ISOP-mOMea,a'

Products ISOP-mOMe$_{a,a'}$ were prepared following the general protocol, using isophosphinoline (180 mg, 0.75mmol, 1 eq), di-o-methoxyphosphine oxide 1e (216 mg, 0,825 mmol, 1,1 eq) and potassium tert-butoxide (16.8 mg, 0,15 mmol, 20 mol%) in 2.5 mL of acetonitrile. Products ISOP-mOMe$_a$ and ISOP-mOMe$_{a'}$ were isolated as a white powder with a yield of 85% (323 mg, ISOP-mOMe$_a$ 184 mg, ISOP-mOMe$_{a'}$ 139 mg).

ISOP-mOMea

^{31}P{H} NMR (162 MHz, Chloroform-*d*) δ 37.30 (d, *J* = 17.8 Hz), 36.19 (d, *J* = 17.8 Hz). ^{1}H NMR (400 MHz, Chloroform-*d*) δ 7.98 (ddd, *J* = 11.7, 8.2, 1.5 Hz, 2H), 7.86 (ddd, *J* = 12.8, 7.6, 1.8 Hz, 1H), 7.60 (ddd, *J* = 13.0, 7.6, 1.8 Hz, 1H), 7.53 – 7.33 (m, 5H), 7.17 – 7.03 (m, 4H), 6.99 – 6.85 (m, 3H), 6.74 (dd, *J* = 8.3, 5.4 Hz, 1H), 4.80 (dddd, *J* = 20.9, 12.9, 8.9, 4.4 Hz, 1H), 4.31 – 4.17 (m, 1H), 3.82 (s, 3H), 3.53 (s, 3H), 3.15 (ddd, *J* = 18.3, 14.9, 1.7 Hz, 1H), 2.86 (tdd, *J* = 16.2, 6.7, 4.4 Hz, 1H), 2.57 (td, *J* = 17.6, 9.0 Hz, 1H). ^{13}C NMR (101 MHz, Chloroform-*d*) δ 160.0 (d, *J* = 3.9 Hz), 159.9 (d, *J* = 3.8 Hz), 134.7 (d, *J* = 6.0 Hz), 134.0, 133.9 (d, *J* = 6.0 Hz), 133.7 (d, *J* = 2.2 Hz), 133.6 (d, *J* = 2.0 Hz), 133.5 (d, *J* = 5.5 Hz), 133.1, 132.6 (d, *J* = 6.6 Hz), 132.6 (d, *J* = 6.5 Hz), 131.8 (d, *J* = 2.9 Hz), 131.5, 131.4, 131.1, 131.0, 130.3 (d, *J* = 2.5 Hz), 130.2 (d, *J* = 2.5 Hz), 128.5 (d, *J* = 12.0 Hz), 128.0 (d, *J* = 2.9 Hz), 126.8, 121.4 (d, *J* = 17.7 Hz), 121.2 (d, *J* = 10.8 Hz), 120.8 (d, *J* = 11.3 Hz), 120.4 (d, *J* = 22.9 Hz), 111.3 (d, *J* = 6.8 Hz), 110.8 (d, *J* = 6.8 Hz), 55.5 (d, *J* = 40.0 Hz), 39.6 (dd, *J* = 70.3, 4.9 Hz), 35.6 (d, *J* = 61.7 Hz), 27.6 (d, *J* = 70.2 Hz). HRMS (ESI, M + H$^+$): m/z calcd for $C_{29}H_{29}O_4P_2$ 503.1536, found: 503.1536.

ISOP-mOMea'

^{31}P{H} NMR (162 MHz, Chloroform-*d*) δ 37.87 (d, *J* = 15.7 Hz), 34.47 (d, *J* = 15.8 Hz). ^{1}H NMR (400 MHz, Chloroform-*d*) δ 7.83 (ddd, *J* = 13.1, 7.7, 1.8 Hz, 1H), 7.52 (ddd, *J* = 13.2, 7.6, 1.8 Hz, 1H), 7.45 (d, *J* = 1.8 Hz, 1H), 7.34 (dt, *J* = 5.8, 1.9 Hz, 2H), 7.25 (ddt, *J* = 10.9, 7.3, 4.9 Hz, 4H), 7.08 – 6.96 (m, 3H), 6.96 – 6.82 (m, 4H), 6.76 (dd, *J* = 8.4, 5.4 Hz, 1H), 4.80 – 4.57 (m, 1H), 4.35 (dd, *J* = 22.0, 15.1 Hz, 1H), 3.84 (s, 3H), 3.59 (s, 3H), 3.10 (t, *J* = 15.0 Hz, 1H), 2.91 (dtd, *J* = 19.9, 16.6, 3.8 Hz, 1H), 2.78 – 2.52 (m, 1H). ^{13}C NMR (101 MHz, Chloroform-*d*) δ 160.0 (d, *J* = 3.7 Hz), 159.6 (d, *J* = 3.9 Hz), 135.3 (d, *J* = 6.0 Hz), 134.2 (d, *J* = 183.8 Hz), 134.1 (d, *J* = 6.5 Hz), 133.9 (d, *J* = 6.0 Hz), 133.7 (dd, *J* = 7.8, 2.2 Hz), 133.3 (d, *J* = 11.7 Hz), 131.4 (d, *J* = 2.8 Hz), 131.0 (dd, *J* = 8.8, 2.5 Hz), 130.2 (d, *J* = 8.5 Hz), 128.2 (d, *J* = 11.3 Hz), 127.8 (d, *J* = 3.0 Hz), 126.7, 121.3 (d, *J* = 10.9 Hz), 120.8 (d, *J* = 11.4 Hz), 120.3 (d, *J* = 95.4 Hz), 110.9 (d, *J* = 6.9 Hz), 110.7 (d, *J* = 6.8 Hz), 55.4 (d, *J* = 26.8 Hz), 42.4 (d, *J* = 4.9 Hz), 41.7 (d, *J* = 5.0 Hz), 35.2, 34.5, 26.5, 25.8. HRMS (ESI, M + H$^+$): m/z calcd for $C_{29}H_{29}O_4P_2$ 503.1536, found: 503.1539.

(Bis (3-methoxyphenyl) phosphoryl)-2-phenyl-1,3,4-trihydroisophosphinoline 2-oxide ISOP-mOMea,a'

Products ISOP-mOMe$_a$ and ISOP-mOMe$_{a'}$ were prepared following the general protocol, using isophosphinoline (180 mg, 0.75mmol, 1 eq), di-m-methoxyphosphine oxide 1f (216 mg, 0,825 mmol, 1,1 eq) and potassium tert-butoxide (17 mg, 0,15 mmol, 20 mol%) in 2.5 mL of

acetonitrile. Products ISOP-mOMe$_a$ and ISOP-mOMe$_{a'}$ were isolated as a white powder with a yield of 88% (333 mg, ISOP-mOMe$_a$ 202 mg, ISOP-mOMe$_{a'}$ 131 mg).

ISOP-mOMea

^{31}P{1H} (162 MHz, Chloroform-*d*) δ 37.20 (d, *J* = 13.7 Hz), 35.83 (d, *J* = 13.8 Hz). ^{1}H NMR (400 MHz, Chloroform-*d*) δ 8.00 (ddd, *J* = 11.9, 8.2, 1.5 Hz, 2H), 7.62 – 7.41 (m, 5H), 7.26 – 7.14 (m, 3H), 7.12 – 6.85 (m, 4H), 6.80 – 6.69 (m, 3H), 4.35 (dd, *J* = 15.1, 8.7 Hz, 1H), 4.09 (ddd, *J* = 16.1, 8.8, 4.5 Hz, 1H), 3.83 (s, 3H), 3.64 (s, 3H), 3.17 (ddd, *J* = 18.4, 15.1, 1.6 Hz, 1H), 2.80 – 2.61 (m, 1H), 2.58 – 2.35 (m, 1H). ^{13}C NMR (101 MHz, Chloroform-*d*) δ 160.2 (d, *J* = 13.9 Hz), 159.5 (d, *J* = 14.3 Hz), 133.4 (d, *J* = 74.4 Hz), 133.0 (d, *J* = 3.2 Hz), 132.6 (d, *J* = 44.5 Hz), 131.9 (d, *J* = 3.0 Hz), 131.6 (d, *J* = 2.1 Hz), 131.5 (d, *J* = 2.1 Hz), 131.4, 131.3 (d, *J* = 9.7 Hz), 131.1, 131.0 (d, *J* = 2.4 Hz), 130.5 (d, *J* = 13.4 Hz), 129.7 (d, *J* = 14.2 Hz), 128.6 (d, *J* = 12.0 Hz), 128.4 (d, *J* = 2.7 Hz), 127.0, 123.1 (d, *J* = 8.6 Hz), 122.9 (d, *J* = 9.5 Hz), 118.4 (dd, *J* = 17.9, 2.8 Hz), 116.9 (d, *J* = 9.0 Hz), 115.9 (d, *J* = 9.2 Hz), 55.5 (d, *J* = 18.4 Hz), 41.1 (d, *J* = 4.6 Hz), 40.4 (d, *J* = 4.6 Hz), 35.4 (d, *J* = 61.7 Hz), 27.5 (d, *J* = 69.3 Hz). HRMS (ESI, M + H$^+$): m/z calcd for $C_{29}H_{29}O_4P_2$ 503.1536, found: 503.1539.

ISOP-mOMea'

^{31}P{1H} NMR (162 MHz, Chloroform-*d*) δ 36.00 (d, *J* = 10.6 Hz), 33.93 (d, *J* = 10.6 Hz). ^{1}H NMR (400 MHz, Chloroform-*d*) δ 7.63 (d, *J* = 12.1 Hz, 1H), 7.51 (d, *J* = 7.1 Hz, 2H), 7.41 – 7.37 (m, 1H), 7.27 (dt, *J* = 8.1, 5.2 Hz, 4H), 7.21 – 6.94 (m, 7H), 6.70 (d, *J* = 7.7 Hz, 1H), 4.60 (dd, *J* = 22.7, 15.1 Hz, 1H), 4.06 (dtd, *J* = 25.9, 9.7, 2.9 Hz, 1H), 3.91 (s, 3H), 3.68 (s, 3H), 3.21 (t, *J* = 15.0 Hz, 1H), 2.89 – 2.48 (m, 3H). ^{13}C{H} NMR (101 MHz, Chloroform-*d*) δ 160.2 (d, *J* = 14.0 Hz), 159.5 (d, *J* = 14.4 Hz), 135.1, 134.1 (d, *J* = 10.6 Hz), 132.7, 132.2, 132.2 (d, *J* = 6.1 Hz), 132.0 (d, *J* = 6.0 Hz), 131.5 (d, *J* = 2.7 Hz), 131.5, 131.4 (d, *J* = 2.5 Hz), 130.9 (dd, *J* = 5.2, 2.6 Hz), 130.3, 130.1 (d, *J* = 9.1 Hz), 129.6 (d, *J* = 14.1 Hz), 128.3 (d, *J* = 11.7 Hz), 127.0, 123.3 (d, *J* = 3.0 Hz), 123.2 (d, *J* = 3.5 Hz), 118.6 (dd, *J* = 24.3, 2.7 Hz), 117.0 (d, *J* = 9.0 Hz), 116.3 (d, *J* = 9.2 Hz), 55.6 (d, *J* = 26.8 Hz), 43.1 (dd, *J* = 65.8, 4.7 Hz), 35.2 (d, *J* = 61.7 Hz), 26.6 (d, *J* = 69.2 Hz). HRMS (ESI, M + H$^+$): m/z calcd for $C_{29}H_{29}O_4P_2$ 503.1536, found: 503.1527.

4-(bis (3-fluorophenyl) phosphoryl)-2-phenyl-1,3,4-trihydroisophosphinoline-2-oxide ISOP-mFa,a'

Products ISOP-mF$_{a,a'}$ were prepared following the general protocol, using isophosphinoline (180 mg, 0.75mmol, 1 eq), di-m-fluorophosphine oxide 1h (196 mg, 0,825 mmol, 1,1 eq) and potassium tert-butoxide (17 mg, 0,15 mmol, 20 mol%) in 2.5 mL of acetonitrile. Products ISOP-mF$_{a,a'}$ were isolated as a white powder with a yield of 80% (287 mg, mixture ISOP-mF$_a$ + ISOP-mF$_{a'}$ 76 mg, ISOP-mF$_{a'}$ 212 mg).

Mixture ISOP-mFa + ISOP-mFa'

^{31}P{H} NMR (162 MHz, $CDCl_3$) δ 35.61, 35.32 (d, *J* = 15.0 Hz), 34.85 (d, *J* = 14.8 Hz), 33.81-33.28 (m). HRMS (ESI, M + H$^+$): m/z calcd for $C_{27}H_{23}F_2O_2P_2$ 479.1136, found: 479.1133.

ISOP-mFa'

^{31}P{H} NMR (162 MHz, Chloroform-*d*) δ (d, *J* = 41 Hz). ^{1}H NMR (400 MHz, Chloroform-*d*) δ 7.88 – 7.77 (m, 1H), 7.75 – 7.65 (m, 1H), 7.60 (tdd, *J* = 8.4, 5.3, 3.6 Hz, 1H), 7.39 – 7.30 (m, 2H), 7.30 – 7.20 (m, 2H), 7.20 – 7.12 (m, 1H), 7.06 (d, *J* = 7.5 Hz, 1H), 6.96 (t, *J* = 7.5 Hz, 1H), 6.66 (d, *J* = 7.6 Hz, 1H), 4.57 (dd, *J* = 22.0, 15.2 Hz, 1H), 4.20 – 3.96 (m, 1H), 3.21 (t, *J* = 14.9 Hz, 1H), 2.65 (td, *J* = 16.3, 15.3, 4.5 Hz, 1H). ^{13}C NMR (101 MHz, Chloroform-*d*) δ 164.3 (d, *J* = 15.9 Hz), 163.8 (d, *J* = 15.9 Hz), 161.8 (d, *J* = 15.7 Hz), 161.3 (d, *J* = 16.3 Hz),

134.2, 132.8, 132.3, 131.8, 131.7, 131.5 (d, J = 7.3 Hz), 131.4 (d, J = 7.3 Hz), 130.7, 130.6, 130.5 (d, J = 7.7 Hz), 130.1 (d, J = 8.6 Hz), 128.9 (d, J = 2.9 Hz), 128.5 (d, J = 11.4 Hz), 127.6 (s), 127.5 (s), 127.5 (s), 127.4 (s), 127.2, 127.1, 127.0, 120.0 (d, J = 21.2 Hz), 119.5 (d, J = 21.2 Hz), 118.8 (d, J = 9.3 Hz), 118.6 (d, J = 9.7 Hz), 118.5 (d, J = 9.7 Hz), 118.3 (d, J = 9.3 Hz), 43.1 (d, J = 63.2 Hz), 35.3 (d, J = 60.2 Hz), 26.8 (d, J = 66.8 Hz). HRMS: m/z calcd for $C_{27}H_{23}F_2O_2P_2$ 479.1136 $[M + H]^+$, found 479.1133

4-(bis (4-fluorophenyl) phosphoryl)-2-phenyl-1,3,4-trihydroisophosphinoline-2-oxide. ISOP-pFa,a'

Products ISOP-pFa,a'were prepared following the general protocol, using isophosphinoline (180 mg, 0.75mmol, 1 eq), di-4-fluorophosphine oxide 1i (196 mg, 0,825 mmol, 1,1 eq) and potassium tert-butoxide (17 mg, 0,15 mmol, 20 mol%) in 2.5 mL of acetonitrile. Products ISOP-pF$_a$, ISOP-pF$_{a'}$ were isolated as a white powder with a yield of 84% (304 mg, ISOP-pF$_a$ 271 mg, mixture ISOP-pF$_a$ + ISOP-pF$_{a'}$ 33 mg).

ISOP-pFa + ISOP-pFa'

^{1}P{H} NMR (162 MHz, Chloroform-*d*) δ 35.89 (d, J = 36.3 Hz), 35.61 (d, J = 36.3 Hz), 34.68 (d, J = 10.3 Hz), 33.98 (d, J = 10.3 Hz). HRMS: m/z calcd for $C_{27}H_{23}F_2O_2P_2$ 479.1136 $[M + H]^+$, found 479.1134

ISOP-pFa

^{31}P{H} NMR (162 MHz, Chloroform-*d*) δ 35.92 (d, J = 14.4 Hz), 35.51 (d, J = 14.5 Hz). ^{1}H NMR (400 MHz, Chloroform-*d*) δ 8.10 – 7.82 (m, 3H), 7.61 – 7.37 (m, 5H), 7.40 – 7.15 (m, 5H), 7.14 – 6.93 (m, 2H), 6.99 – 6.92 (m, 1H), 6.74 (d, J = 7.3 Hz, 1H), 4.30 (dd, J = 15.1, 8.1 Hz, 1H), 4.16 – 3.96 (m, 1H), 3.35 – 3.08 (m, 1H), 2.72 – 2.56 (m, 1H), 2.46 (dd, J = 16.9, 9.4 Hz, 1H). ^{13}C{H} NMR (101 MHz, Chloroform-*d*) δ 166.8 (d, J = 2.8 Hz), 166.4 (d, J = 2.8 Hz), 164.2 (d, J = 3.1 Hz), 163.9 (d, J = 2.9 Hz), 134.0 (d, J = 9.3 Hz), 133.9, 133.7, 133.5, 133.5, 133.0 (d, J = 5.5 Hz), 133.0 (d, J = 5.3 Hz), 132.1 (d, J = 2.8 Hz), 131.8 (d, J = 7.1 Hz), 131.2, 131.0 (d, J = 4.3 Hz), 130.9 (d, J = 7.2 Hz), 130.8, 128.7 (d, J = 12.0 Hz), 127.2, 127.2 (d, J = 74.1 Hz), 126.2 (d, J = 68.4 Hz), 117.1 (d, J = 12.2 Hz), 116.9 (d, J = 12.2 Hz), 116.2 (d, J = 12.8 Hz), 116.0 (d, J = 12.9 Hz), 40.8 (d, J = 65.9 Hz), 35.3 (d, J = 60.9 Hz), 27.5 (d, J = 68.0 Hz). HRMS: m/z calcd for $C_{27}H_{23}F_2O_2P_2$ 479.1136 $[M + H]^+$, found 479.1139

4-(bis(4-chloroxyphenyl) phosphoryl)-2-phenyl-1,3,4-trihydroisophosphinoline 2-oxide ISOP-pCla,a'

Products ISOP-pCl$_{a,a'}$ were prepared following the general protocol, using isophosphinoline (180 mg, 0.75mmol, 1 eq), bis(4-chlorophenyl)phosphine oxide 1k (224 mg, 0,825 mmol, 1,1 eq) and potassium tert-butoxide (17 mg, 0,15 mmol, 20 mol%) in 2.5 mL of acetonitrile. Products ISOP-pCl$_a$ and ISOP-pCl$_{a'}$ were isolated as a white powder with a yield of 70% (268 mg, ISOP-pCl$_a$ 231 mg, mixture ISOP-pCl$_a$ + ISOP-pCl$_{a'}$ 37 mg).

ISOP-pCla

^{1}P{H} NMR (162 MHz, Chloroform-*d*) δ 35.87 (d, J = 15.1 Hz), 35.25 (d, J = 15.1 Hz). ^{1}H NMR (400 MHz, Chloroform-*d*) δ 8.05 – 7.90 (m, 2H), 7.86 (dd, J = 10.2, 8.4 Hz, 2H), 7.64 – 7.36 (m, 7H), 7.33 (d, J = 2.5 Hz, 2H), 7.22 (s, 2H), 6.98 (dt, J = 8.1, 4.2 Hz, 1H), 6.83 – 6.73 (m, 1H), 4.35 – 4.24 (m, 1H), 4.16 – 3.99 (m, 1H), 3.20 (ddd, J = 18.3, 15.1, 1.6 Hz, 1H), 2.60 (ddd, J = 15.6, 6.9, 4.0 Hz, 1H), 2.54 – 2.35 (m, 1H). ^{13}C {H} NMR (101 MHz, Chloroform-*d*) δ 146.1, 139.6, 139.0 (d, J = 3.4 Hz), 133.3 (d, J = 39.5 Hz), 132.8 (d, J = 9.1 Hz), 132.4 (d, J = 9.7 Hz), 132.1 (d, J = 2.8 Hz), 131.9 (d, J = 7.0 Hz), 131.2 (d, J = 9.7 Hz), 130.9 (d, J = 4.1

Hz), 130.8, 130.3 (d, J = 57.5 Hz), 129.9 (d, J = 11.7 Hz), 129.1 (d, J = 12.3 Hz), 128.9 (d, J = 95.5 Hz), 128.7 (d, J = 12.1 Hz), 127.3, 40.5 (dd, J = 68.2, 3.9 Hz), 35.3 (d, J = 61.6 Hz), 27.5 (d, J = 68.7 Hz). HRMS: m/z calculé pour $C_{27}H_{23}Cl_2O_2P_2$ 511.0545 $[M + H]^+$, trouvé 511.0540

Mixture ISOP-pCla + ISOP-pCla'

^{31}P NMR (162 MHz, Chloroform-*d*) δ 35.91 (d, J = 15.1 Hz), 35.44 (d, J = 15.2 Hz), 34.50 (d, J = 10.4 Hz), 33.47 (d, J = 10.6 Hz). HRMS: m/z calcd for $C_{27}H_{23}Cl_2O_2P_2$ 511.0545 $[M + H]^+$, found 511.0541

4-(bis(2,3-dichloroxyphenyl)phosphoryl)-2-phenyl-1,3,4-trihydroisophosphinoline 2-oxide ISOP-o,mCla,a'

Products ISOP-o,mCl$_{a,a'}$ were prepared following the general protocol, using isophosphinoline (180 mg, 0.75mmol, 1 eq), 4-(bis(2,3-dichlorophenyl)phosphine oxide 11 (280 mg, 0,825 mmol, 1,1 eq) and potassium tert-butoxide (17 mg, 0,15 mmol, 20 mol%) in 2.5 mL of acetonitrile. Products 3i,4i were isolated as a white powder with a yield of 79% (345 mg, ISOP-o,mCl$_a$ 25 mg, mixture ISOP-o,mCl$_a$ + ISOP-o,mCl$_{a'}$4i 128 mg), ISOP-o,mCl$_{a'}$ 192 mg.

ISOP-o,mCla

$^{1}P\{H\}$ NMR (162 MHz, Chloroform-*d*) δ 35.45 (m), 33.73 (d, J = 16.9 Hz). ^1H NMR (400 MHz, Chloroform-*d*) δ 8.03 (dd, J = 10.9, 1.8 Hz, 1H), 7.89 (dd, J = 11.8, 7.4 Hz, 2H), 7.76 – 7.68 (m, 1H), 7.65 (dd, J = 8.1, 3.1 Hz, 1H), 7.60 (d, J = 1.8 Hz, 1H), 7.57 (d, J = 1.7 Hz, 1H), 7.53 (dd, J = 7.4, 1.6 Hz, 1H), 7.46 (dd, J = 8.0, 3.0 Hz, 2H), 7.37 – 7.28 (m, 1H), 7.23 (d, J = 4.3 Hz, 2H), 7.02 (d, J = 4.4 Hz, 1H), 6.86 (d, J = 7.7 Hz, 1H), 4.21 (q, J = 8.6, 7.2 Hz, 2H), 3.27 (t, J = 16.9 Hz, 1H), 2.74 – 2.39 (m, 2H). ^{13}C {H} NMR (101 MHz, Chloroform-*d*) δ 146.2, 137.8 (d, J = 62.0 Hz), 134.8 (d, J = 15.1 Hz), 133.9 (d, J = 15.8 Hz), 133.5 (d, J = 9.6 Hz), 132.9 (d, J = 10.3 Hz), 132.4, 132.0 (d, J = 7.4 Hz), 131.8 (d, J = 12.4 Hz), 131.1 (d, J = 9.6 Hz), 131.0, 130.0, 129.8 (d, J = 9.1 Hz), 129.1, 128.82 (d, J = 12.2 Hz), 127.5, 40.1 (d, J = 64.3 Hz), 35.1 (d, J = 61.8 Hz), 27.5 (d, J = 68.1 Hz). HRMS: m/z calcd for $C_{27}H_{21}Cl_4O_2P_2$ 578.9765 $[M + H]^+$, found 578.9761

Mixture ISOP-o,mCla + ISOP-o,mCla'

^{31}P NMR (162 MHz, Chloroform-d) δ 35.46, 33.74 (d, J = 16.8 Hz), 33.09, 32.58.

ISOP-o,mCla'

$^{31}P\{H\}$ NMR (162 MHz, $CDCl_3$) δ 33.06, 32.64. ^1H NMR (400 MHz, Chloroform-*d*) δ 8.04 (d, J = 10.7 Hz, 1H), 7.85 (t, J = 8.9 Hz, 1H), 7.67 (d, J = 8.2 Hz, 1H), 7.55 (d, J = 11.1 Hz, 1H), 7.46 – 7.30 (m, 2H), 7.29 – 7.16 (m, 5H), 7.14 – 6.97 (m, 2H), 6.69 (d, J = 7.7 Hz, 1H), 4.54 (dd, J = 22.7, 15.3 Hz, 1H), 4.02 (s, 1H), 3.23 (t, J = 15.0 Hz, 1H), 2.64 (s, 2H). ^{13}C NMR (101 MHz, Chloroform-*d*) δ 138.1, 137.4, 134.6, 134.3 (d, J = 34.5 Hz), 133.7 (d, J = 15.5 Hz), 133.4 (d, J = 10.2 Hz), 133.2 (d, J = 10.0 Hz), 132.0, 131.8 (d, J = 12.9 Hz), 131.0, 130.8, 130.1 (d, J = 8.8 Hz), 129.2, 128.5 (d, J = 11.6 Hz), 127.4, 43.1 (d, J = 67.7 Hz), 35.3 (d, J = 61.6 Hz), 27.0 (d, J = 68.1 Hz). HRMS: m/z calcd for $C_{27}H_{21}Cl_4O_2P_2$ 578.9765 $[M + H]^+$, found 578.9761

S2 - ^{31}P{1H}, ^{1}H and ^{13}C{1H} NMR and HRMS spectra of the synthesized compounds

Diphenylphosphine oxide 1a

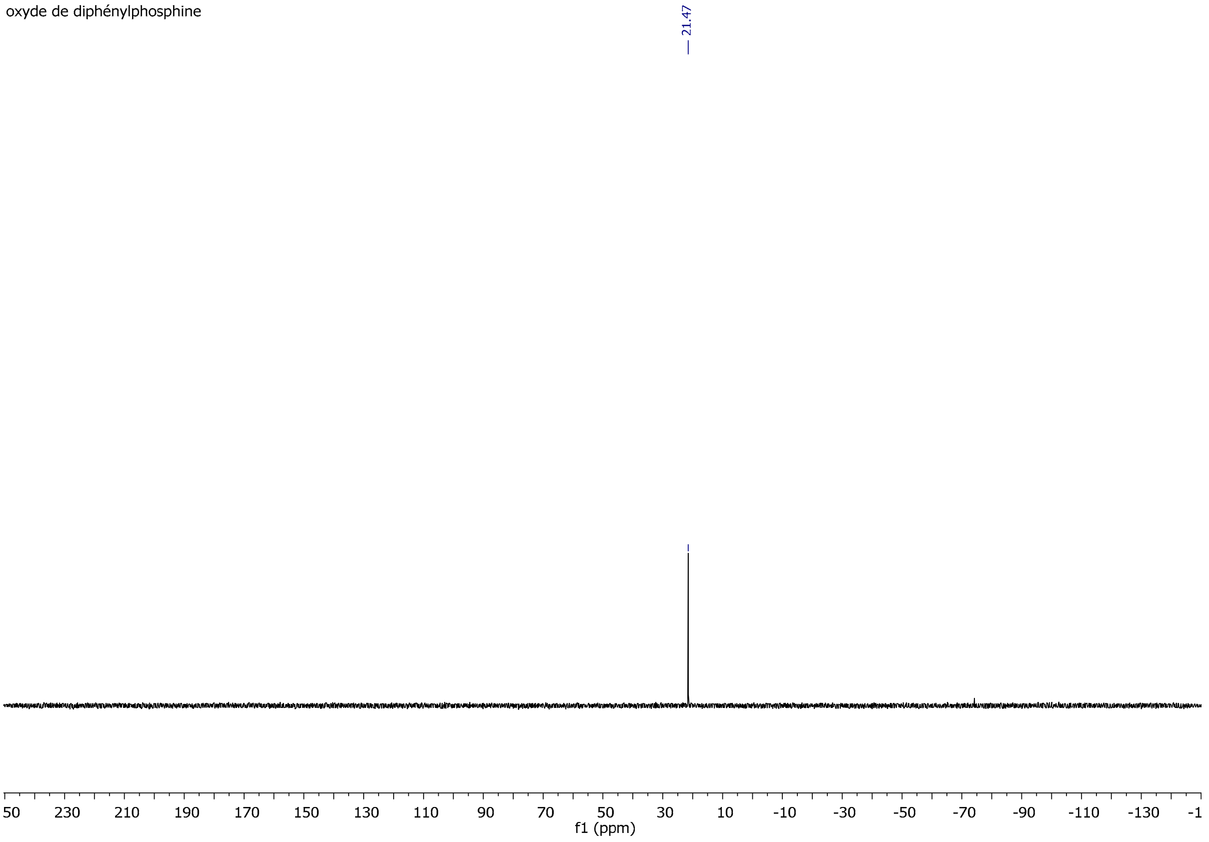

oxyde de diphénylphosphine

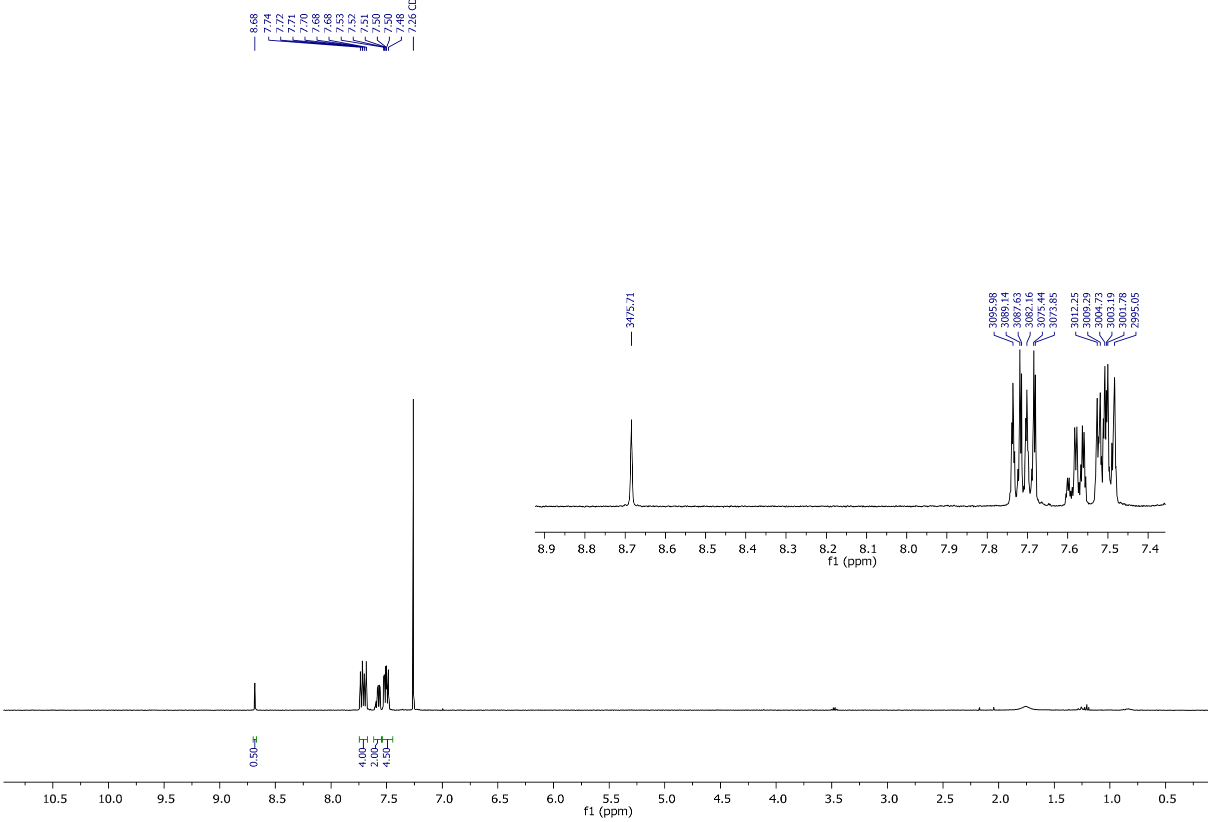

Di-o-tolylphosphine oxide 1b

oxyde de di-o-tolylphosphine

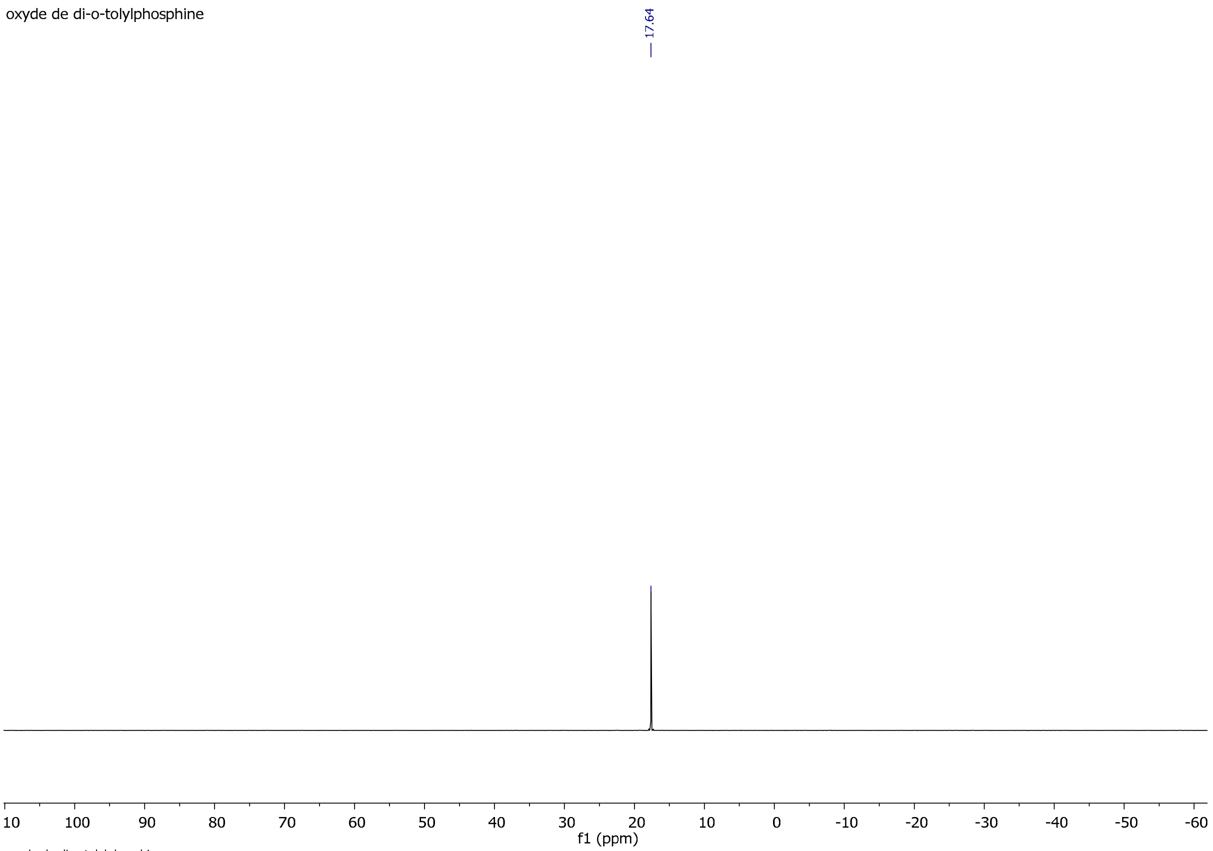

oxyde de di-o-tolylphosphine

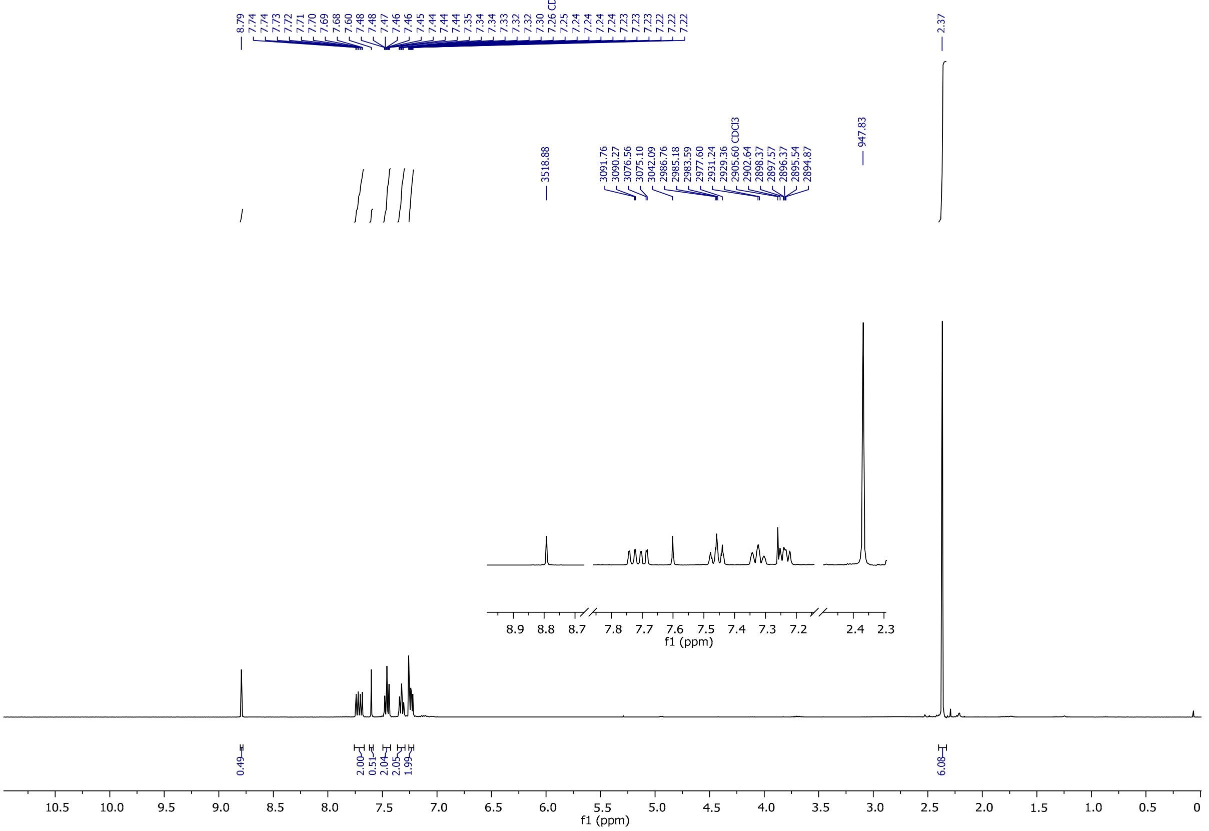

Di-m-tolylphosphine oxide 1c.

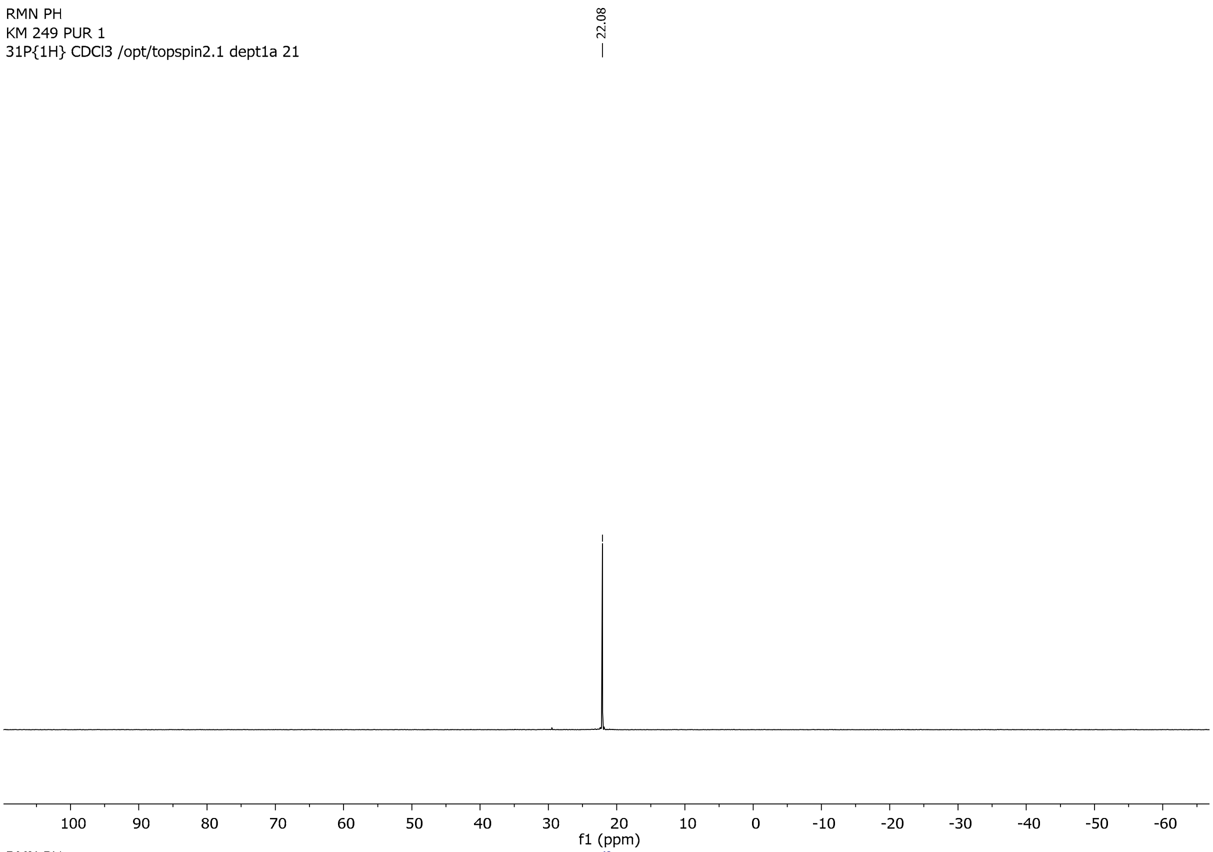

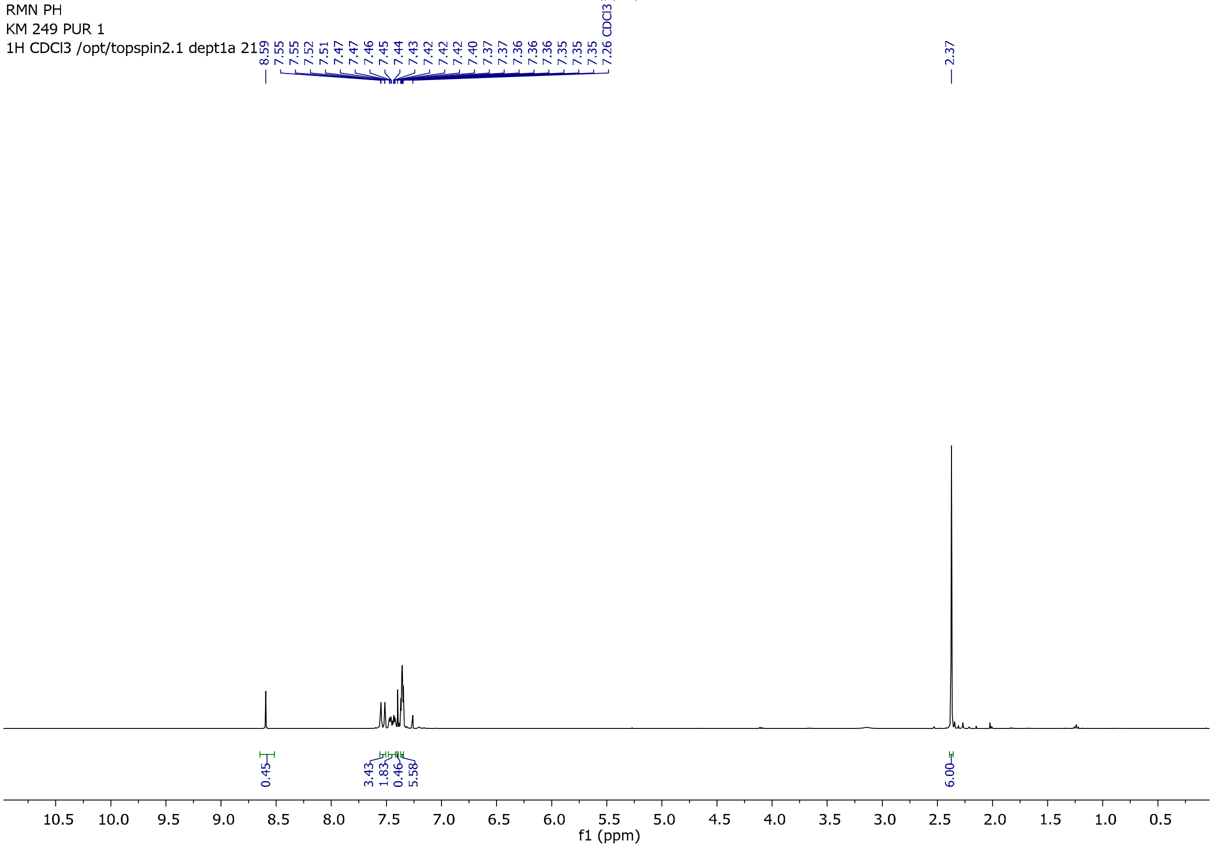

Di-p-tolylphosphine oxide 1d.

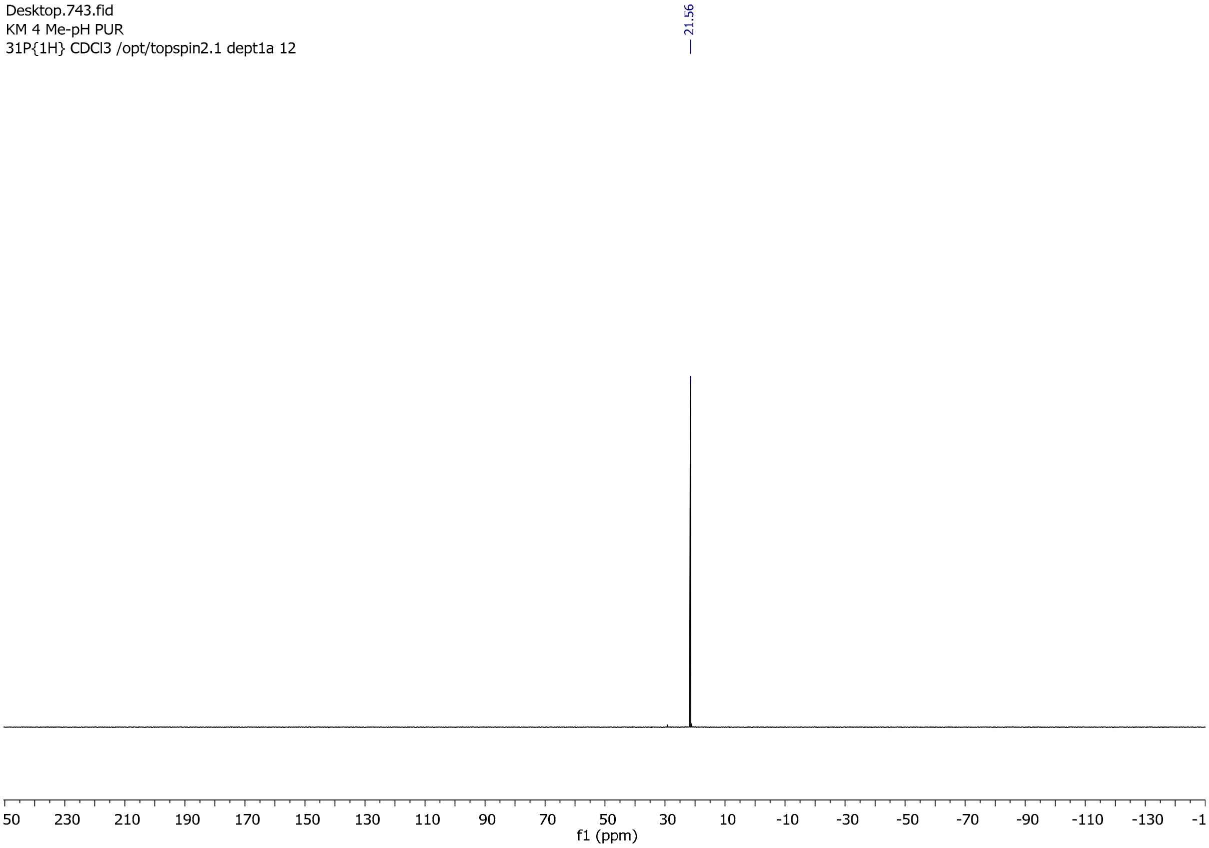

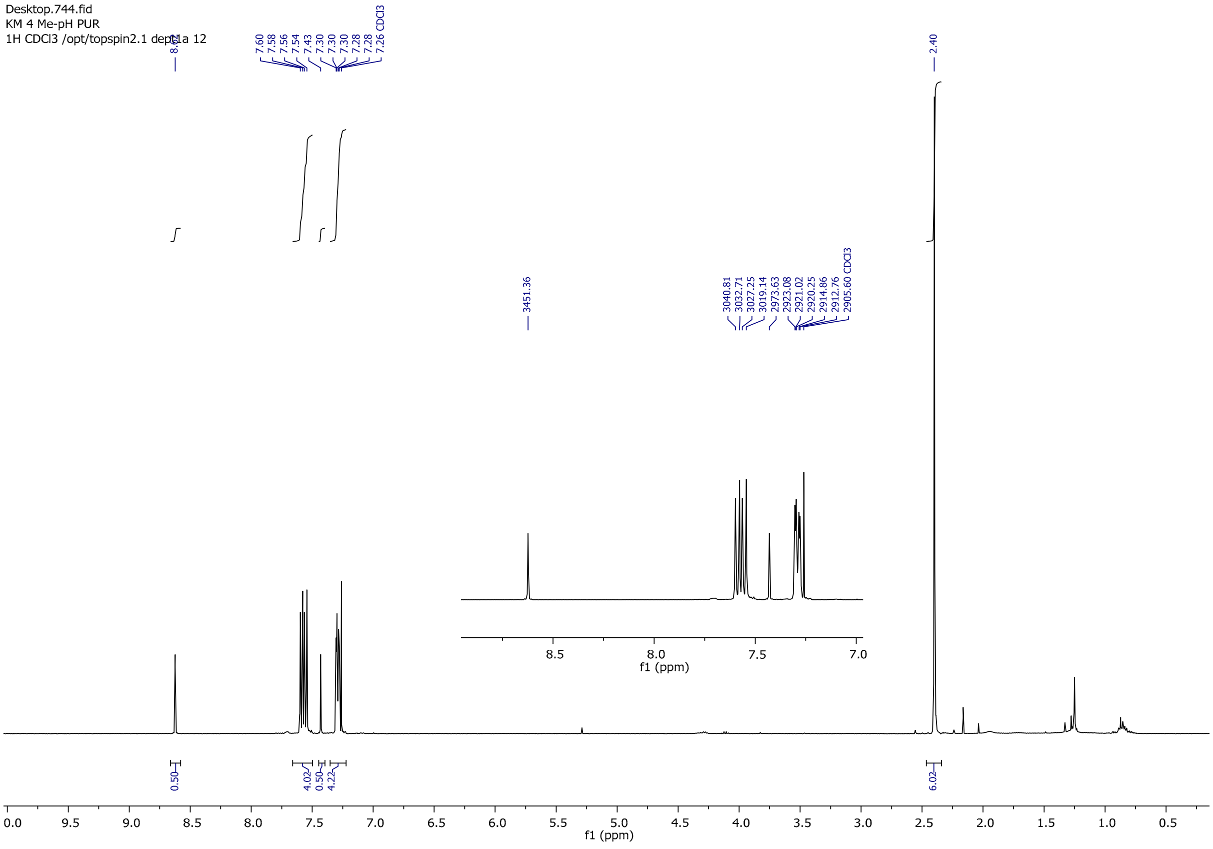

Bis(2-methoxyphenyl)phosphine oxide 1e

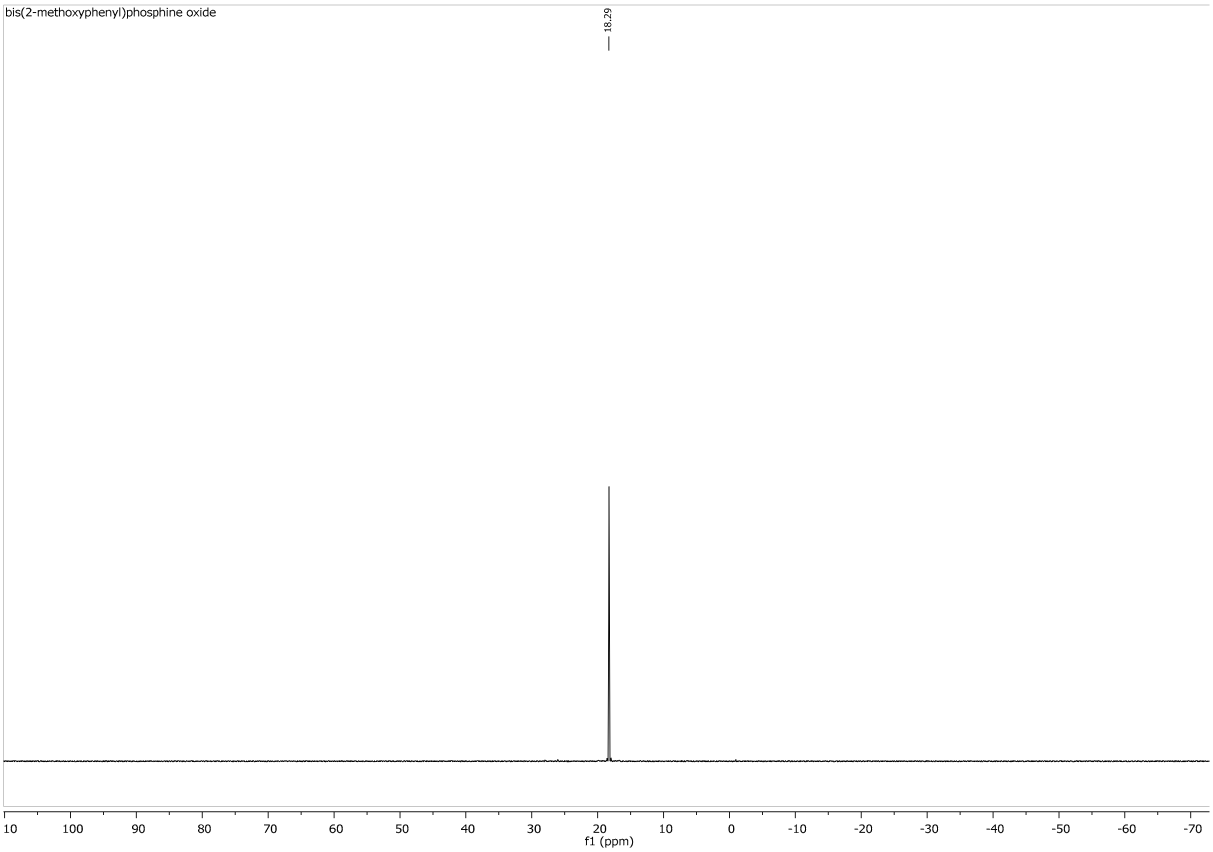

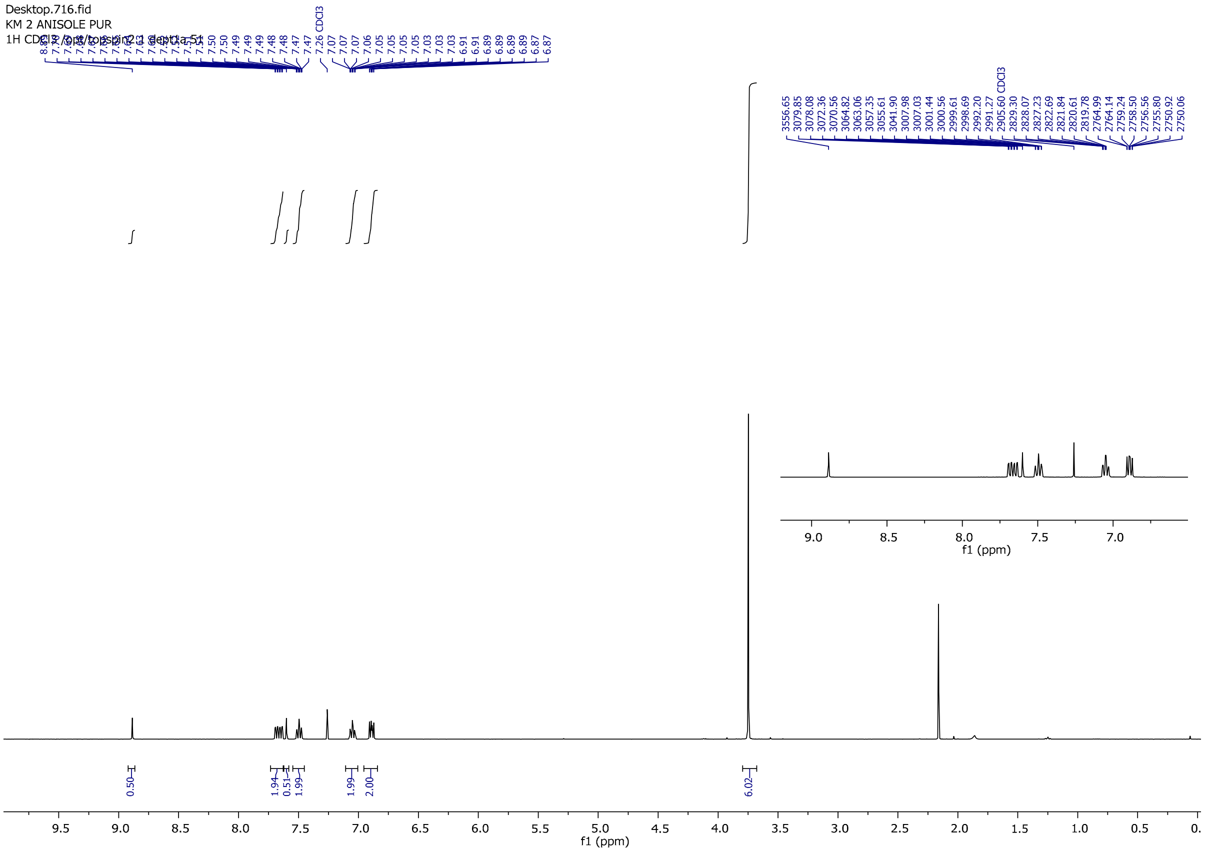

Bis(3-metoxyphenyl) phosphine oxide 1f

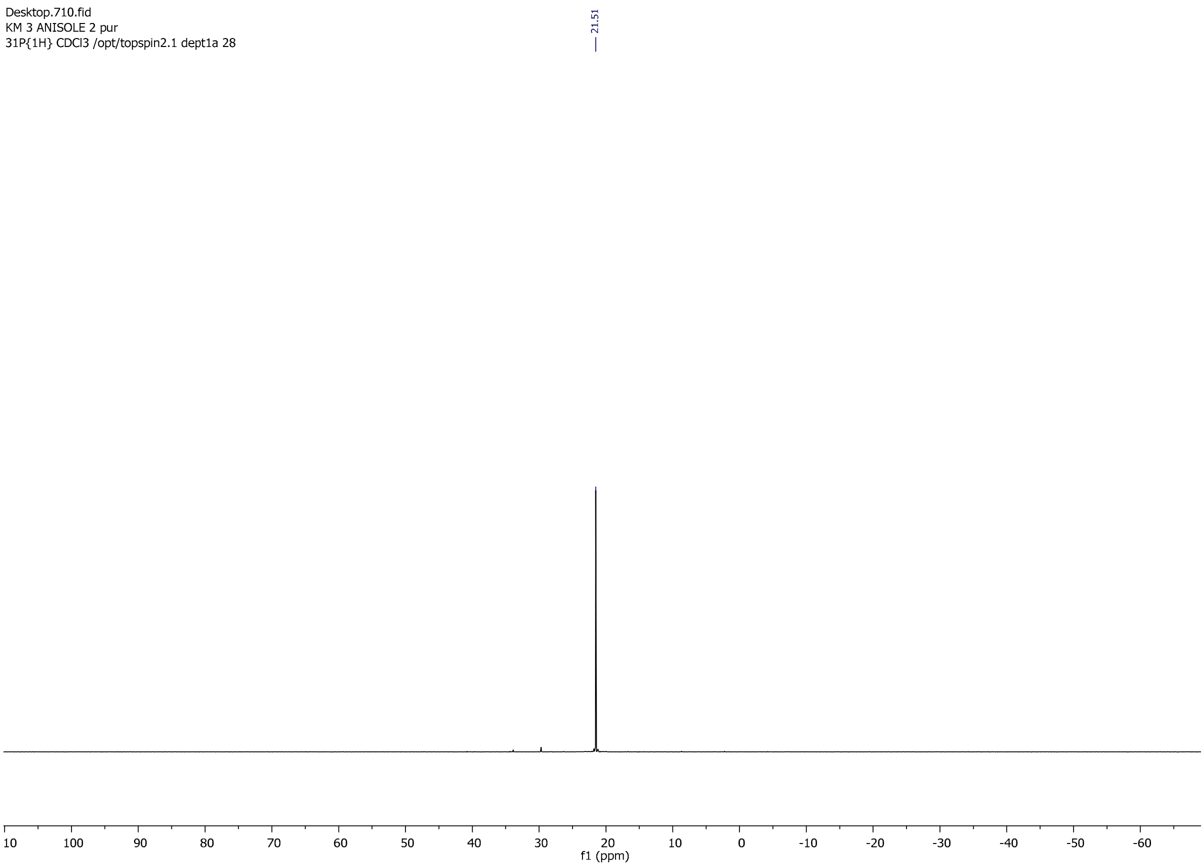

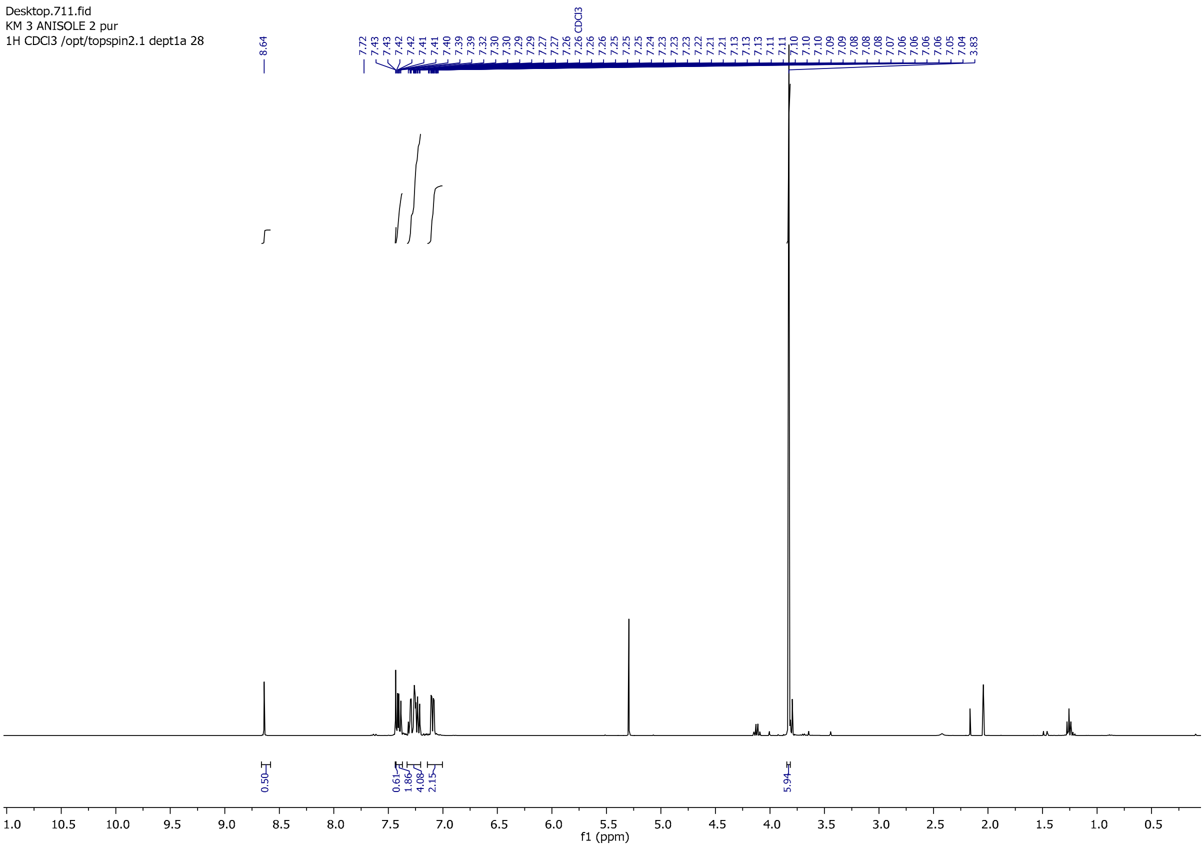

Bis(4-methoxyphenyl)phosphine oxide 1g

Bis(3-fluorophenyl)phosphine oxide 1h.

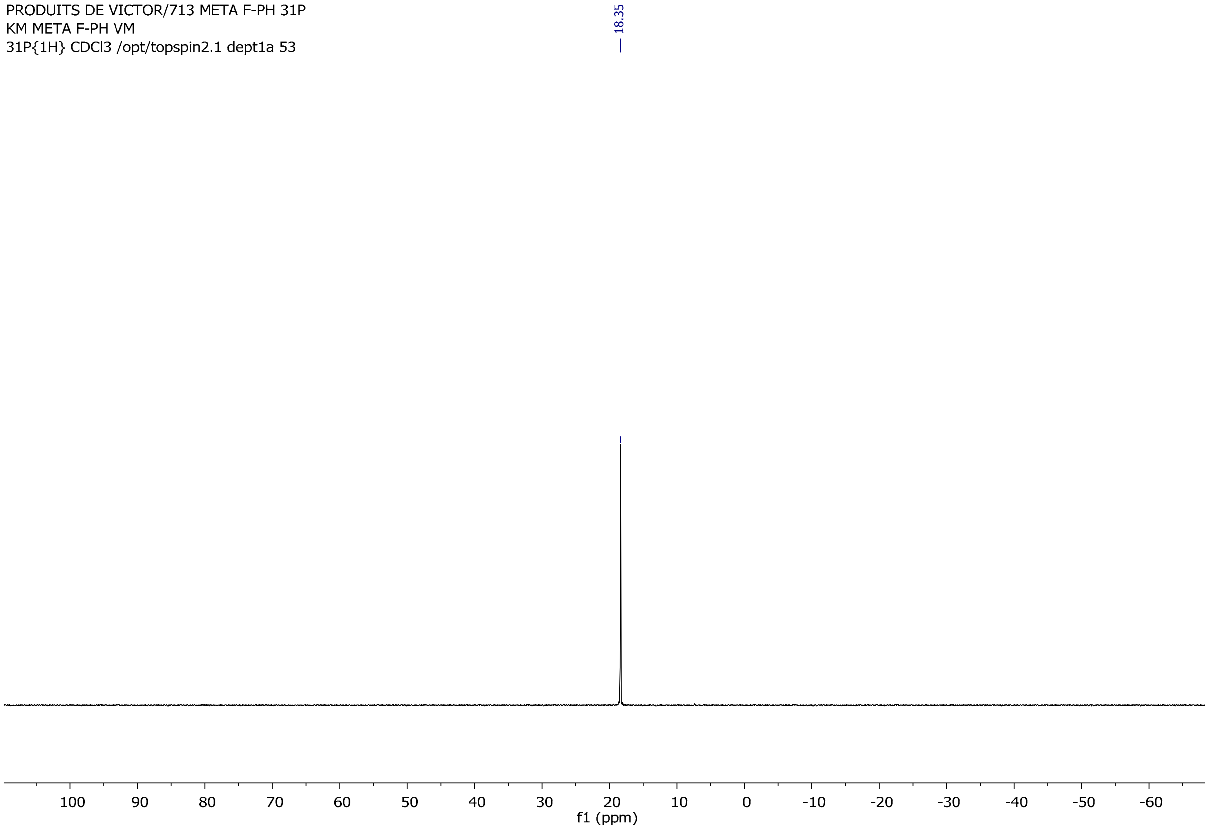

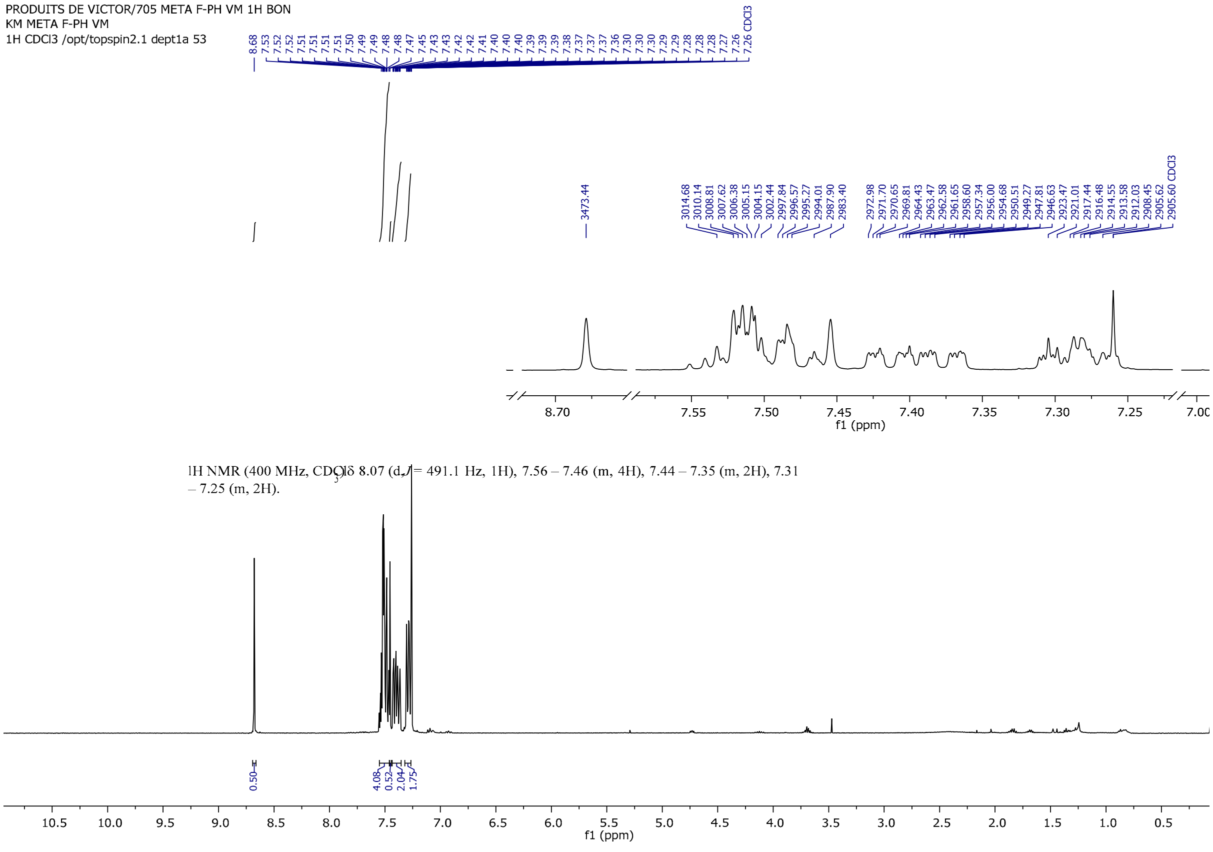

1H NMR (400 MHz, $CDCl_3$) δ 8.07 (d, *J* = 491.1 Hz, 1H), 7.56 – 7.46 (m, 4H), 7.44 – 7.35 (m, 2H), 7.31 – 7.25 (m, 2H).

Bis(4-fluorophenyl)phosphine oxide 1i.

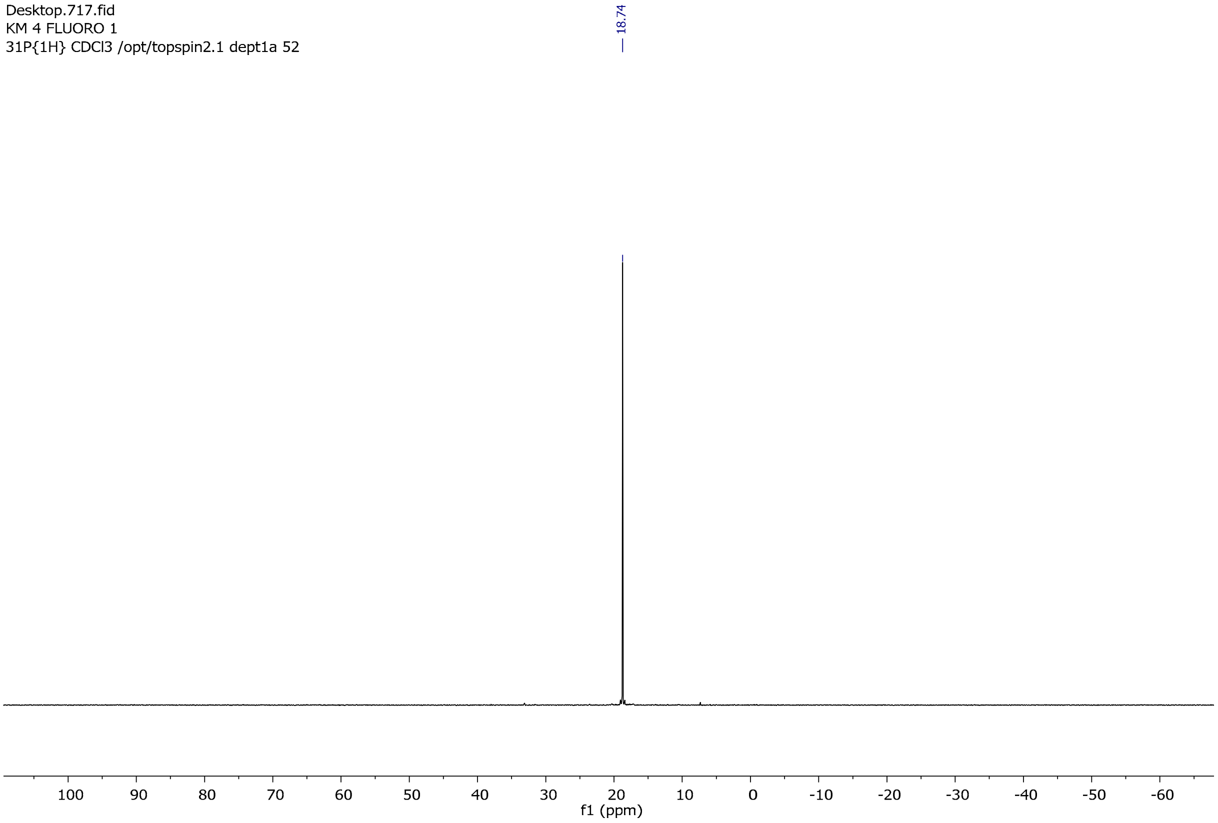

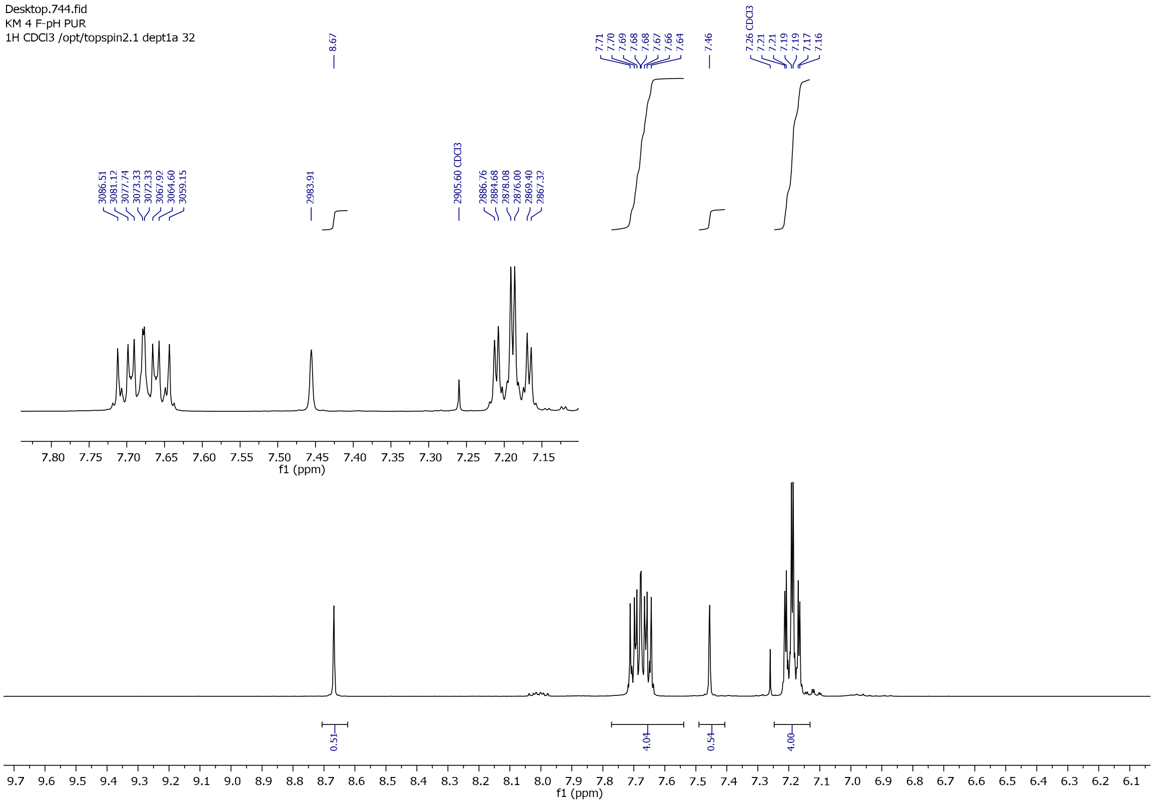

Bis(3-chlorophenyl)phosphine oxide 1j.

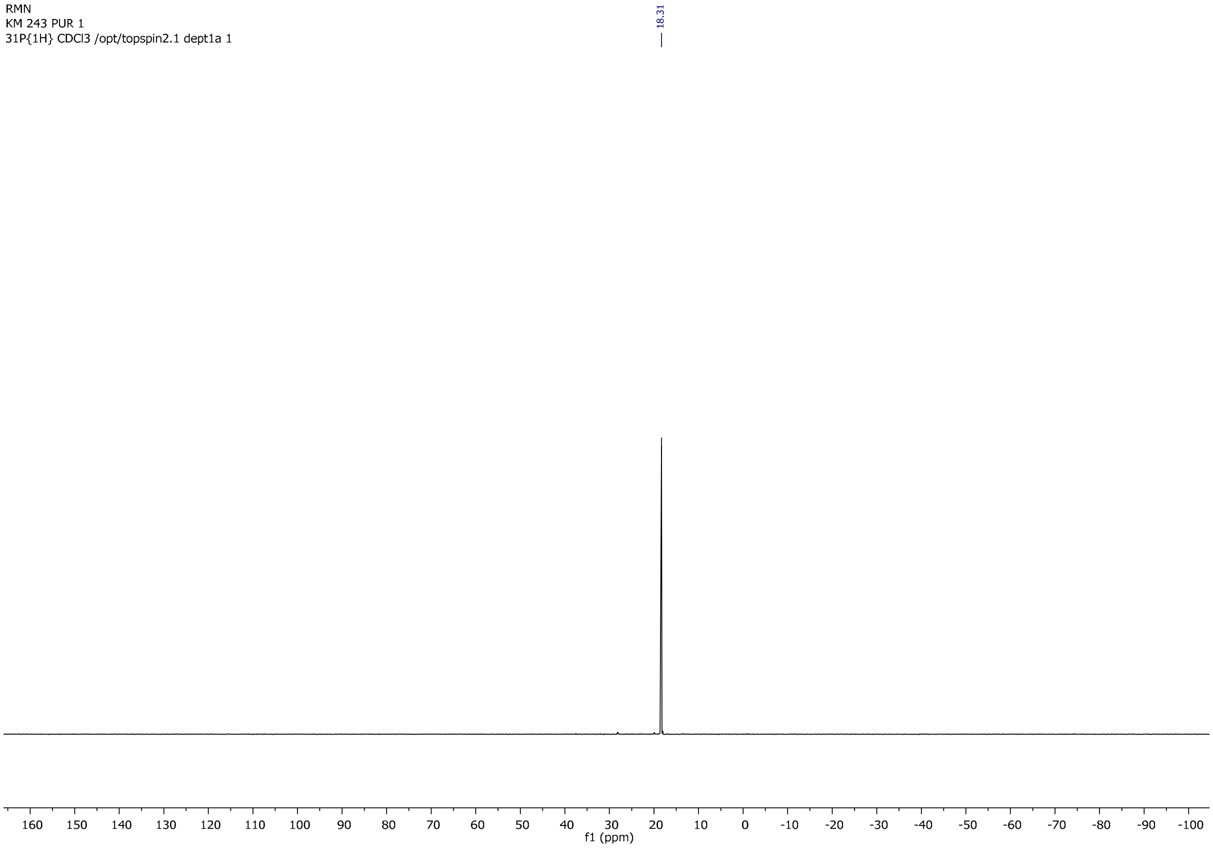

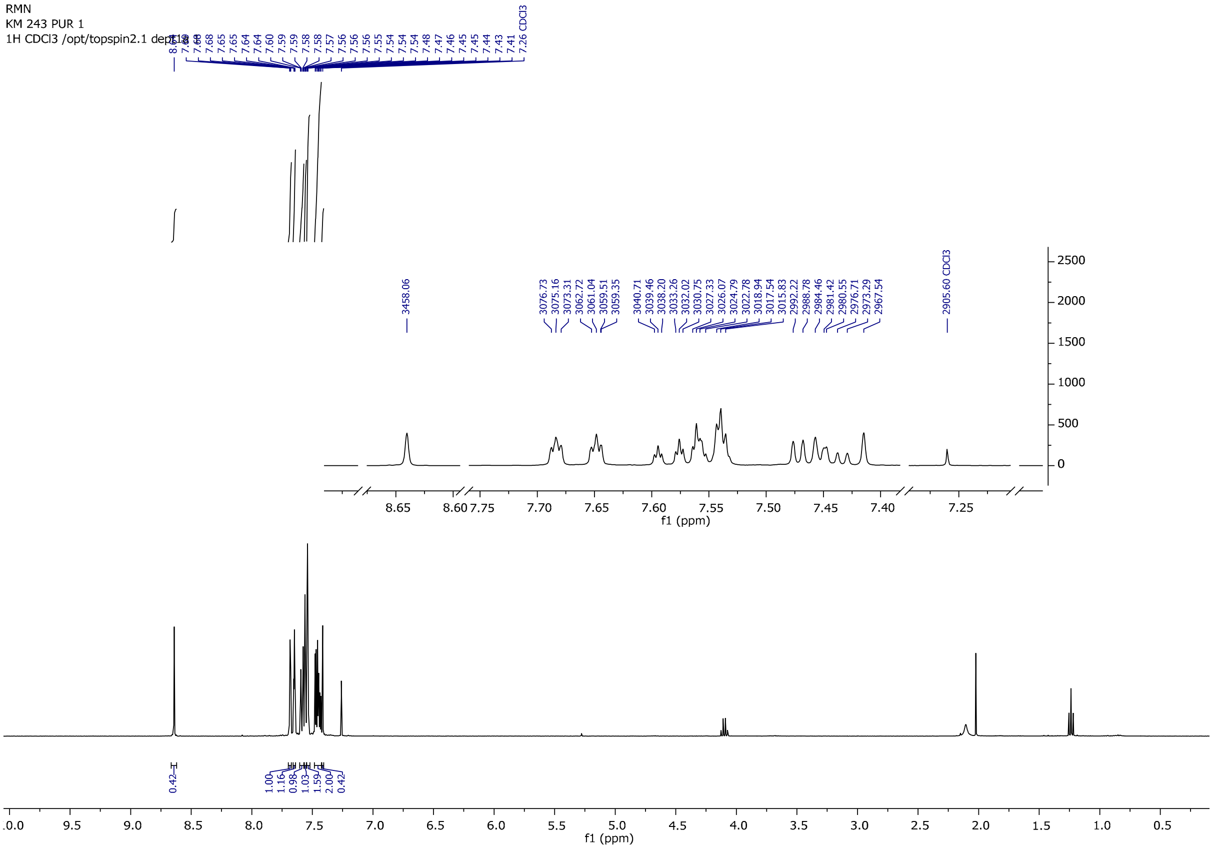

Bis(4-chlorophenyl)phosphine oxide 1k.

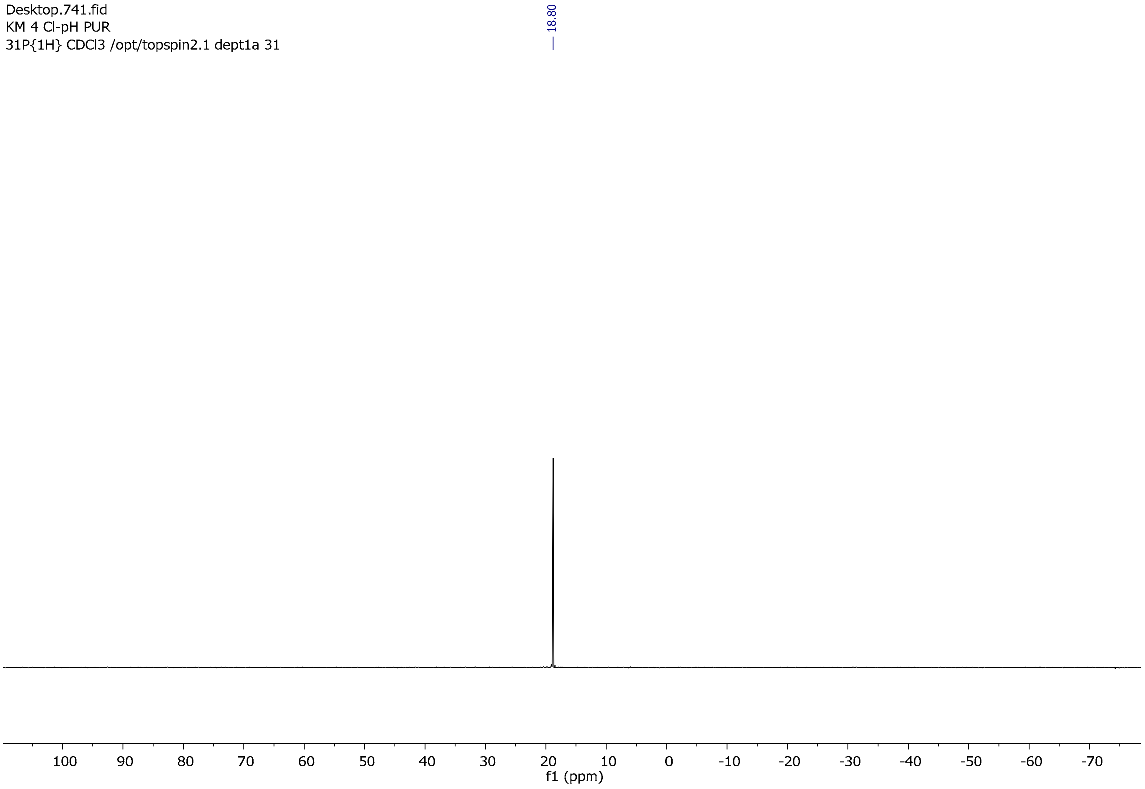

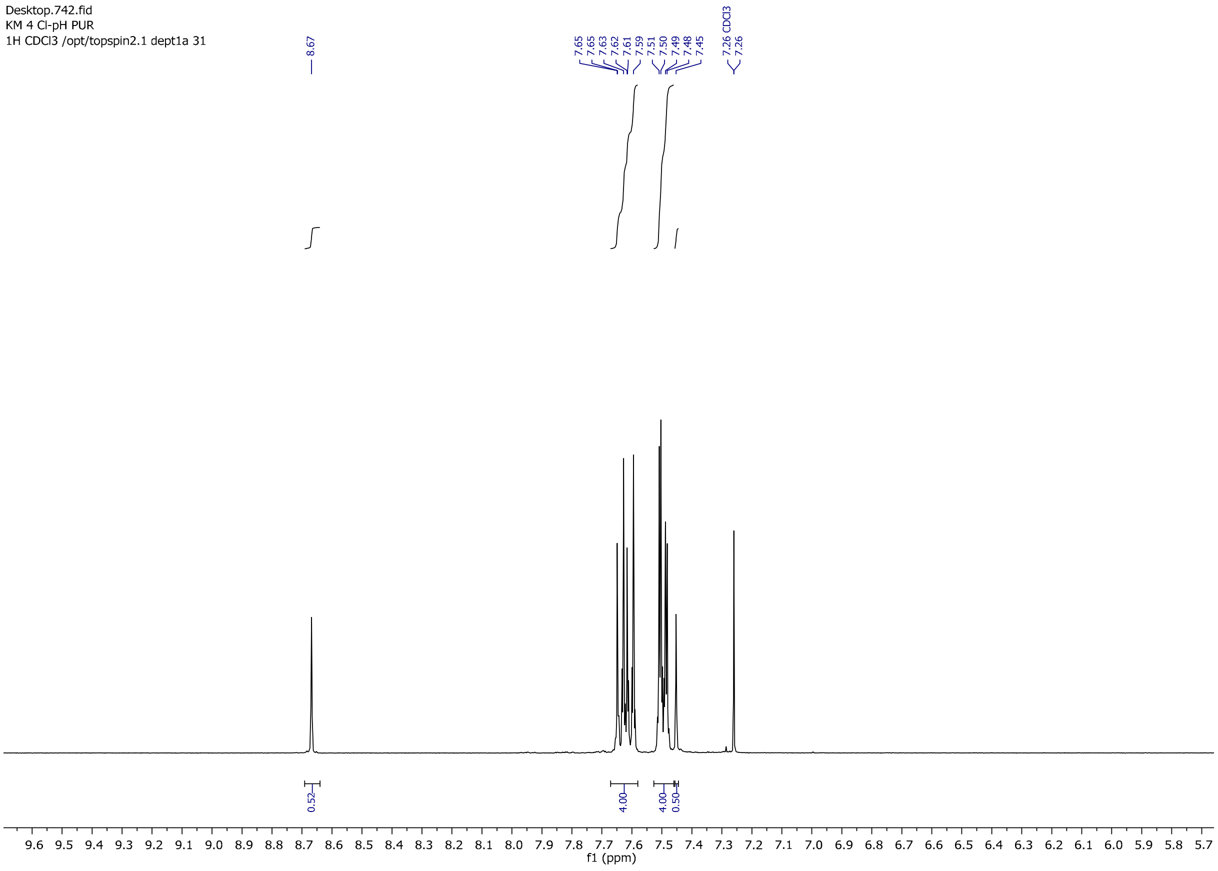

Bis(2,3-dichlorophenyl)phosphine oxide 1l.

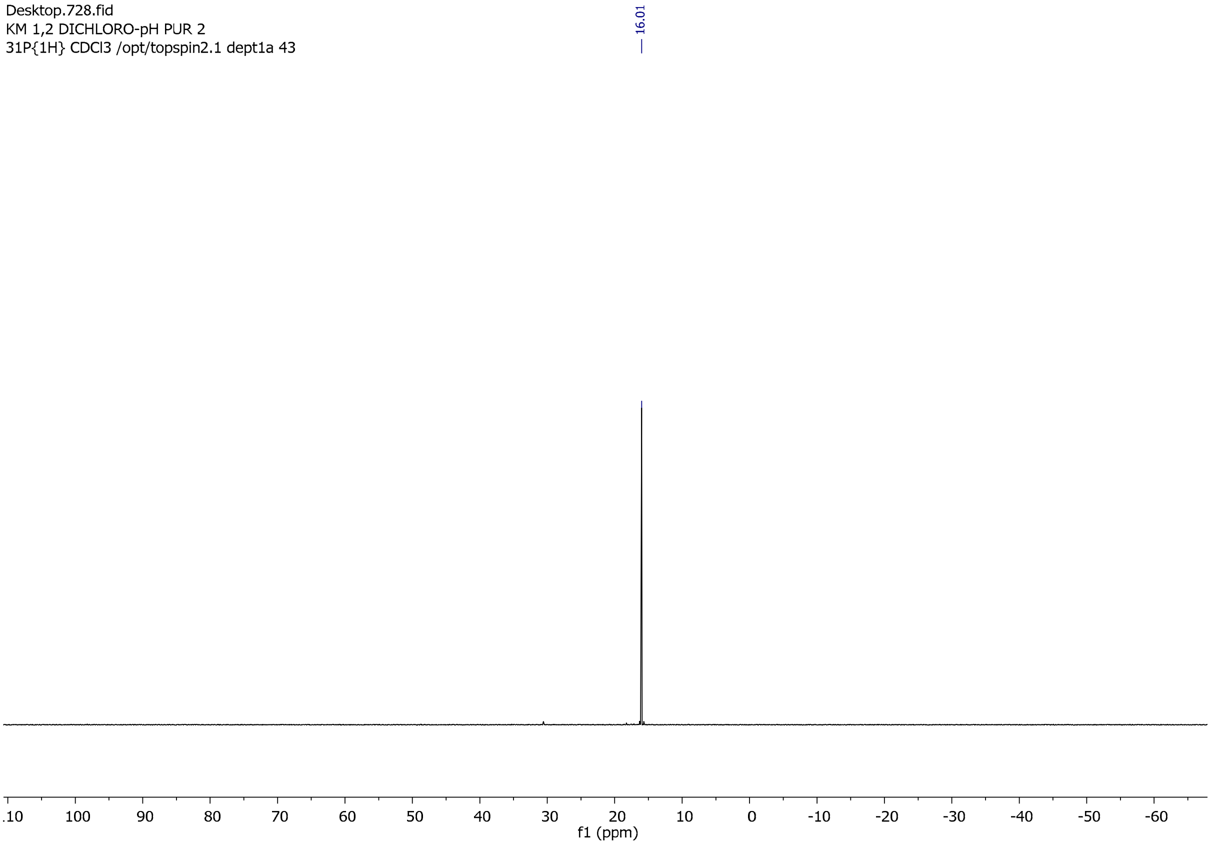

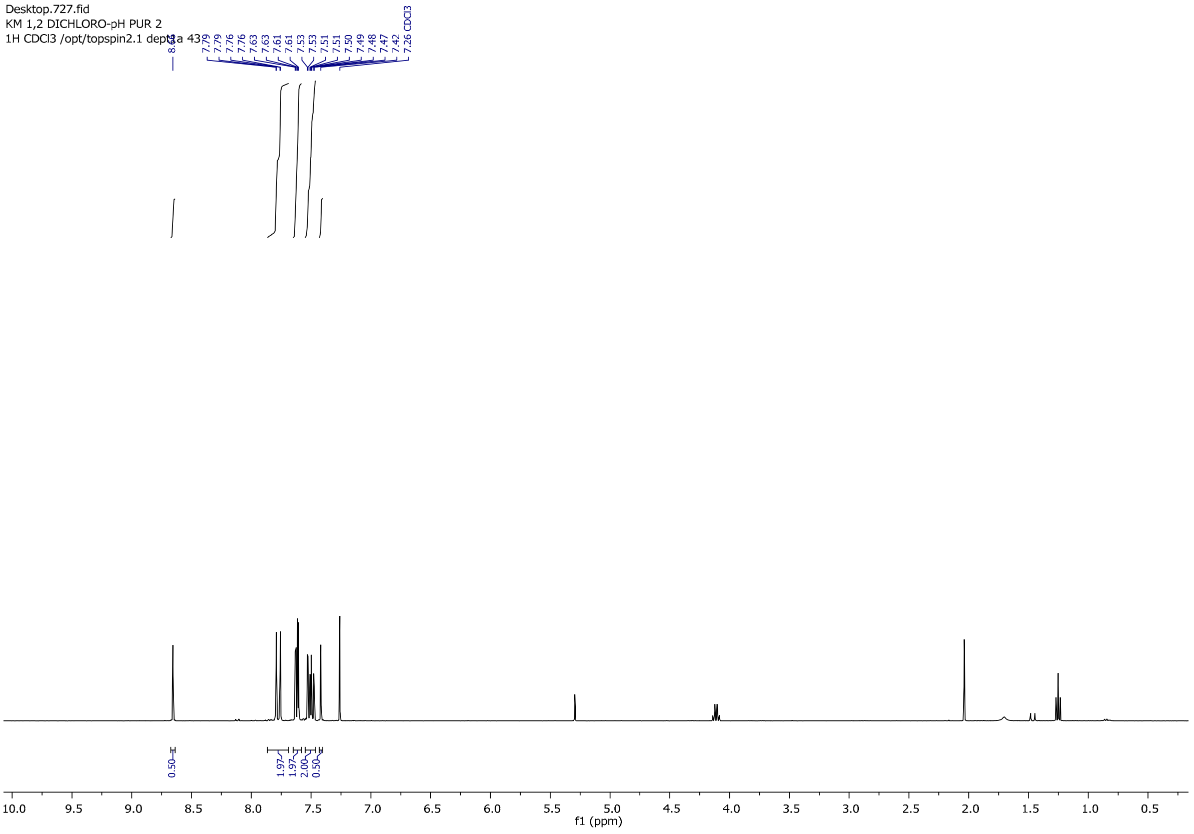

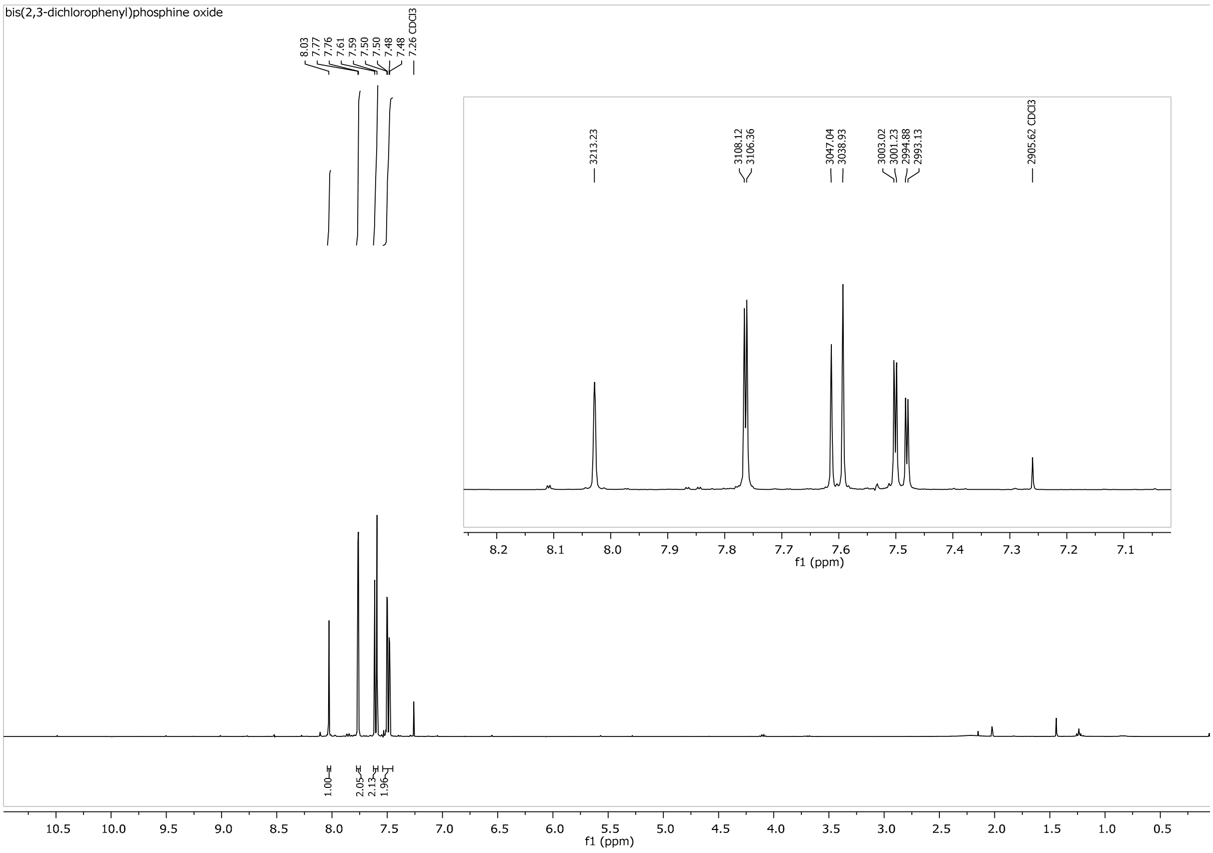

bis(2,3-dichlorophenyl)phosphine oxide
8.03
7.77
7.76
7.61
7.59
7.50
7.50
7.48
7.48
7.26 CDCl3
3213.23
3108.12
3106.36
3047.04
3038.93
3003.02
3001.23
2994.88
2993.13
2905.62 CDCl3
1.00
2.05
2.13
1.96
f1 (ppm)
f1 (ppm)

Bis(3,4,5-trichlorophenyl)phosphine oxide 1m.

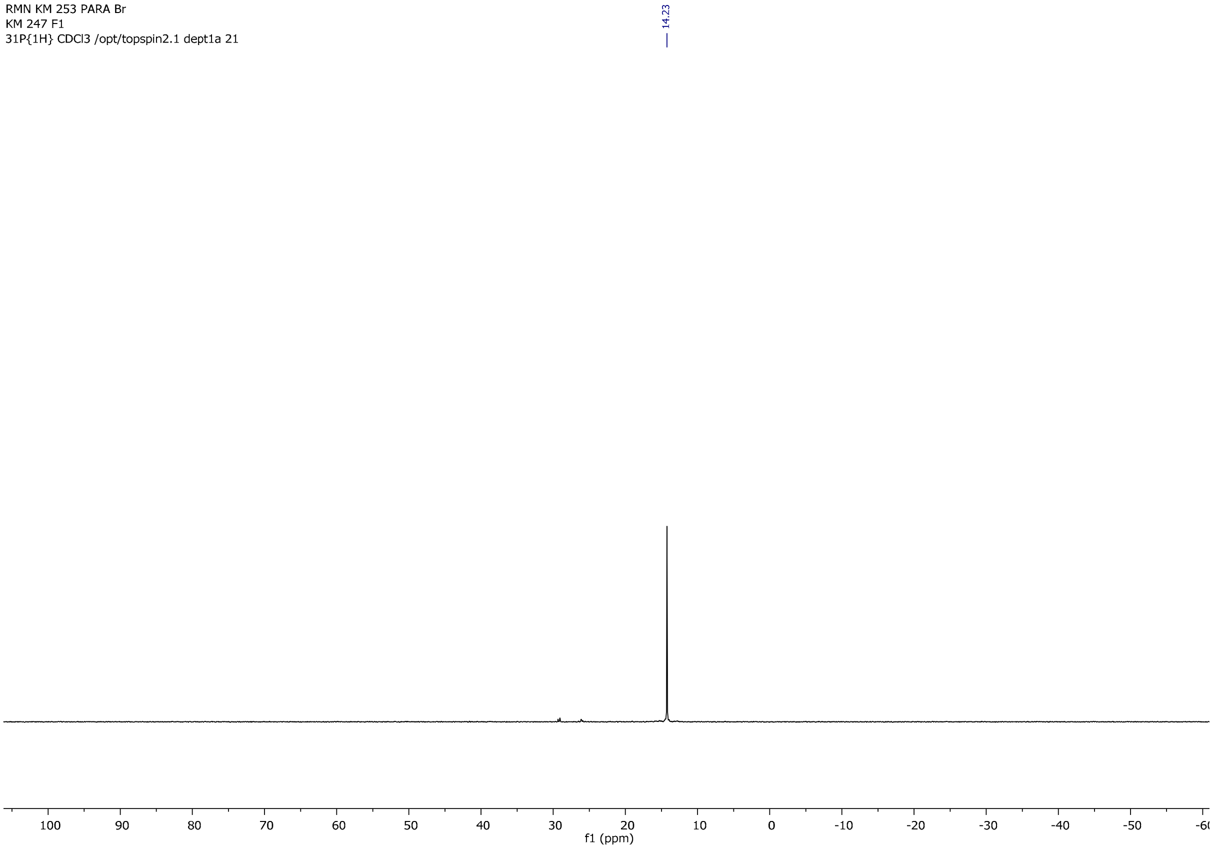

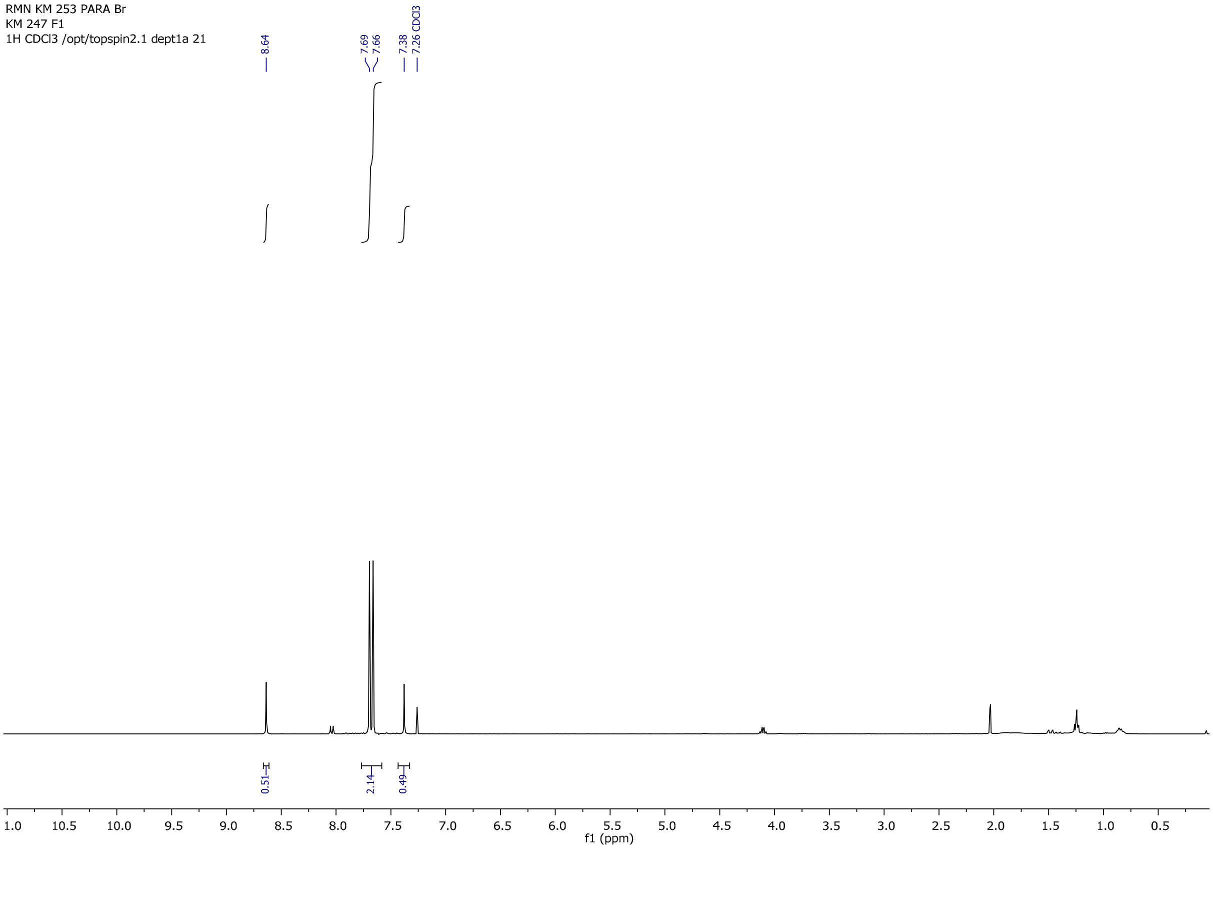

Bis(4-bromophenyl)phosphine oxide 1n.

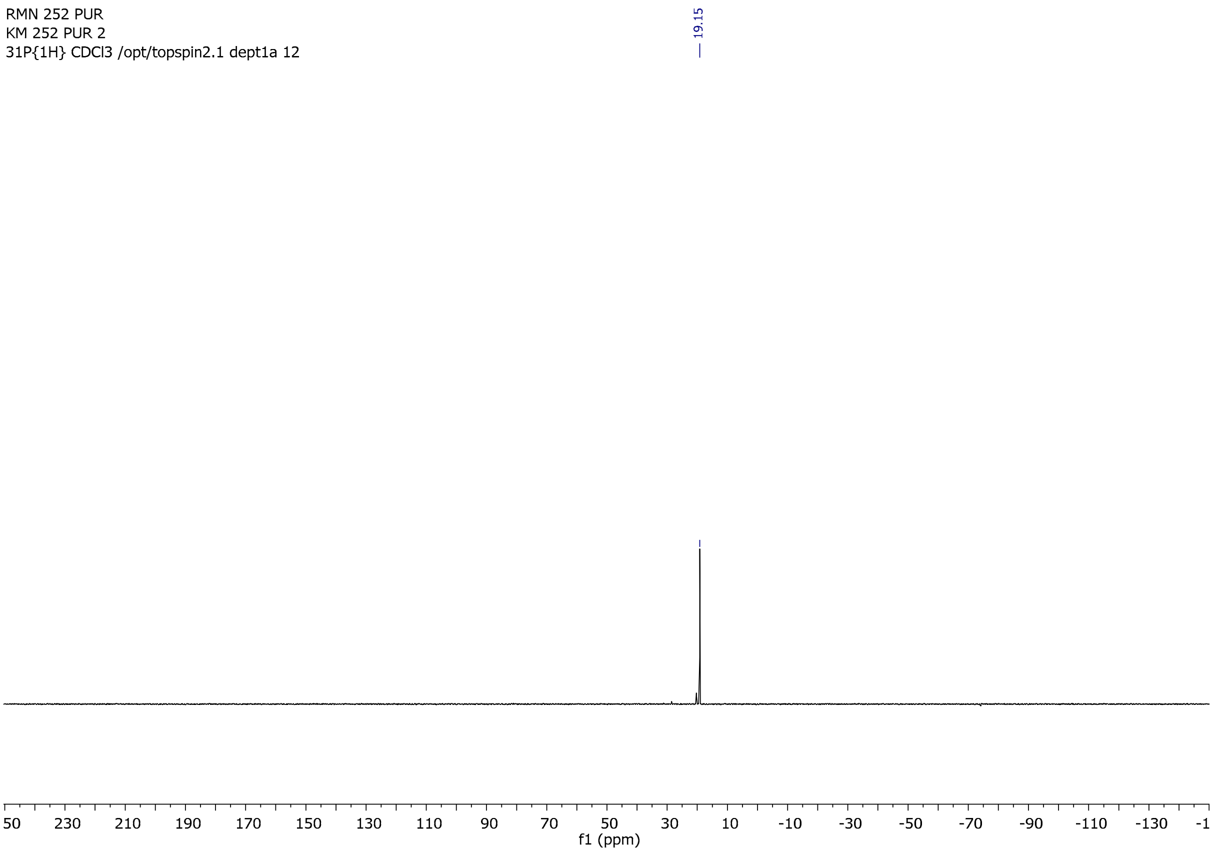

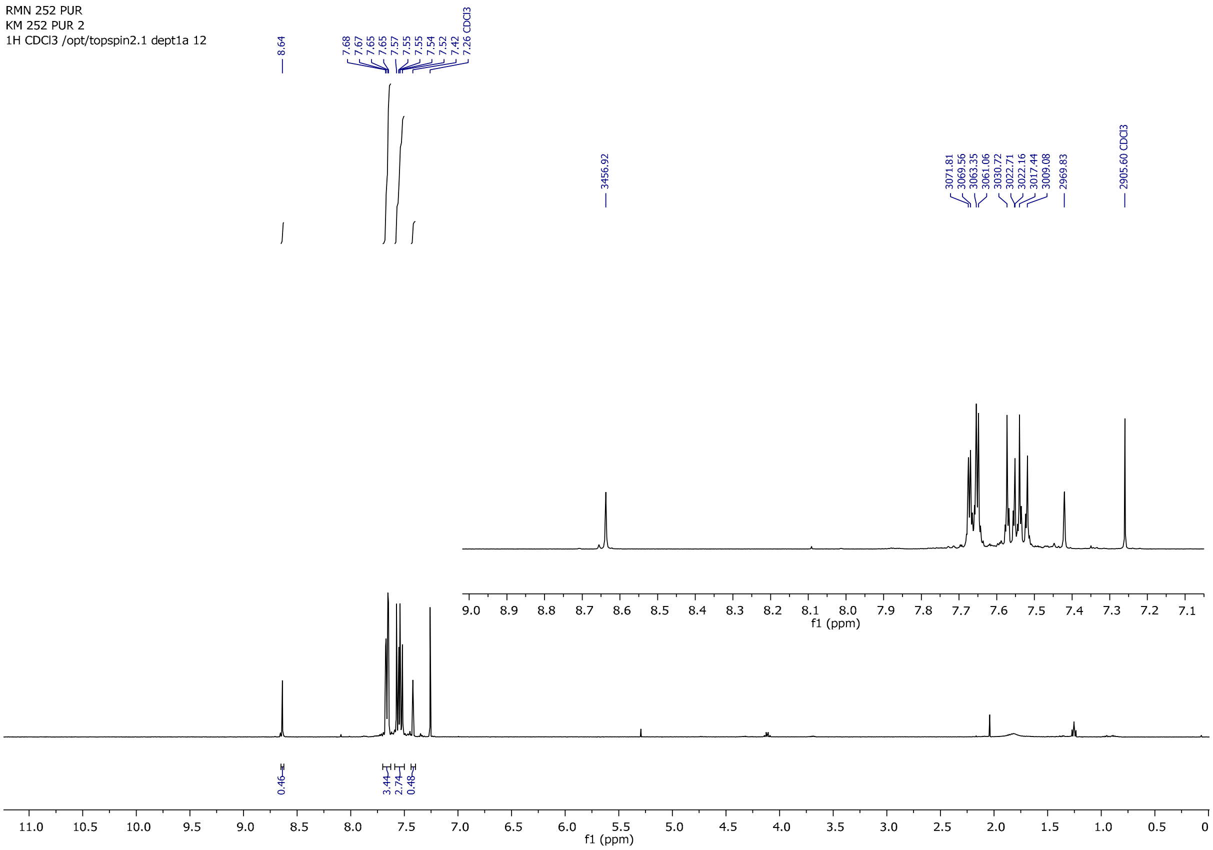

4-(diphenylphosphoryl)chroman-2-one COUM-H

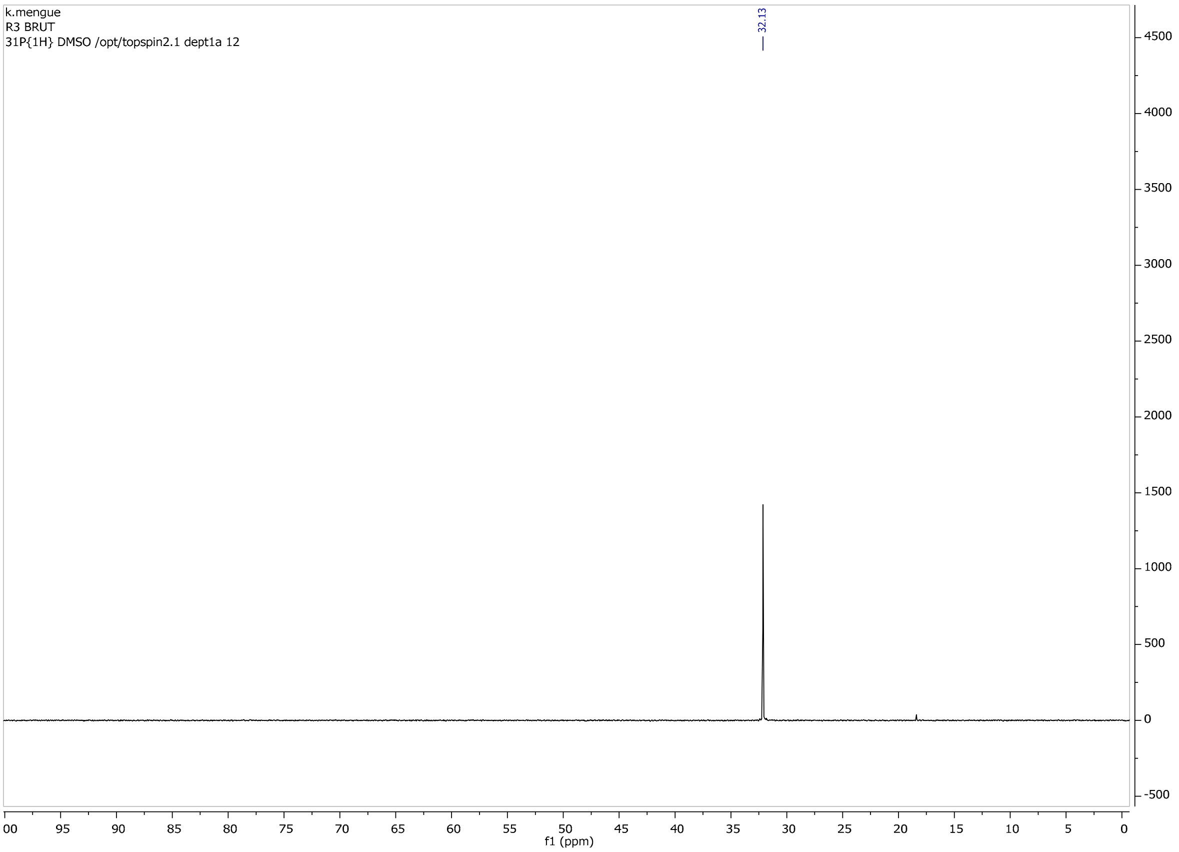

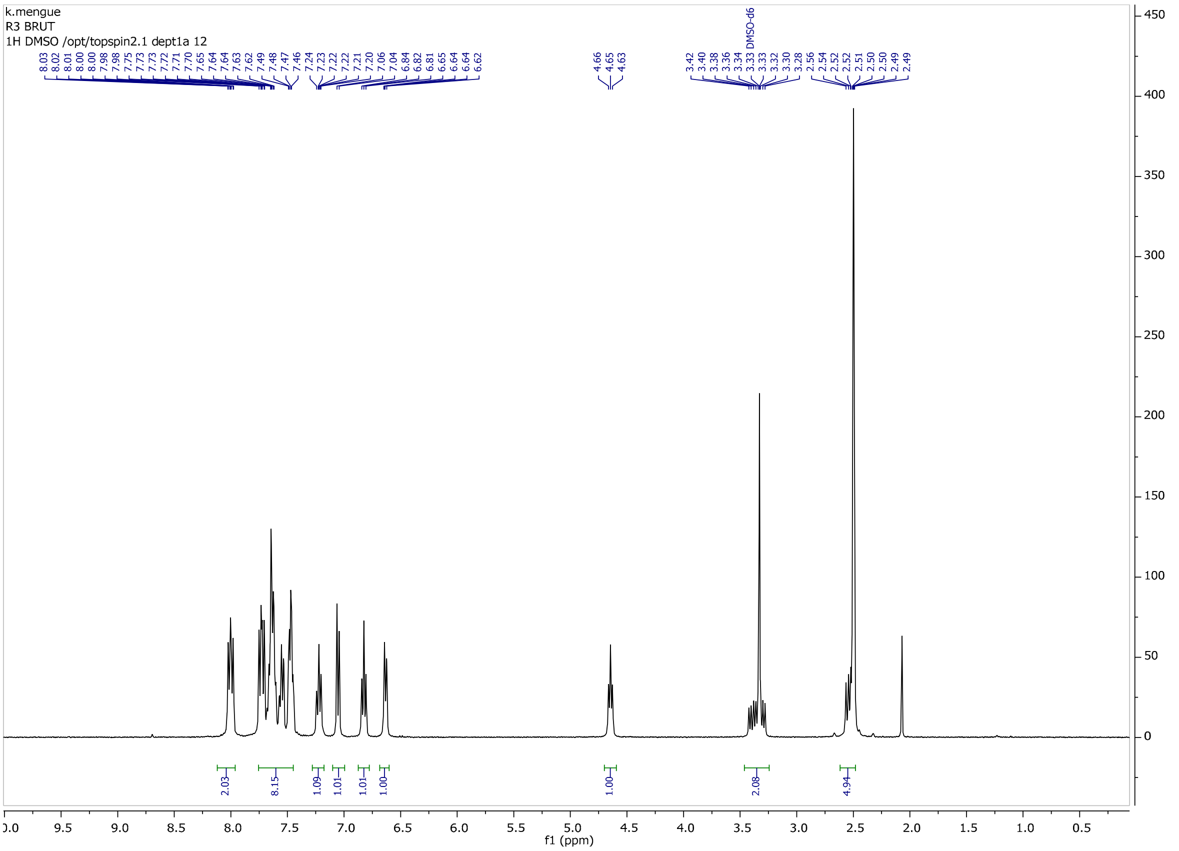

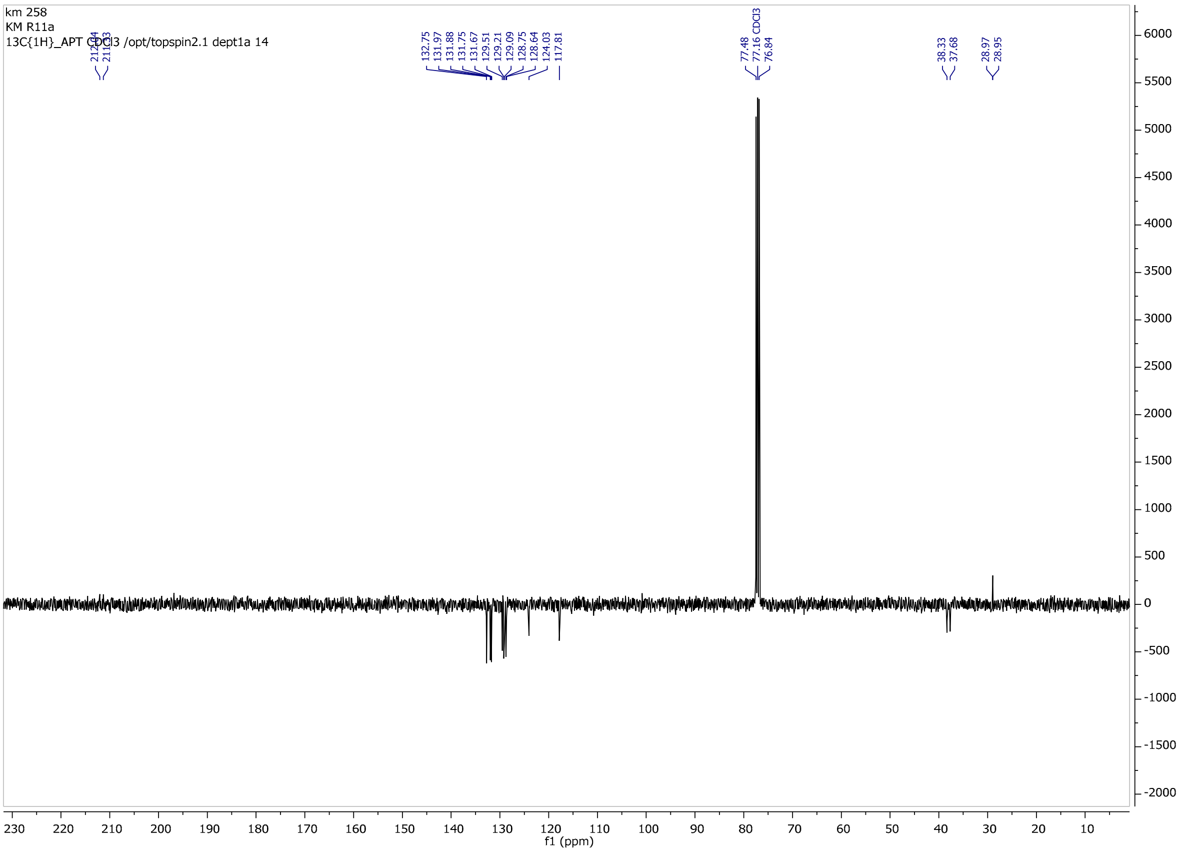

Mass Result

Analysis Info

Sample Name **MMN-3 a _ AR 11 a**

Acquisition Date 6/29/2023 4:43:02 PM

Instrument / Ser# micrOTOF-Q 228888.10300

Acquisition Parameter

Source Type	ESI	Ion Polarity	Positive	Scan Begin	50 m/z	Scan End	3000 m/z

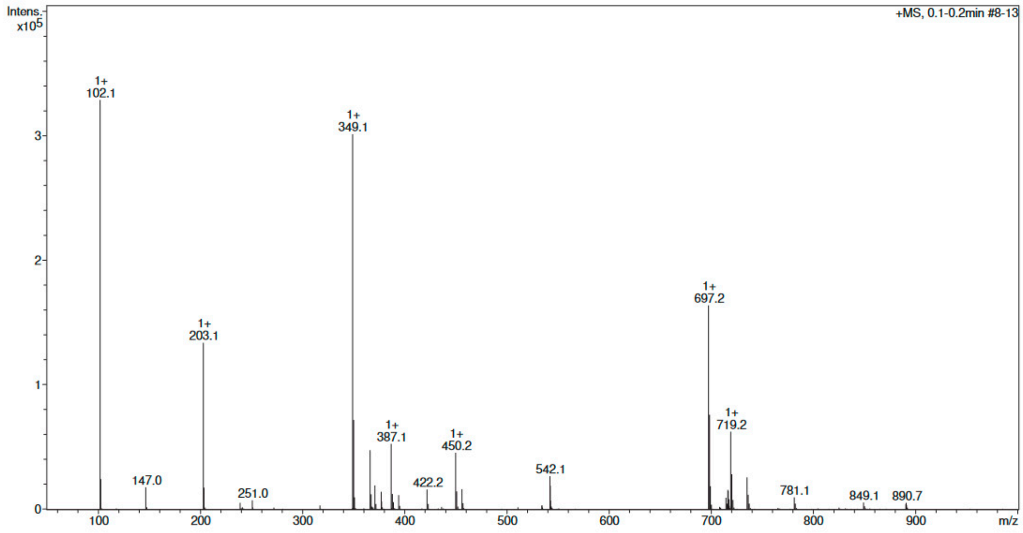

High Resolution Mass Result

Analysis Info

Sample Name **MMN-3 a _ AR 11 a**

Acquisition Date 6/29/2023 4:43:02 PM

Instrument / Ser# micrOTOF-Q 228888.10300

Acquisition Parameter

Source Type ESI   Ion Polarity Positive   Scan Begin 50 m/z   Scan End 3000 m/z

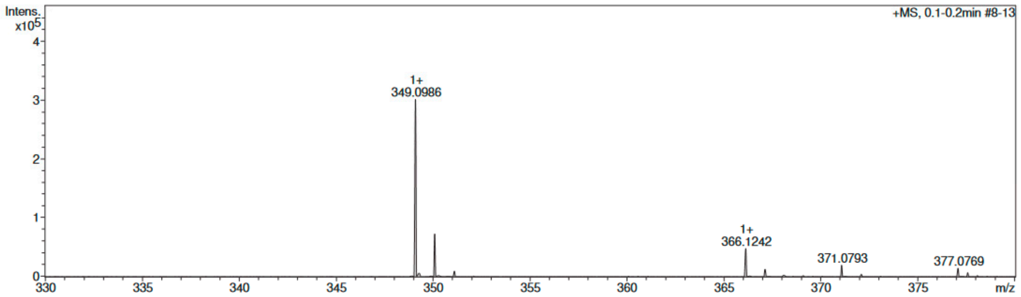

Meas. m/z	#	Ion Formula	Score	m/z	err [mDa]	err [ppm]	mSigma	rdb	e⁻ Conf	N-Rule	Adduct
349.098612	1	C21H18O3P	100.00	349.098807	0.2	0.6	4.9	13.5	even	ok	M+H

4-(di-o-tolylphosphoryl)chroman-2-one COUM-2oMe

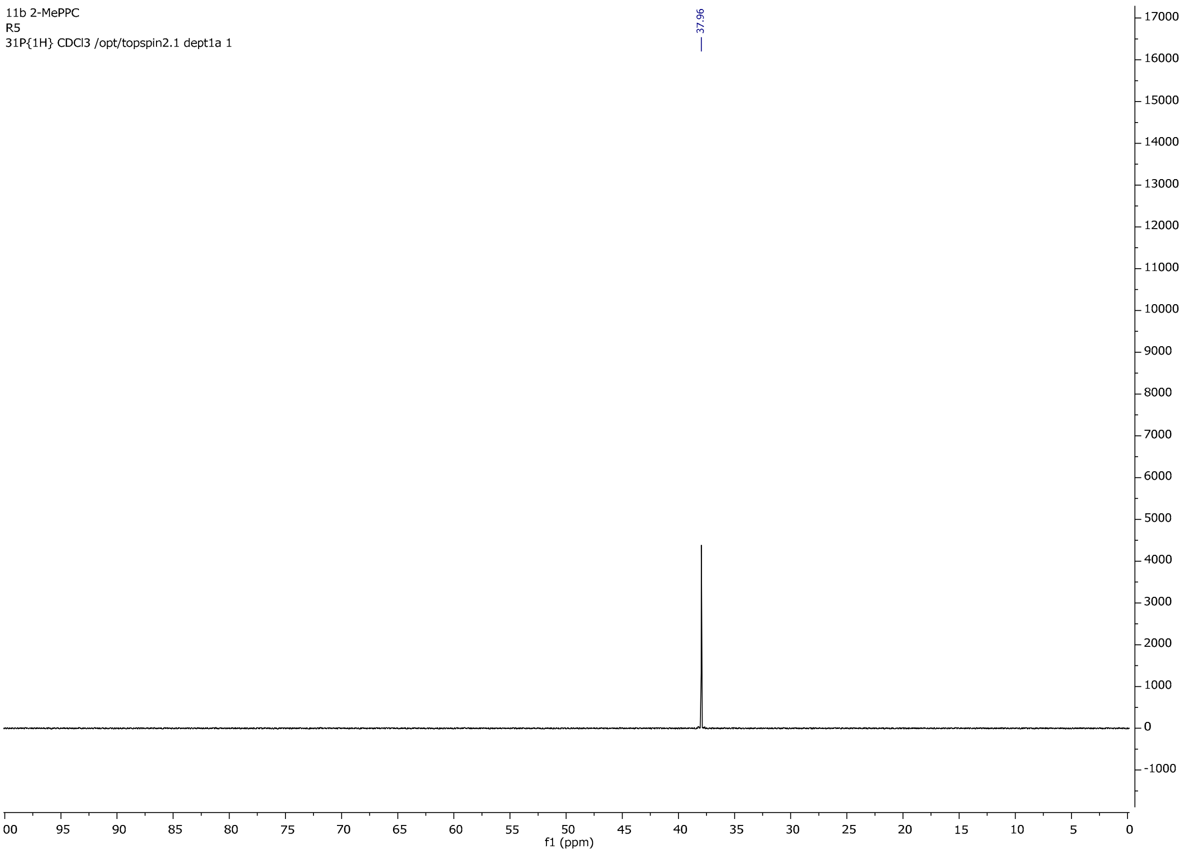

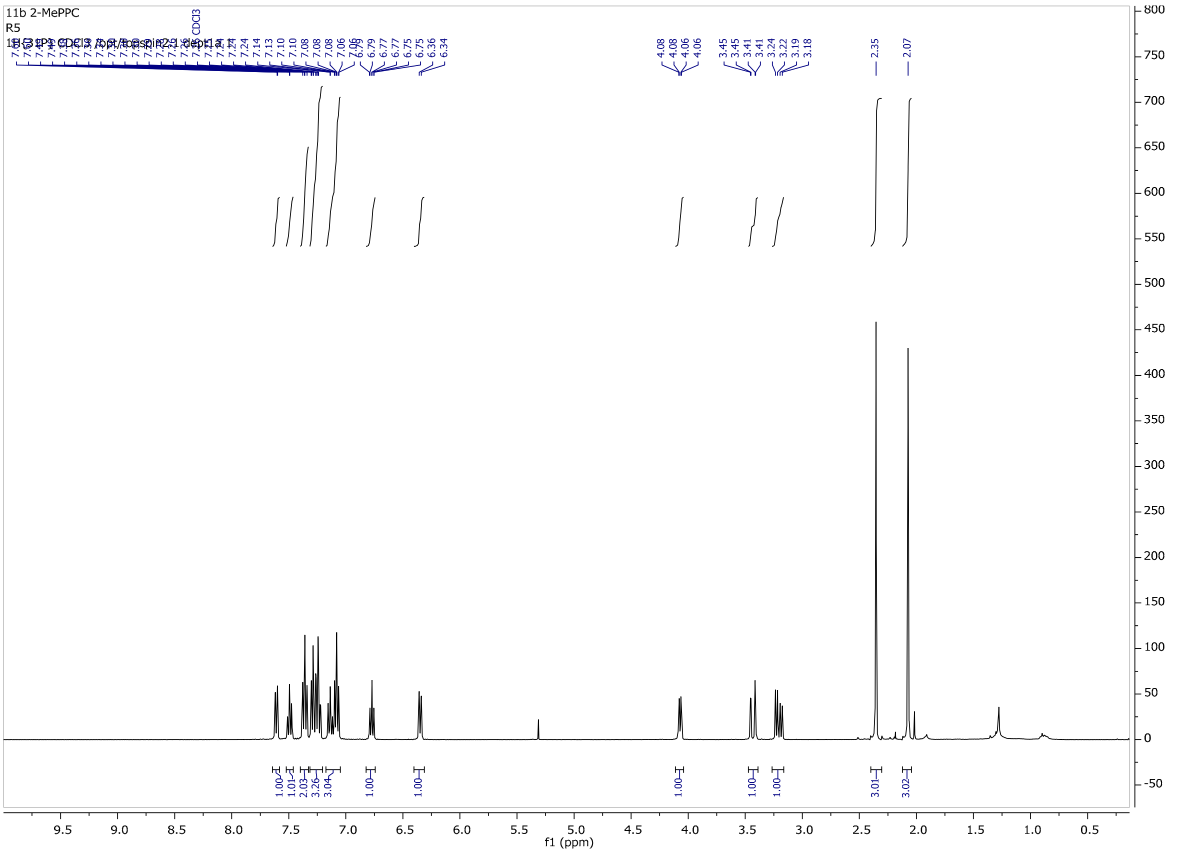

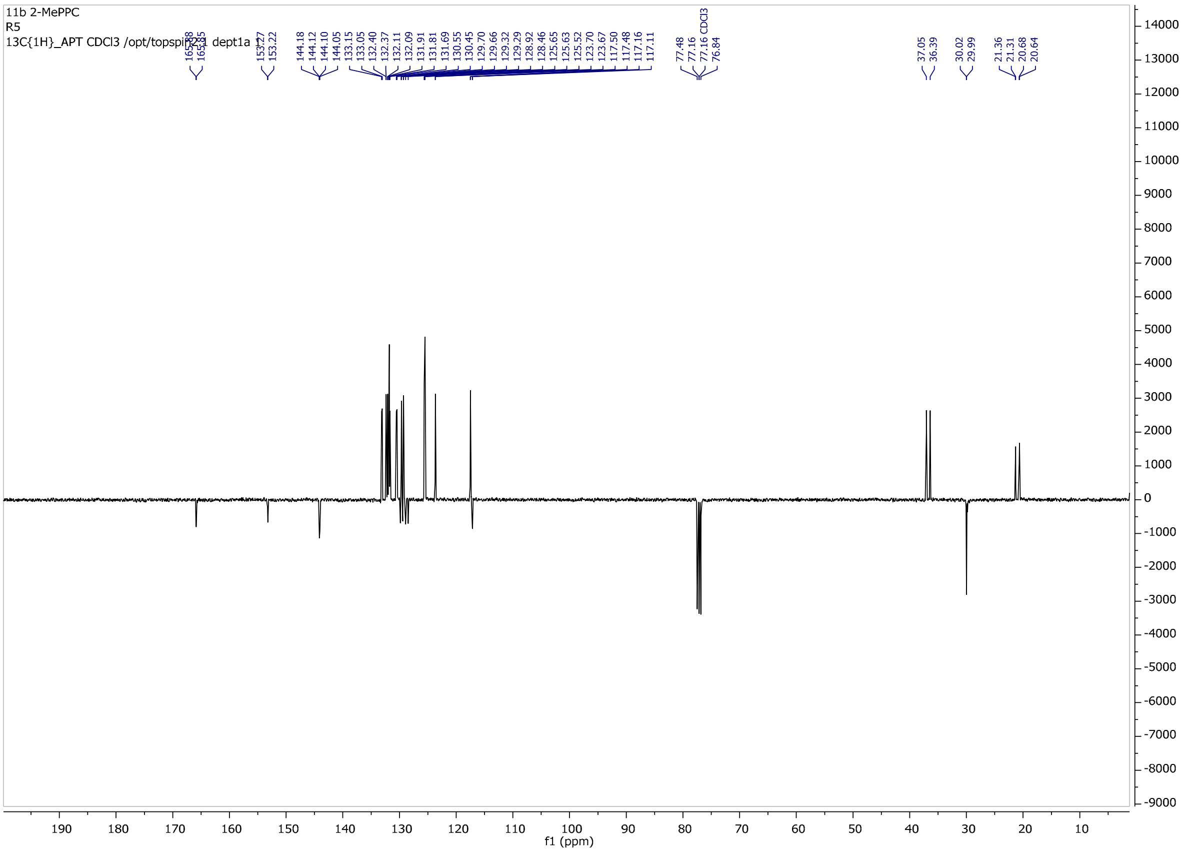

Mass Result

Analysis Info

Sample Name **MMN-3 b _ AR 11 b**

Acquisition Date 6/29/2023 4:46:04 PM

Instrument / Ser# micrOTOF-Q 228888.10300

Acquisition Parameter

Source Type ESI Ion Polarity Positive Scan Begin 50 m/z Scan End 3000 m/z

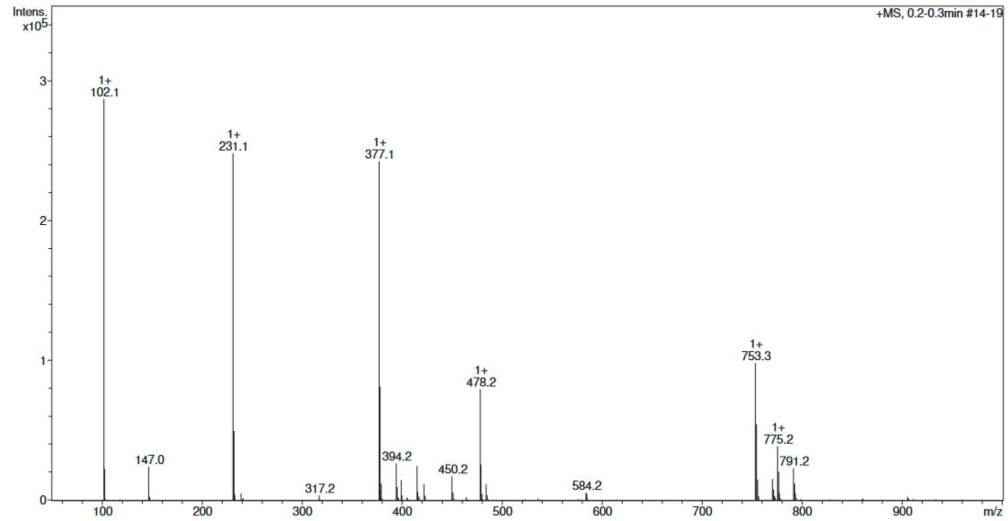

High Resolution Mass Result

**Analysis Info**		Acquisition Date	6/29/2023 4:46:04 PM	
Sample Name	**MMN-3 b _ AR 11 b**	Instrument / Ser#	micrOTOF-Q	228888.10300

Acquisition Parameter

Source Type	ESI	Ion Polarity	Positive	Scan Begin	50 m/z	Scan End	3000 m/z

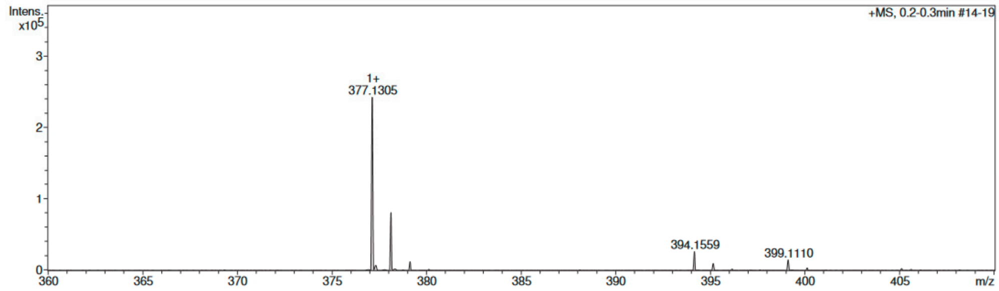

Meas. m/z	#	Ion Formula	Score	m/z	err [mDa]	err [ppm]	mSigma	rdb	e⁻ Conf	N-Rule	Adduct
377.1305	1	C23H22O3P	100.00	377.1301	-0.4	-0.9	41.7	13.5	even	ok	M+H

4-(di-m-tolylphosphoryl)chroman-2-one COUM-2mMe

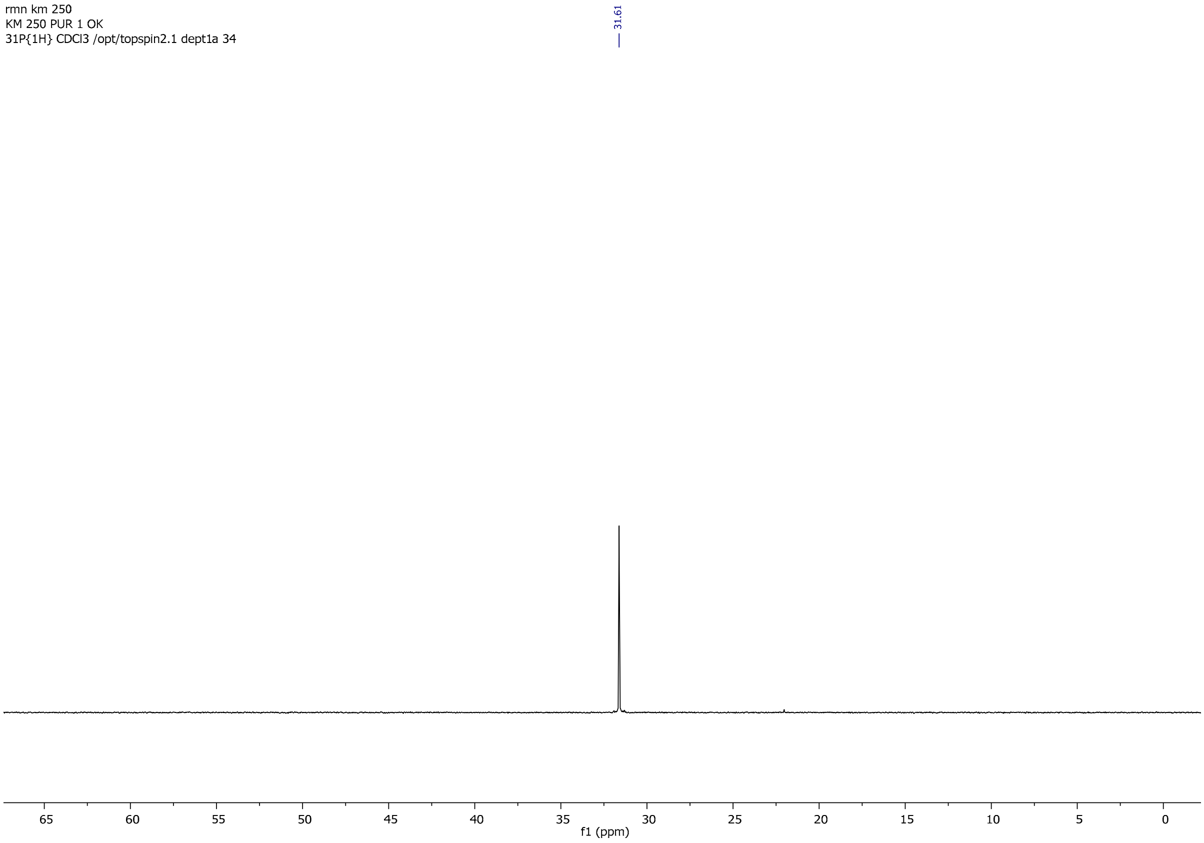

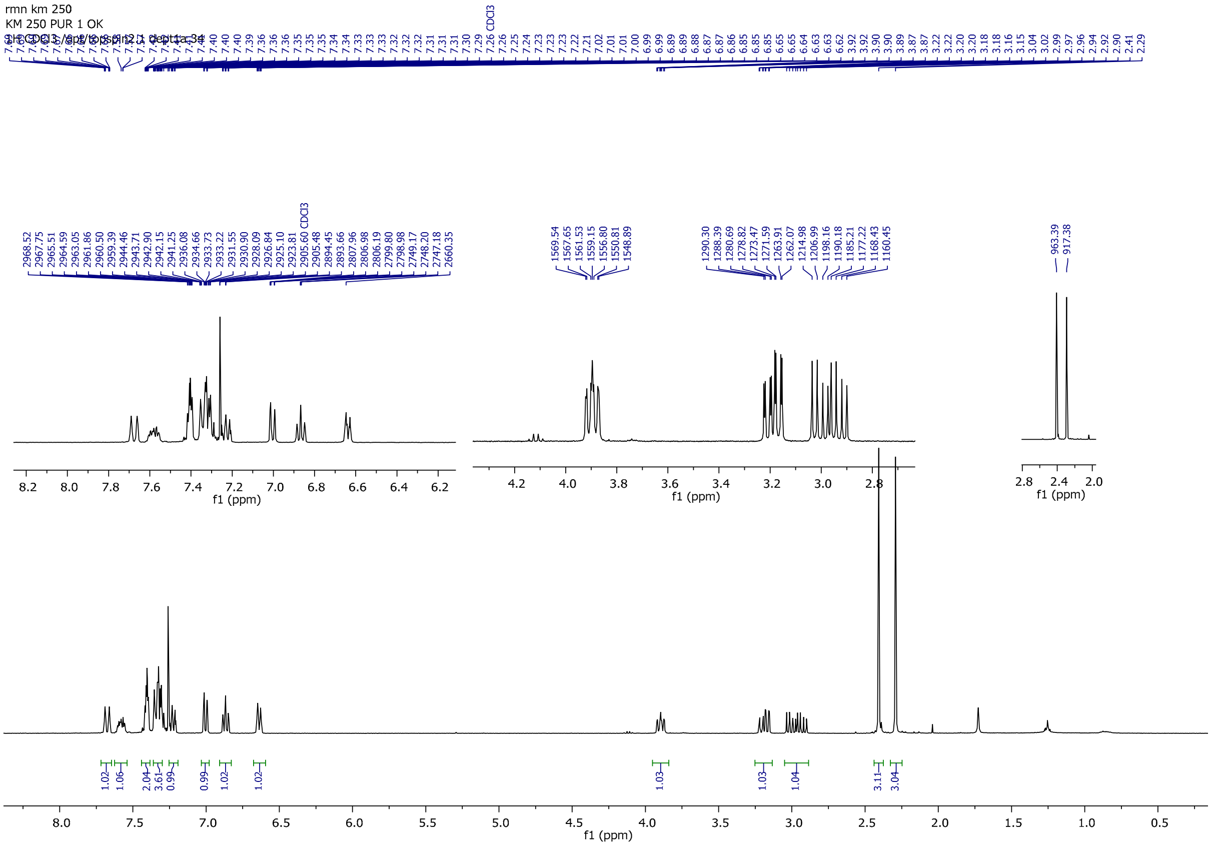

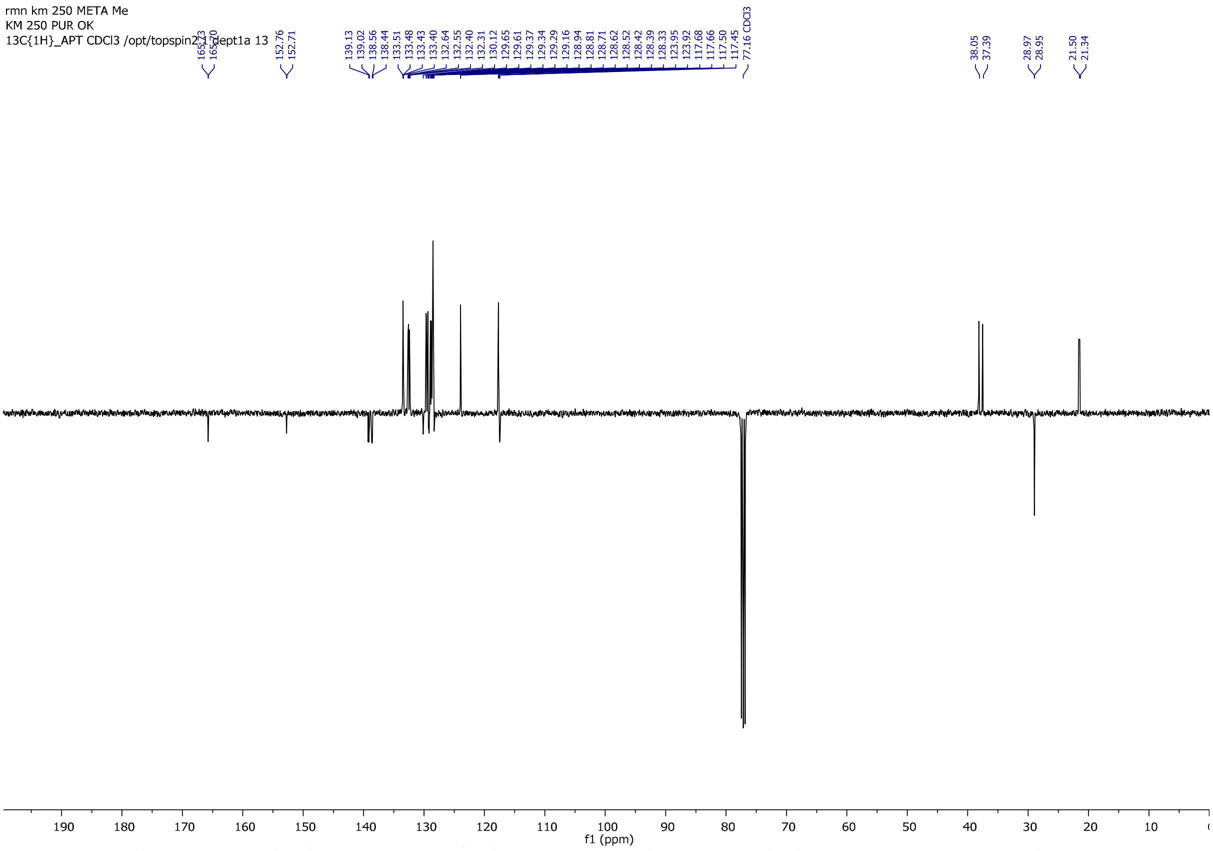

Mass Result

Analysis Info

Sample Name **MMN-6 a _ KM16**

Acquisition Date 2/1/2024 11:22:37 AM

Instrument / Ser# micrOTOF-Q 228888.10300

Acquisition Parameter

Source Type ESI   Ion Polarity Positive   Scan Begin 50 m/z   Scan End 3000 m/z

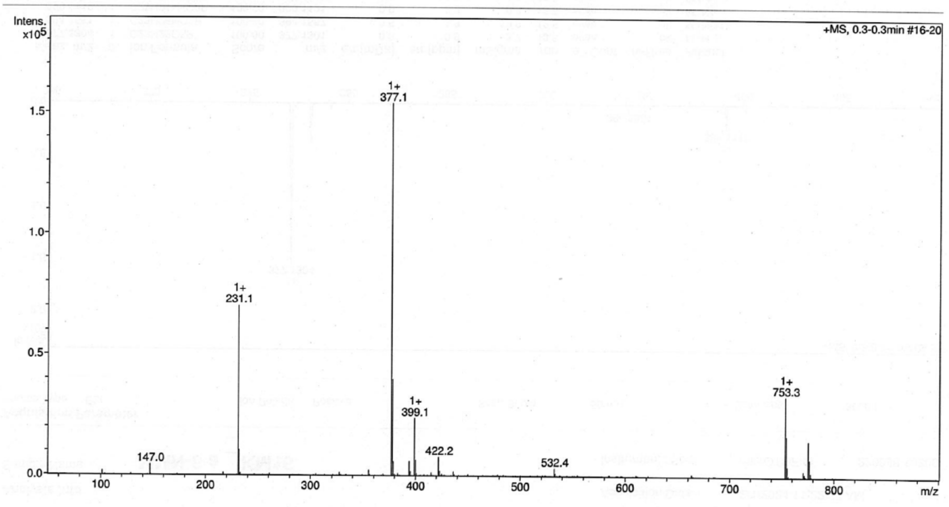

High Resolution Mass Result

Analysis Info

Sample Name **MMN-6 a _ KM16**

Acquisition Date 2/1/2024 11:22:37 AM

Instrument / Ser# micrOTOF-Q 228888.10300

Acquisition Parameter

Source Type ESI | Ion Polarity Positive | Scan Begin 50 m/z | Scan End 3000 m/z

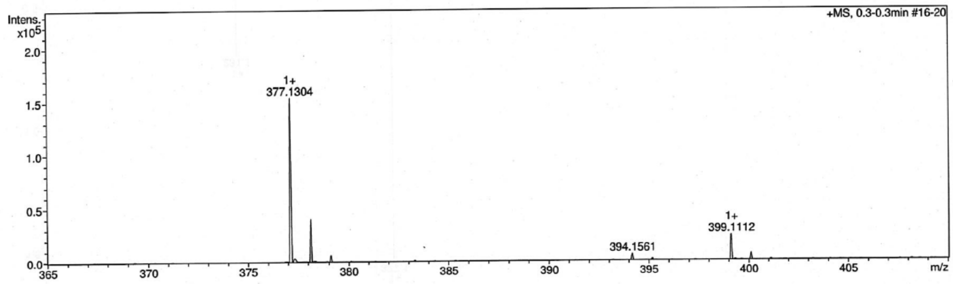

Meas. m/z	#	Ion Formula	Score	m/z	err [mDa]	err [ppm]	mSigma	rdb	e⁻ Conf	N-Rule	Adduct
377.1304	1	C23H22O3P	100.00	377.1301	0.3	0.8	3.7	13.5	even	ok	M+H
394.1561	1	C23H25NO3P	100.00	394.1567	0.5	1.3	19.8	12.5	even	ok	M+NH4
399.1112	1	C23H21NaO3P	100.00	399.1121	0.9	2.2	6.9	13.5	even	ok	M+Na
753.2534	1	C46H43O6P2	100.00	753.2529	0.5	0.7	1.8	26.5	even	ok	2M+H
770.2791	1	C46H46NO6P2	100.00	770.2795	-0.4	-0.5	31.0	25.5	even	ok	2M+NH4
775.2352	1	C46H42NaO6P2	100.00	775.2349	0.3	0.4	6.0	26.5	even	ok	2M+Na

4-(di-p-tolylphosphoryl)chroman-2-one COUM-2pMe

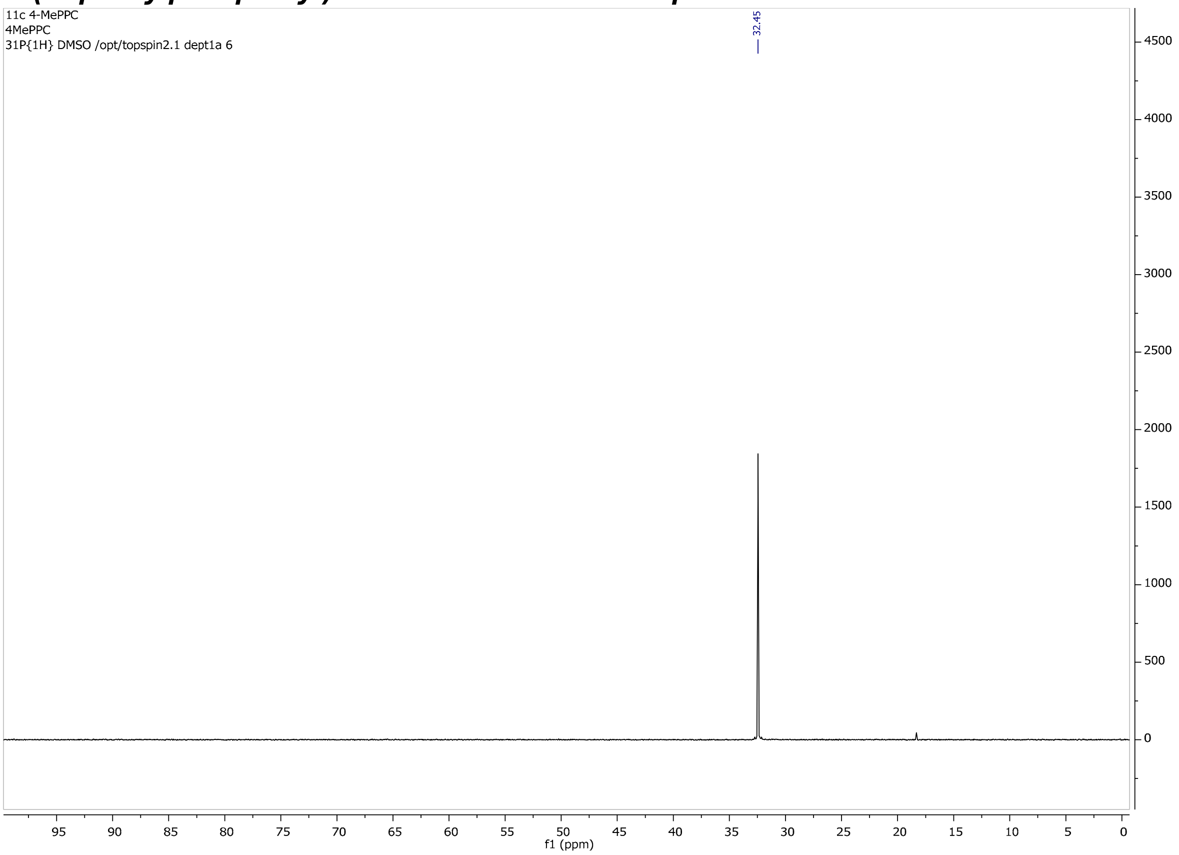

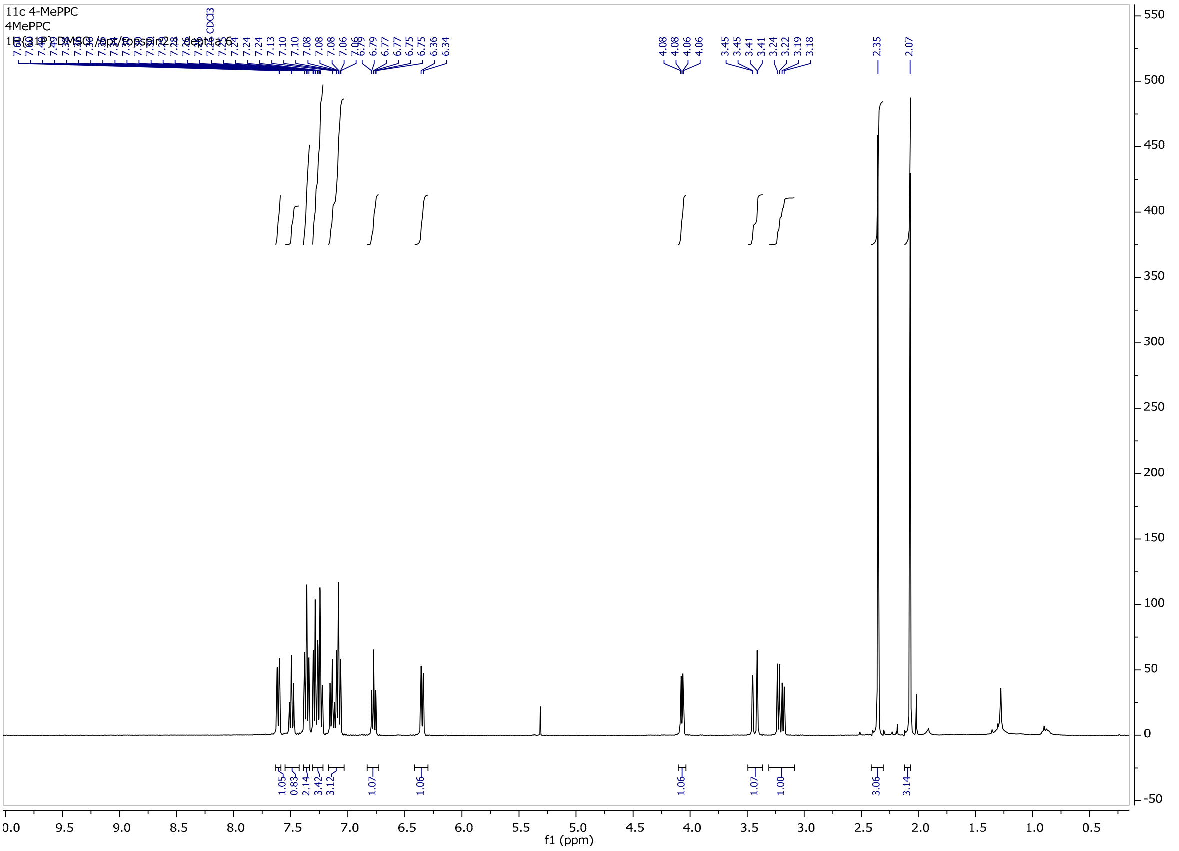

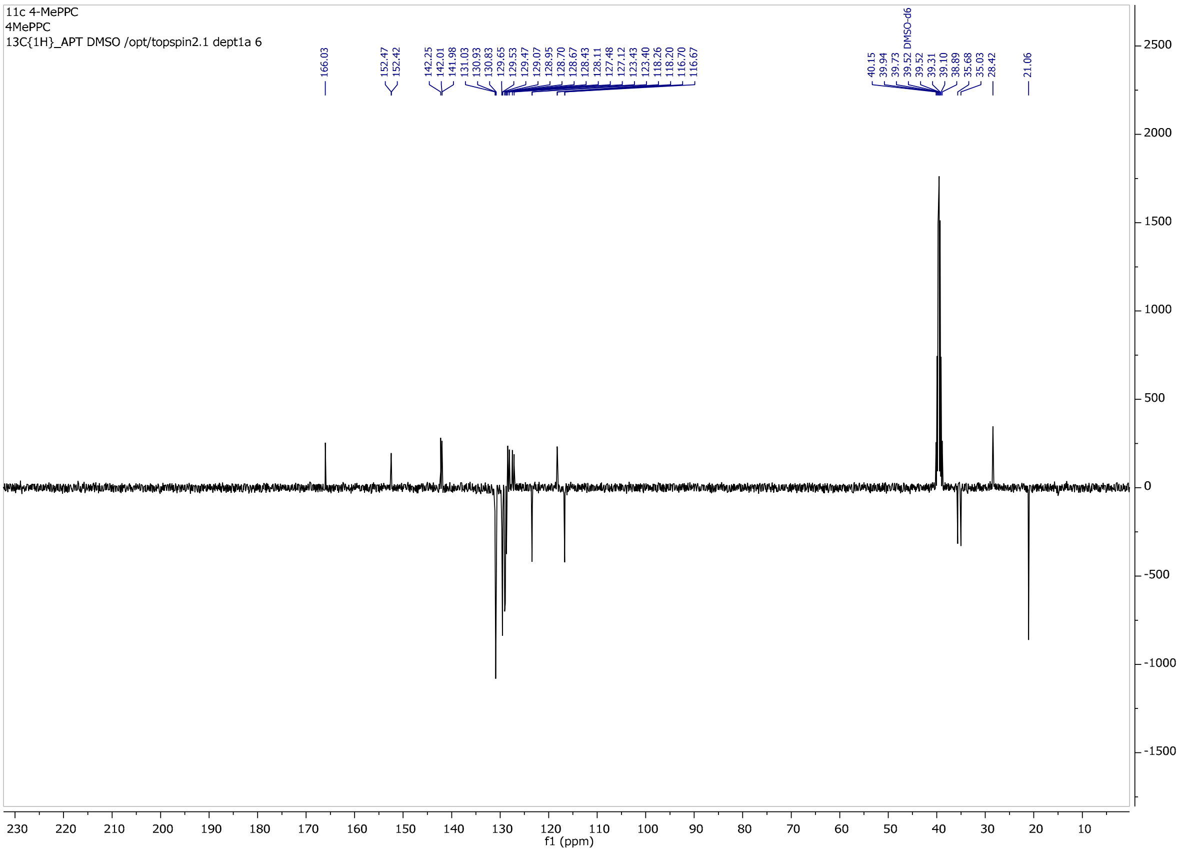

Mass Result

Analysis Info

Sample Name **MMN-3 c _ AR 11 c**

Acquisition Date 6/29/2023 4:49:06 PM

Instrument / Ser# micrOTOF-Q 228888.10300

Acquisition Parameter

Source Type	ESI	Ion Polarity	Positive	Scan Begin	50 m/z	Scan End	3000 m/z

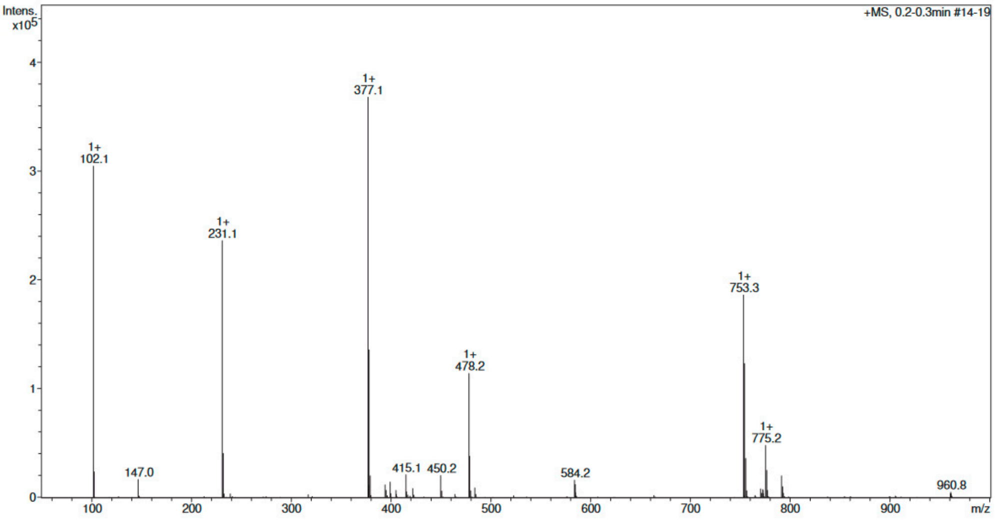

High Resolution Mass Result

Analysis Info

Sample Name **MMN-3 c _ AR 11 c**

Acquisition Date 6/29/2023 4:49:06 PM

Instrument / Ser# micrOTOF-Q 228888.10300

Acquisition Parameter

Source Type ESI   Ion Polarity Positive   Scan Begin 50 m/z   Scan End 3000 m/z

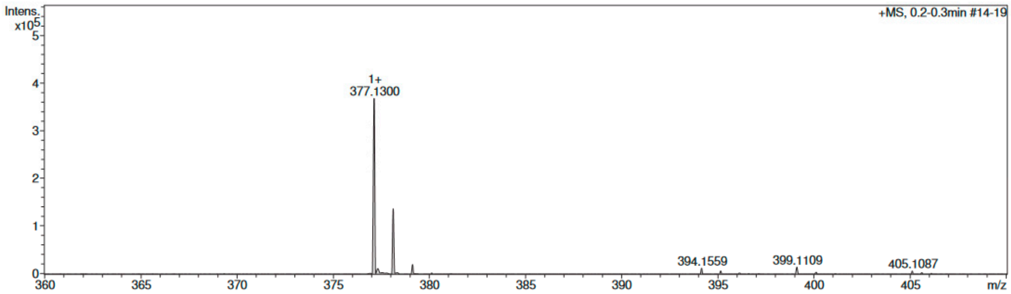

Meas. m/z	#	Ion Formula	Score	m/z	err [mDa]	err [ppm]	mSigma	rdb	e⁻ Conf	N-Rule	Adduct
377.1300	1	C23H22O3P	100.00	377.1301	-0.1	-0.2	59.5	13.5	even	ok	M+H

4-(bis(2-methoxyphenyl)phosphoryl)chroman-2-one COUM-2oOMe

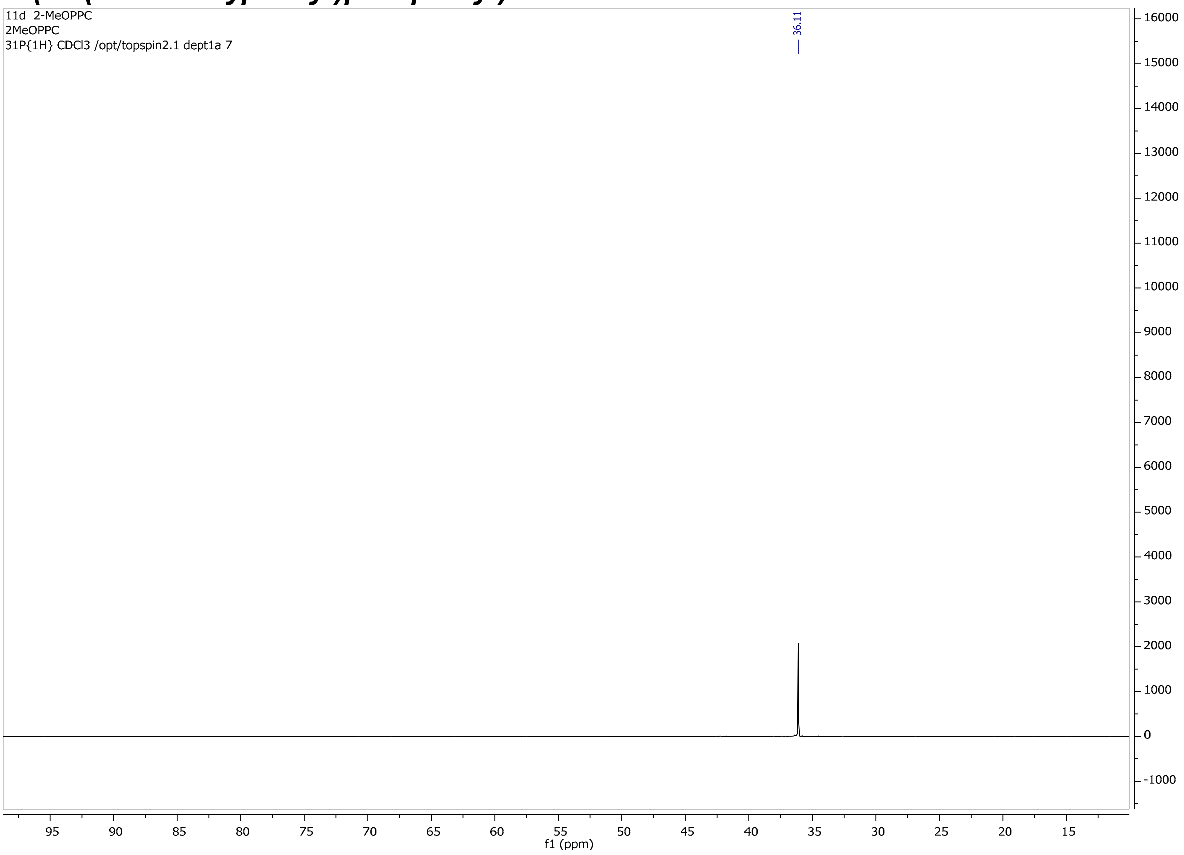

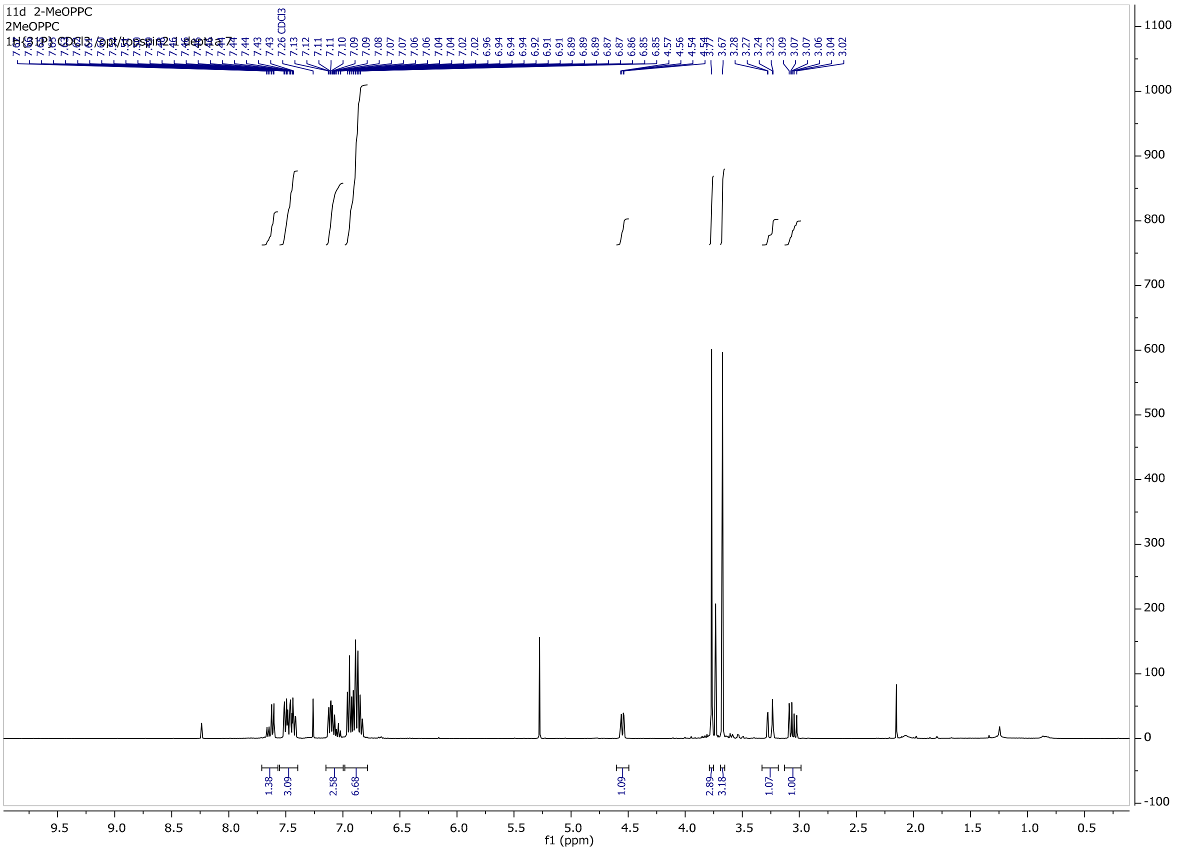

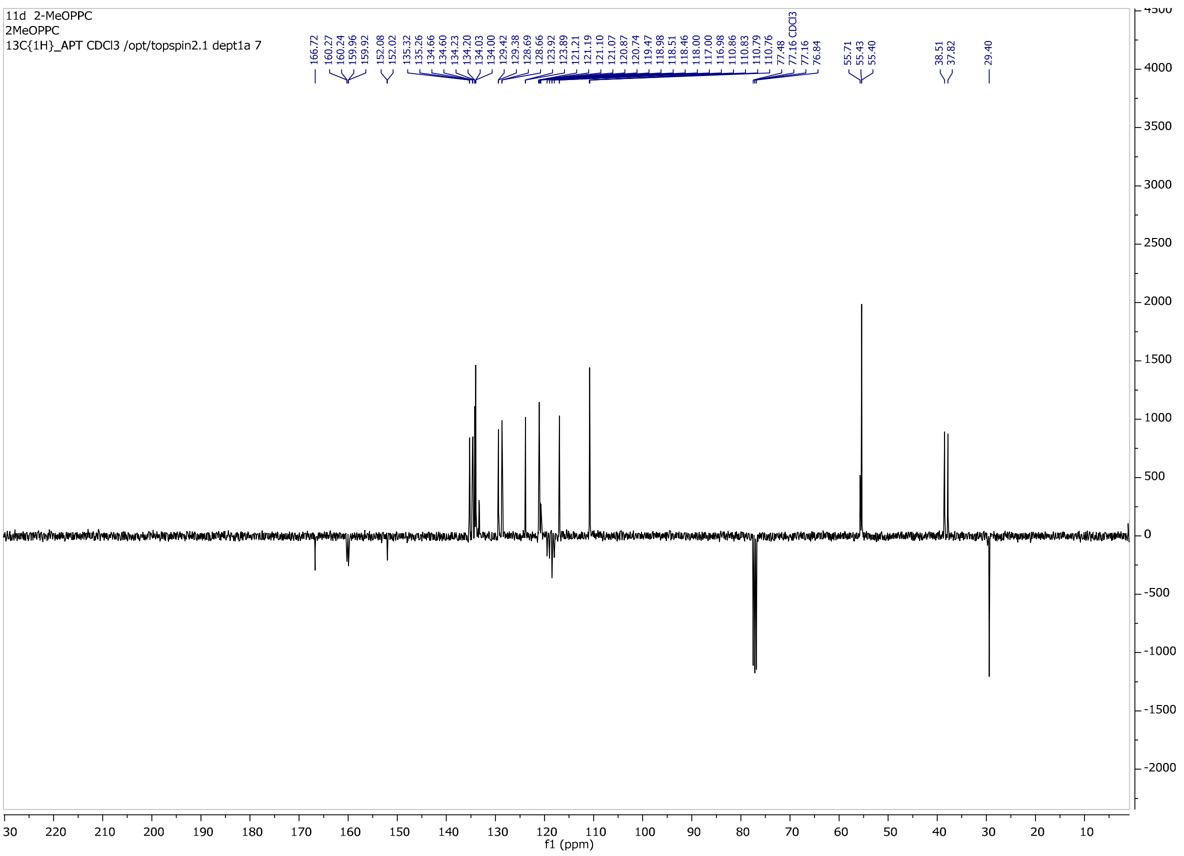

Mass Result

Analysis Info

Sample Name **MMN-3 d _ AR 11 d**

Acquisition Date 6/29/2023 4:52:08 PM

Instrument / Ser# micrOTOF-Q 228888.10300

Acquisition Parameter

Source Type ESI | Ion Polarity Positive | Scan Begin 50 m/z | Scan End 3000 m/z

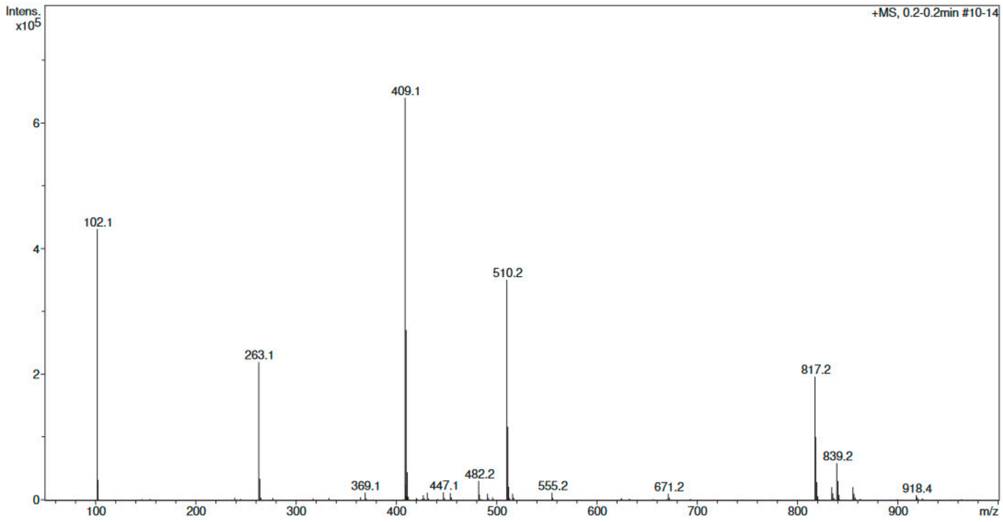

High Resolution Mass Result

Analysis Info

Sample Name **MMN-3 d _ AR 11 d**

Acquisition Date 6/29/2023 4:52:08 PM

Instrument / Ser# micrOTOF-Q 228888.10300

Acquisition Parameter

Source Type ESI | Ion Polarity Positive | Scan Begin 50 m/z | Scan End 3000 m/z

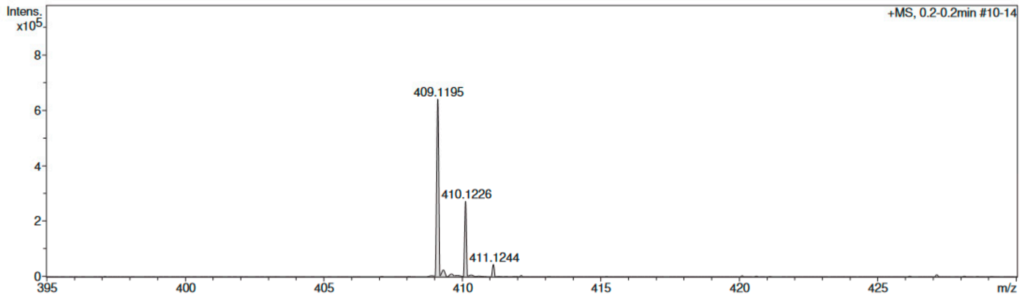

Meas. m/z	#	Ion Formula	Score	m/z	err [mDa]	err [ppm]	mSigma	rdb	e^- Conf	N-Rule	Adduct
409.1195	1	C23H22O5P	100.00	409.1199	0.4	1.0	86.5	13.5	even	ok	M+H

4-(bis(3-methoxyphenyl)phosphoryl)chroman-2-one COUM-2mOMe

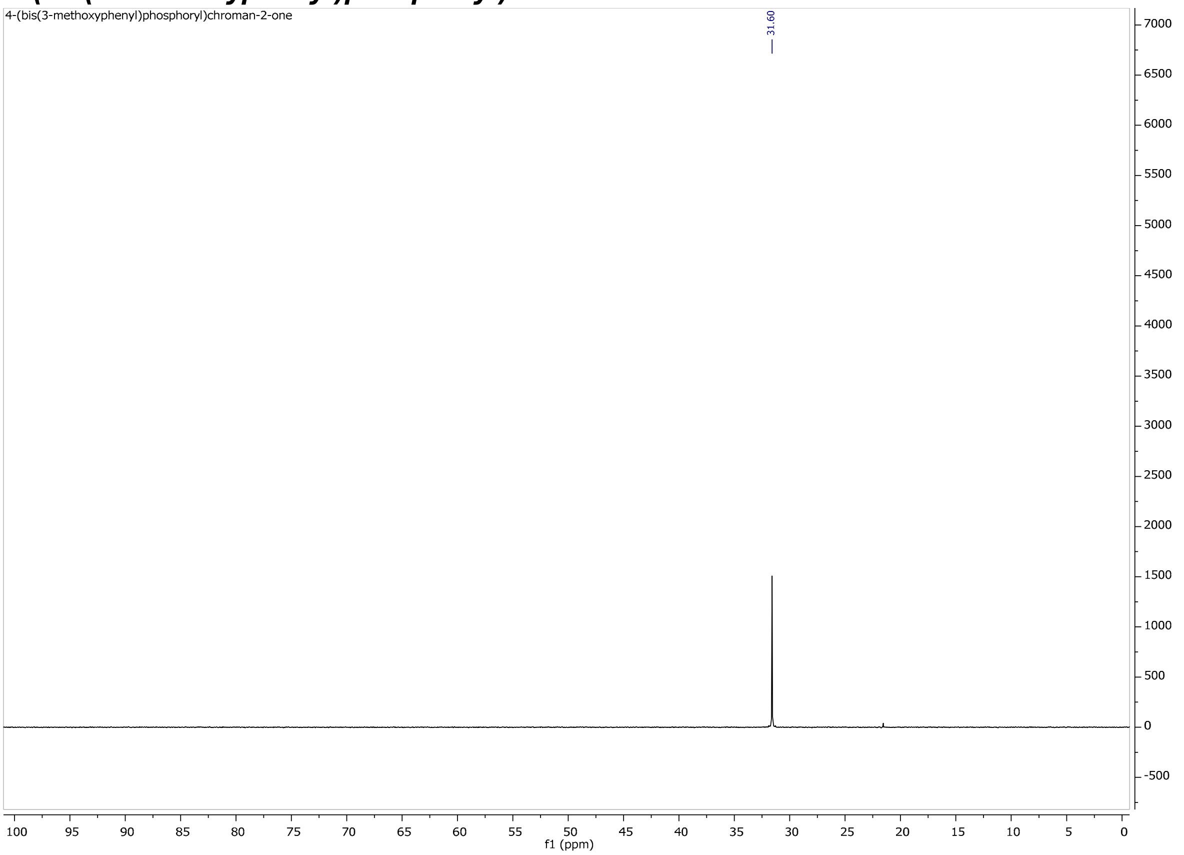

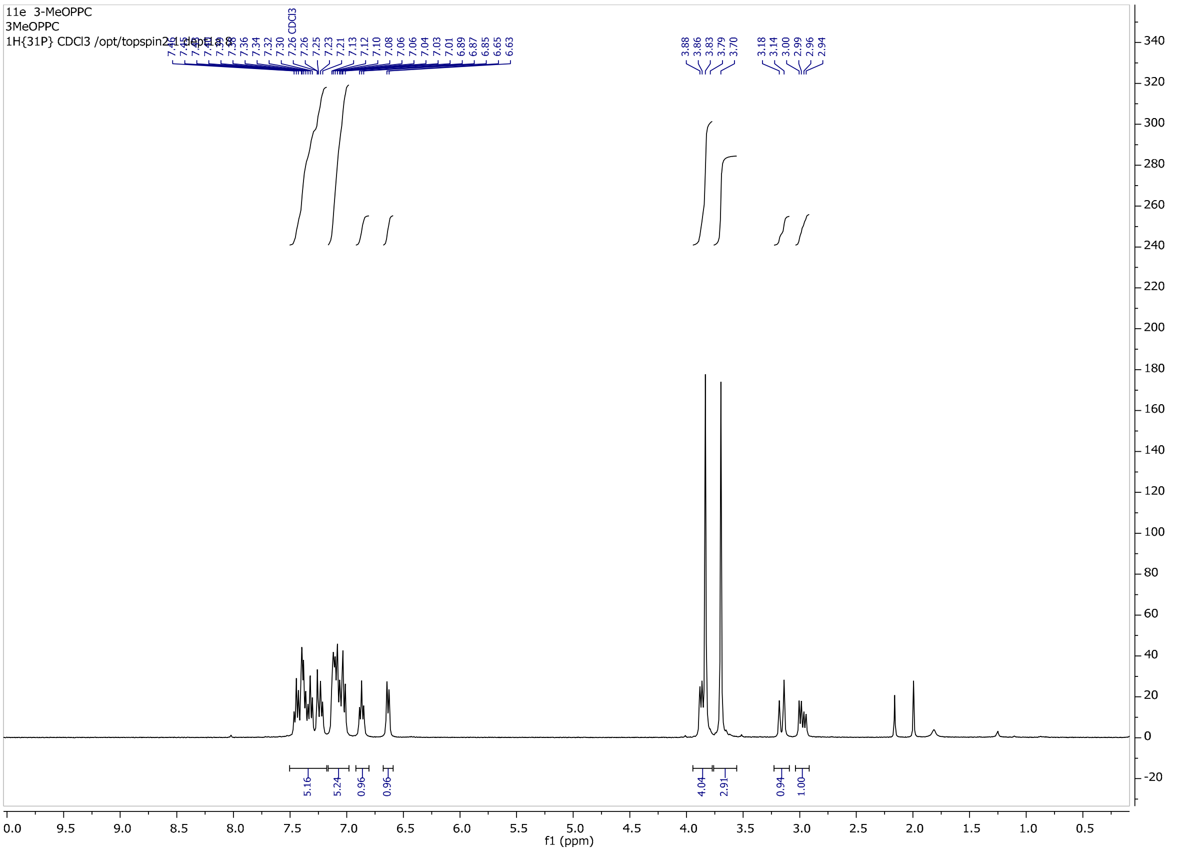

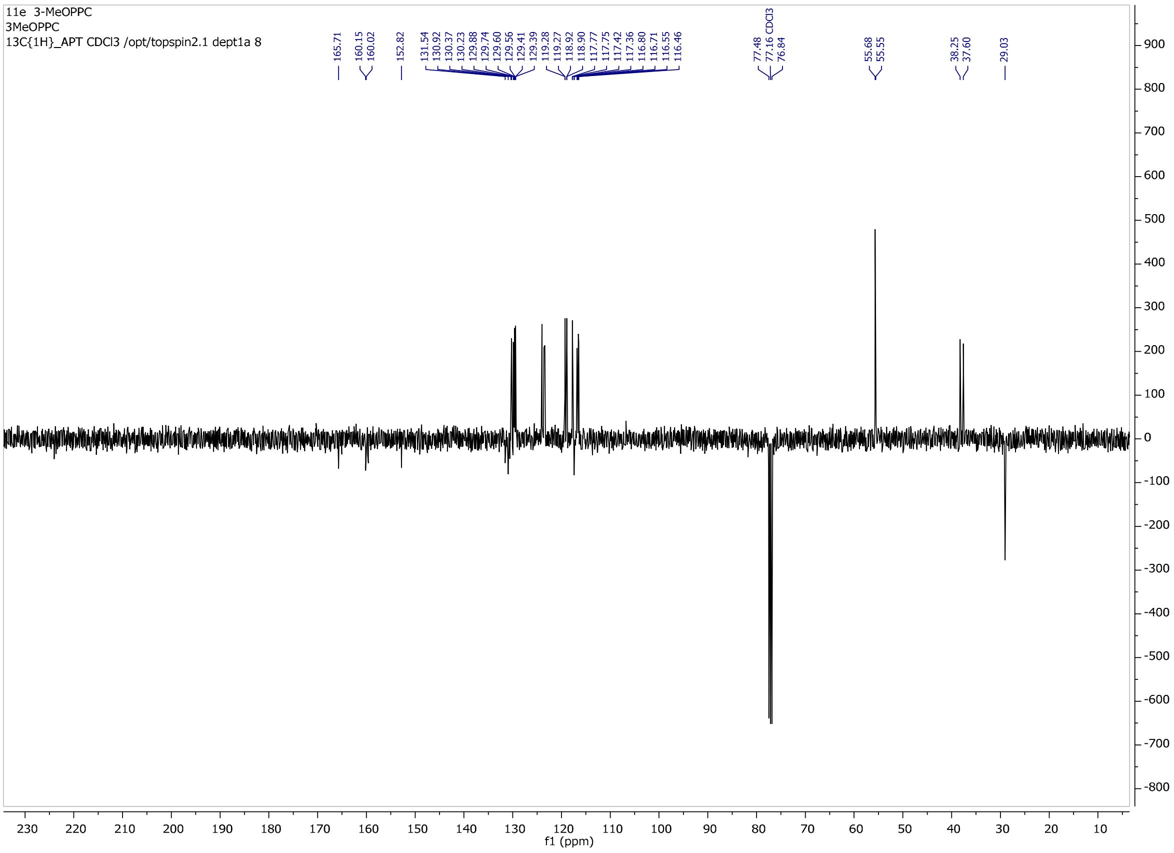

Mass Result

**Analysis Info**		Acquisition Date	6/29/2023 4:55:12 PM	
Sample Name	**MMN-3 e _ AR 11 e**	Instrument / Ser#	micrOTOF-Q	228888.10300

Acquisition Parameter

Source Type	ESI	Ion Polarity	Positive	Scan Begin	50 m/z	Scan End	3000 m/z

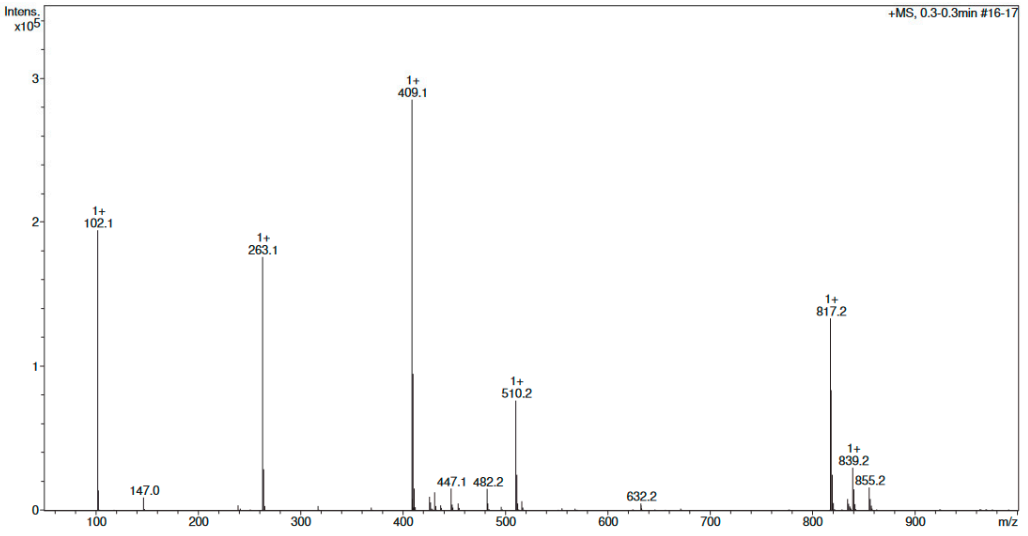

High Resolution Mass Result

Analysis Info

Sample Name **MMN-3 e _ AR 11 e**

Acquisition Date 6/29/2023 4:55:12 PM

Instrument / Ser# micrOTOF-Q 228888.10300

Acquisition Parameter

Source Type ESI | Ion Polarity Positive | Scan Begin 50 m/z | Scan End 3000 m/z

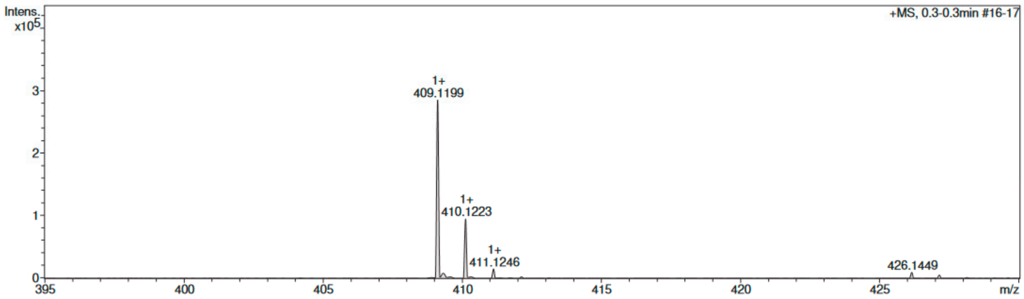

Meas. m/z	#	Ion Formula	Score	m/z	err [mDa]	err [ppm]	mSigma	rdb	e⁻ Conf	N-Rule	Adduct
409.119890	1	C24H18N4OP	66.46	409.121274	1.4	3.4	28.7	18.5	even	ok	M+H
	2	C23H22O5P	100.00	409.119937	0.0	0.1	40.0	13.5	even	ok	M+H

4-(bis(4-methoxyphenyl)phosphoryl)chroman-2-one COUM-2pOMe

RMN 244
KM 244 PUR 1 2 EM COL
31P{1H} CDCl3 /opt/topspin2.1 dept1a 21

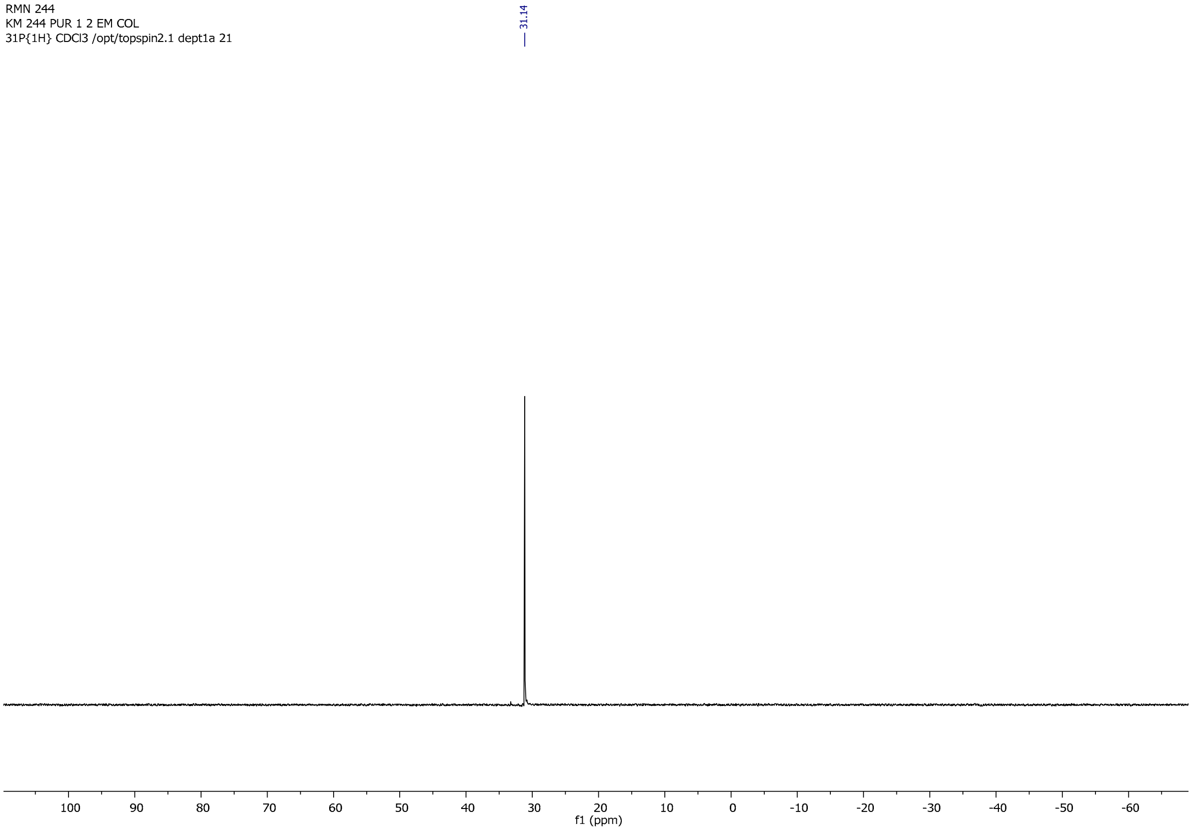

RMN 244 para OMe
KM 244 PUR 1 B ROUGE
1H{31P} CDCl3 /opt/topspin2.1 dept1a 12

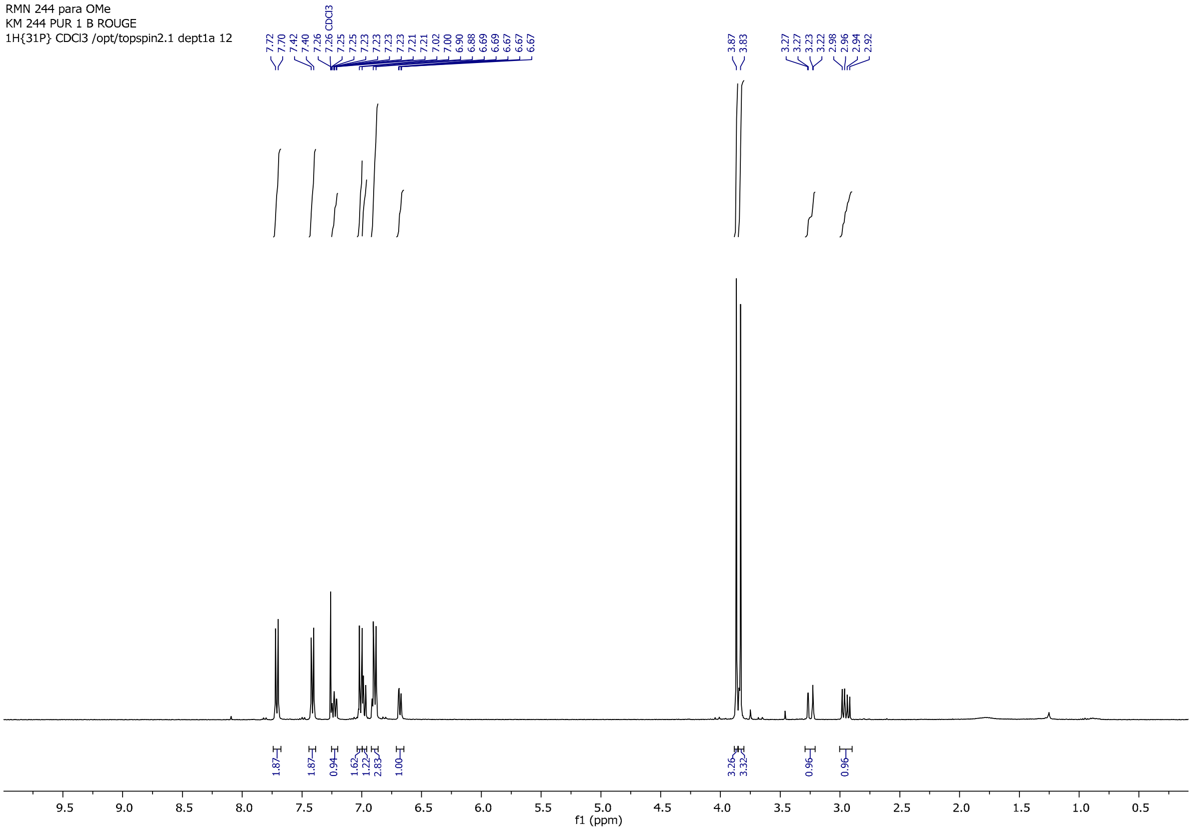

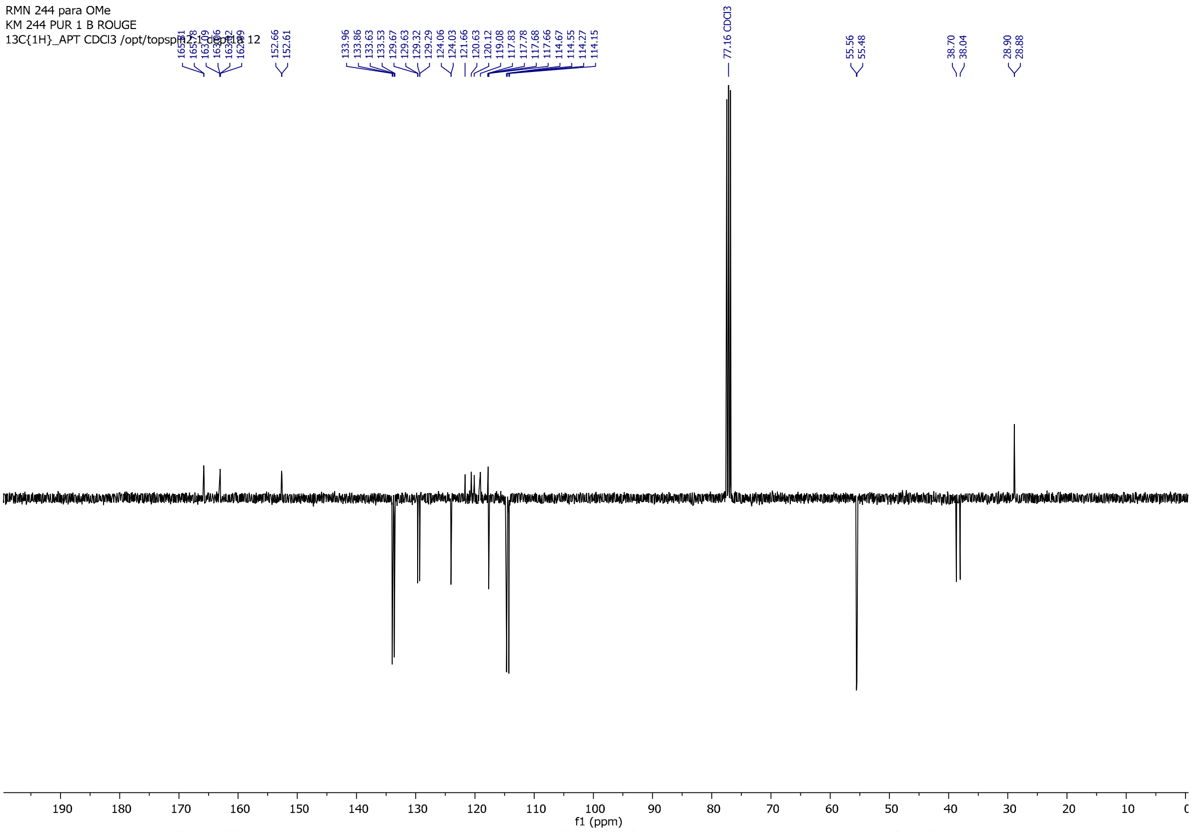

RMN 244 para OMe
KM 244 PUR 1 B ROUGE
13C{1H}_APT CDCl3 /opt/topspin2.1 dept1a 12
165.81
165.78
163.09
163.06
163.02
162.99
152.66
152.61
133.96
133.86
133.63
133.53
129.67
129.63
129.32
129.29
124.06
124.03
121.66
120.63
120.12
119.08
117.83
117.78
117.68
117.66
114.67
114.55
114.27
114.15
77.16 CDCl3
55.56
55.48
38.70
38.04
28.90
28.88
190 180 170 160 150 140 130 120 110 100 90 80 70 60 50 40 30 20 10
f1 (ppm)

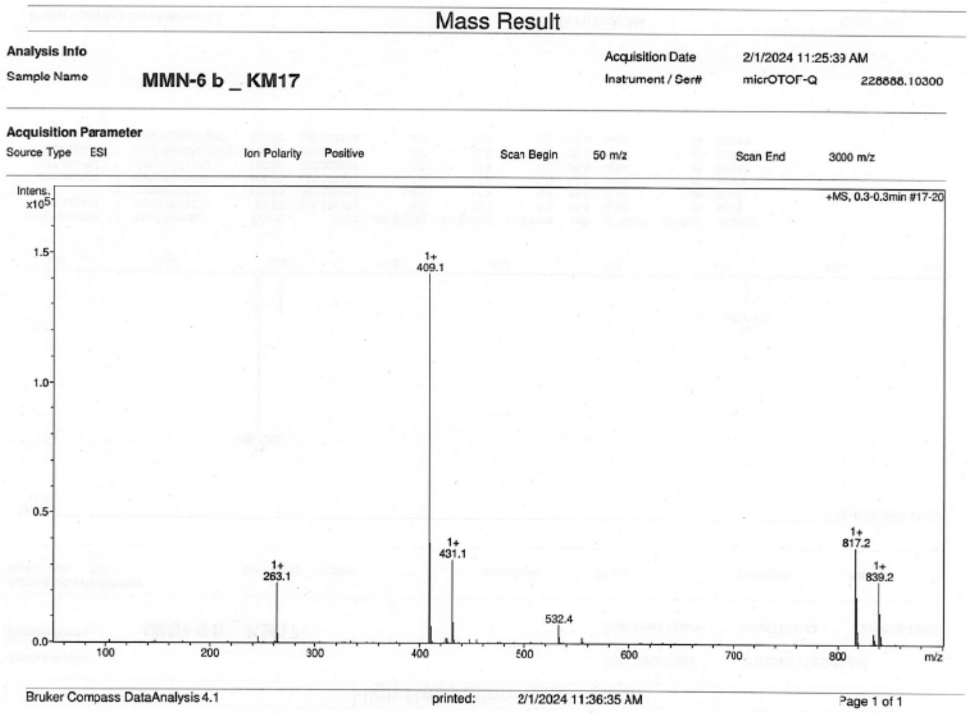

Mass Result
Analysis Info
Sample Name
MMN-6 b _ KM17
Acquisition Date
2/1/2024 11:25:39 AM
Instrument / Ser#
micrOTOF-Q
228888.10300
Acquisition Parameter
Source Type
ESI
Ion Polarity
Positive
Scan Begin
50 m/z
Scan End
3000 m/z
Intens.
x10^5
+MS, 0.3-0.3min #17-20
1.5
1.0
0.5
0.0
1+ 409.1
1+ 263.1
1+ 431.1
532.4
1+ 817.2
1+ 839.2
100 200 300 400 500 600 700 800
m/z
Bruker Compass DataAnalysis 4.1
printed:
2/1/2024 11:36:35 AM
Page 1 of 1

High Resolution Mass Result

Analysis Info

Sample Name **MMN-6 b _ KM17**

Acquisition Date 2/1/2024 11:25:39 AM

Instrument / Ser# micrOTOF-Q 228888.10300

Acquisition Parameter

Source Type ESI | Ion Polarity Positive | Scan Begin 50 m/z | Scan End 3000 m/z

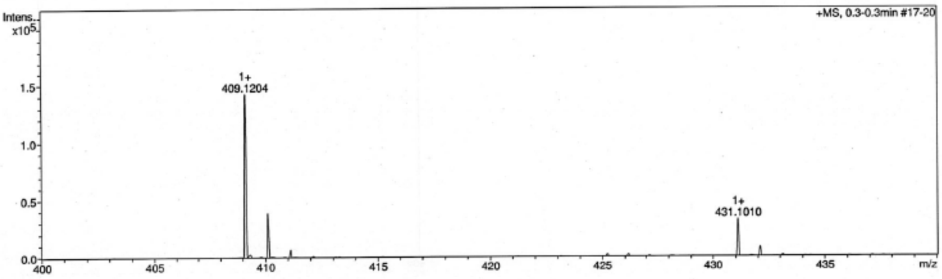

Meas. m/z	#	Ion Formula	Score	m/z	err [mDa]	err [ppm]	mSigma	rdb	e⁻ Conf	N-Rule	Adduct
409.120414	1	$C_{24}H_{18}N_4OP$	91.37	409.121274	-0.9	-2.1	3.8	18.5	even	ok	M+H
	2	$C_{23}H_{22}O_5P$	100.00	409.119937	-0.5	-1.2	9.8	13.5	even	ok	M+H
431.100999	1	$C_{23}H_{21}NaO_5P$	100.00	431.101881	0.9	2.0	3.0	13.5	even	ok	M+Na
817.234194	1	$C_{46}H_{43}O_{10}P_2$	100.00	817.232597	-1.6	-2.0	4.9	26.5	even	ok	2M+H
839.216540	1	$C_{48}H_{34}N_8NaO_2P_2$	100.00	839.217216	-0.7	-0.8	24.8	36.5	even	ok	2M+Na
	1	$C_{46}H_{42}NaO_{10}P_2$	100.00	839.214542	-2.0	-2.4	3.6	26.5	even	ok	2M+Na

4-(bis(3-fluorophenyl)phosphoryl)chroman-2-one COUM-2mF

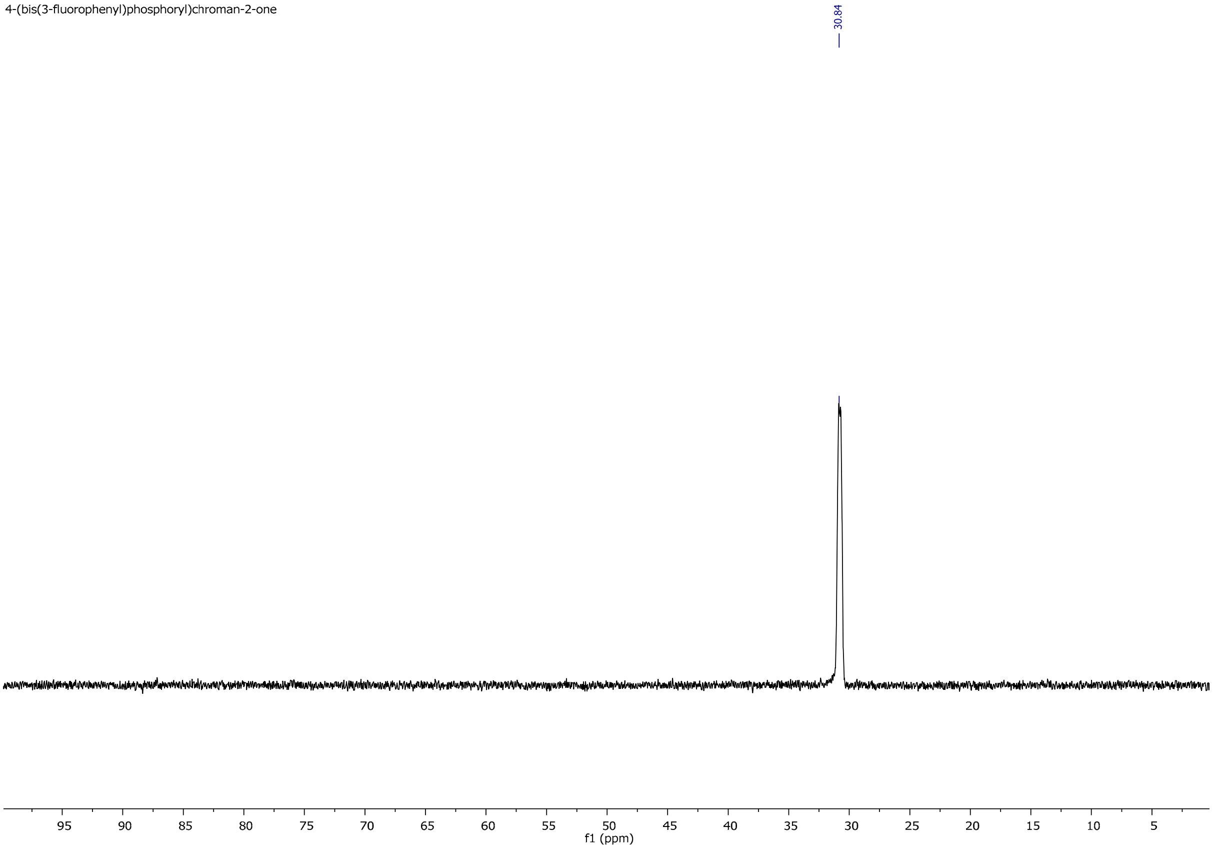

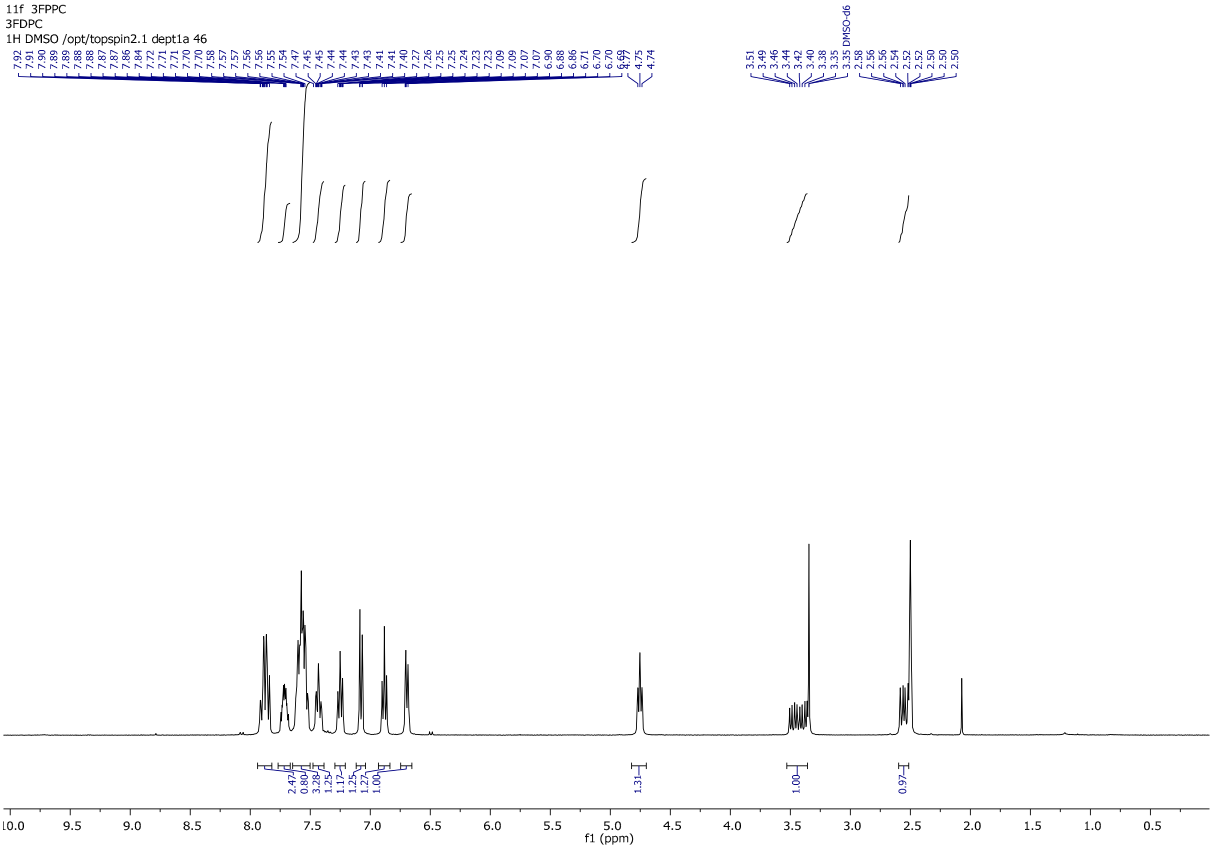

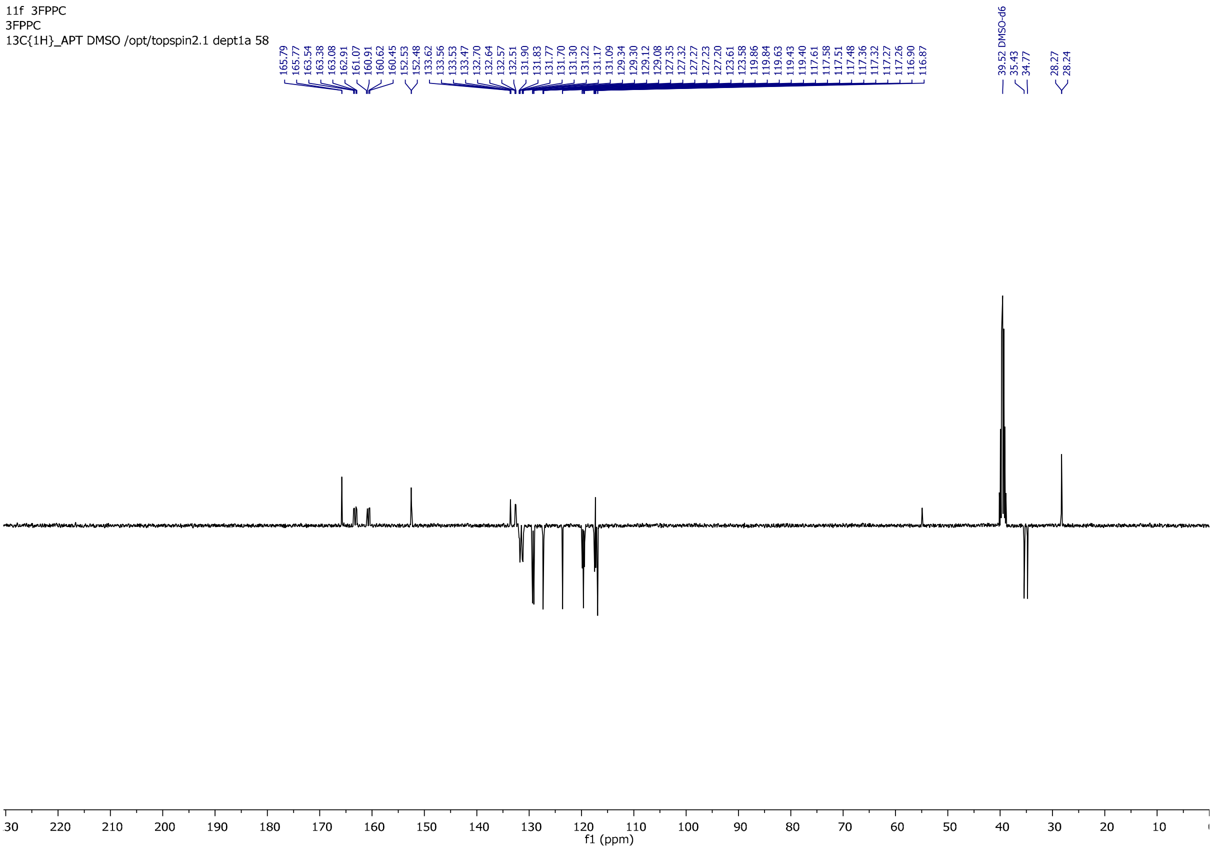

Mass Result

**Analysis Info**		Acquisition Date	6/29/2023 4:58:15 PM
Sample Name	**MMN-3 f _ AR 11 f**	Instrument / Ser#	micrOTOF-Q 228888.10300

Acquisition Parameter

Source Type	ESI	Ion Polarity	Positive	Scan Begin	50 m/z	Scan End	3000 m/z

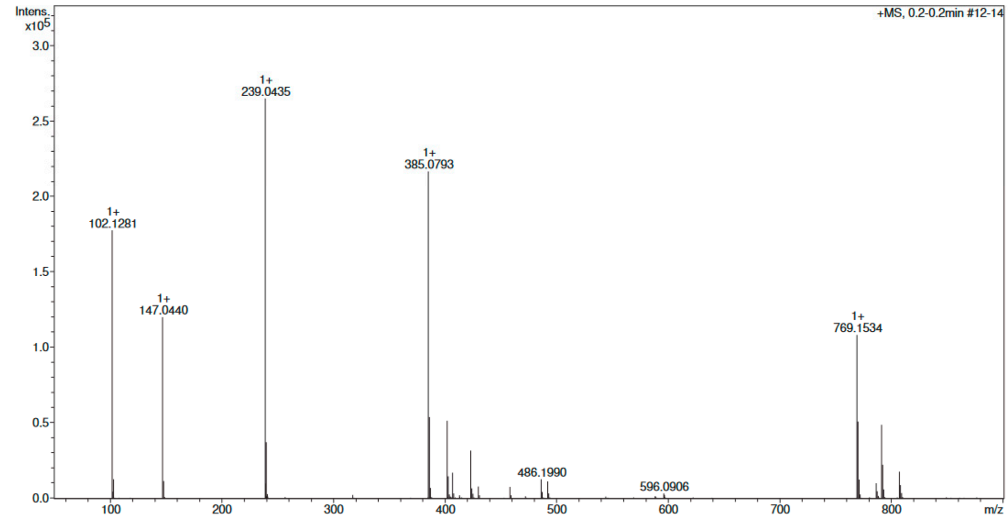

High Resolution Mass Result

Analysis Info

Sample Name	**MMN-3 f _ AR 11 f**	Acquisition Date	6/29/2023 4:58:15 PM
		Instrument / Ser#	micrOTOF-Q 228888.10300

Acquisition Parameter

Source Type	ESI	Ion Polarity	Positive	Scan Begin	50 m/z	Scan End	3000 m/z

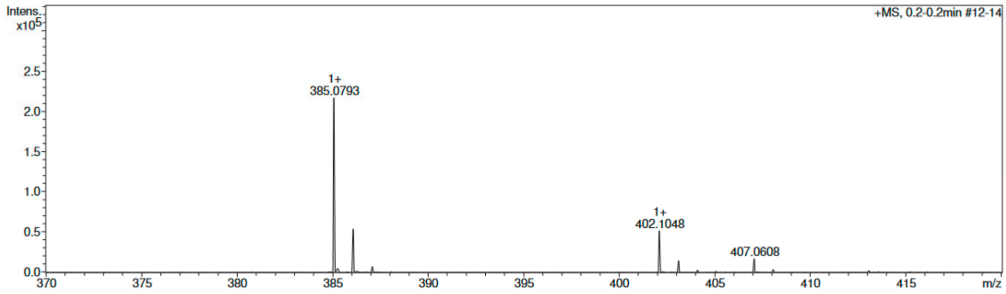

Meas. m/z	#	Ion Formula	Score	m/z	err [mDa]	err [ppm]	mSigma	rdb	e⁻ Conf	N-Rule	Adduct
385.0793	1	C21H16F2O3P	100.00	385.0800	0.6	1.6	10.3	13.5	even	ok	M+H
	2	C16H16F6O2P	46.00	385.0787	0.7	1.8	42.5	6.5	even	ok	M+H

4-(bis(4-fluorophenyl)phosphoryl)chroman-2-one COUM-2pF

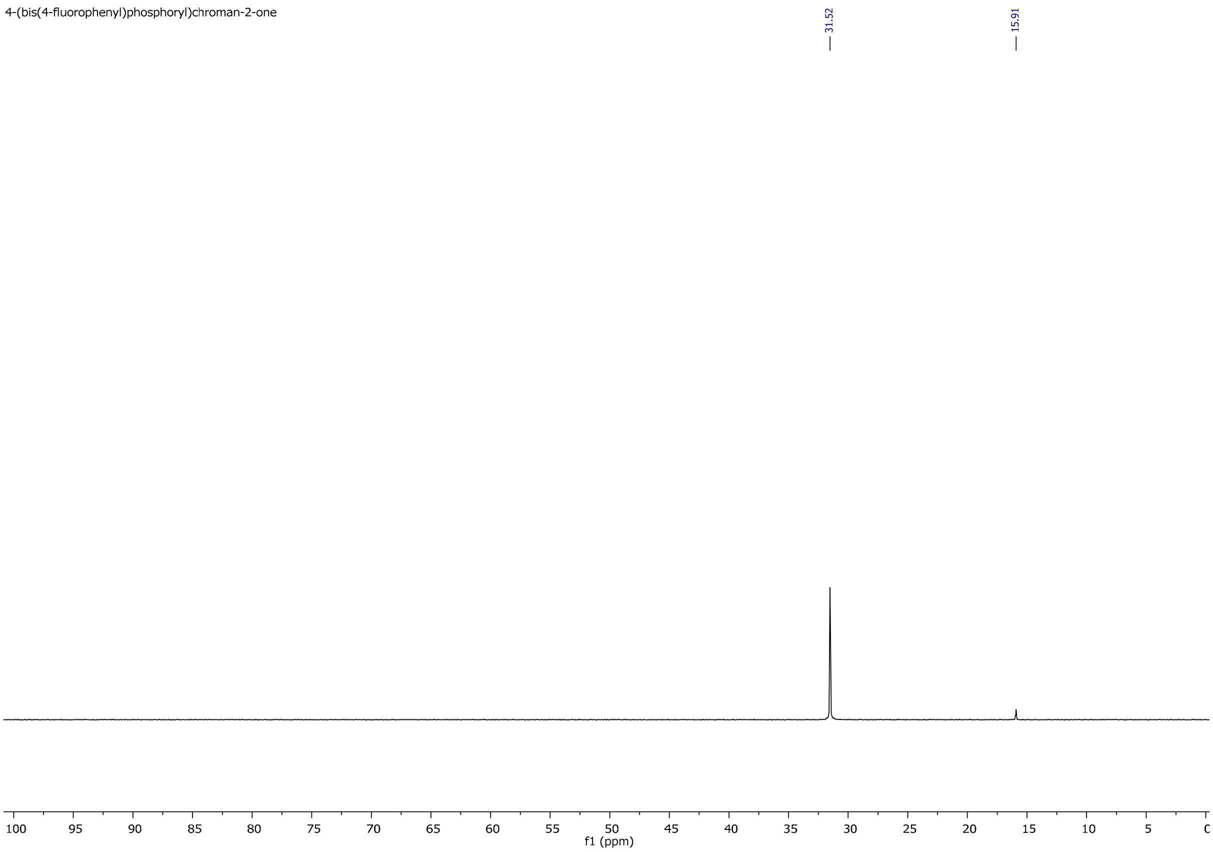

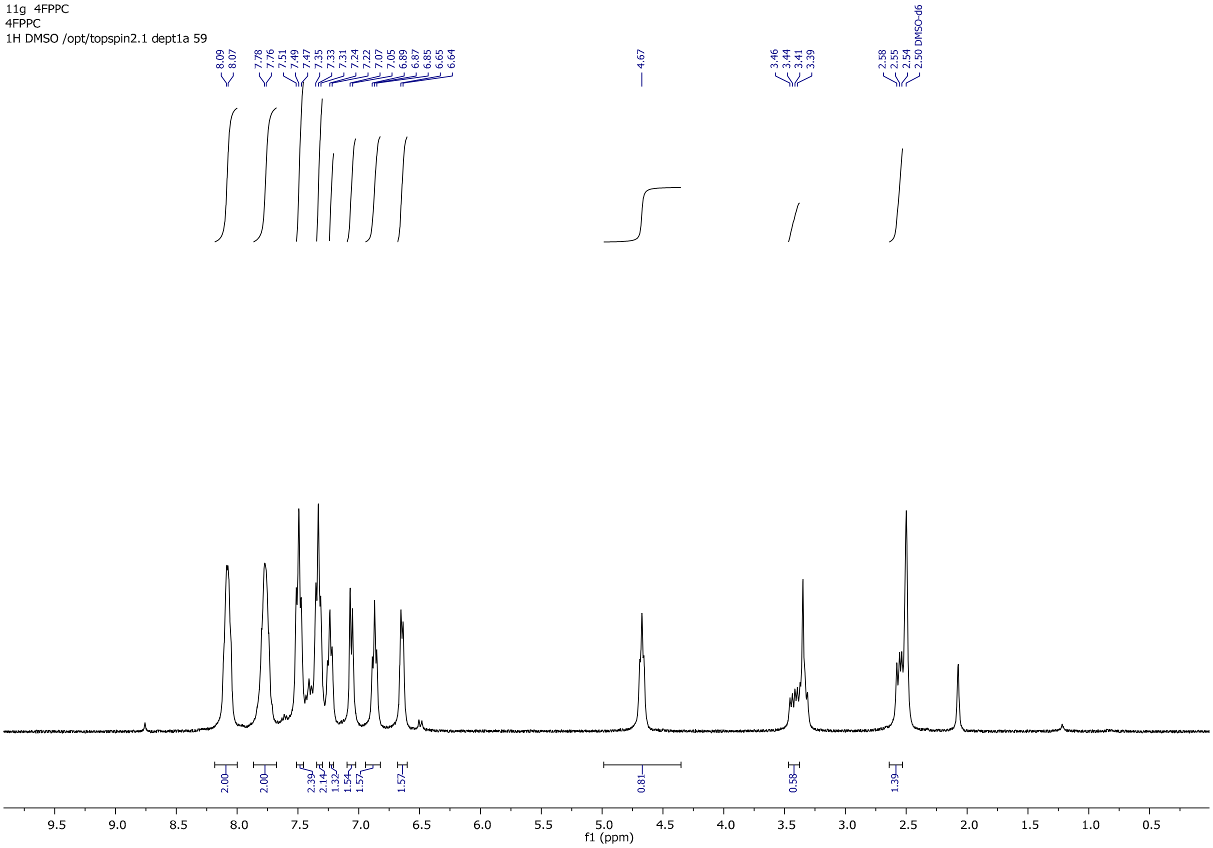

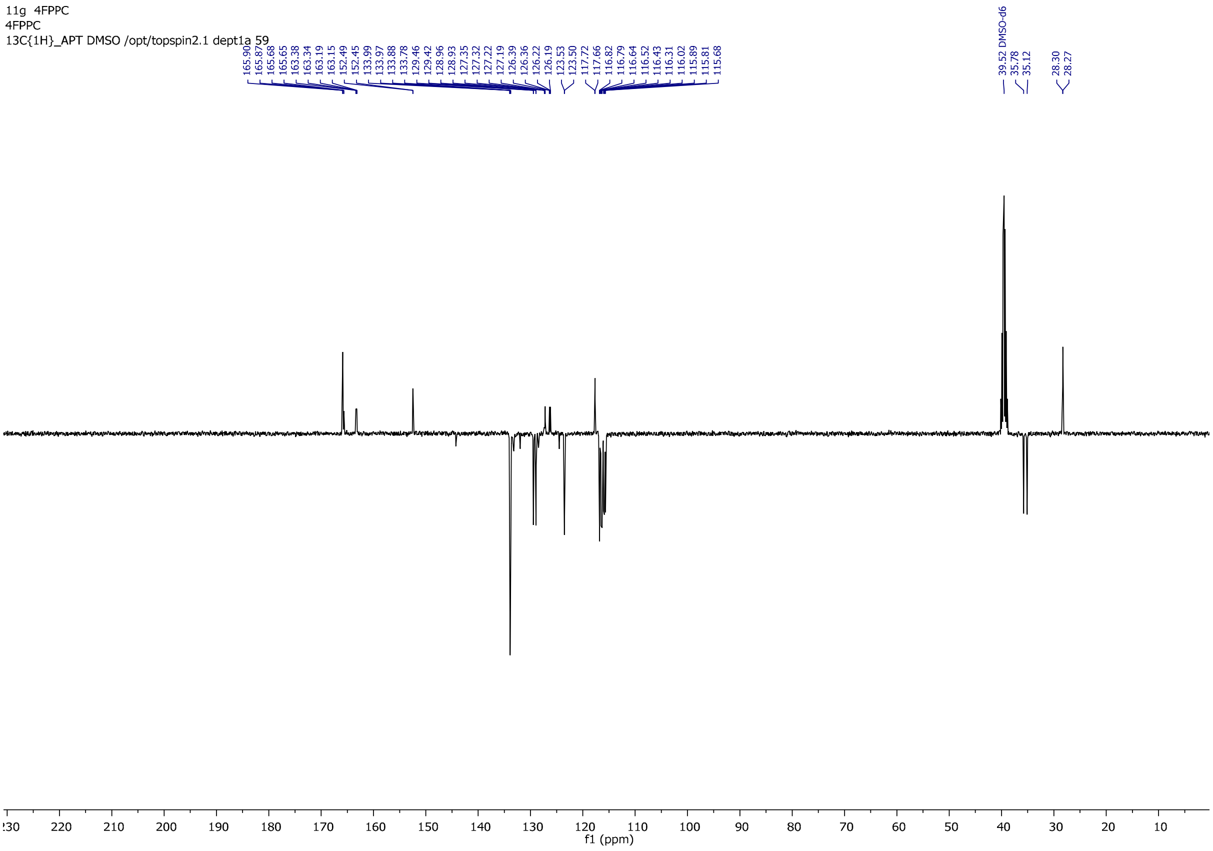

Mass Result

Analysis Info

Sample Name **MMN-3 g _ AR 11 g**

Acquisition Date 6/29/2023 5:01:17 PM

Instrument / Ser# micrOTOF-Q 228888.10300

Acquisition Parameter

Source Type ESI | Ion Polarity Positive | Scan Begin 50 m/z | Scan End 3000 m/z

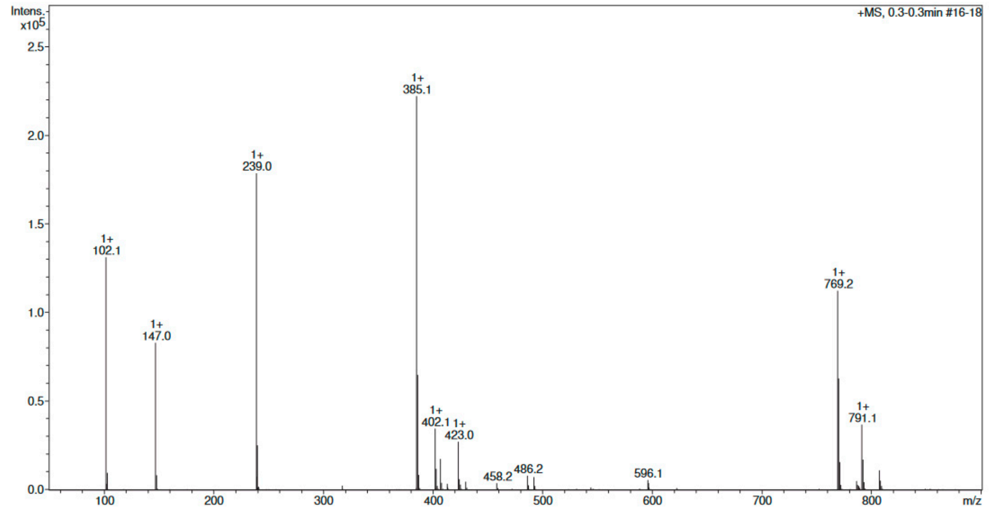

High Resolution Mass Result

Analysis Info

Sample Name	**MMN-3 g _ AR 11 g**	Acquisition Date	6/29/2023 5:01:17 PM
		Instrument / Ser#	micrOTOF-Q 228888.10300

Acquisition Parameter

Source Type	ESI	Ion Polarity	Positive	Scan Begin	50 m/z	Scan End	3000 m/z

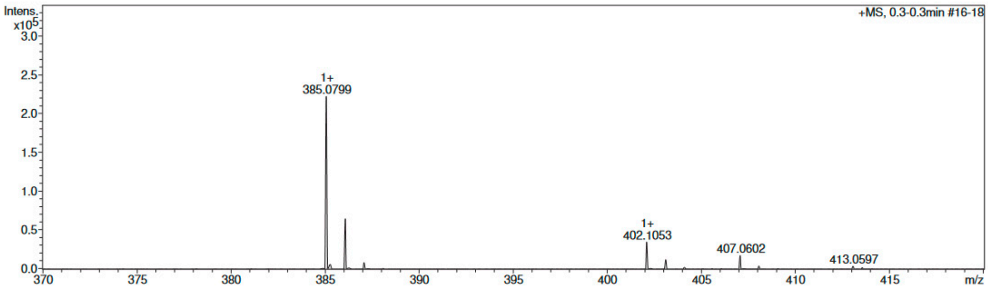

Meas. m/z	#	Ion Formula	Score	m/z	err [mDa]	err [ppm]	mSigma	rdb	e⁻ Conf	N-Rule	Adduct
385.0799	1	C21H16F2O3P	100.00	385.0800	0.1	0.2	36.0	13.5	even	ok	M+H
	2	C18H17F3O4P	32.50	385.0811	-1.2	-3.2	54.6	9.5	even	ok	M+H

4-(bis(3-chlorophenyl)phosphoryl)chroman-2-one COUM-2mCl

RMN
KM 245 CO
31P{1H} DMSO /opt/topspin2.1 dept1a 13

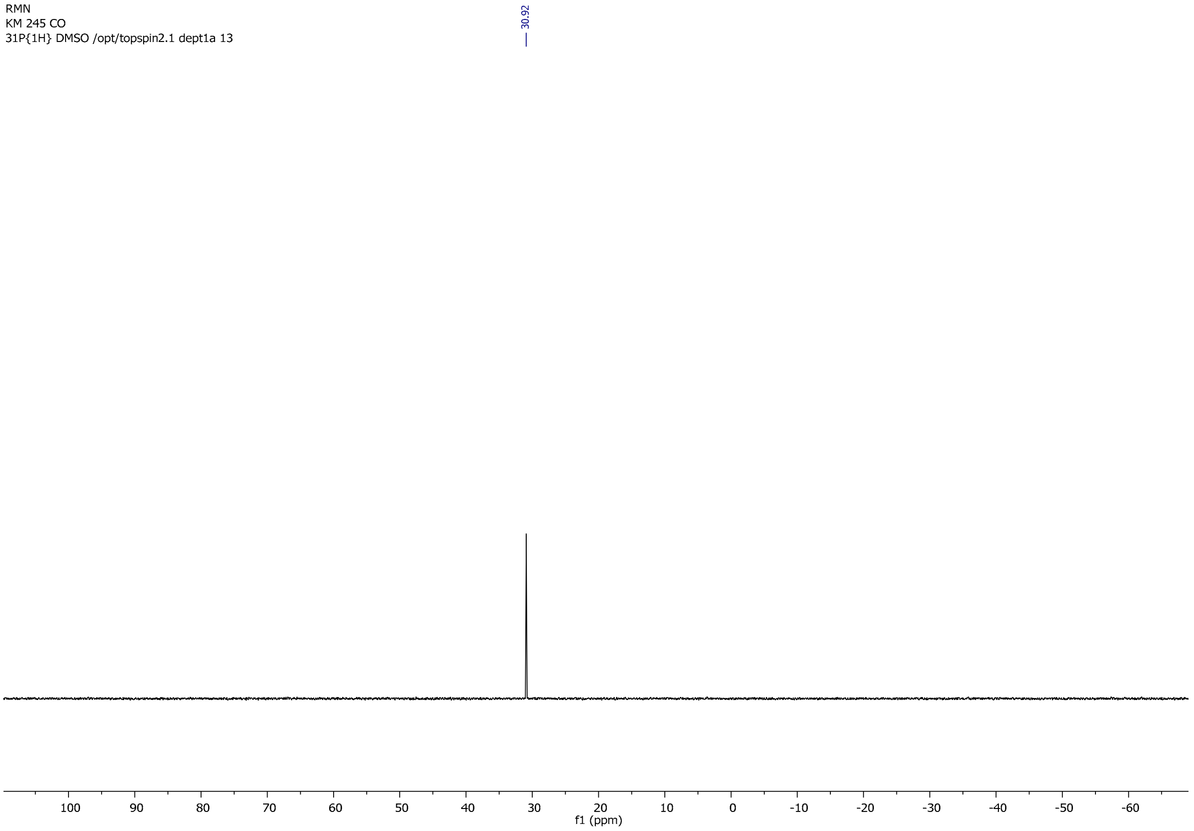

RMN
KM 245 CO
1H DMSO /opt/topspin2.1 dept1a 13

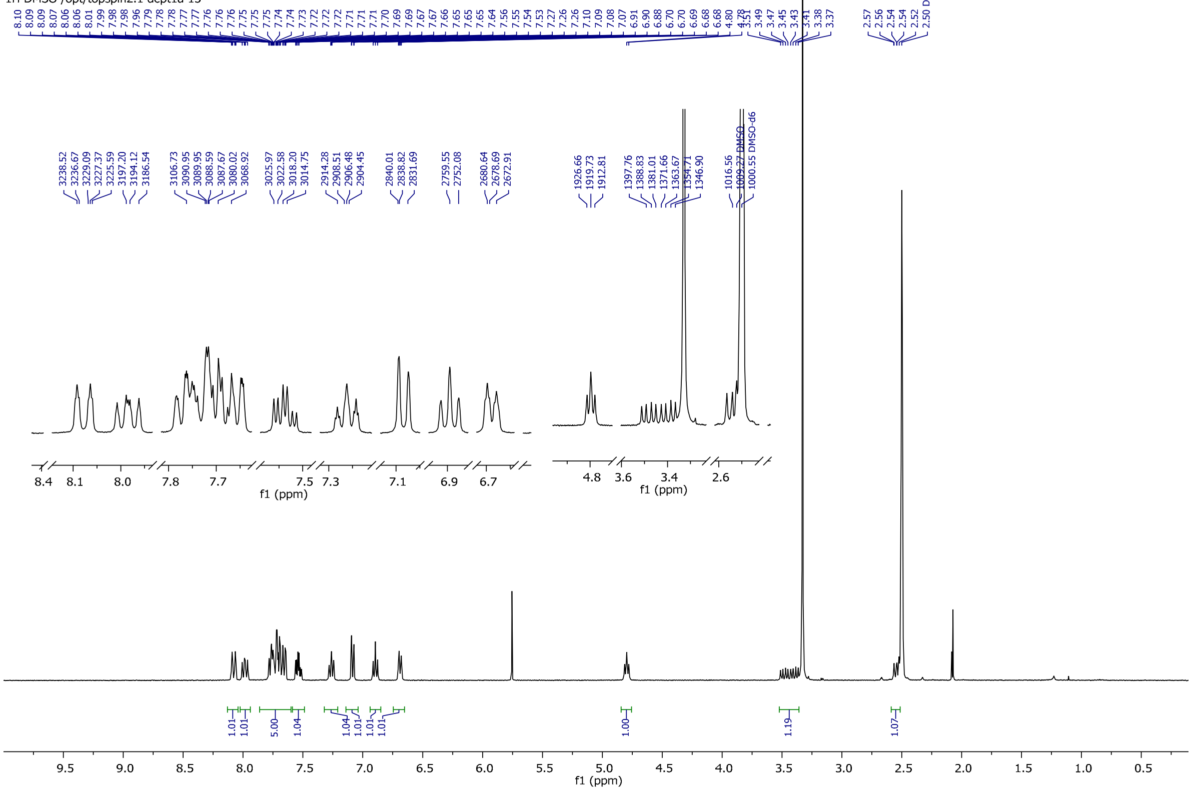

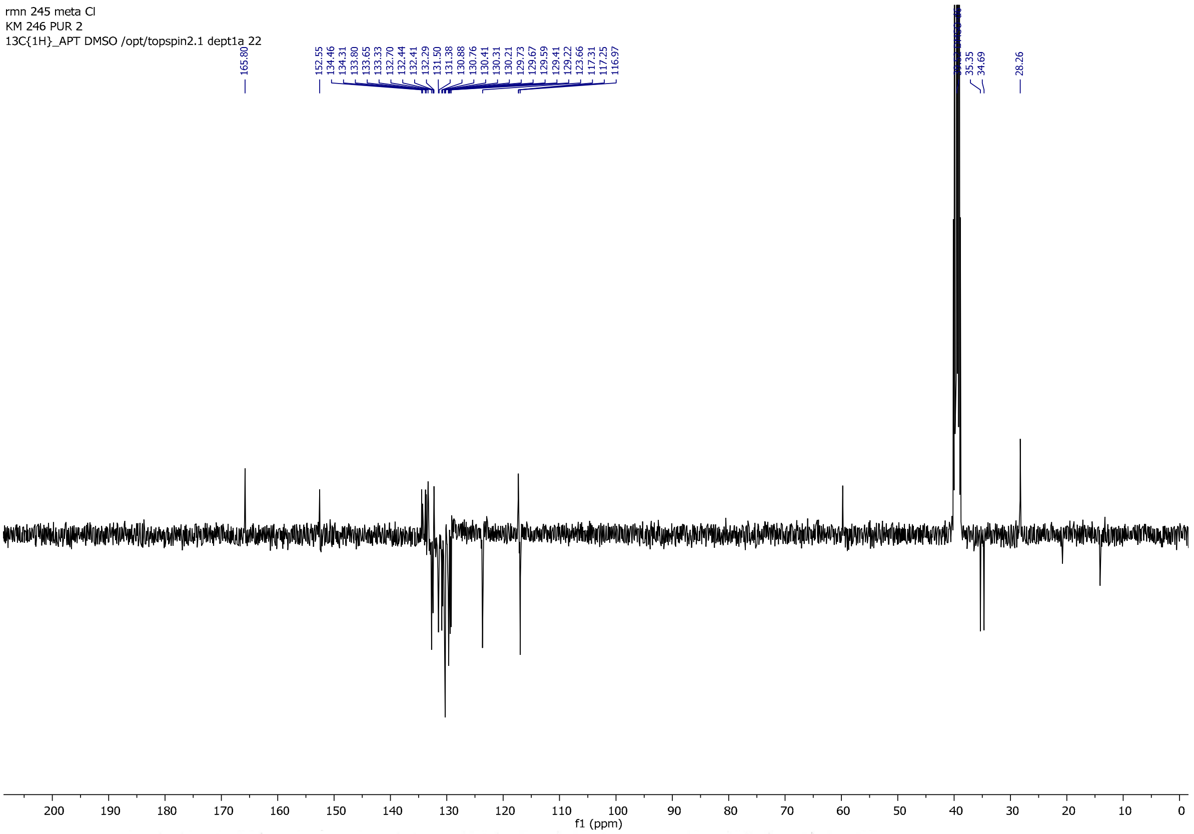

Mass Result

**Analysis Info**		Acquisition Date	2/1/2024 11:28:41 AM
Sample Name	**MMN-6 c _ KM18**	Instrument / Ser#	micrOTOF-Q 228888.10300

Acquisition Parameter

Source Type	ESI	Ion Polarity	Positive	Scan Begin	50 m/z	Scan End	3000 m/z

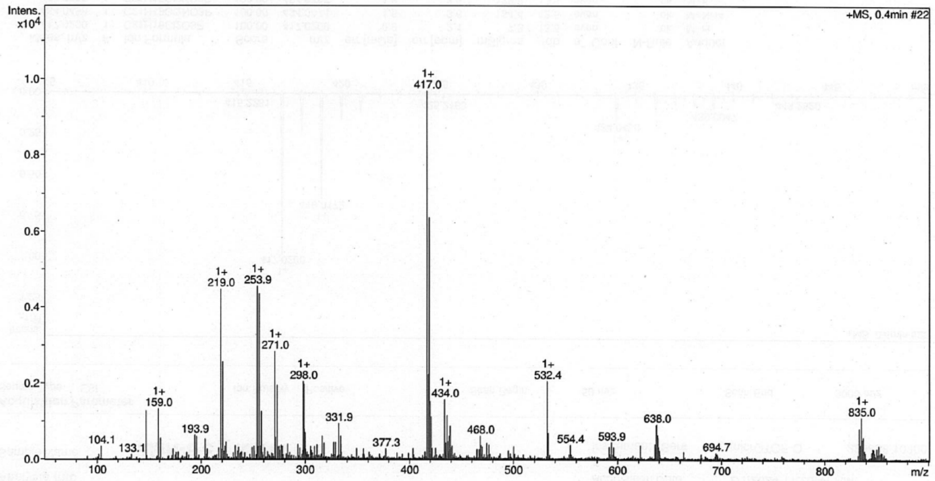

High Resolution Mass Result

Analysis Info

Sample Name **MMN-6 c _ KM18**

Acquisition Date 2/1/2024 11:28:41 AM

Instrument / Ser# micrOTOF-Q 228888.10300

Acquisition Parameter

Source Type ESI Ion Polarity Positive Scan Begin 50 m/z Scan End 3000 m/z

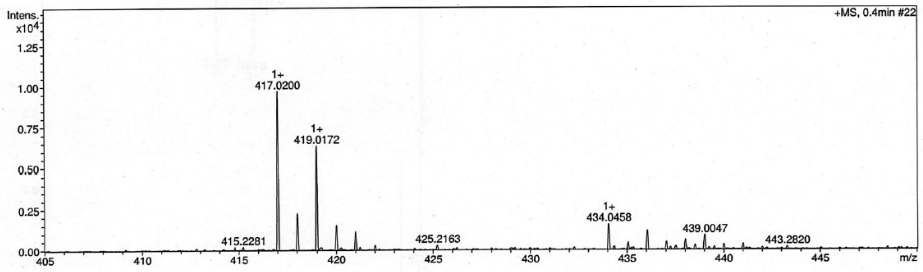

Meas. m/z	#	Ion Formula	Score	m/z	err [mDa]	err [ppm]	mSigma	rdb	e⁻ Conf	N-Rule	Adduct
417.0200	1	C21H16Cl2O3P	100.00	417.0209	0.9	2.1	7.3	13.5	even	ok	M+H
434.0458	1	C21H19Cl2NO3P	100.00	434.0474	1.6	3.6	134.4	12.5	even	ok	M+NH4
454.9752	1	C21H15Cl2KO3P	100.00	454.9767	1.6	3.5	128.8	13.5	even	ok	M+K
833.0350	1	C42H31Cl4O6P2	100.00	833.0344	0.5	0.6	23.1	26.5	even	ok	2M+H
850.0583	1	C42H34Cl4NO6P2	100.00	850.0610	-2.7	-3.2	176.7	25.5	even	ok	2M+NH4

4-(bis(4-chlorophenyl)phosphoryl)chroman-2-one COUM-2pCl

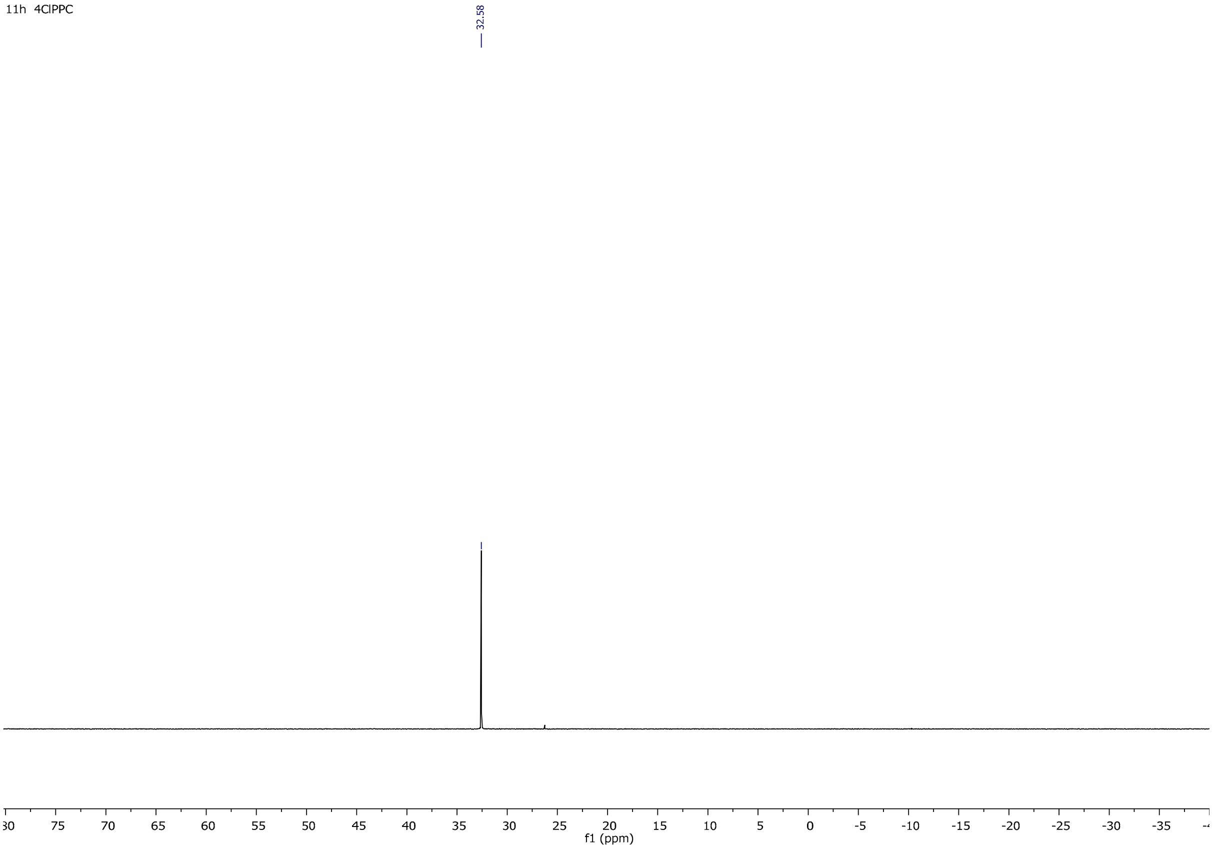

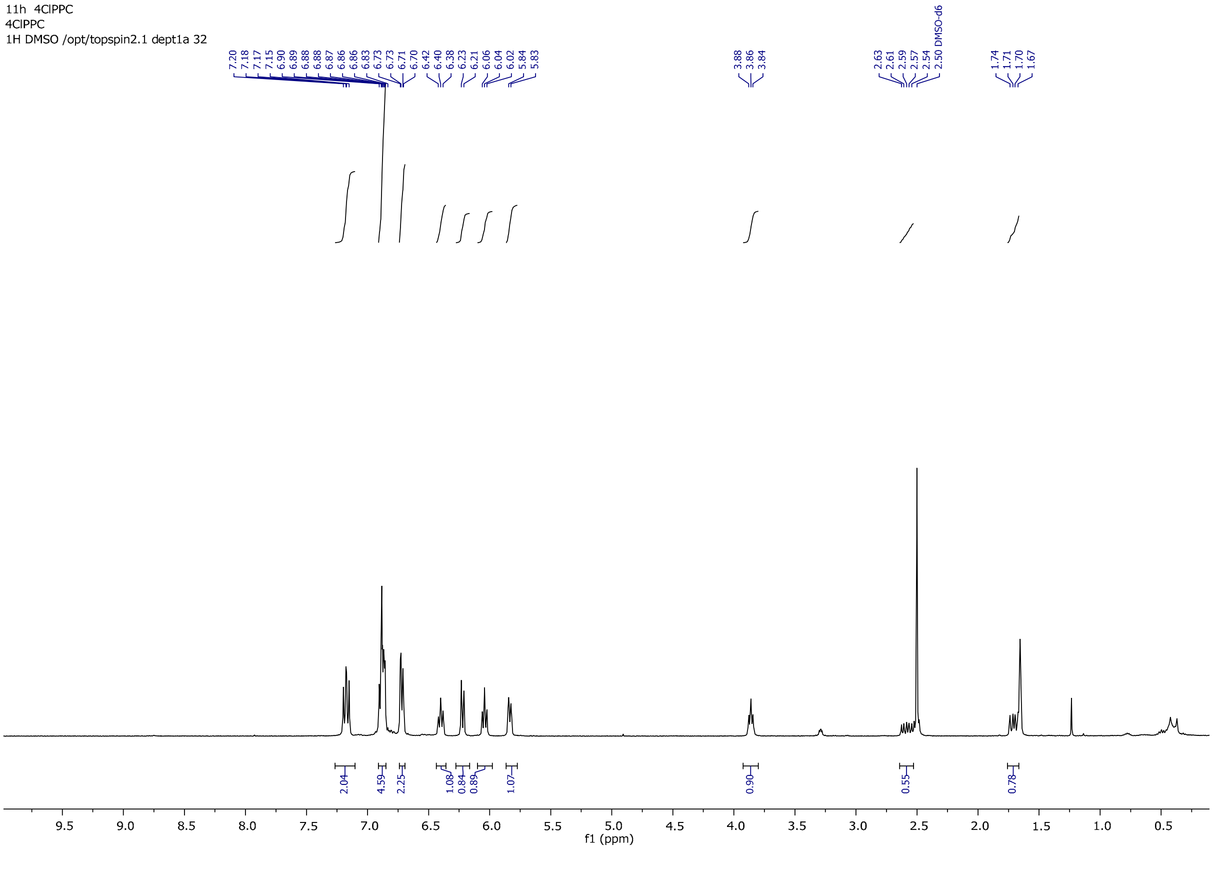

11h 4ClPPC
4ClPPC
13C{1H}_APT DMSO /opt/topspin2.1 dept1a 32

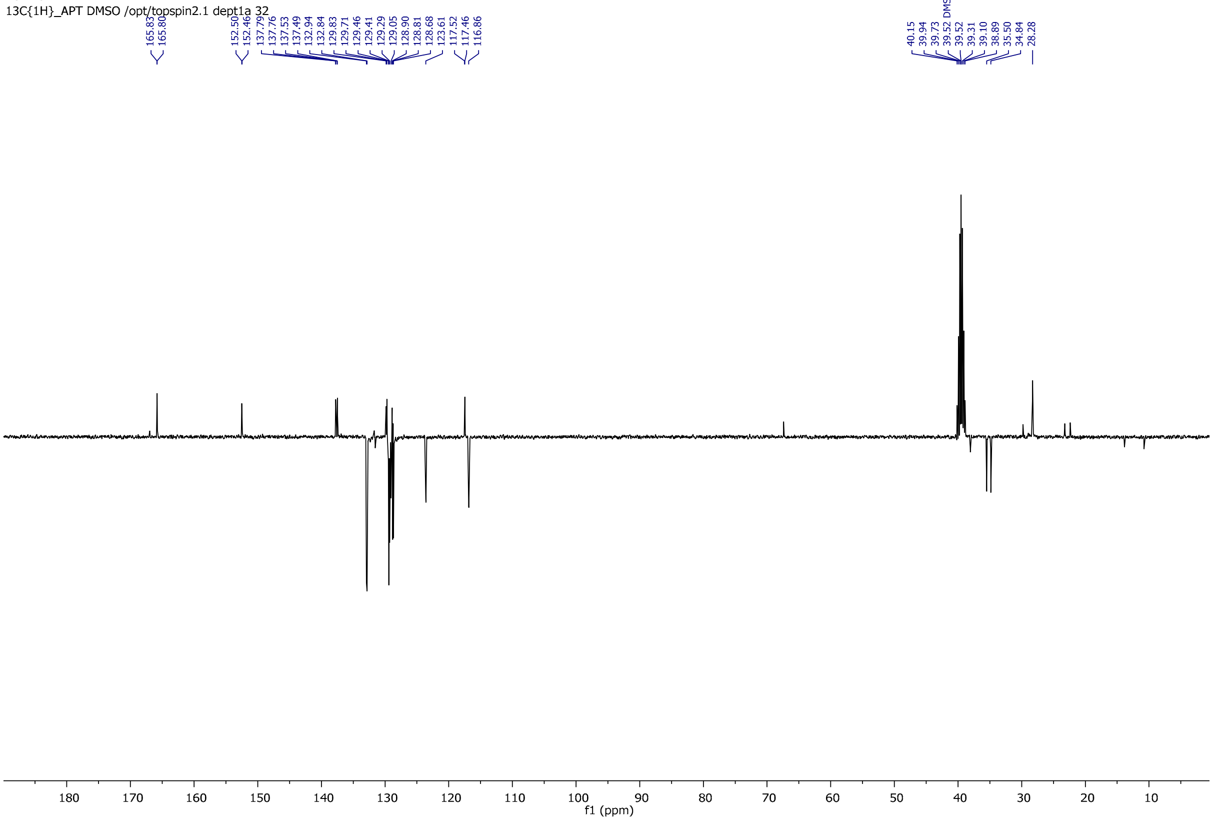

Mass Result

Analysis Info

Sample Name	**MMN-3 h _ AR 11 h**	Acquisition Date	6/29/2023 5:04:20 PM
		Instrument / Ser#	micrOTOF-Q 228888.10300

Acquisition Parameter

Source Type	ESI	Ion Polarity	Positive	Scan Begin	50 m/z	Scan End	3000 m/z

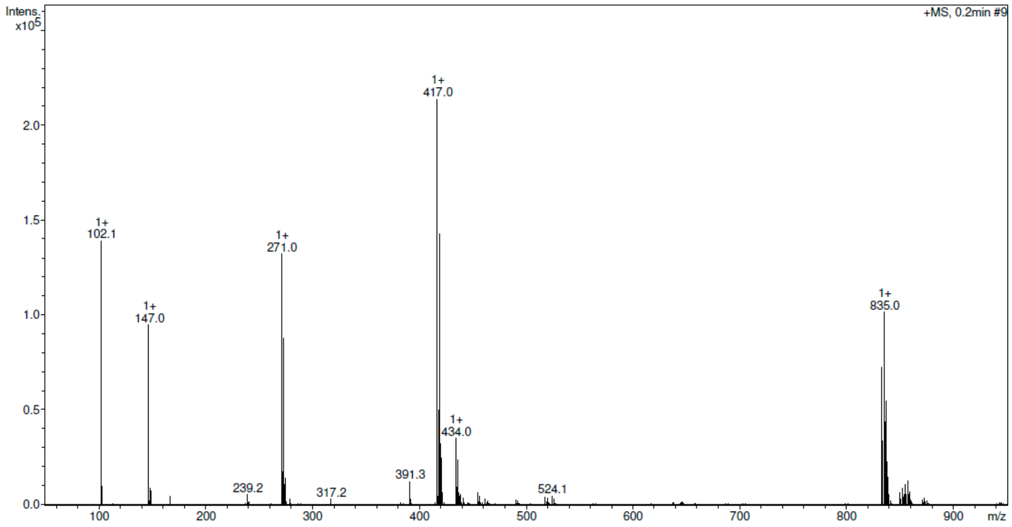

High Resolution Mass Result

Analysis Info

Sample Name **MMN-3 h _ AR 11 h**

Acquisition Date 6/29/2023 5:04:20 PM

Instrument / Ser# micrOTOF-Q 228888.10300

Acquisition Parameter

Source Type ESI | Ion Polarity Positive | Scan Begin 50 m/z | Scan End 3000 m/z

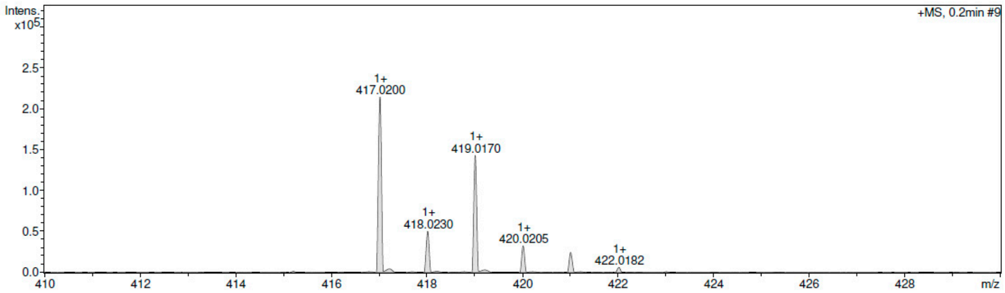

Meas. m/z	#	Ion Formula	Score	m/z	err [mDa]	err [ppm]	mSigma	rdb	e⁻ Conf	N-Rule	Adduct
417.0200	1	C21H16Cl2O3P	100.00	417.0209	0.9	2.1	4.2	13.5	even	ok	M+H
	2	C16H17Cl3N4OP	1.47	417.0200	-0.0	-0.1	135.3	9.5	even	ok	M+H
	3	C15H21Cl3O5P	0.65	417.0187	1.3	3.1	137.9	4.5	even	ok	M+H

4-(bis(2,3-dichlorophenyl)phosphoryl)chroman-2-one COUM-2o,mCl

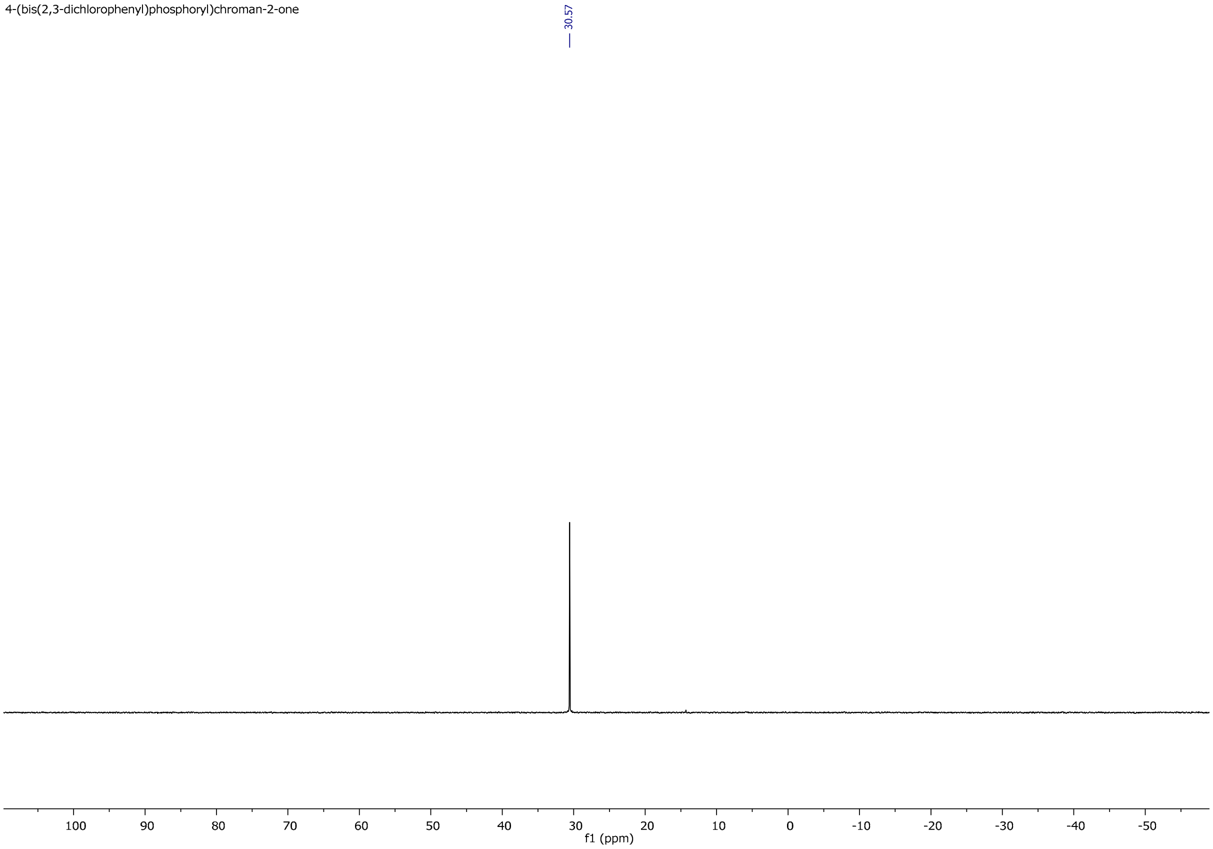

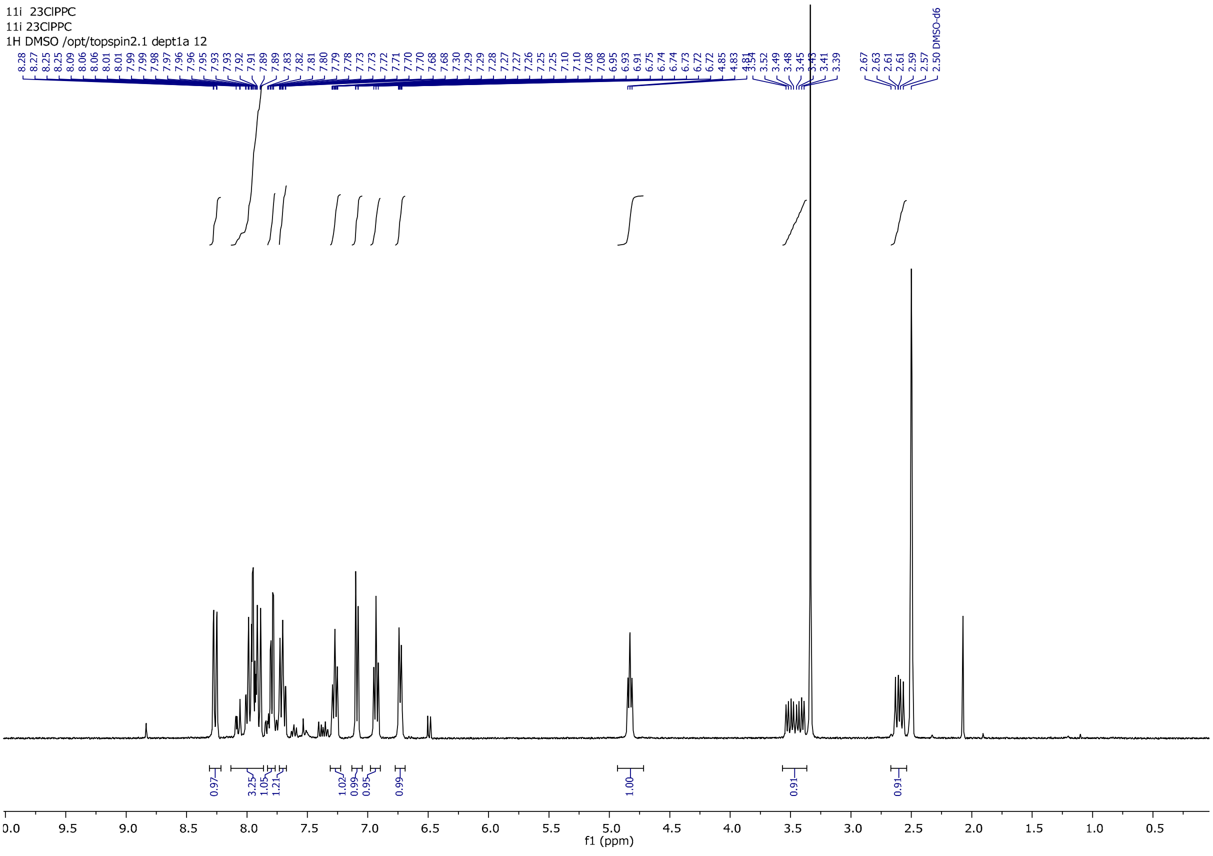

vendredi d'au revoir
11i 23ClPPC
13C{1H}_APT DMSO /opt/topspin2.1 dept1a 12

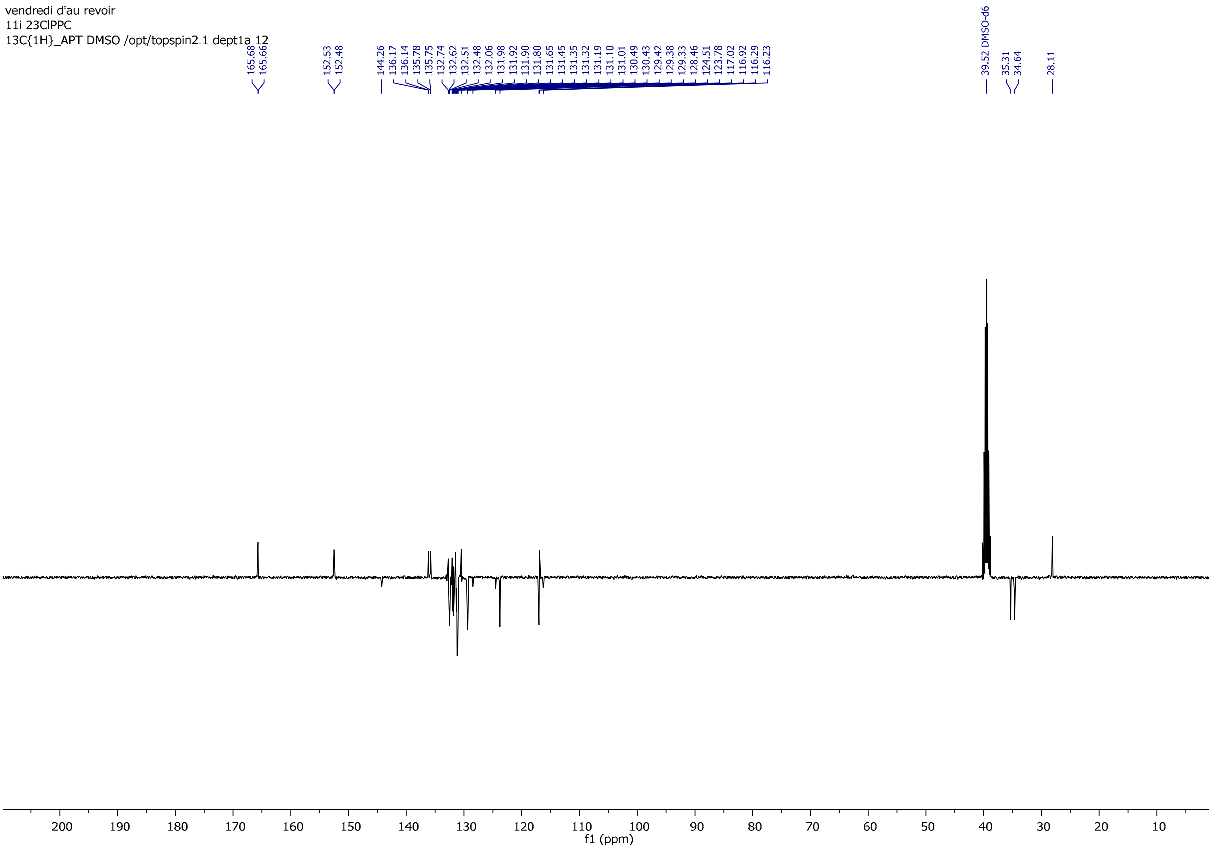

Mass Result

Analysis Info

Sample Name **MMN-3 i _ AR 11 i**

Acquisition Date 6/29/2023 5:07:23 PM

Instrument / Ser# micrOTOF-Q 228888.10300

Acquisition Parameter

Source Type ESI | Ion Polarity Positive | Scan Begin 50 m/z | Scan End 3000 m/z

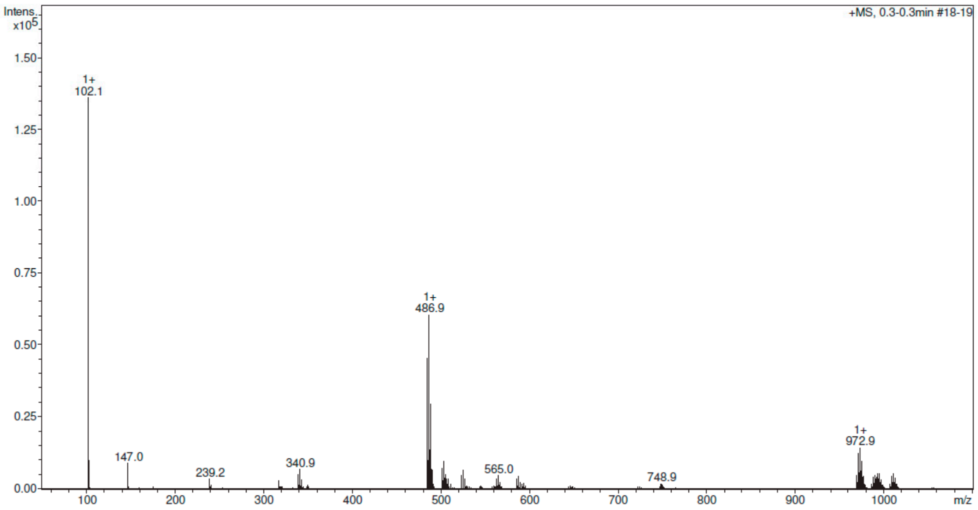

High Resolution Mass Result

Analysis Info

Sample Name	**MMN-3 i _ AR 11 i**	Acquisition Date	6/29/2023 5:07:23 PM	
		Instrument / Ser#	micrOTOF-Q	228888.10300

Acquisition Parameter

Source Type	ESI	Ion Polarity	Positive	Scan Begin	50 m/z	Scan End	3000 m/z

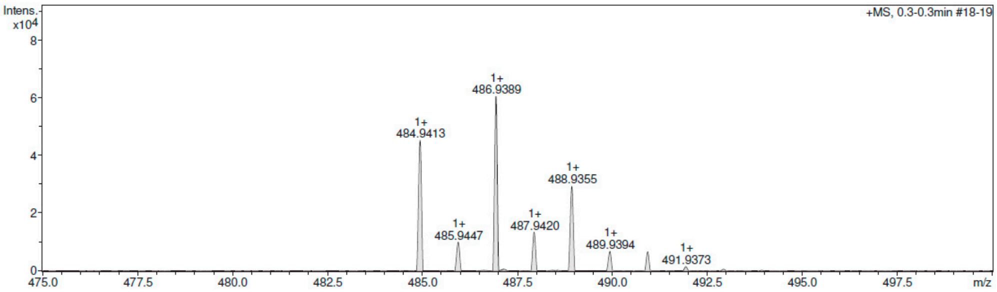

Meas. m/z	#	Ion Formula	Score	m/z	err [mDa]	err [ppm]	mSigma	rdb	e⁻ Conf	N-Rule	Adduct
484.9413	1	C21H16Br2N2P	100.00	484.9412	0.1	0.1	100.5	14.5	even	ok	M+H
	2	C19H21Br2OP2	41.42	484.9429	1.6	3.3	101.5	9.5	even	ok	M+H

4-(bis(3,4,5-trichlorophenyl)phosphoryl)chroman-2-one COUM-2m,m,pCl

rmn km 254 trichloro
KM254 PUR F2
31P{1H} CDCl3 /opt/topspin2.1 dept1a 2

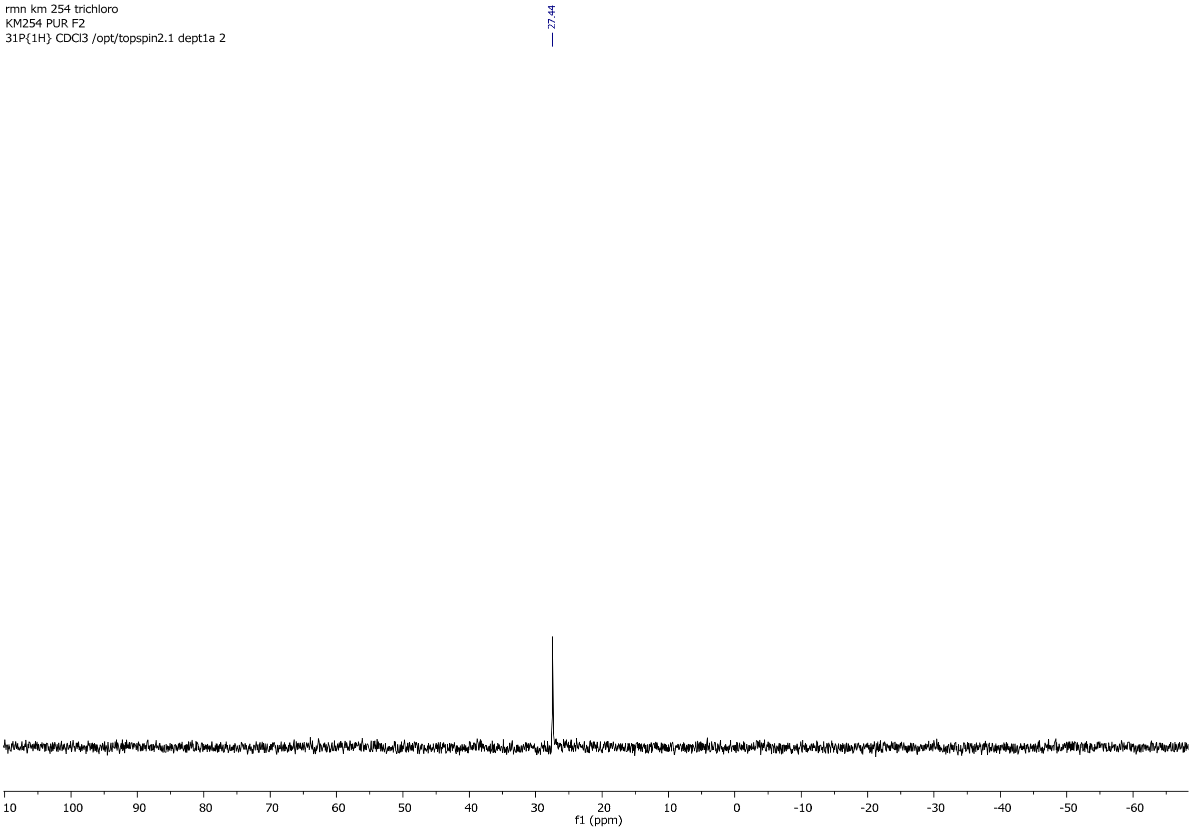

rmn km 254 trichloro
KM254 PUR F2
1H CDCl3 /opt/topspin2.1 dept1a 2

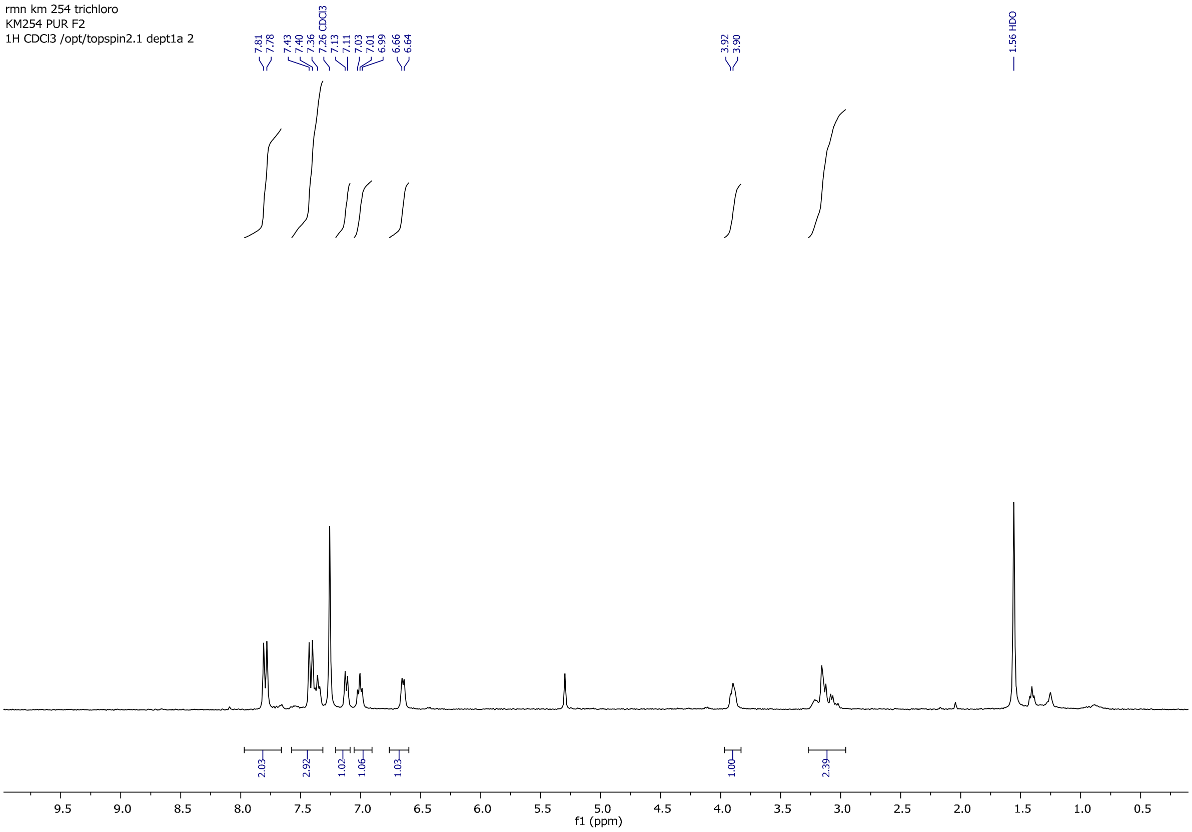

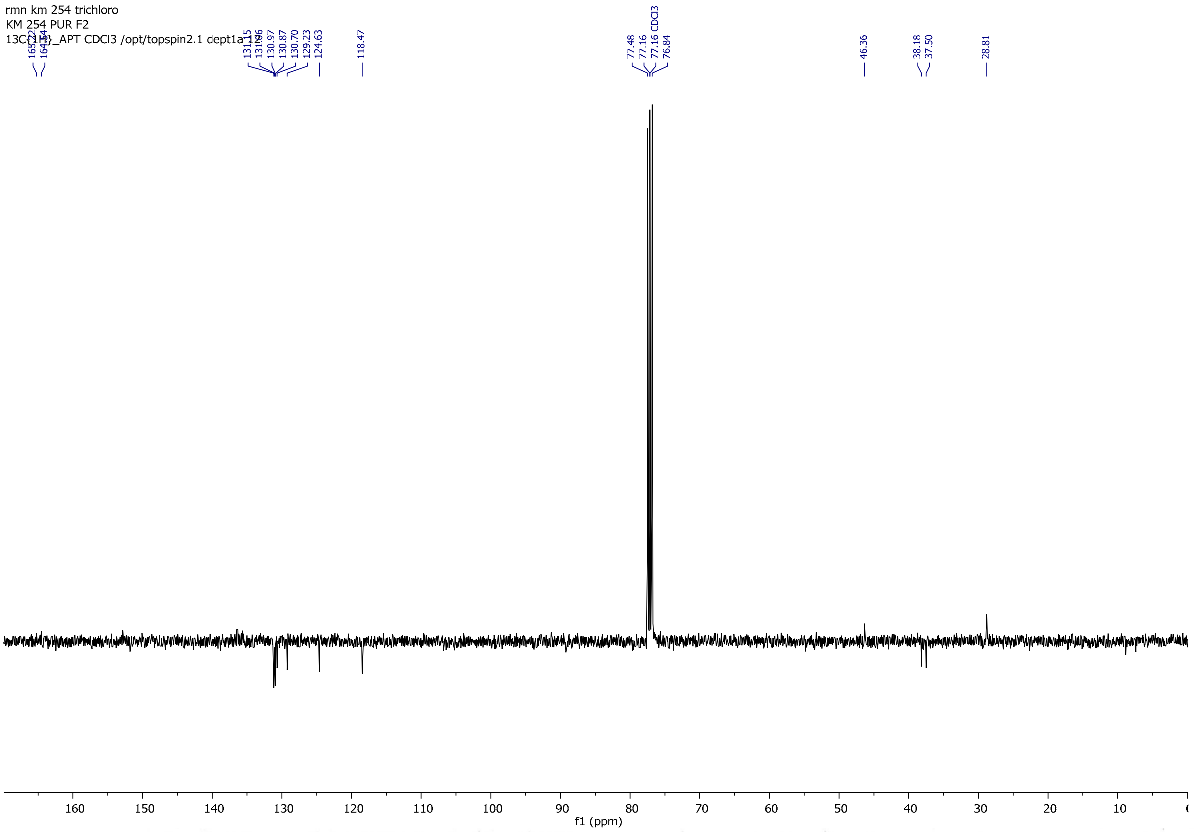
rmn km 254 trichloro
KM 254 PUR F2
13C{1H}_APT CDCl3 /opt/topspin2.1 dept1a 1
131.15
130.97
130.87
130.70
129.23
124.63
118.47
77.48
77.16
77.16 CDCl3
76.84
46.36
38.18
37.50
28.81
f1 (ppm)

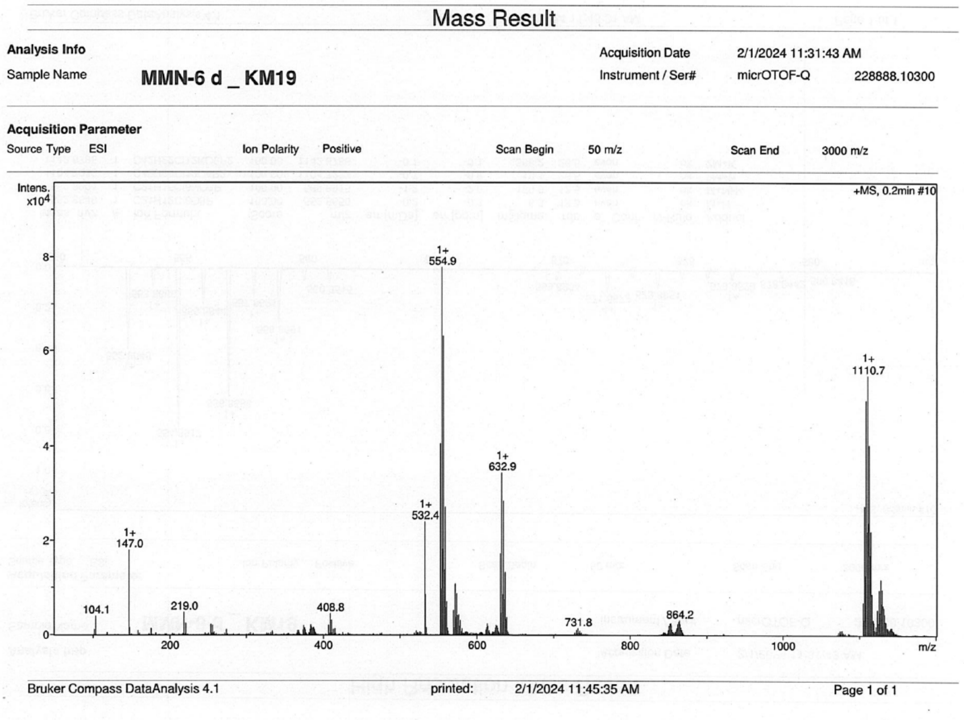
Mass Result
Analysis Info
Sample Name MMN-6 d _ KM19
Acquisition Date 2/1/2024 11:31:43 AM
Instrument / Ser# micrOTOF-Q 228888.10300
Acquisition Parameter
Source Type ESI
Ion Polarity Positive
Scan Begin 50 m/z
Scan End 3000 m/z
Intens. x10^4
+MS, 0.2min #10
1+ 554.9
1+ 1110.7
1+ 632.9
1+ 532.4
1+ 147.0
104.1
219.0
408.8
731.8
864.2
m/z
Bruker Compass DataAnalysis 4.1
printed: 2/1/2024 11:45:35 AM
Page 1 of 1

High Resolution Mass Result

**Analysis Info**		Acquisition Date	2/1/2024 11:31:43 AM	
Sample Name	**MMN-6 d _ KM19**	Instrument / Ser#	micrOTOF-Q	228888.10300

Acquisition Parameter

Source Type	ESI	Ion Polarity	Positive	Scan Begin	50 m/z	Scan End	3000 m/z

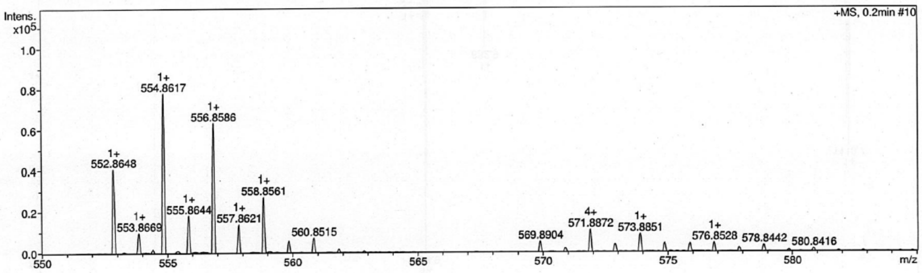

Meas. m/z	#	Ion Formula	Score	m/z	err [mDa]	err [ppm]	mSigma	rdb	e⁻ Conf	N-Rule	Adduct
552.8648	1	C21H12Cl6O3P	100.00	552.8650	-0.2	-0.3	6.3	13.5	even	ok	M+H
569.8904	1	C21H15Cl6NO3P	100.00	569.8915	-1.2	-2.0	150.9	12.5	even	ok	M+NH4
1104.7233	1	C42H23Cl12O6P2	100.00	1104.7227	-0.7	-0.6	10.4	26.5	even	ok	2M+H
1142.6786	1	C42H22Cl12KO6P2	100.00	1142.6786	-0.1	-0.1	368.2	26.5	even	ok	2M+K

4-(bis(4-bromophenyl)phosphoryl)chroman-2-one COUM-2pBr

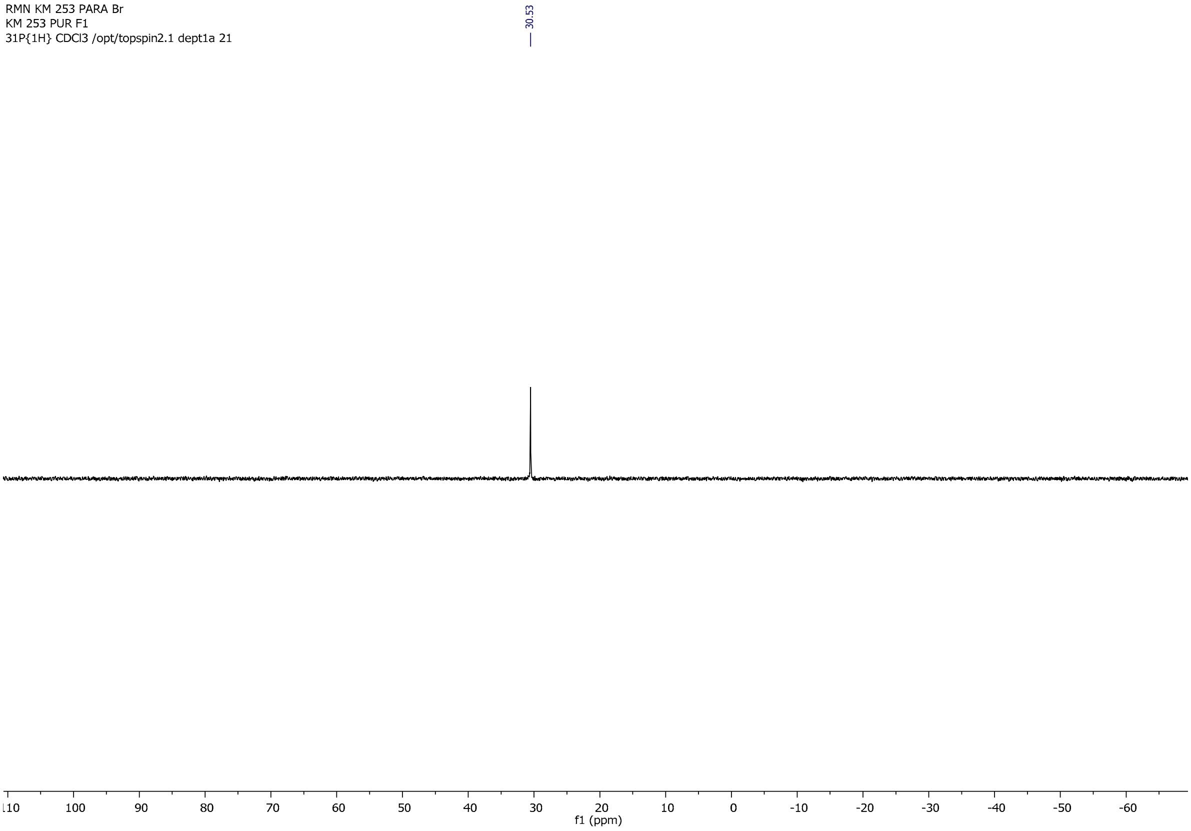

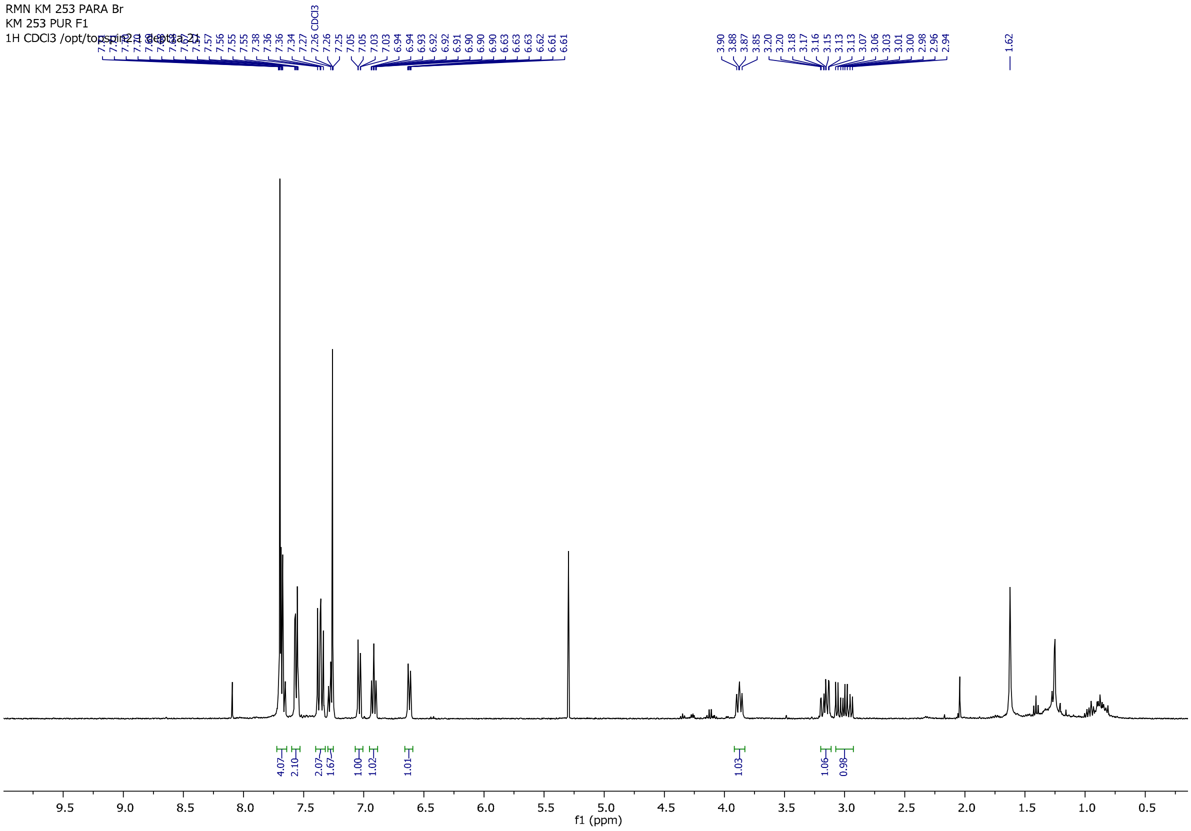

RMN KM 253 PARA Br
KM 253 PUR 1
13C{1H}_APT CDCl3 /opt/topspin2.1 dept1a 11

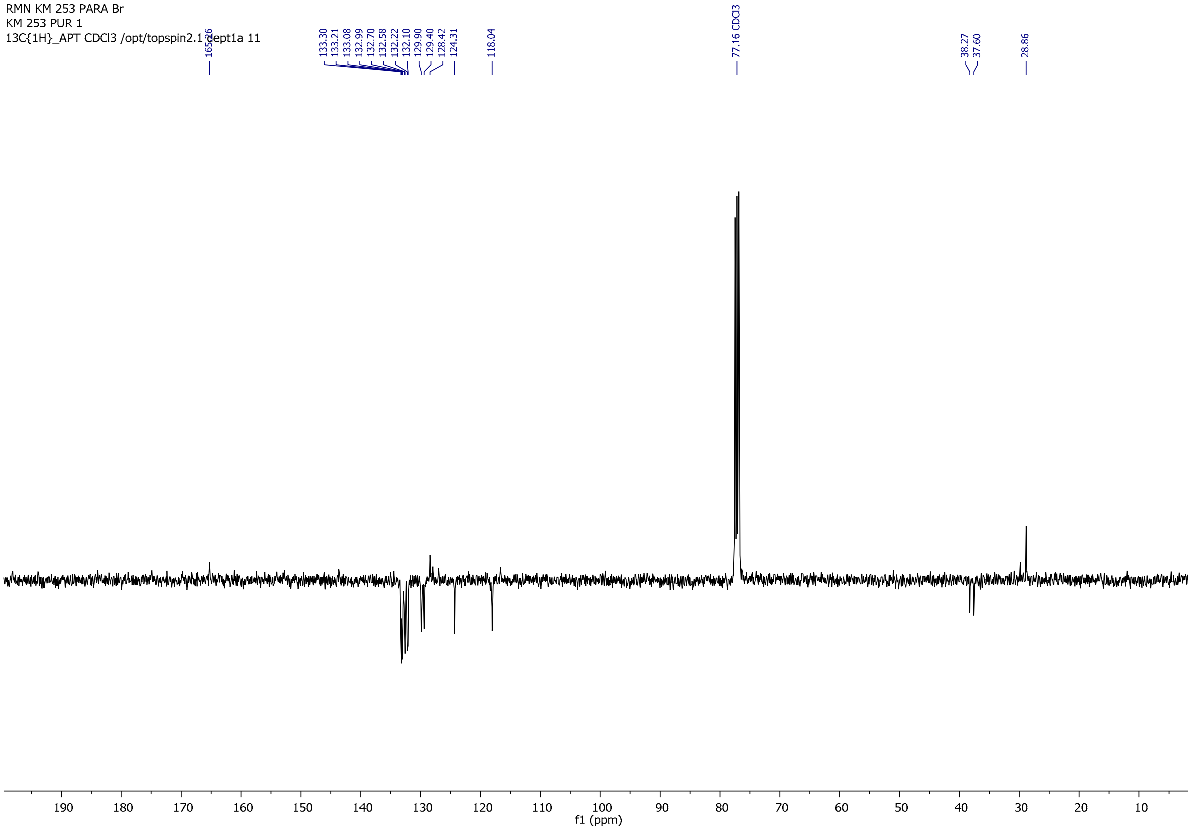

Mass Result

Analysis Info

Sample Name **MMN-6 e _ KM20**

Acquisition Date 2/1/2024 11:34:46 AM

Instrument / Ser# micrOTOF-Q 228888.10300

Acquisition Parameter

Source Type ESI Ion Polarity Positive Scan Begin 50 m/z Scan End 3000 m/z

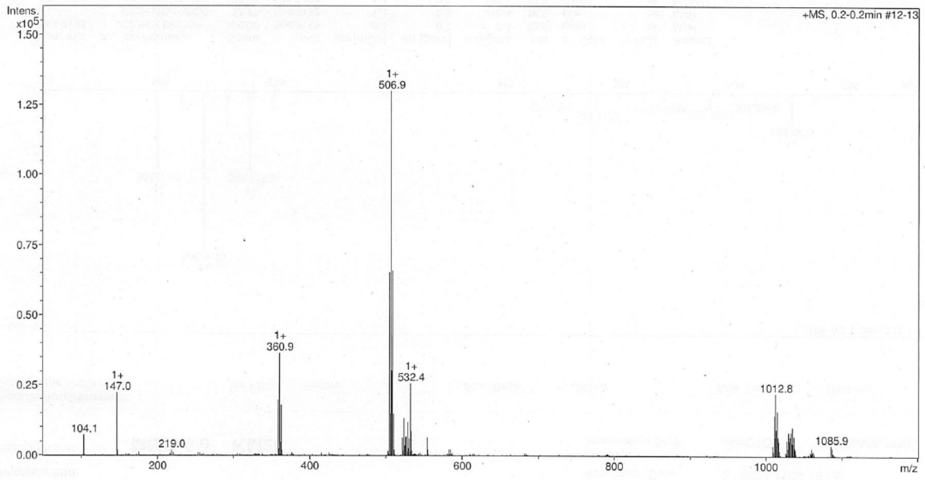

Bruker Compass DataAnalysis 4.1 printed: 2/1/2024 11:49:20 AM Page 1 of 1

High Resolution Mass Result

Analysis Info

Sample Name **MMN-6 e _ KM20**

Acquisition Date 2/1/2024 11:34:46 AM

Instrument / Ser# micrOTOF-Q 228888.10300

Acquisition Parameter

Source Type ESI | Ion Polarity Positive | Scan Begin 50 m/z | Scan End 3000 m/z

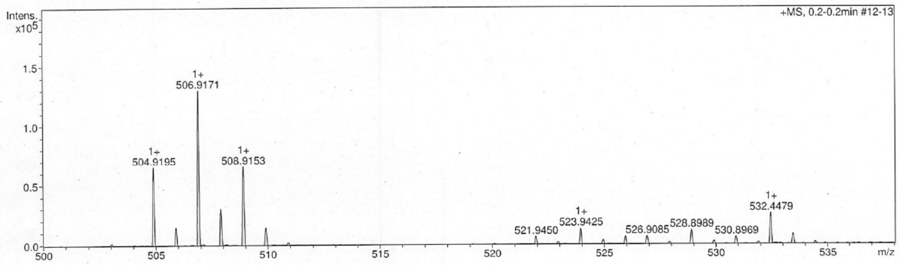

Meas. m/z	#	Ion Formula	Score	m/z	err [mDa]	err [ppm]	mSigma	rdb	e⁻ Conf	N-Rule	Adduct
504.9195	1	C21H16Br2O3P	100.00	504.9198	0.4	0.7	3.1	13.5	even	ok	M+H
	2	C23H11Br2N2O2	53.75	504.9182	1.3	2.5	10.4	18.5	even	ok	M+H
521.9450	1	C21H19Br2NO3P	100.00	521.9464	1.4	2.7	156.6	12.5	even	ok	M+NH4

4-(diphenylphosphoryl)-2-phenyl-1,3,4-trihydroisophosphinoline 2-oxide ISOP-Ha

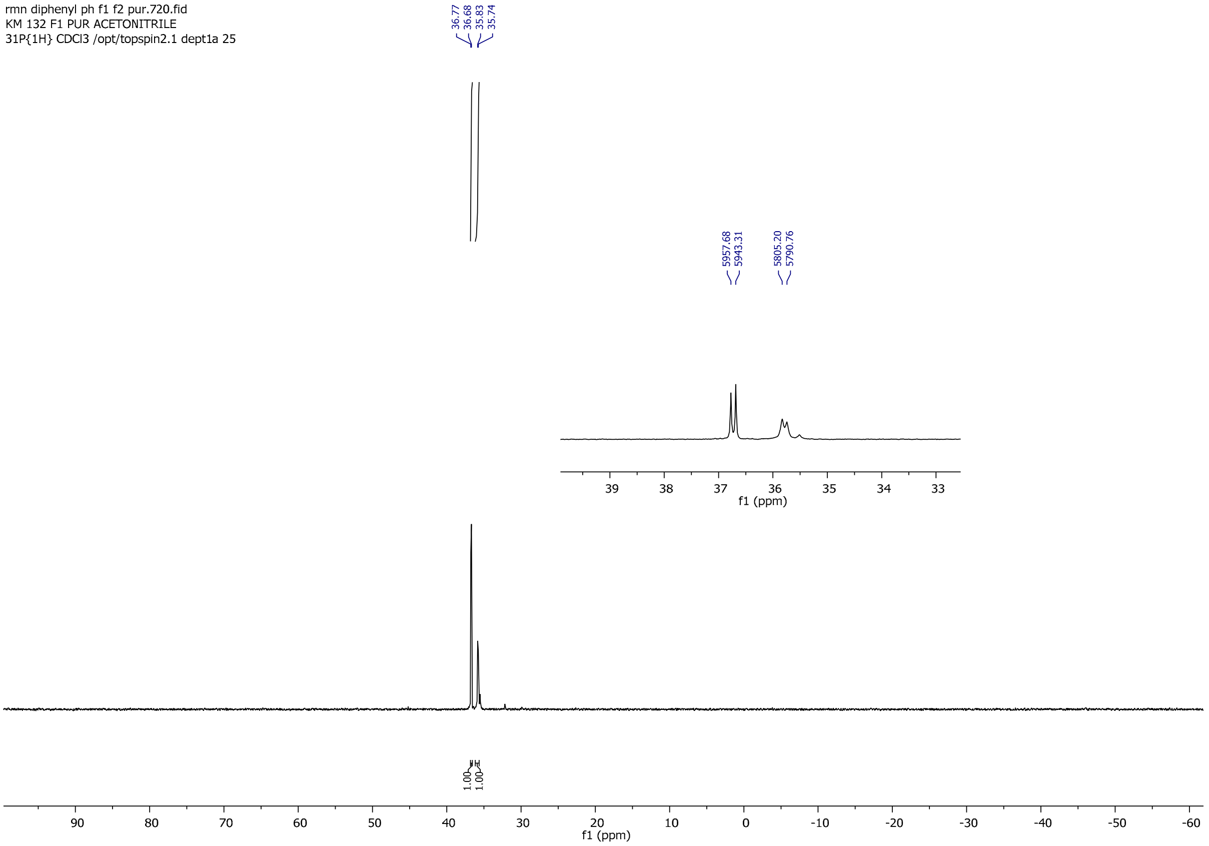

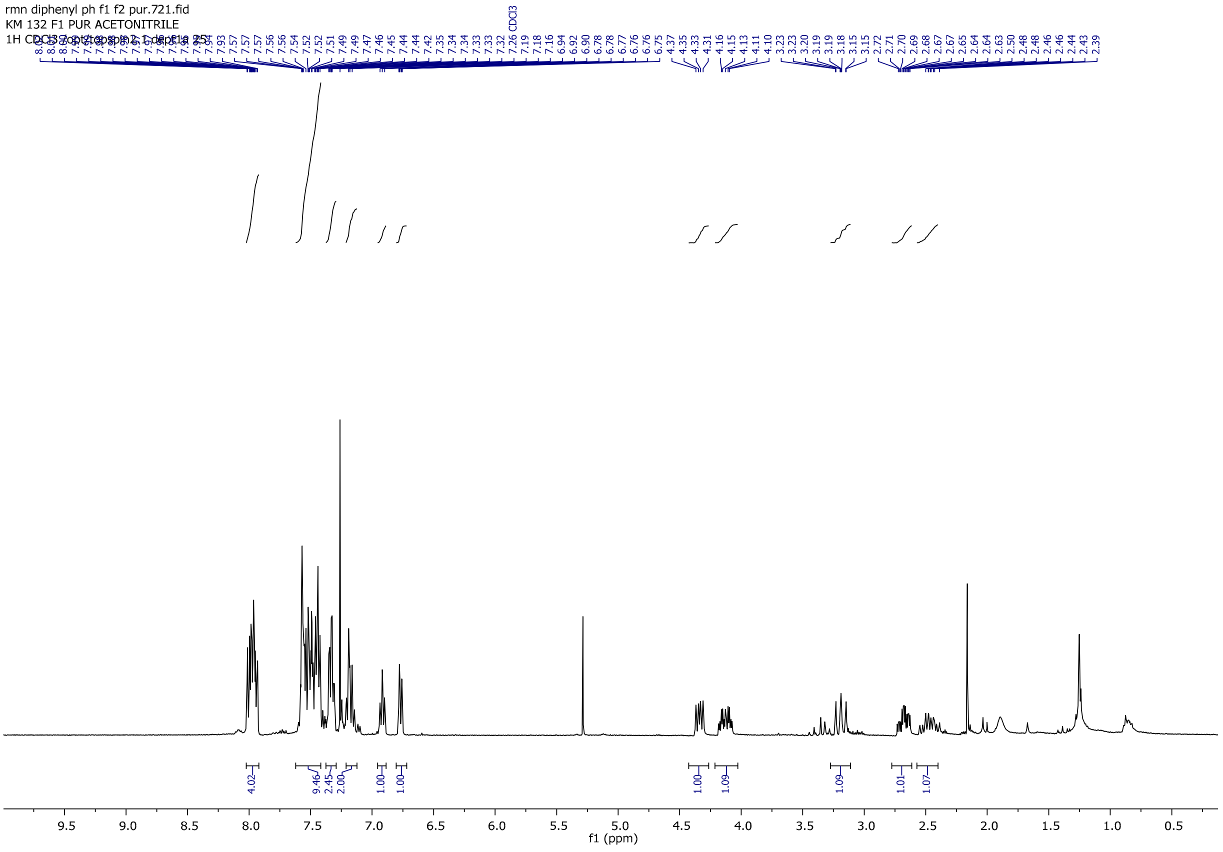

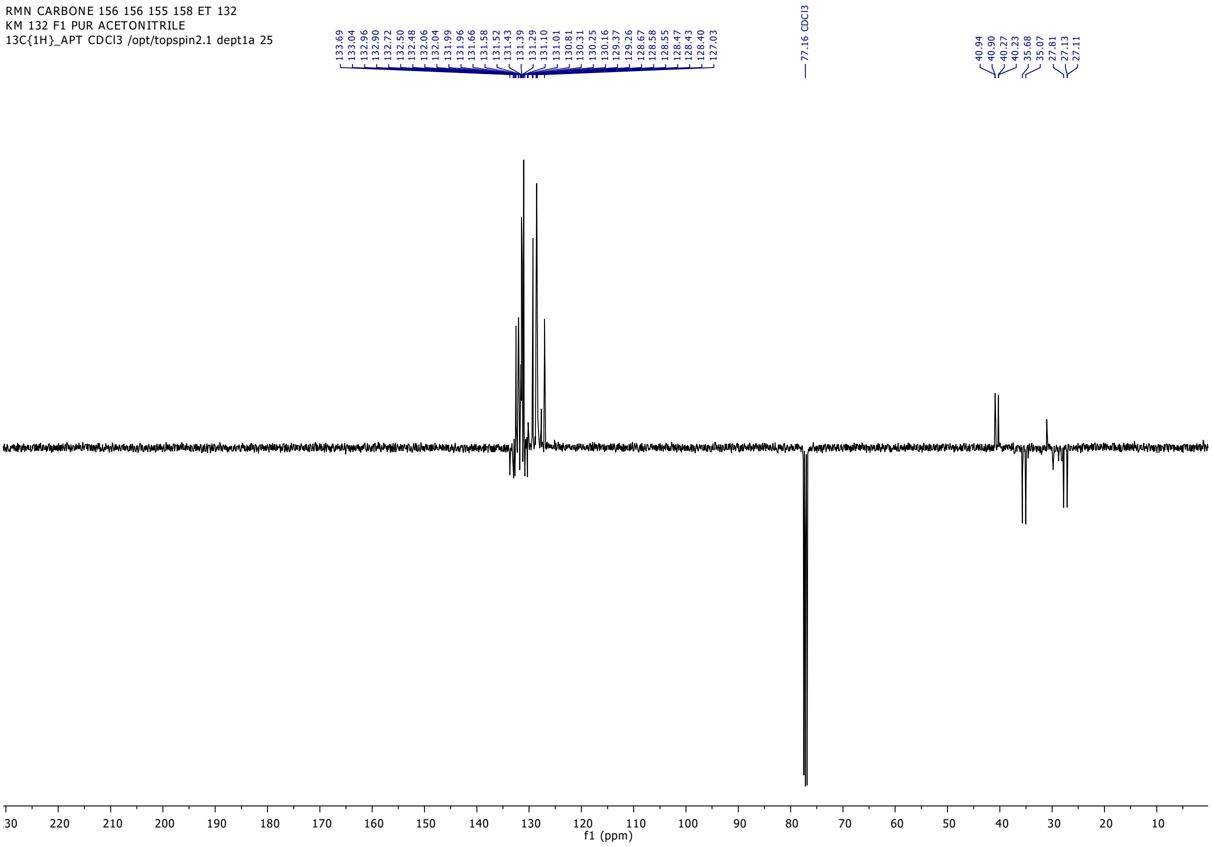

Acquisition Parameter

Source Type	ESI	Ion Polarity	Positive	Scan Begin	50 m/z	Scan End	2200 m/z

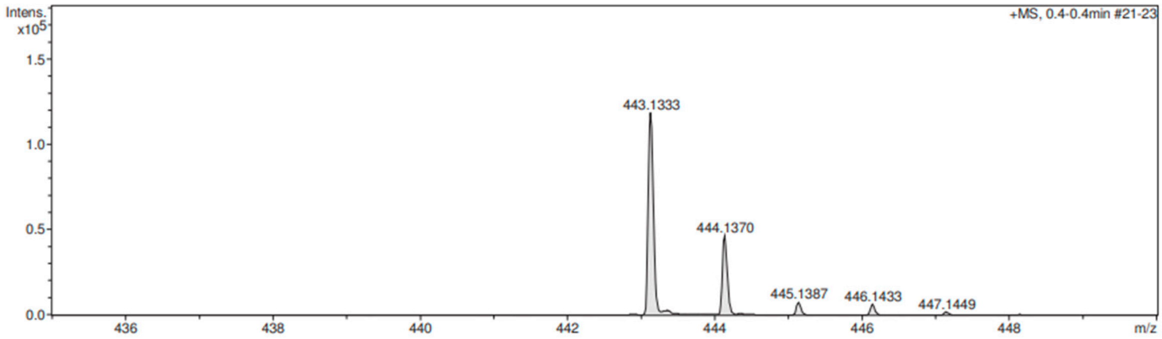

Meas. m/z	#	Ion Formula	m/z	err [ppm]	mSigma	# Sigma	Score	rdb	e⁻ Conf	N-Rule
443.1333	1	C27H25O2P2	443.1324	-2.0	55.3	1	100.00	16.5	even	ok

4-(diphenylphosphoryl)-2-phenyl-1,3,4-trihydroisophosphinoline 2-oxide ISOP-Ha'

rmn diphenyl ph f1 f2 pur.723.fid
KM 132 F2 PUR ACETONITRILE
31P{1H} CDCl3 /opt/topspin2.1 dept1a 26

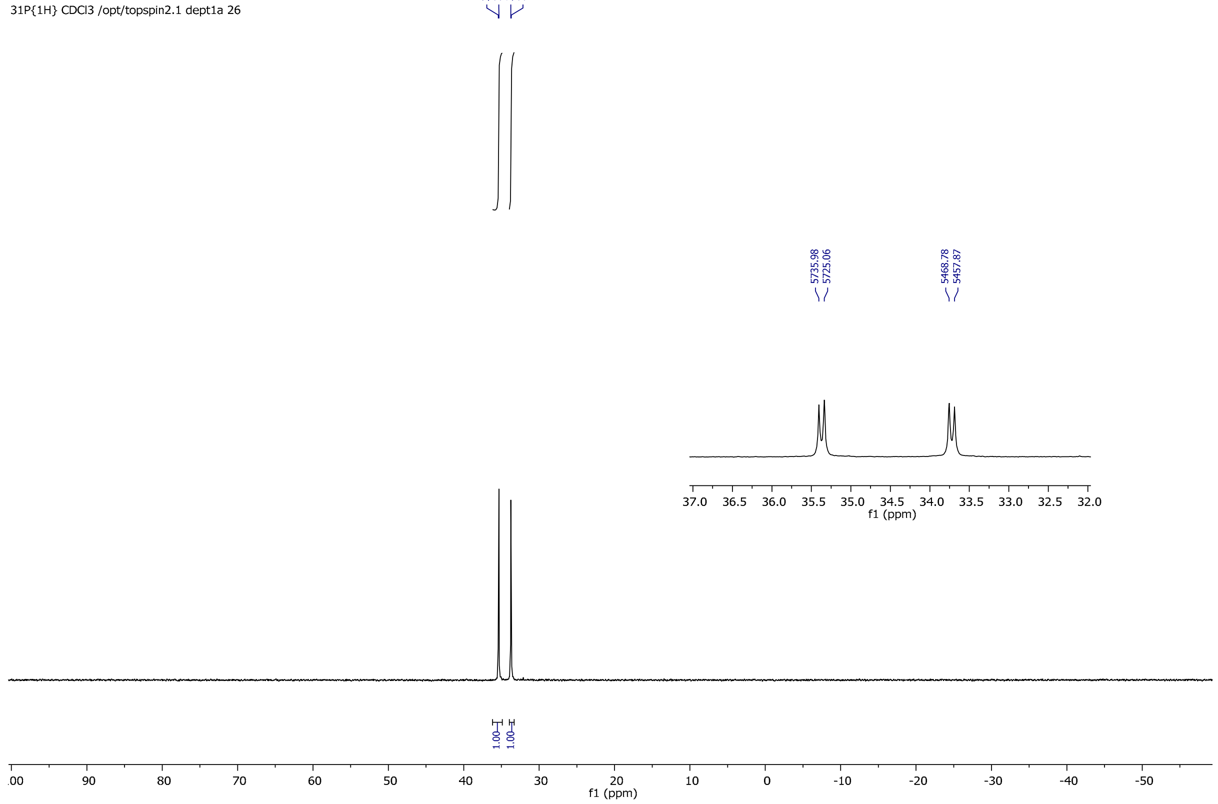

rmn diphenyl ph f1 f2 pur.724.fid
KM 132 F2 PUR ACETONITRILE
1H CDCl3 /opt/topspin2.1 dept1a 26

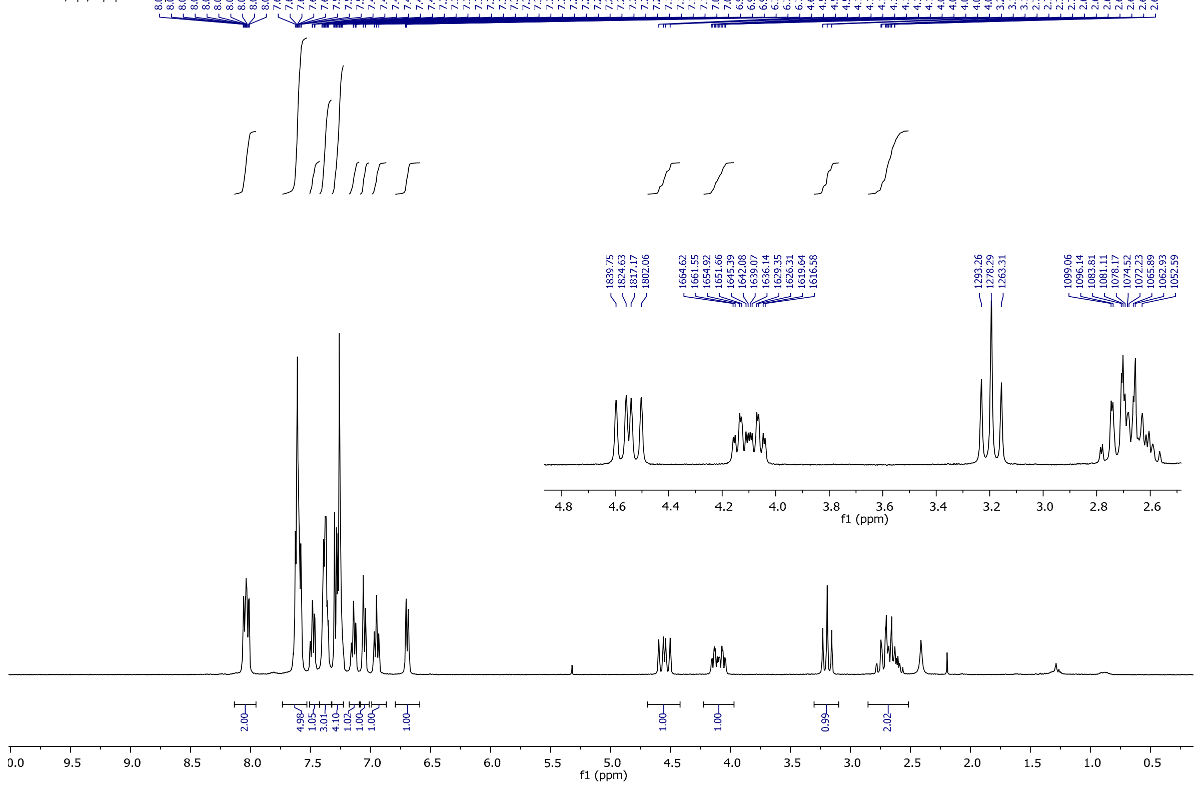

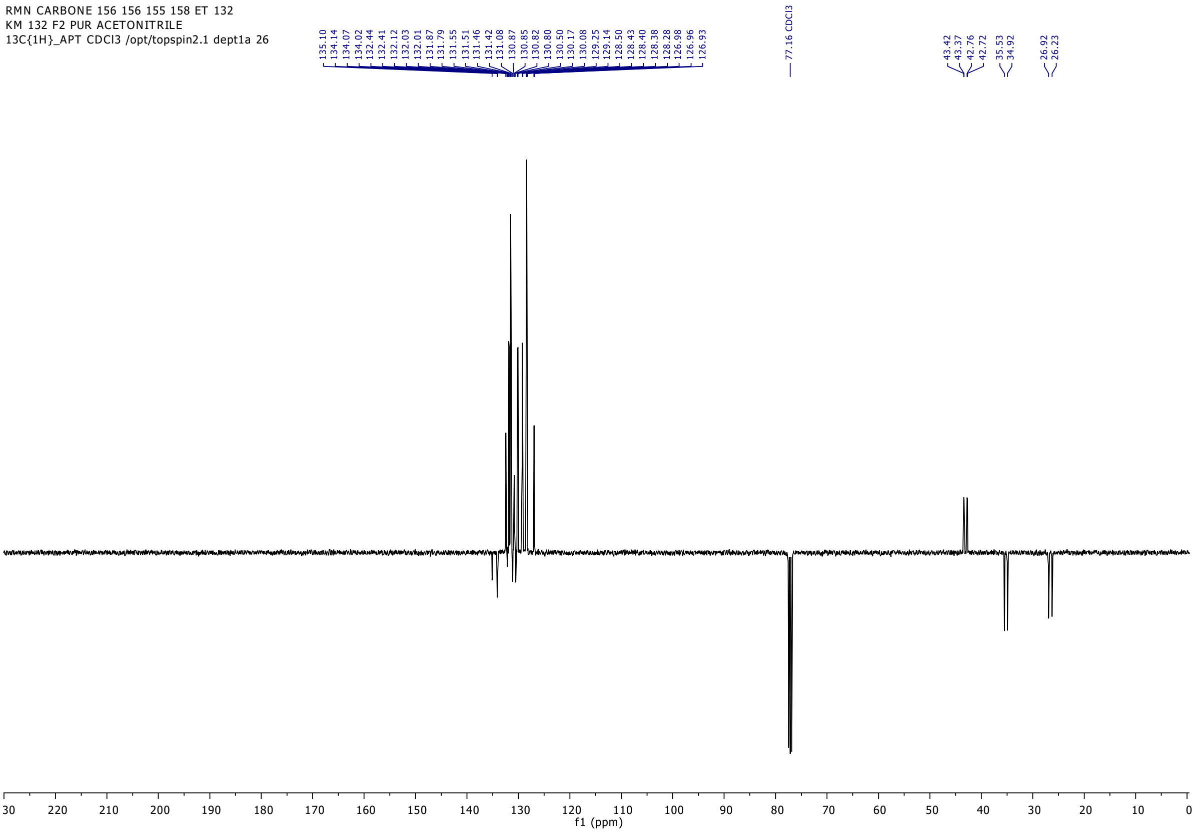

Acquisition Parameter

Source Type	ESI	Ion Polarity	Positive	Scan Begin	50 m/z	Scan End	2200 m/z

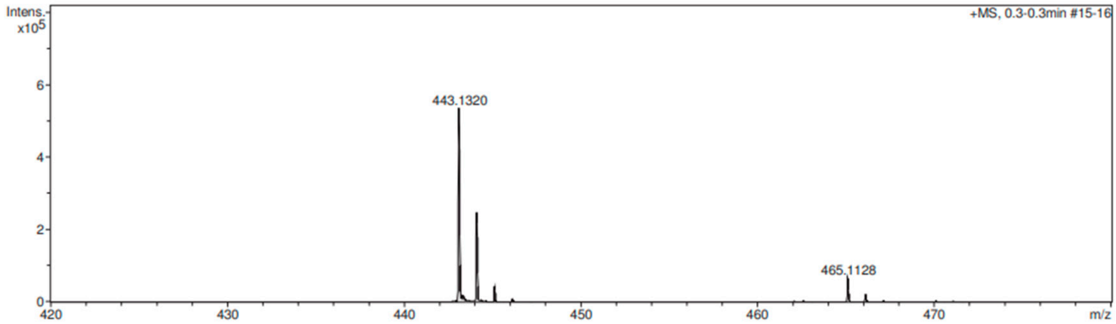

Meas. m/z	#	Ion Formula	m/z	err [ppm]	mSigma	# Sigma	Score	rdb	e⁻ Conf	N-Rule
443.1320	1	C27H25O2P2	443.1324	0.9	85.8	1	100.00	16.5	even	ok
	2	C11H25N8O7P2	443.1316	-0.9	181.7	2	0.24	4.5	even	ok

4-(di-o-tolyphosphoryl)-2-phenyl-1,3,4-trihydroisophosphinoline 2-oxide ISOP-oMea

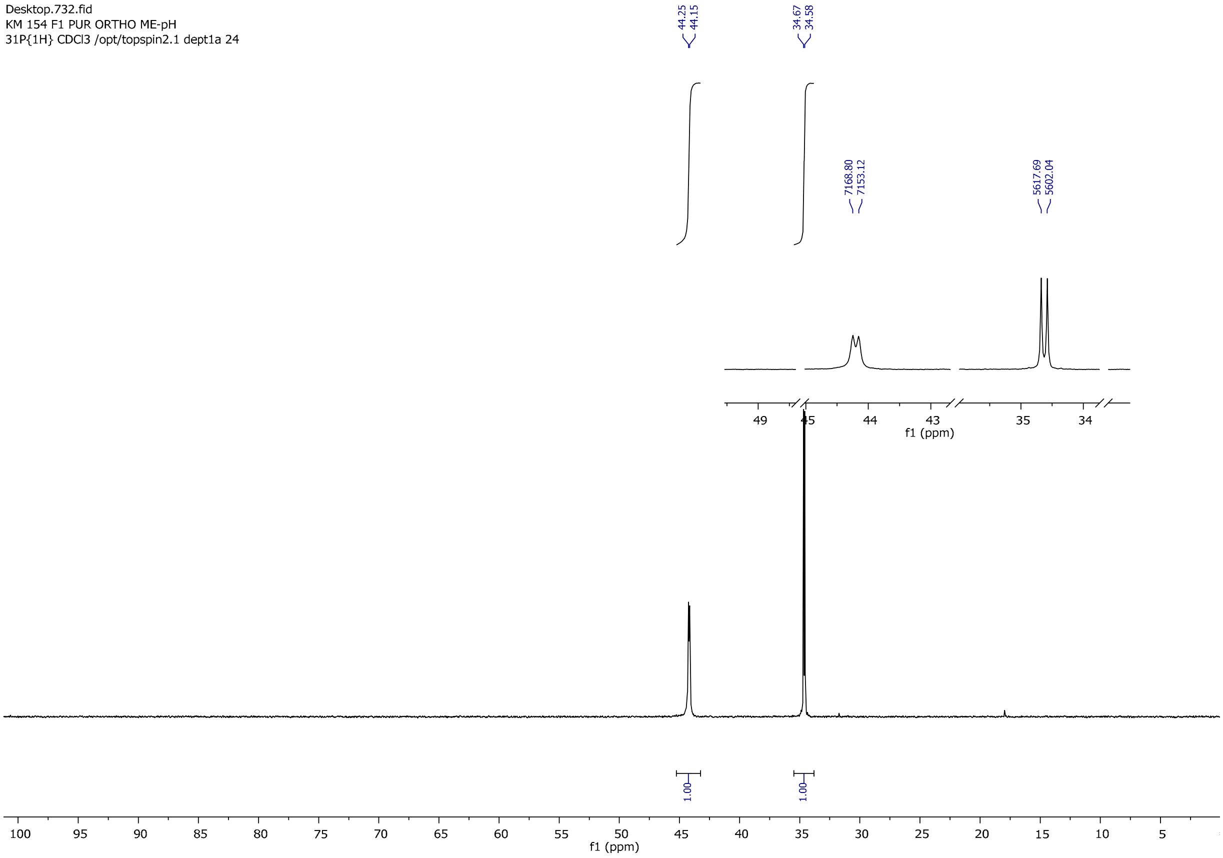

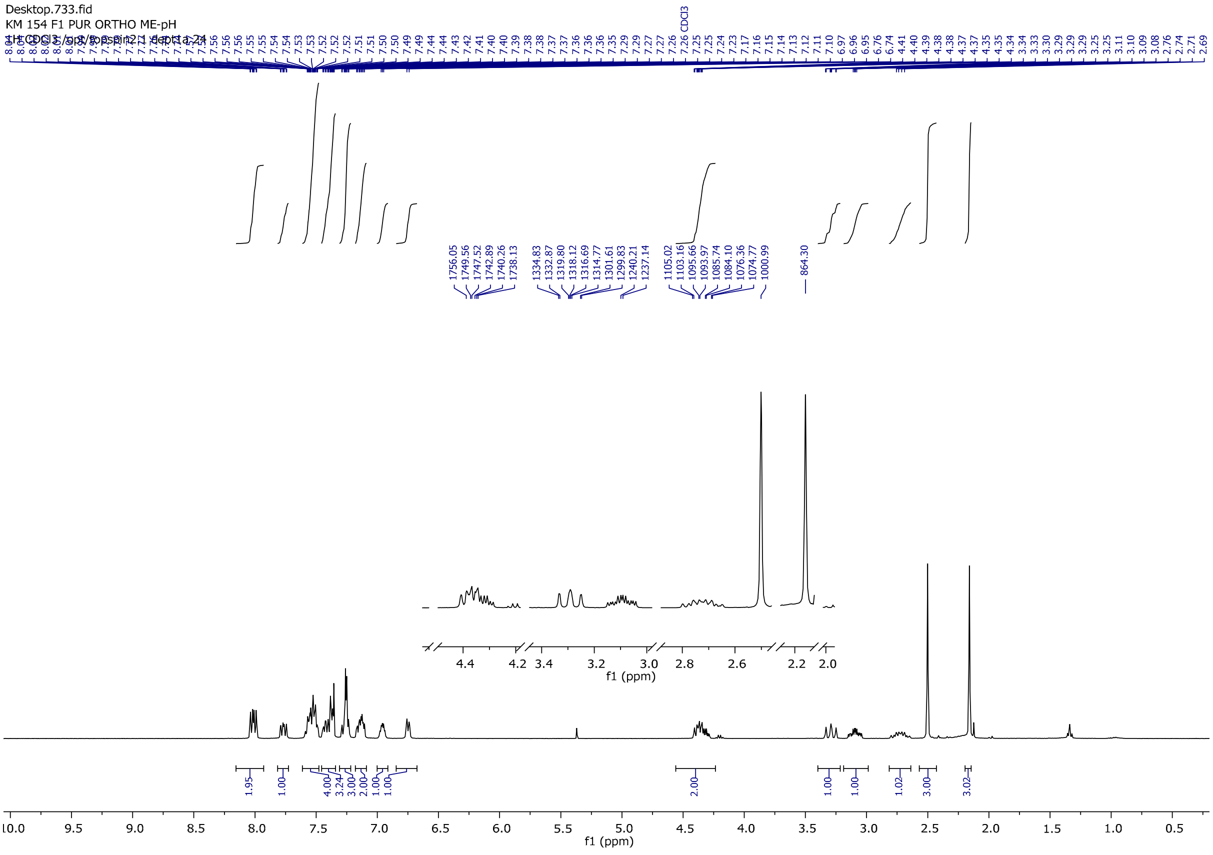

RMN CARBONE KM 153 ET KM154
KM 154 F1 PUR ORTHO Me-pH
13C{1H}_APT CDCl3 /opt/topspin2.1 dept1a 7

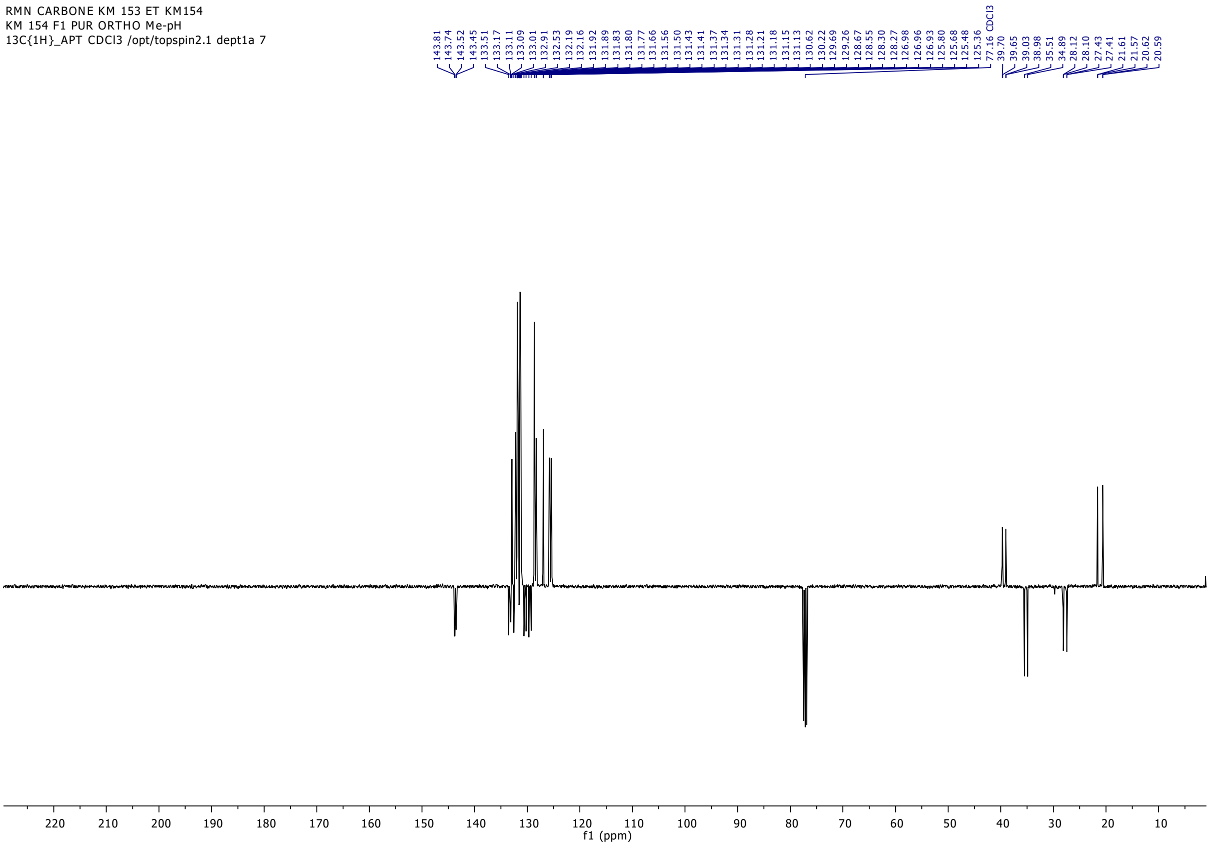

High Resolution Mass Result

Analysis Info

Sample Name **MMM-1 c _ KM154 f1**

Acquisition Date 7/26/2022 11:36:25 AM

Instrument / Ser# micrOTOF-Q 228888.10300

Acquisition Parameter

Source Type ESI | Ion Polarity Positive | Scan Begin 50 m/z | Scan End 2200 m/z

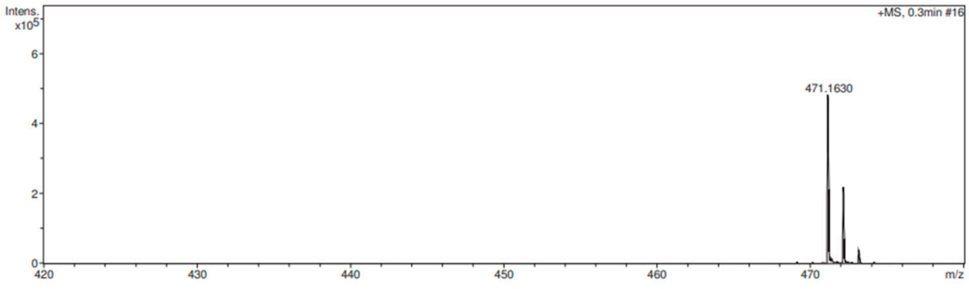

Meas. m/z	#	Ion Formula	m/z	err [ppm]	mSigma	# Sigma	Score	rdb	e⁻ Conf	N-Rule
471.1630	1	C29H29O2P2	471.1637	1.6	70.4	1	100.00	16.5	even	ok
	2	C13H29N8O7P2	471.1629	-0.2	164.4	2	0.66	4.5	even	ok

4-(di-o-tolyphosphoryl)-2-phenyl-1,3,4-trihydroisophosphinoline 2-oxide ISOP-oMea’

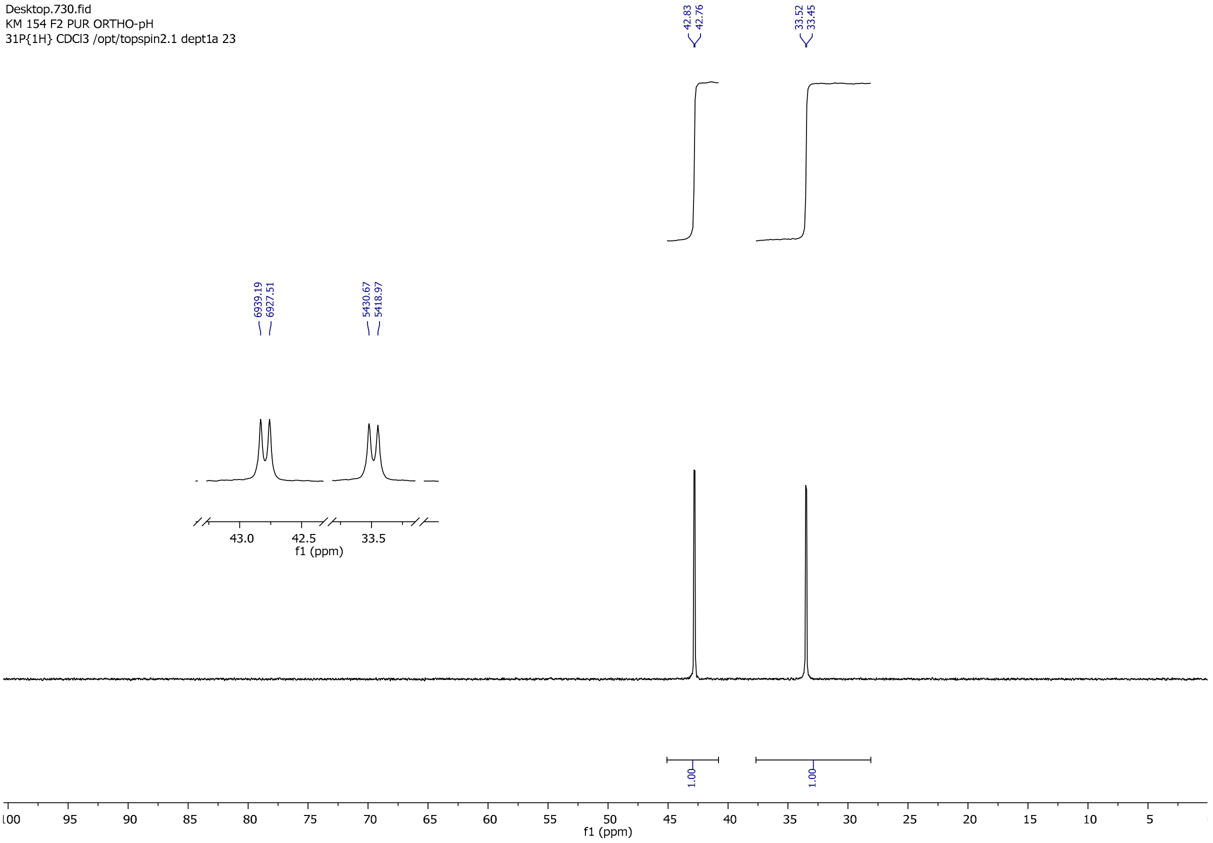

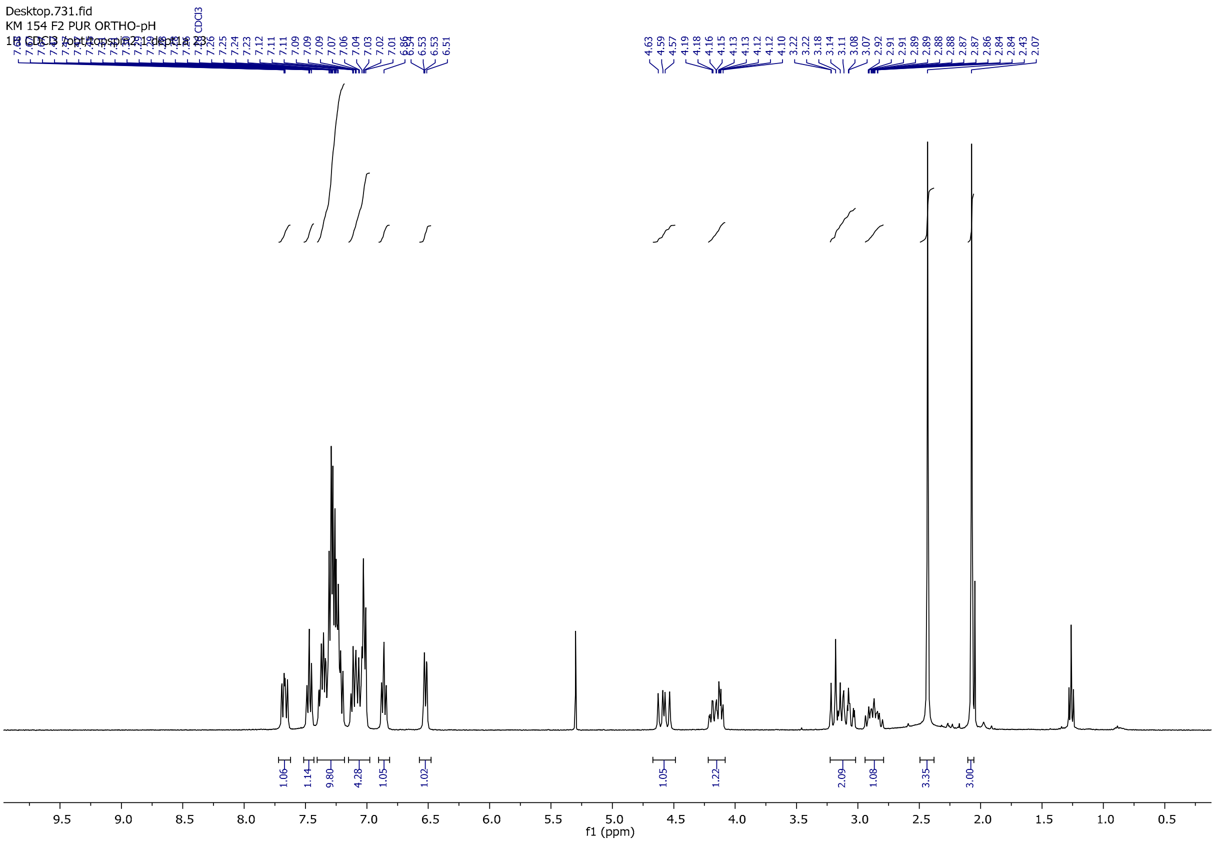

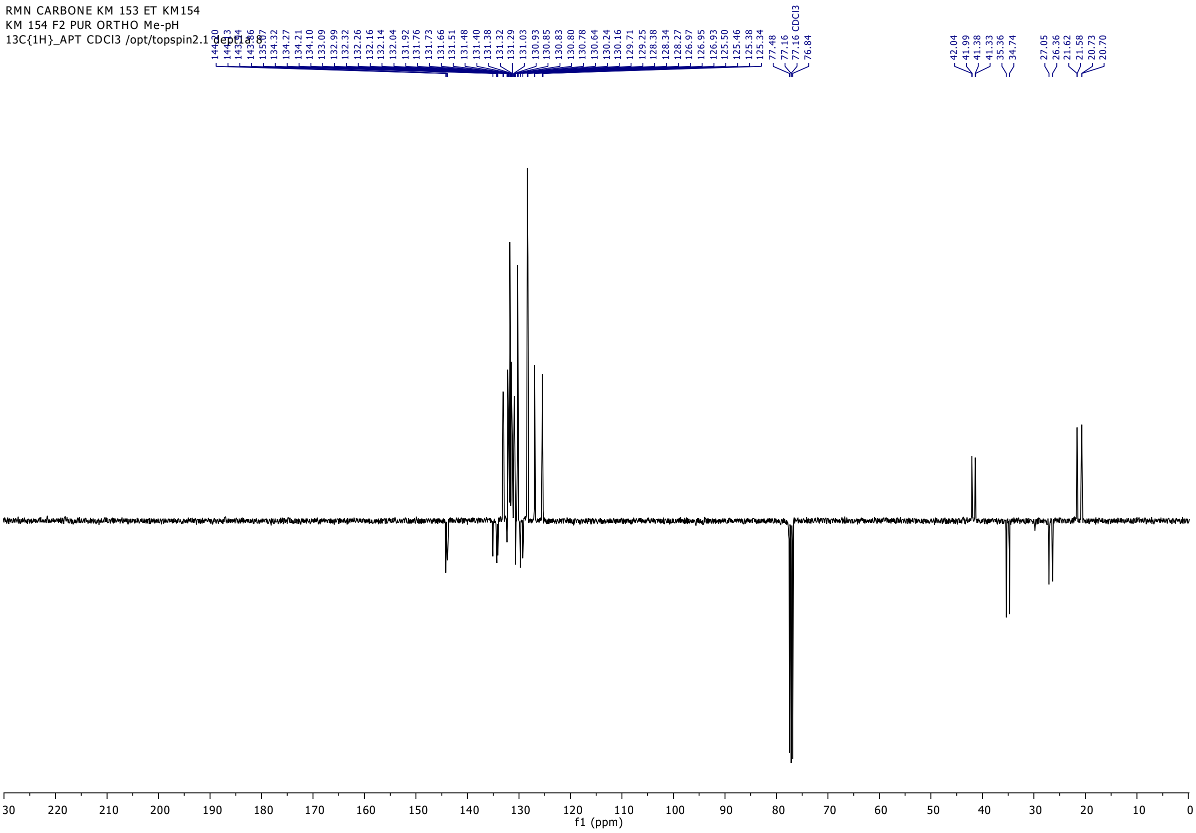

High Resolution Mass Result

Analysis Info

Sample Name **MMM-1 d _ KM154 f2**

Acquisition Date 7/26/2022 11:39:27 AM

Instrument / Ser# micrOTOF-Q 228888.10300

Acquisition Parameter

Source Type ESI Ion Polarity Positive Scan Begin 50 m/z Scan End 2200 m/z

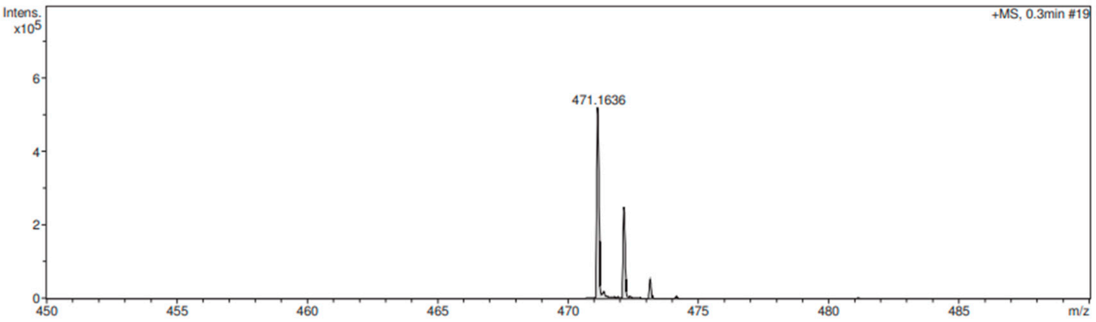

Meas. m/z	#	Ion Formula	m/z	err [ppm]	mSigma	# Sigma	Score	rdb	e⁻ Conf	N-Rule
471.1636	1	C29H29O2P2	471.1637	-0.3	85.2	1	100.00	16.5	even	ok
	2	C14H25N12O3P2	471.1642	-1.4	168.5	2	0.50	9.5	even	ok
	3	C13H29N8O7P2	471.1629	-1.4	181.1	3	0.20	4.5	even	ok

4-(di-p-tolyphosphoryl)-2-phenyl-1,3,4-trihydroisophosphinoline 2-oxide ISOP-pMea

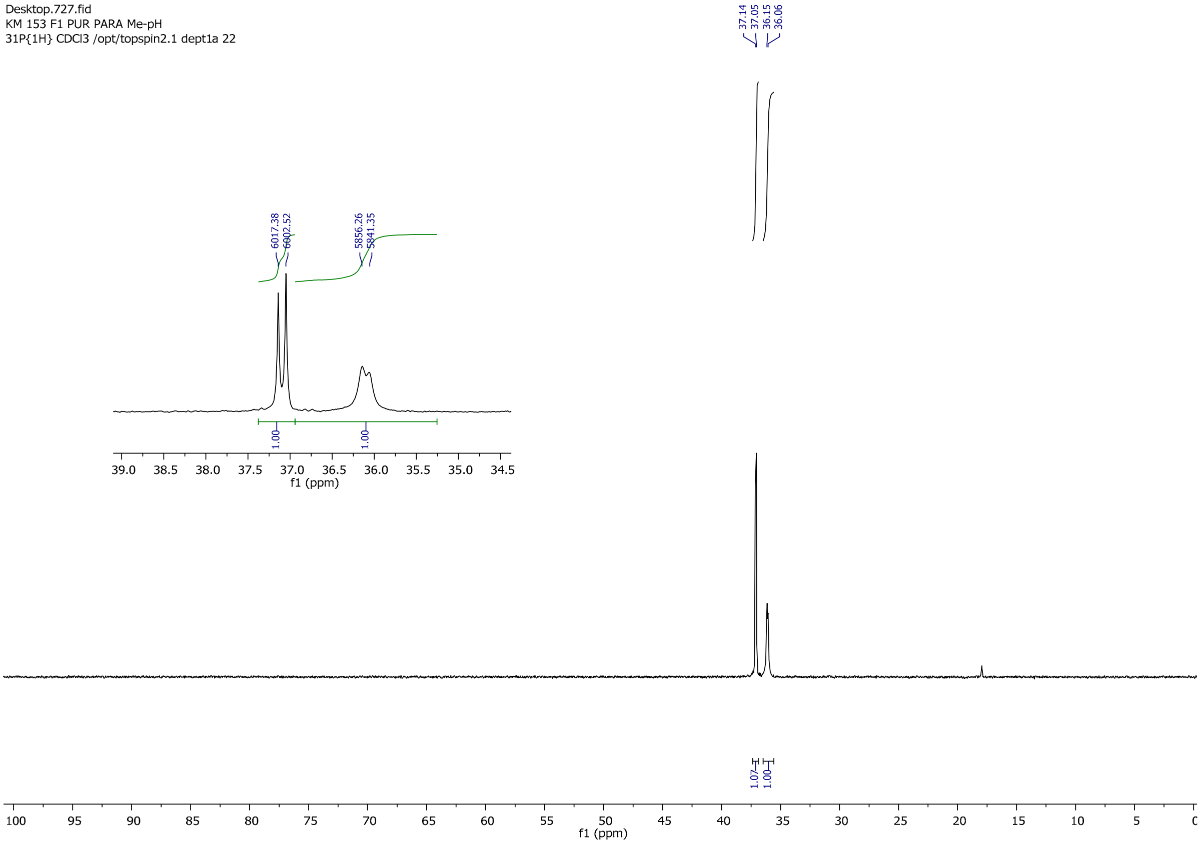

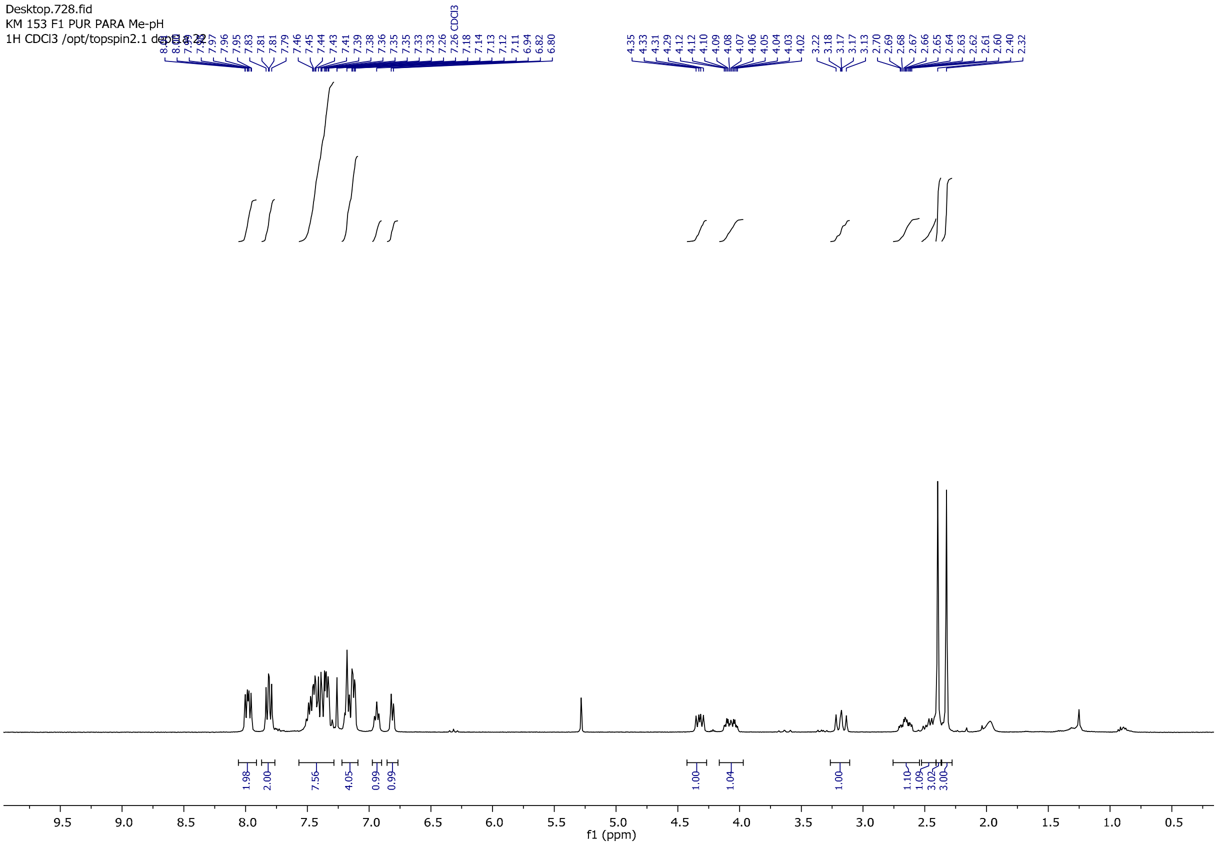

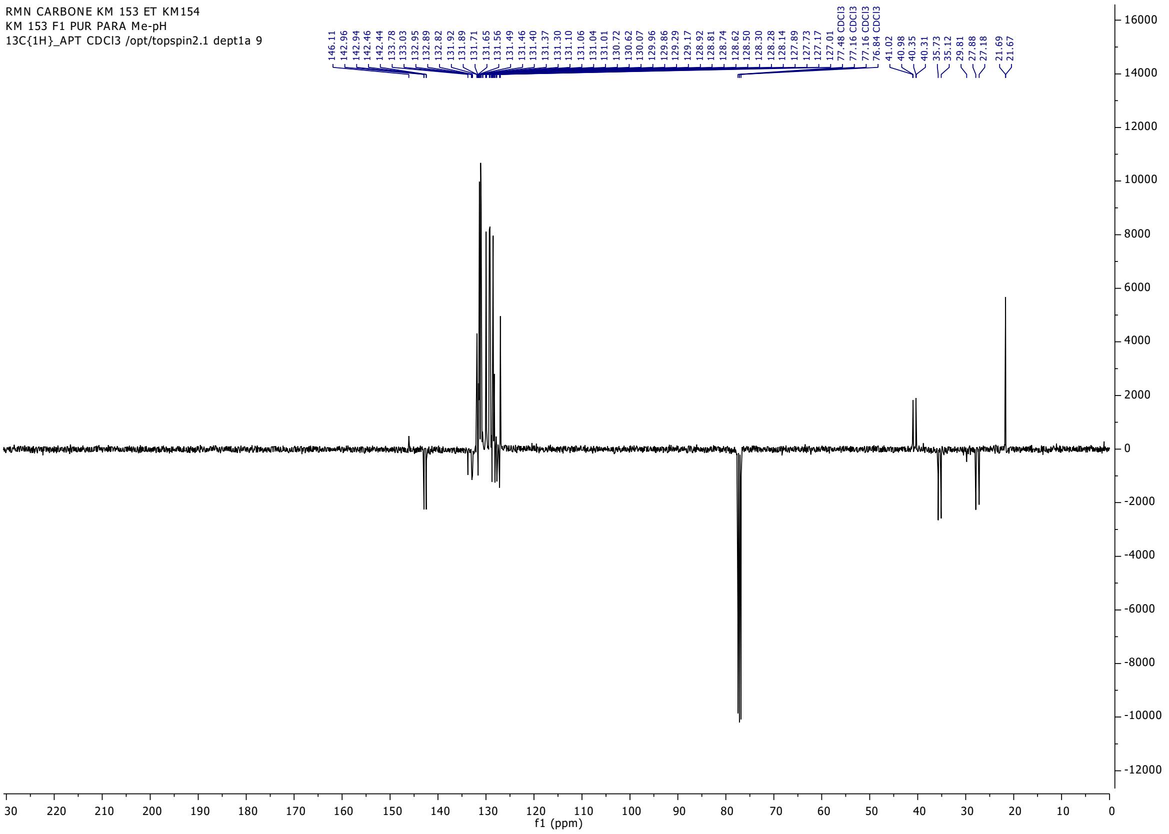

High Resolution Mass Result

Analysis Info

Sample Name **MMM-1 g _ KM153 f1**

Acquisition Date 7/26/2022 11:48:34 AM

Instrument / Ser# micrOTOF-Q 228888.10300

Acquisition Parameter

Source Type ESI | Ion Polarity Positive | Scan Begin 50 m/z | Scan End 2200 m/z

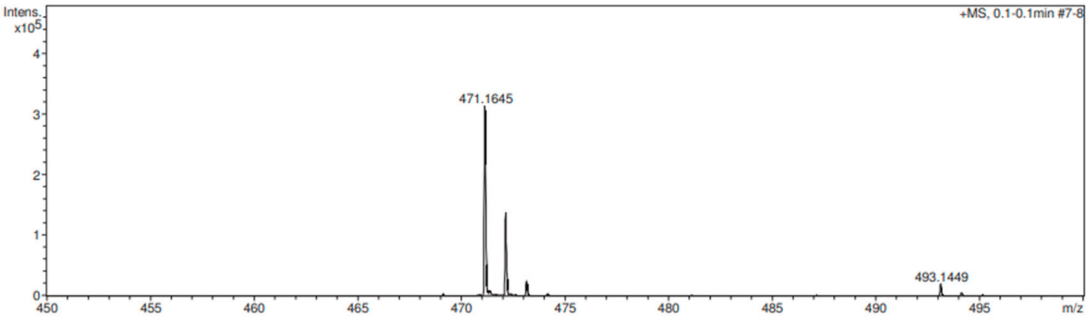

Meas. m/z	#	Ion Formula	m/z	err [ppm]	mSigma	# Sigma	Score	rdb	e⁻ Conf	N-Rule
471.1645	1	C29H29O2P2	471.1637	1.6	61.1	1	100.00	16.5	even	ok
	2	C14H25N12O3P2	471.1642	-0.5	140.8	2	2.10	9.5	even	ok

4-(di-p-tolyphosphoryl)-2-phenyl-1,3,4-trihydroisophosphinoline 2-oxide ISOP-pMea, + ISOP-pMea'

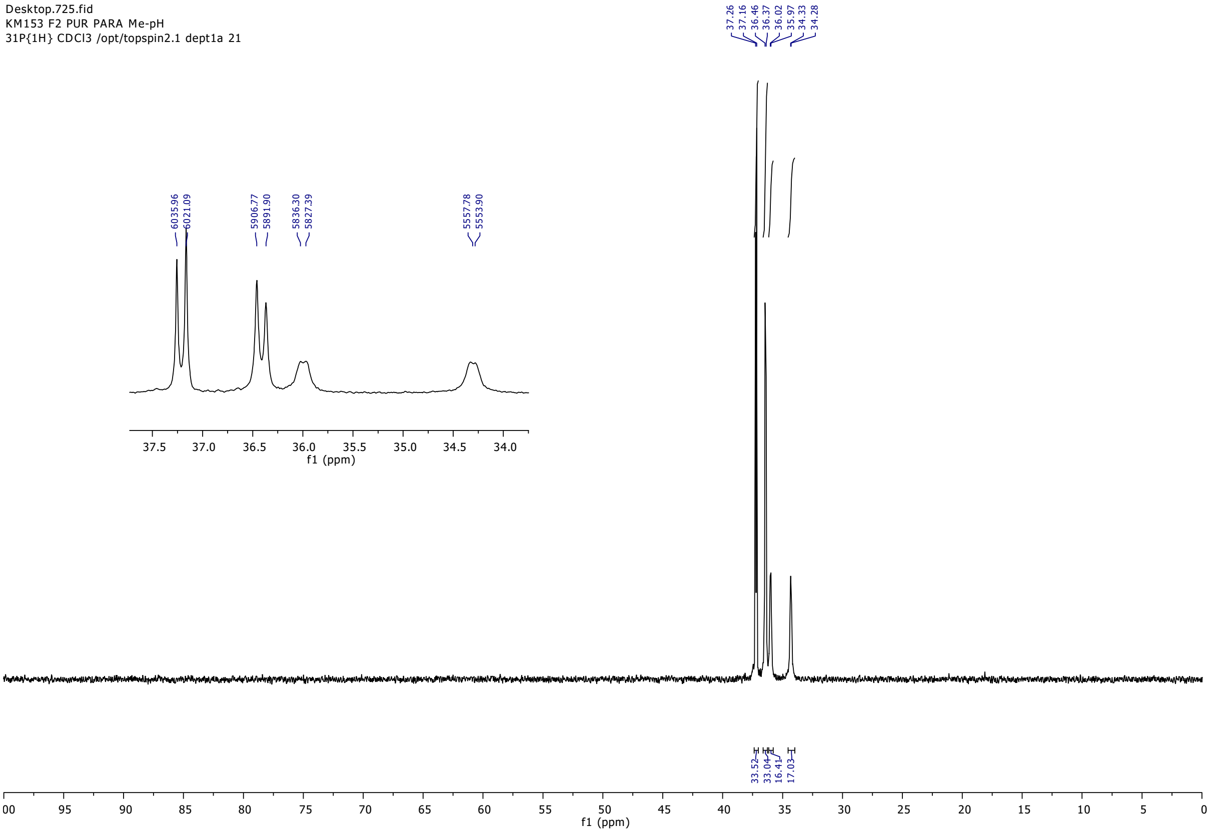

High Resolution Mass Result

Analysis Info

Sample Name **MMM-1 h _ KM153 f2**

Acquisition Date 7/26/2022 11:51:37 AM

Instrument / Ser# micrOTOF-Q 228888.10300

Acquisition Parameter

Source Type ESI | Ion Polarity Positive | Scan Begin 50 m/z | Scan End 2200 m/z

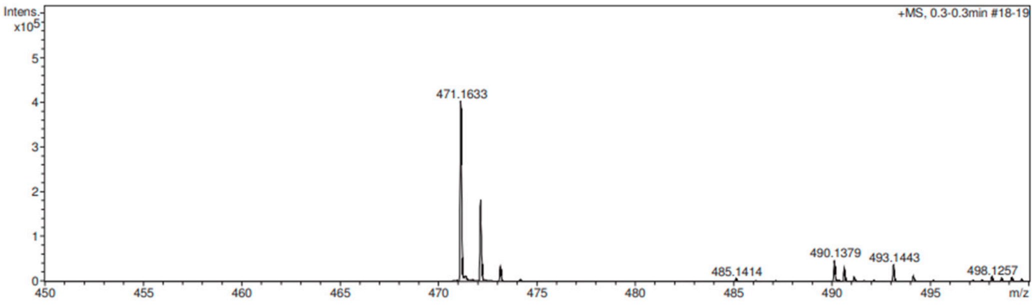

Meas. m/z	#	Ion Formula	m/z	err [ppm]	mSigma	# Sigma	Score	rdb	e⁻ Conf	N-Rule
471.1633	1	C29H29O2P2	471.1637	0.9	67.9	1	100.00	16.5	even	ok
	2	C14H25N12O3P2	471.1642	1.9	148.7	2	0.98	9.5	even	ok
	3	C13H29N8O7P2	471.1629	-0.9	161.4	3	0.53	4.5	even	ok

(Bis(2-methoxyphenyl) phosphoryl)-2-phenyl-1,3,4-trihydroisophosphinoline 2-oxide ISOP-mOMea

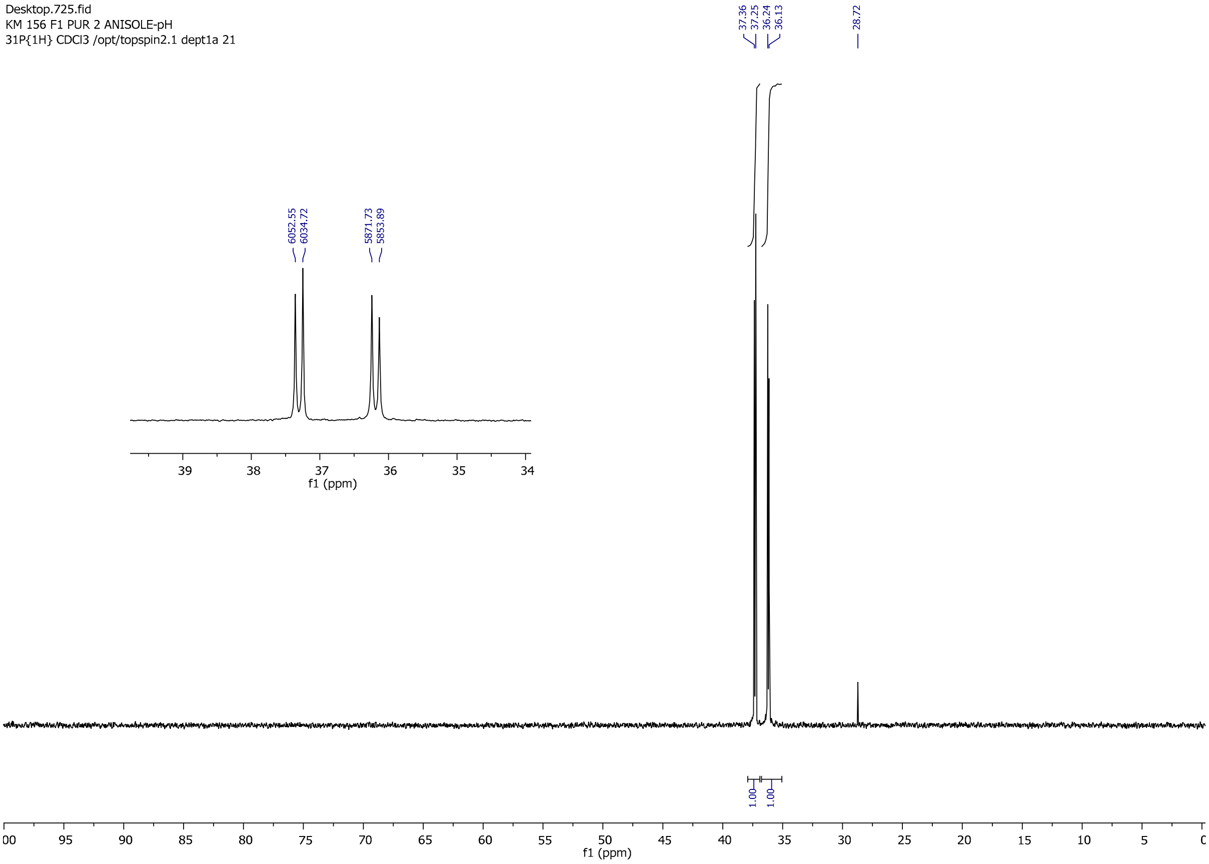

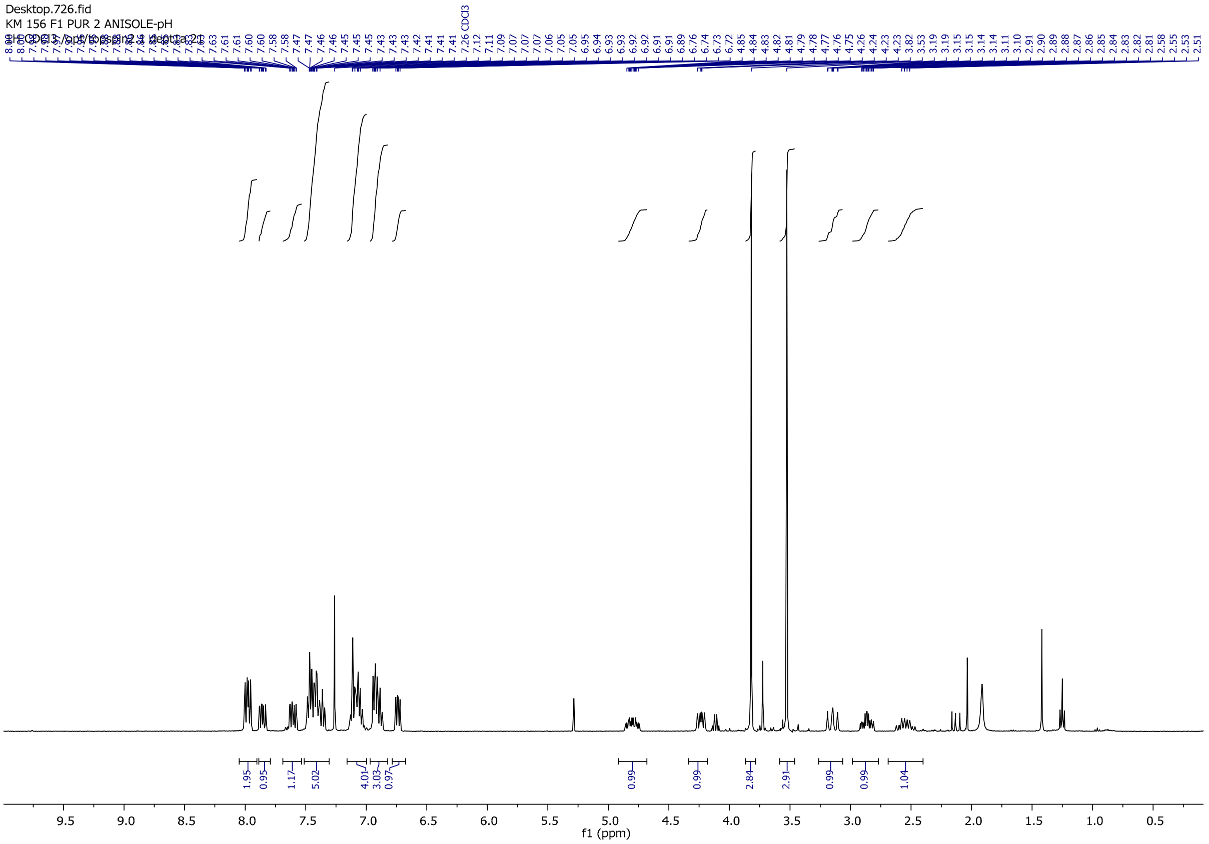

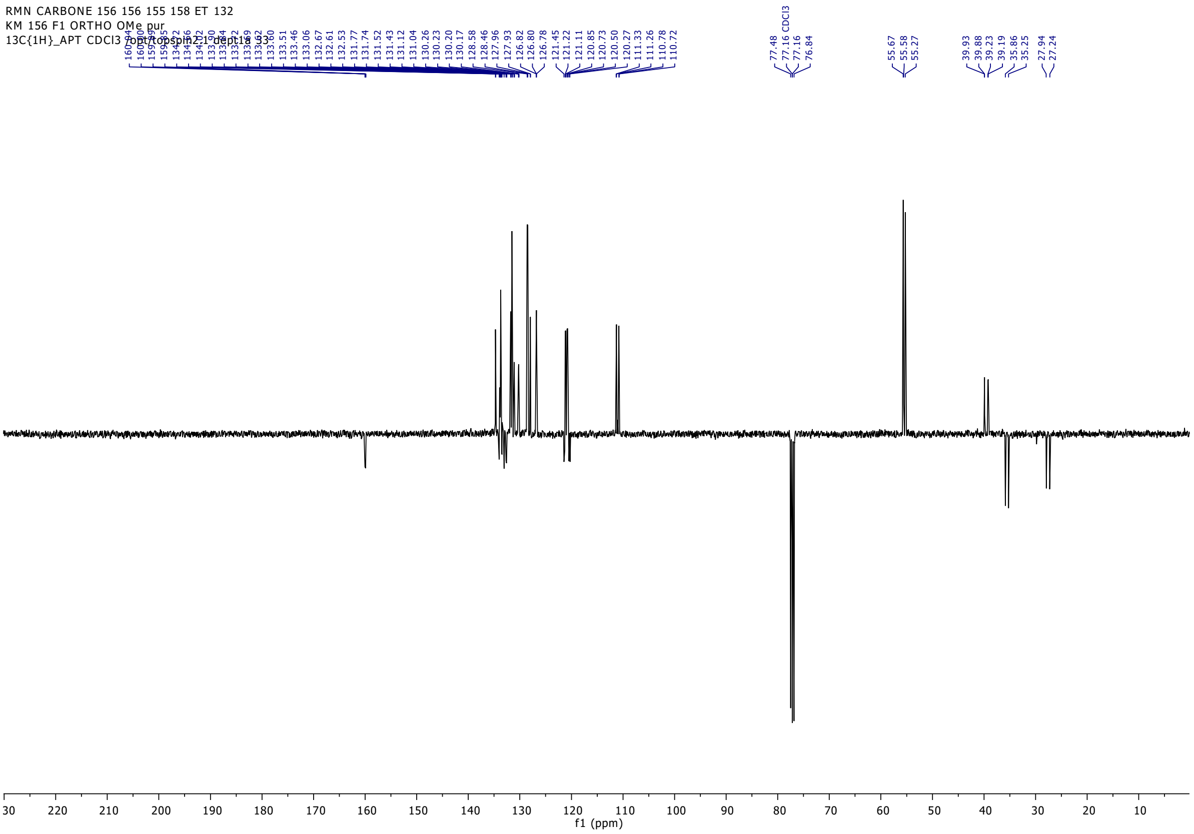

High Resolution Mass Result

Analysis Info

Sample Name **MMM-1 i _ KM156 f1**

Acquisition Date 7/26/2022 11:54:39 AM

Instrument / Ser# micrOTOF-Q 228888.10300

Acquisition Parameter

Source Type ESI | Ion Polarity Positive | Scan Begin 50 m/z | Scan End 2200 m/z

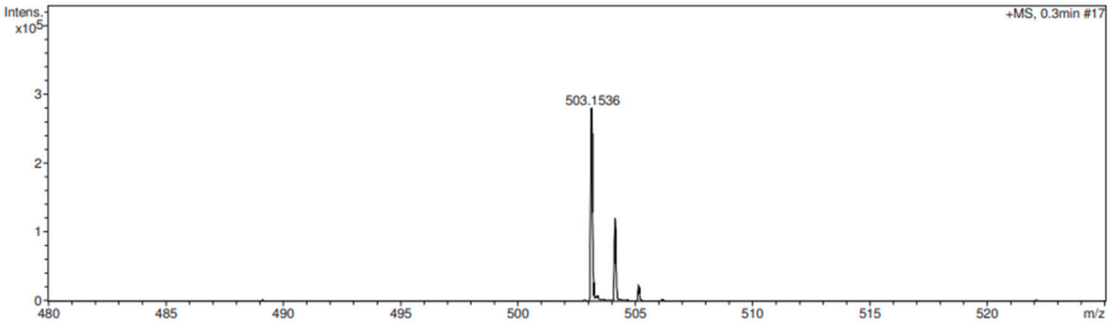

Meas. m/z	#	Ion Formula	m/z	err [ppm]	mSigma	# Sigma	Score	rdb	e⁻ Conf	N-Rule
503.1536	1	C29H29O4P2	503.1536	0.2	56.2	1	100.00	16.5	even	ok

(Bis(2-methoxyphenyl) phosphoryl)-2-phenyl-1,3,4-trihydroisophosphinoline 2-oxide ISOP-mOMea'

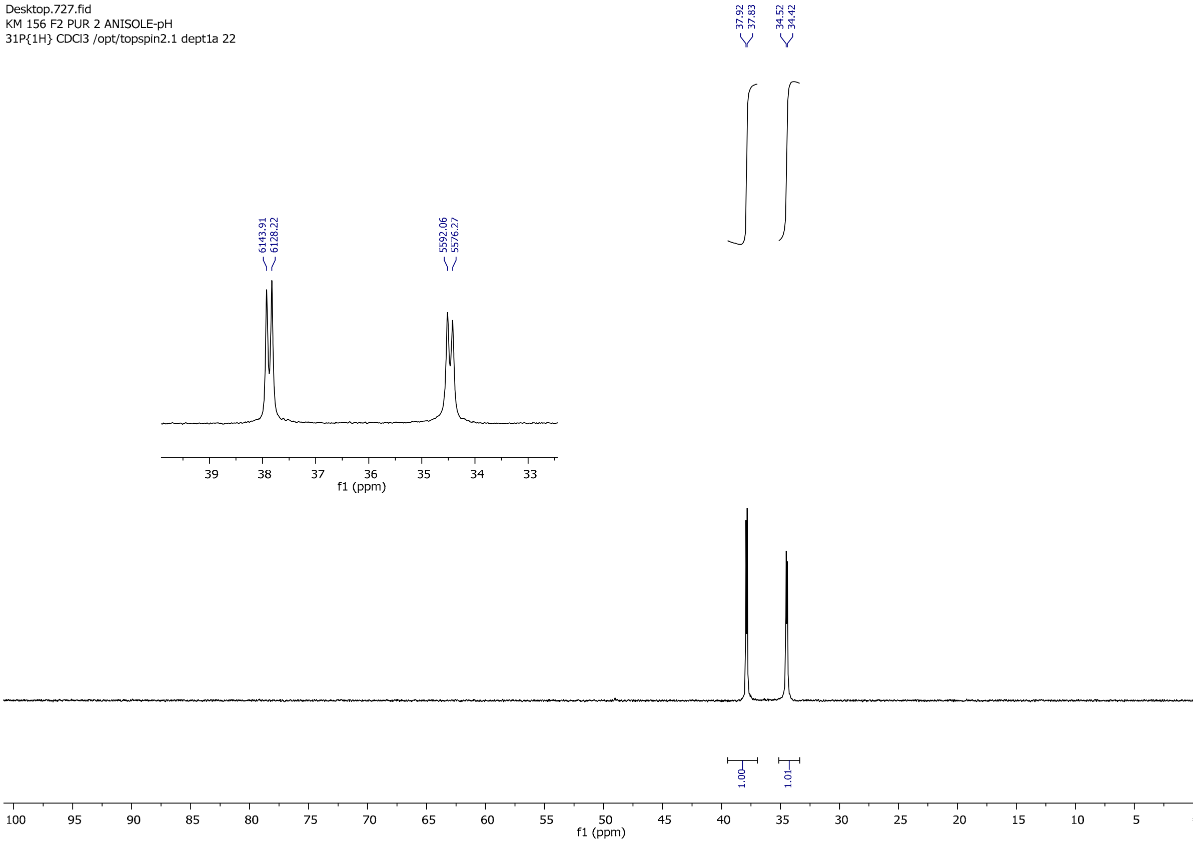

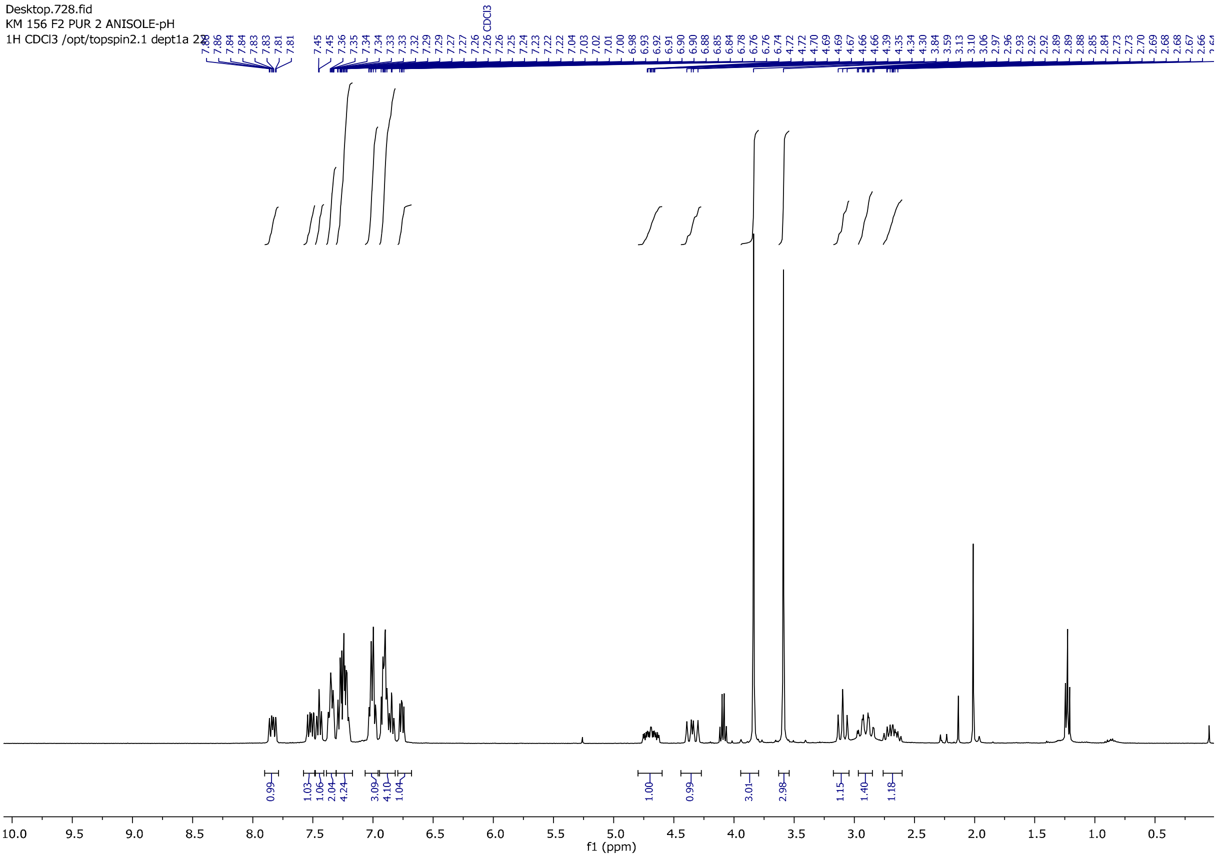

RMN CARBONE 156 156 155 158 ET 132
KM 156 F2 OHTHO OMe pur
13C{1H}_APT CDCl3 /opt/topspin2.1 dept18 34

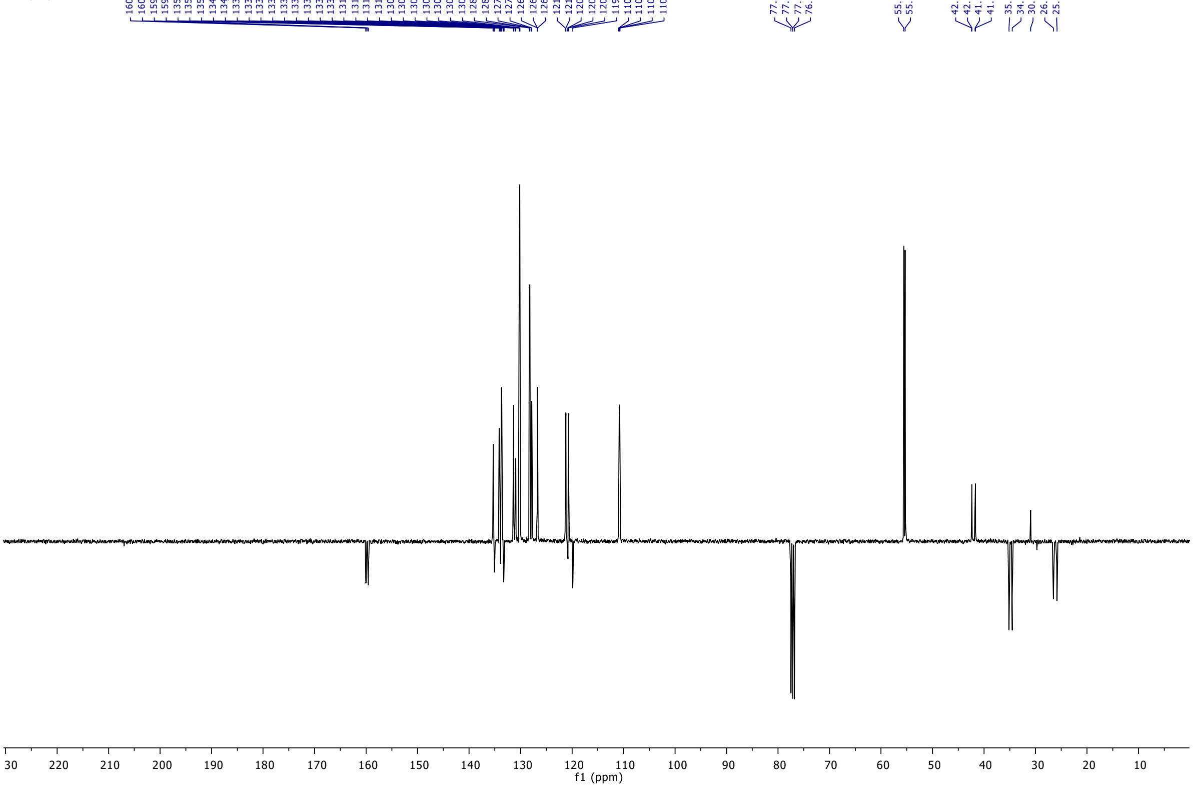

High Resolution Mass Result

**Analysis Info**		Acquisition Date	7/26/2022 11:57:43 AM	
Sample Name	**MMM-1 j _ KM156 f2**	Instrument / Ser#	micrOTOF-Q	228888.10300

Acquisition Parameter

Source Type	ESI	Ion Polarity	Positive	Scan Begin	50 m/z	Scan End	2200 m/z

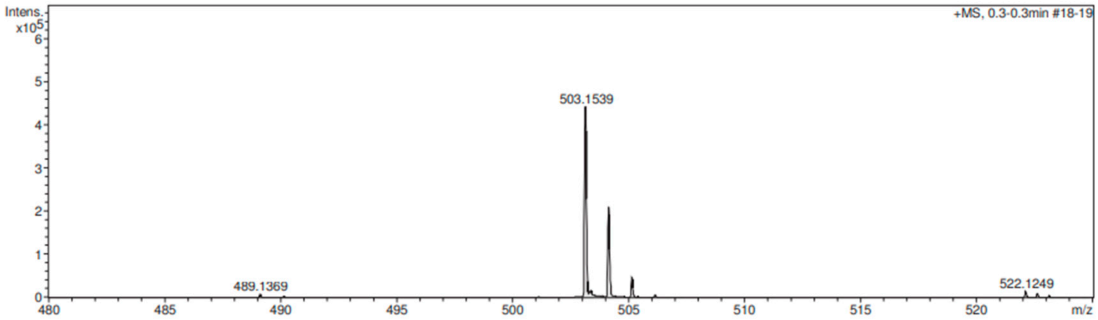

Meas. m/z	#	Ion Formula	m/z	err [ppm]	mSigma	# Sigma	Score	rdb	e⁻ Conf	N-Rule
503.1539	1	C30H25N4P2	503.1549	1.9	71.5	1	100.00	21.5	even	ok
	2	C29H29O4P2	503.1536	0.8	82.5	2	86.85	16.5	even	ok
	3	C14H25N12O5P2	503.1541	-0.2	165.3	3	0.70	9.5	even	ok

(Bis (3-methoxyphenyl) phosphoryl)-2-phenyl-1,3,4-trihydroisophosphinoline 2-oxide ISOP-mOMea

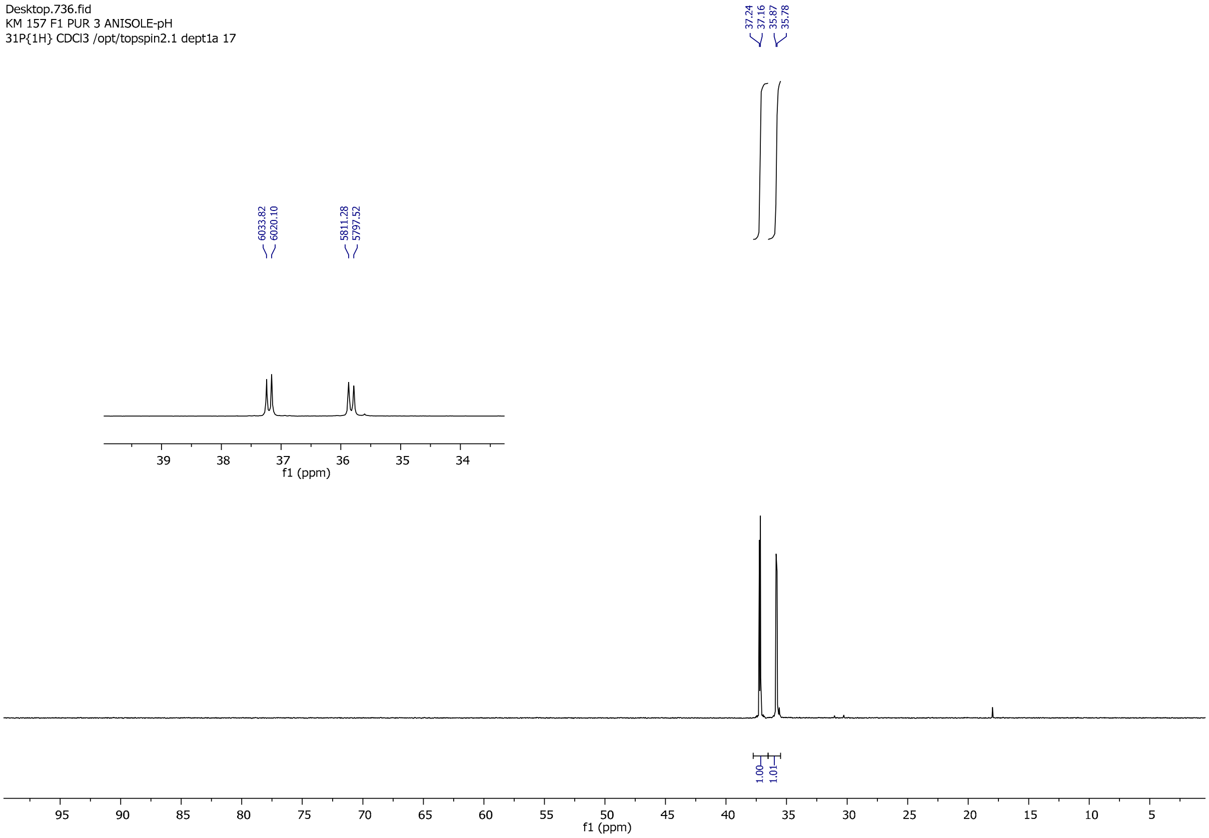

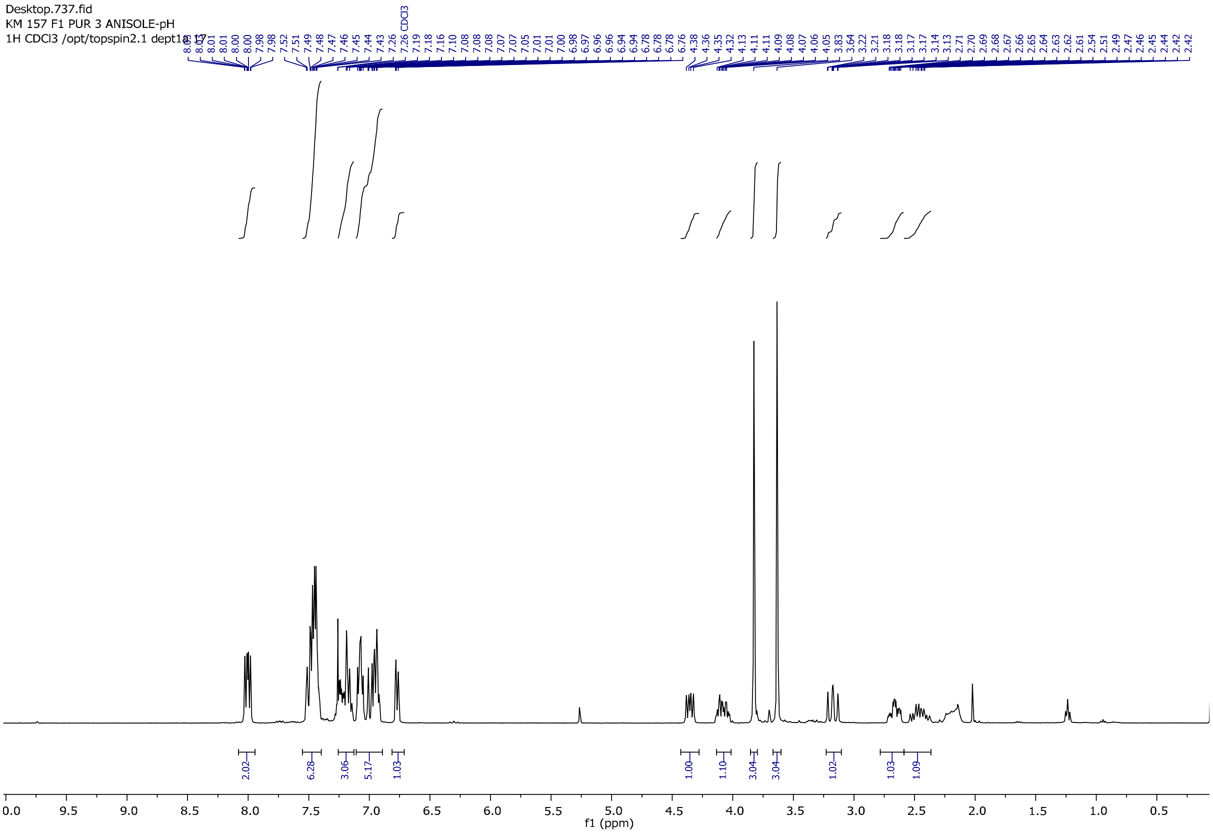

RMN CARBONE 156 156 155 158 ET 132
KM157 F1 META OMe pur
13C{1H}_APT CDCl3 /opt/topspin2.1.dept1a 31

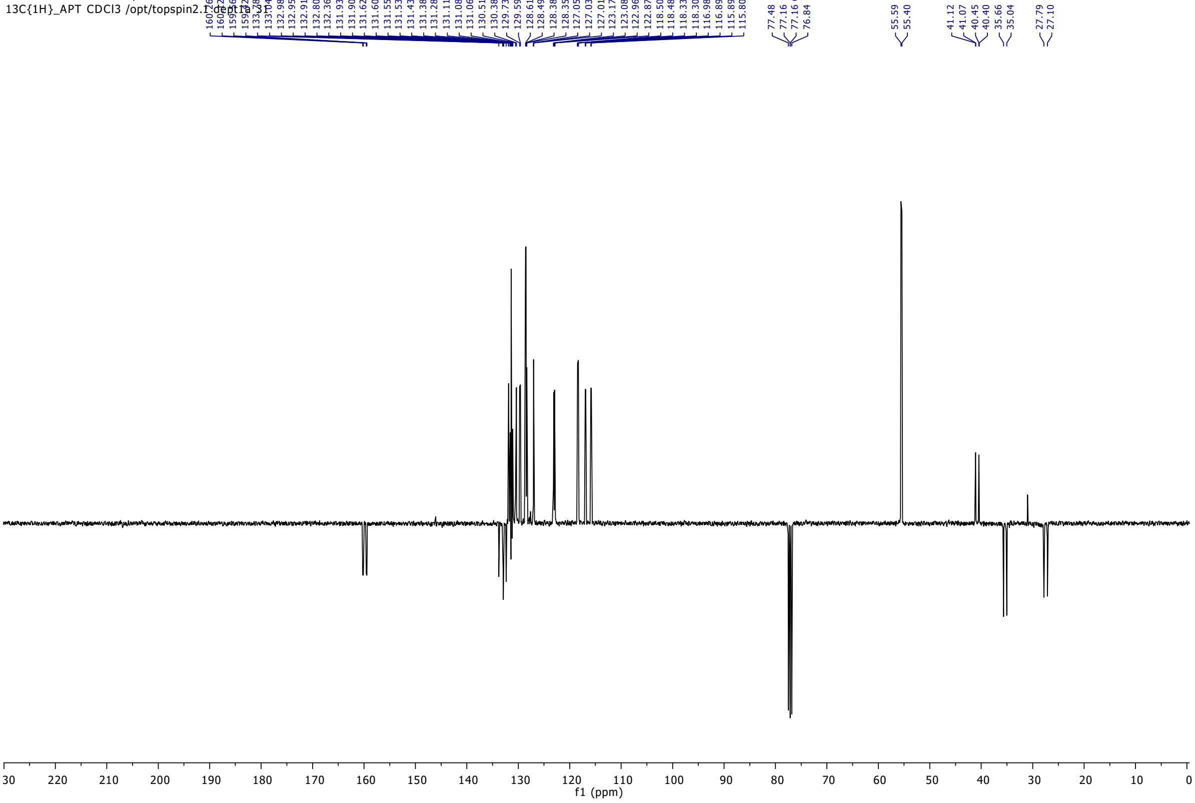

High Resolution Mass Result

Analysis Info

Sample Name **MMM-1 k _ KM157 f1**

Acquisition Date 7/26/2022 12:00:45 PM

Instrument / Ser# micrOTOF-Q 228888.10300

Acquisition Parameter

Source Type ESI | Ion Polarity Positive | Scan Begin 50 m/z | Scan End 2200 m/z

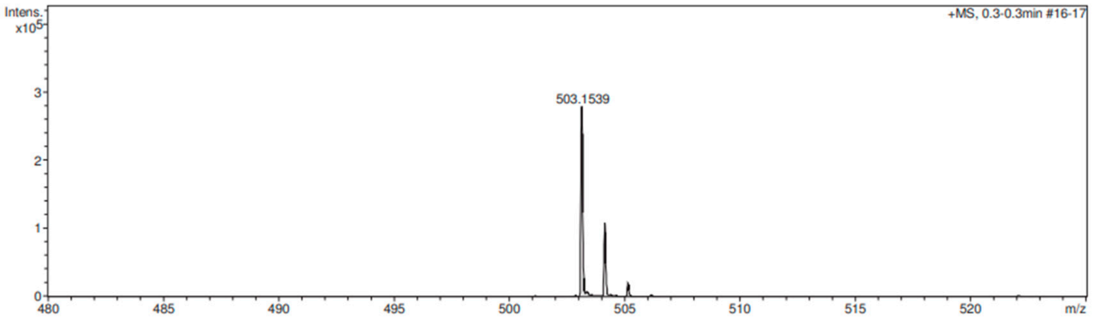

Meas. m/z	#	Ion Formula	m/z	err [ppm]	mSigma	# Sigma	Score	rdb	e⁻ Conf	N-Rule
503.1539	1	C30H25N4P2	503.1549	2.0	25.3	1	91.64	21.5	even	ok
	2	C29H29O4P2	503.1536	-0.6	36.5	2	100.00	16.5	even	ok

(Bis (3-methoxyphenyl) phosphoryl)-2-phenyl-1,3,4-trihydroisophosphinoline 2-oxide ISOP-mOMea'

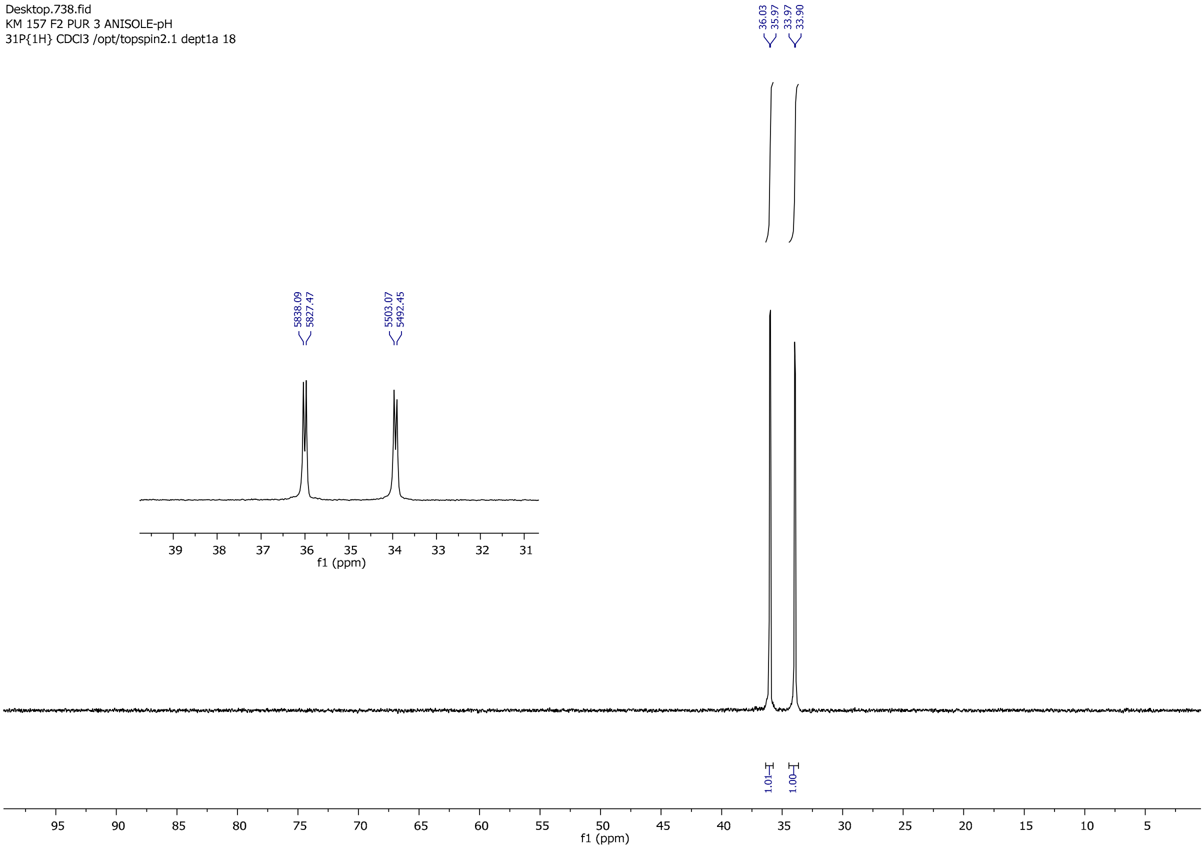

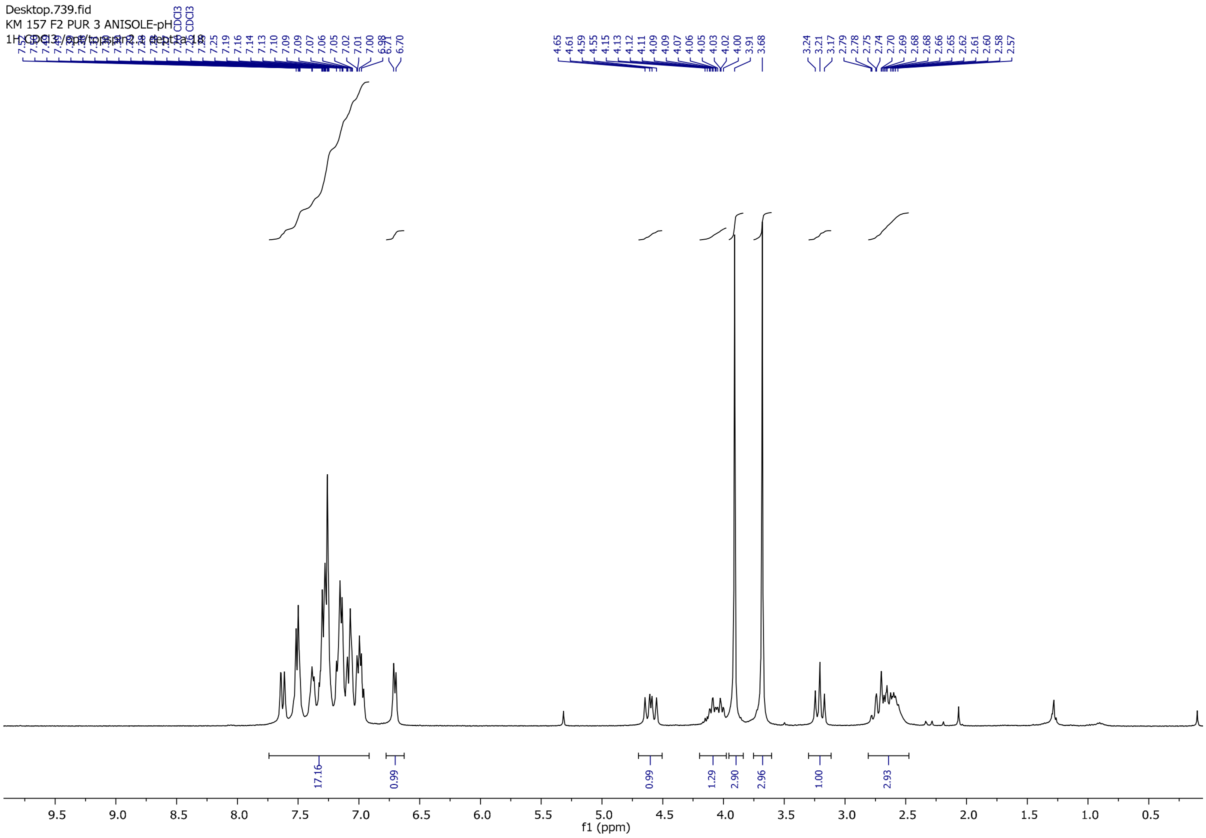

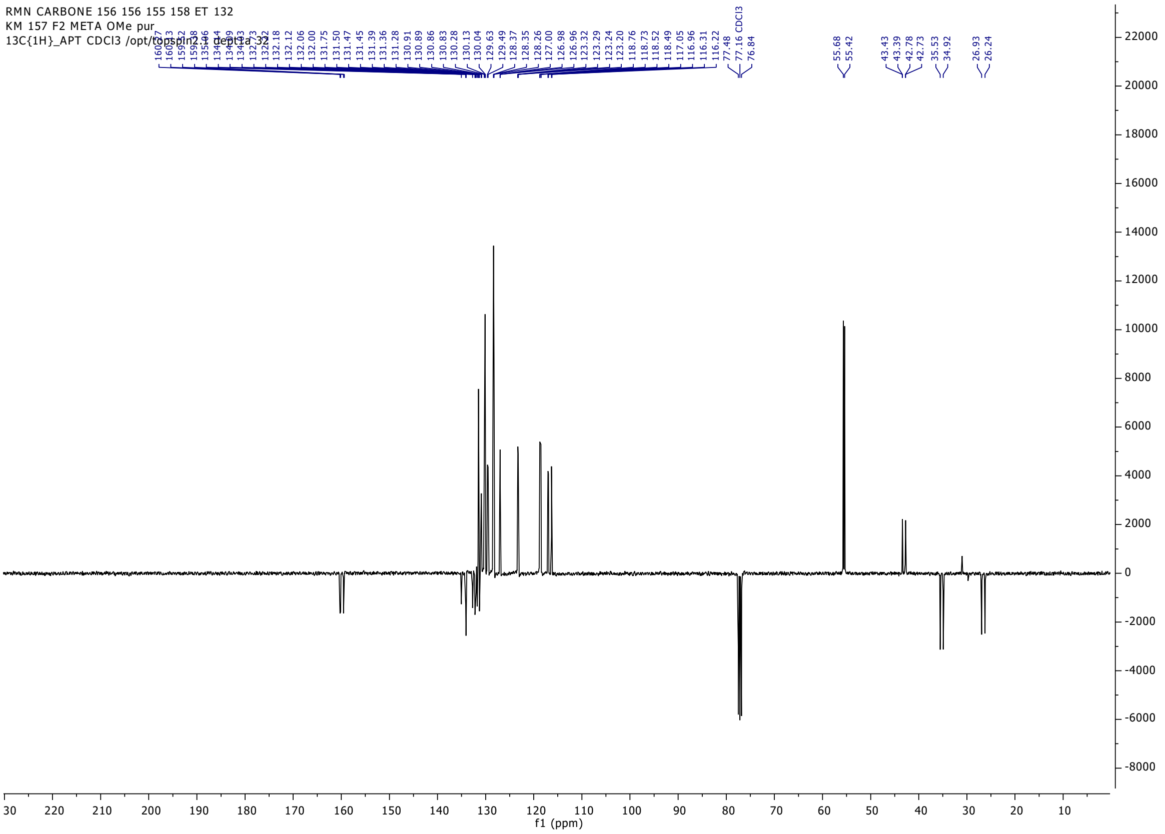

High Resolution Mass Result

Analysis Info

Sample Name	**MMM-1 I _ KM157 f2**	Acquisition Date	7/26/2022 12:03:48 PM
		Instrument / Ser#	micrOTOF-Q 228888.10300

Acquisition Parameter

Source Type	ESI	Ion Polarity	Positive	Scan Begin	50 m/z	Scan End	2200 m/z

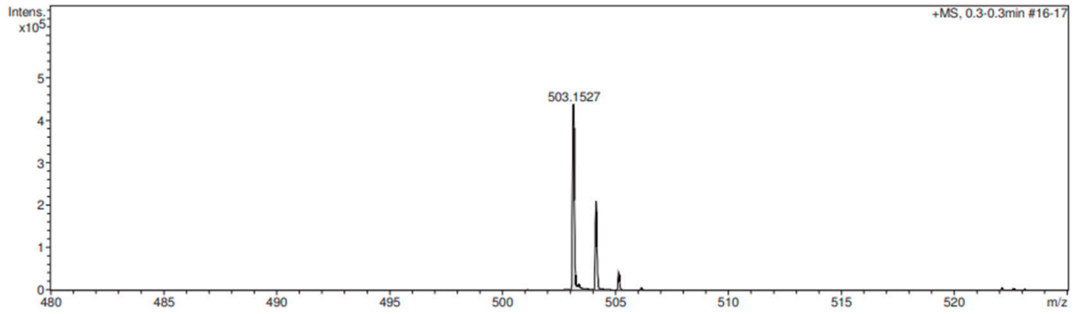

Meas. m/z	#	Ion Formula	m/z	err [ppm]	mSigma	# Sigma	Score	rdb	e⁻ Conf	N-Rule
503.1527	1	C29H29O4P2	503.1536	1.7	82.2	1	100.00	16.5	even	ok
	2	C13H29N8O9P2	503.1527	-0.0	154.0	2	3.05	4.5	even	ok

4-(bis (3-fluorophenyl) phosphoryl)-2-phenyl-1,3,4-trihydroisophosphinoline-2-oxide
Mixture ISOP-mFa + ISOP-mFa'

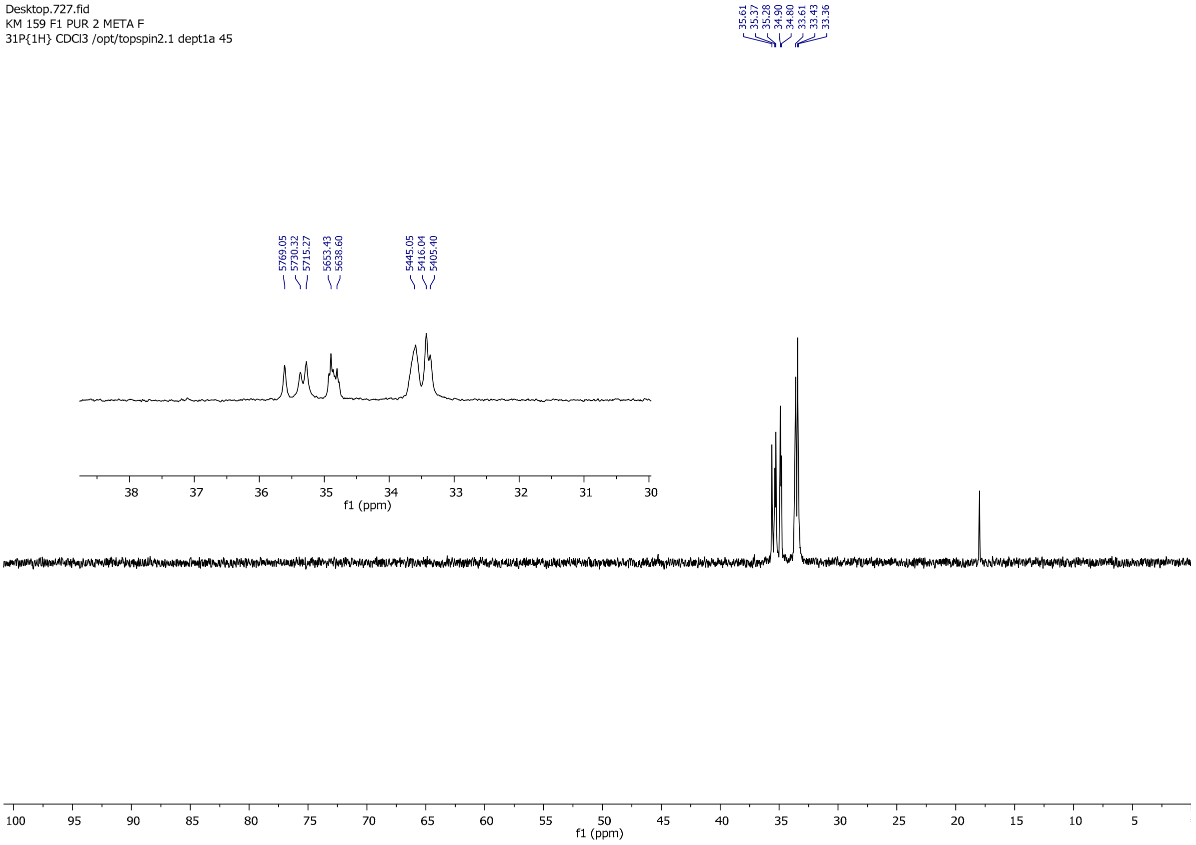

ISOP-mFa'

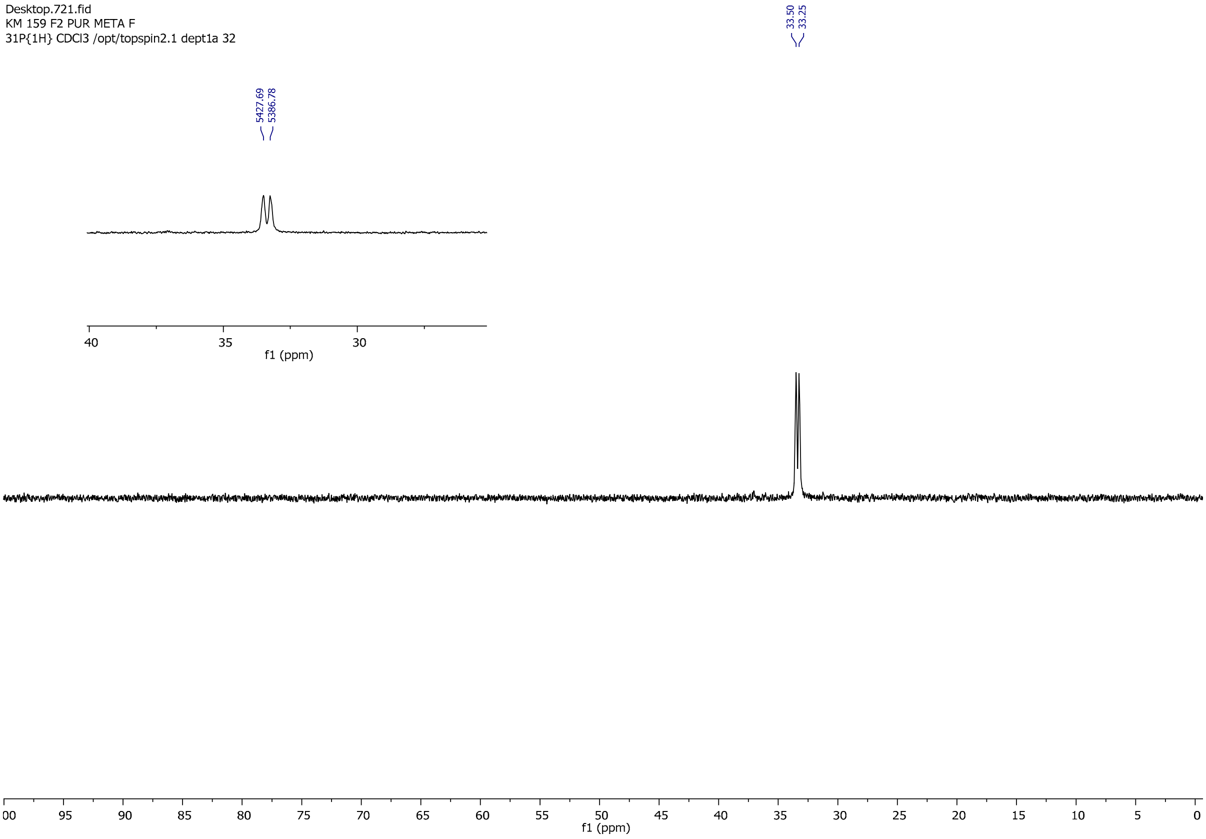

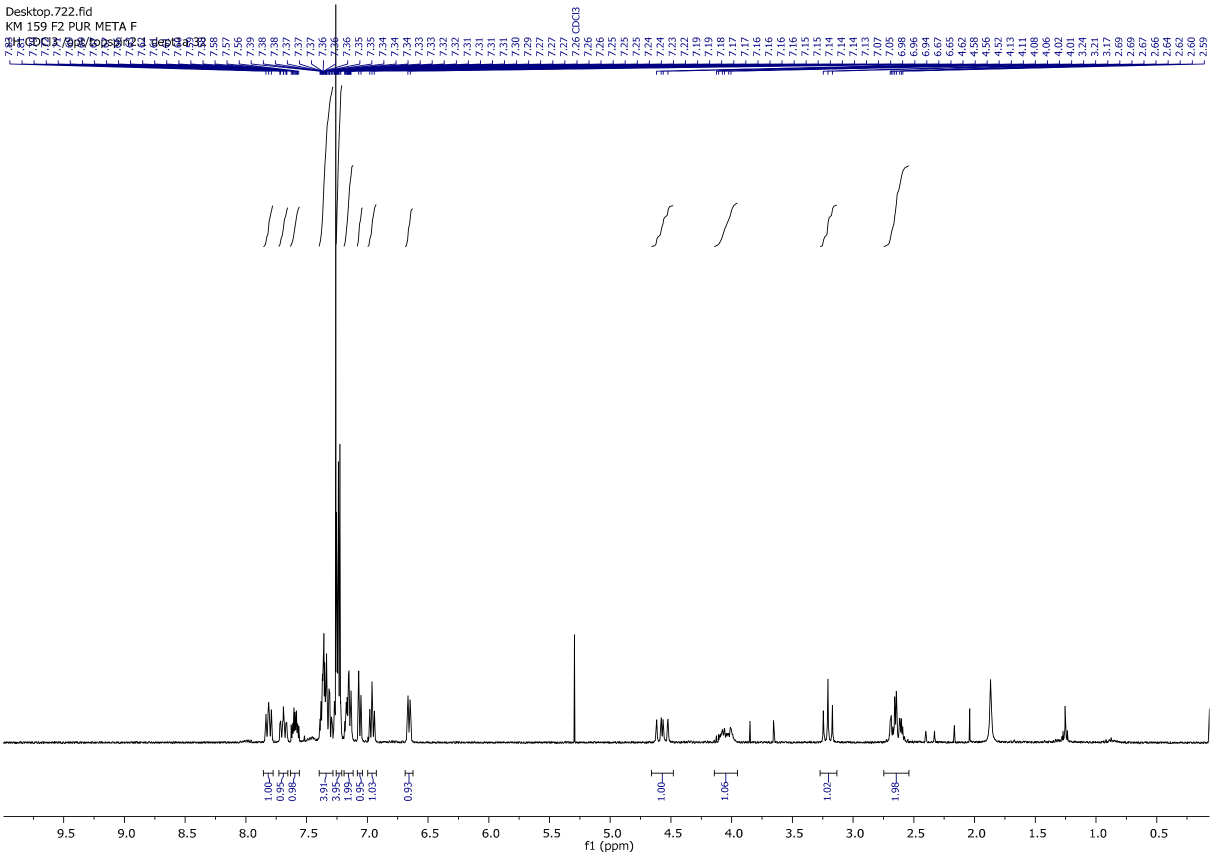
Desktop.722.fid
KM 159 F2 PUR META F
CDCl3
f1 (ppm)

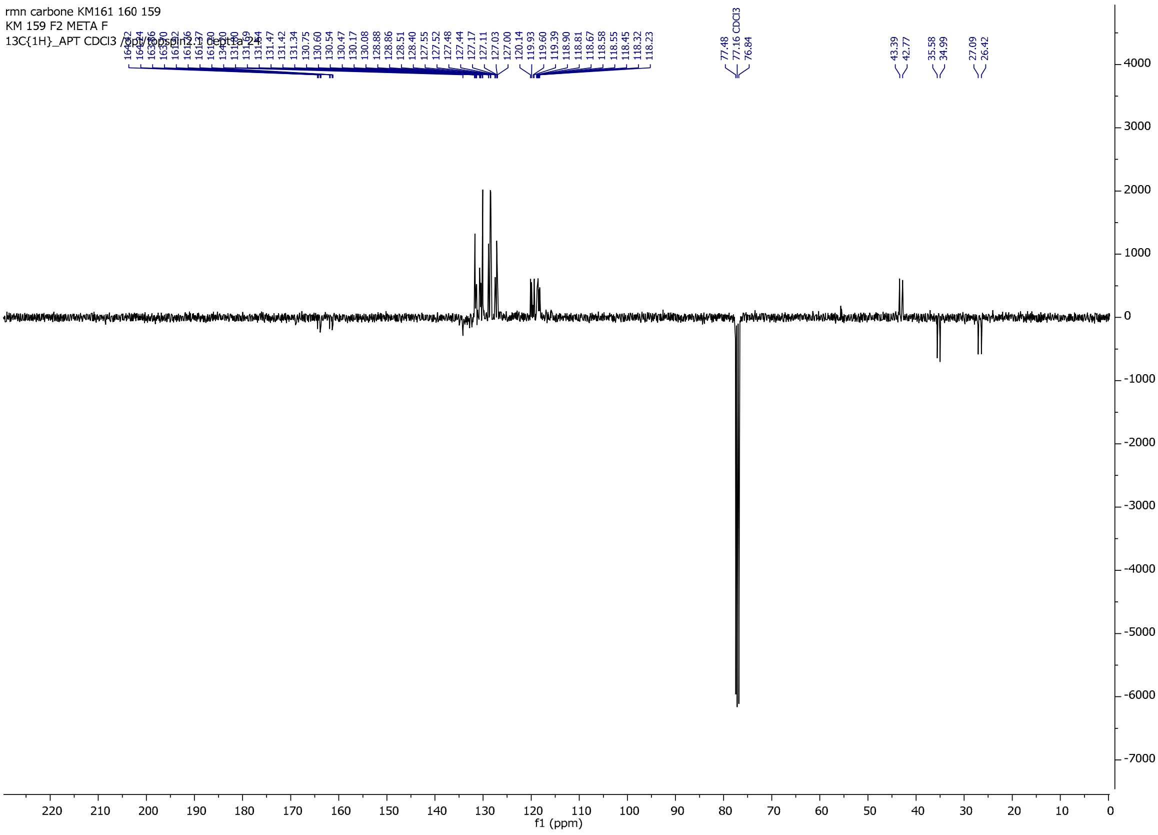
rmn carbone KM161 160 159
KM 159 F2 META F
77.48
77.16 CDCl3
76.84
43.39
42.77
35.58
34.99
27.09
26.42
f1 (ppm)

High Resolution Mass Result

Analysis Info

Sample Name **MMM-1 o _ KM159 f1**

Acquisition Date 7/26/2022 12:12:55 PM

Instrument / Ser# micrOTOF-Q 228888.10300

Acquisition Parameter

Source Type ESI Ion Polarity Positive Scan Begin 50 m/z Scan End 2200 m/z

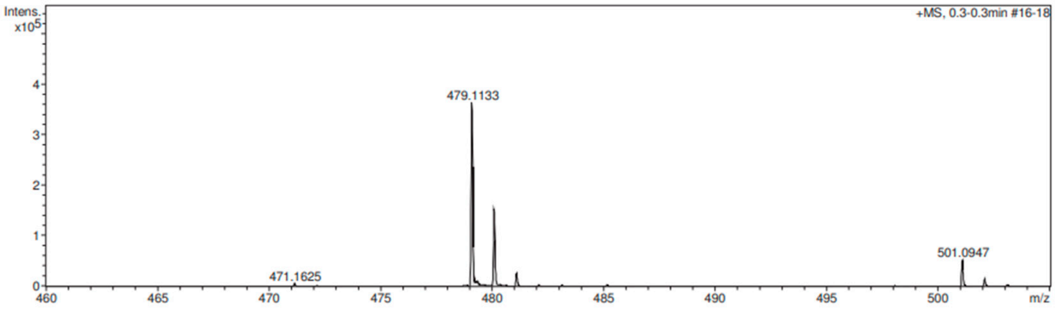

Meas. m/z	#	Ion Formula	m/z	err [ppm]	mSigma	# Sigma	Score	rdb	e⁻ Conf	N-Rule
479.1133	1	C27H23F2O2P2	479.1136	-0.5	65.4	1	100.00	16.5	even	ok

4-(bis (4-fluorophenyl) phosphoryl)-2-phenyl-1,3,4-trihydroisophosphinoline-2-oxide ISOP-pFa + ISOP-pFa'

Desktop.723.fid
KM 160 F2 PUR PARA F
31P{1H} CDCl3 /opt/topspin2.1 dept1a 43

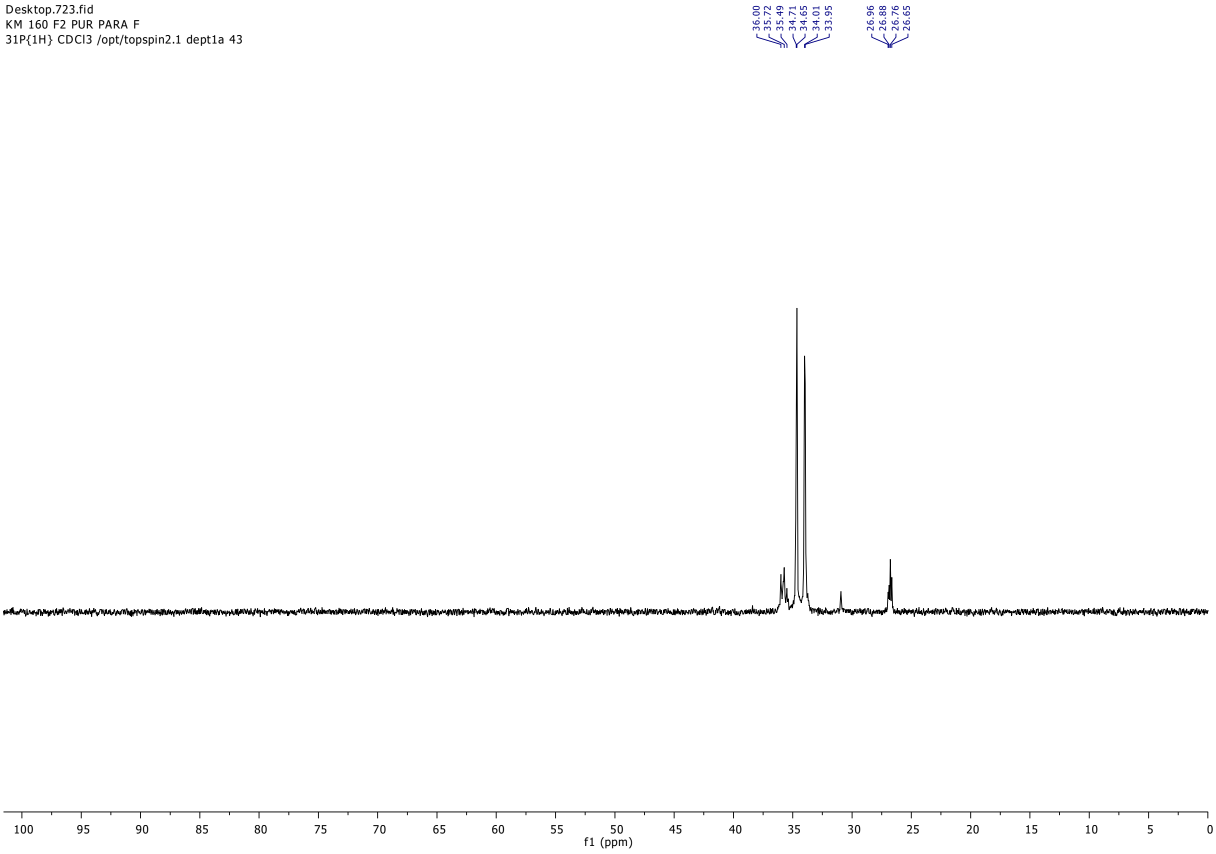

High Resolution Mass Result

Analysis Info

Sample Name **MMM-1 r _ KM160 f2**

Acquisition Date 7/26/2022 12:22:04 PM

Instrument / Ser# micrOTOF-Q 228888.10300

Acquisition Parameter

Source Type	ESI	Ion Polarity	Positive	Scan Begin	50 m/z	Scan End	2200 m/z

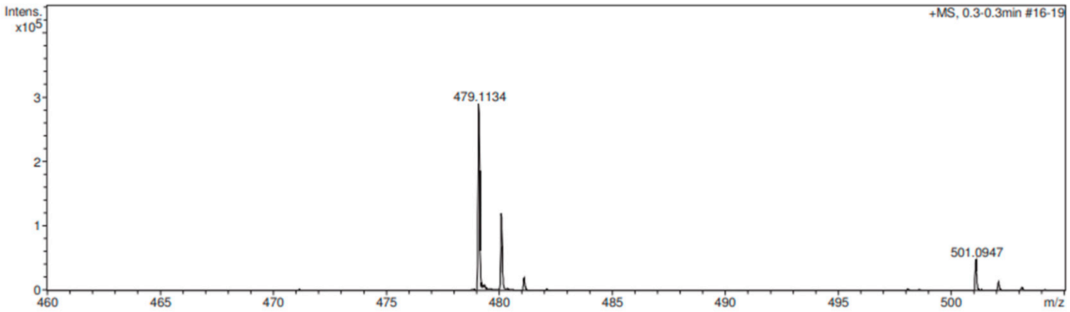

Meas. m/z	#	Ion Formula	m/z	err [ppm]	mSigma	# Sigma	Score	rdb	e⁻ Conf	N-Rule
479.1134	1	C27H23F2O2P2	479.1136	-0.3	59.8	1	100.00	16.5	even	ok

4-(bis (4-fluorophenyl) phosphoryl)-2-phenyl-1,3,4-trihydroisophosphinoline-2-oxide ISOP-pFa

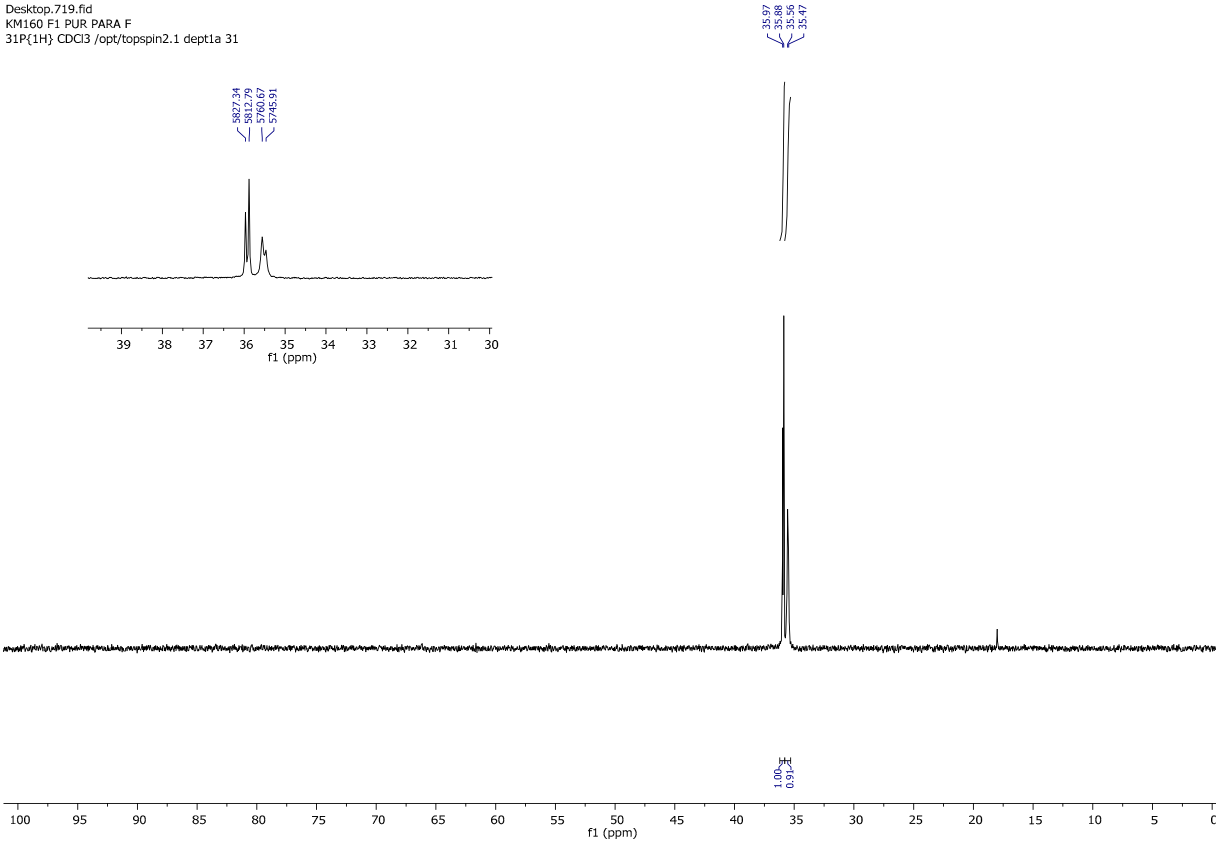

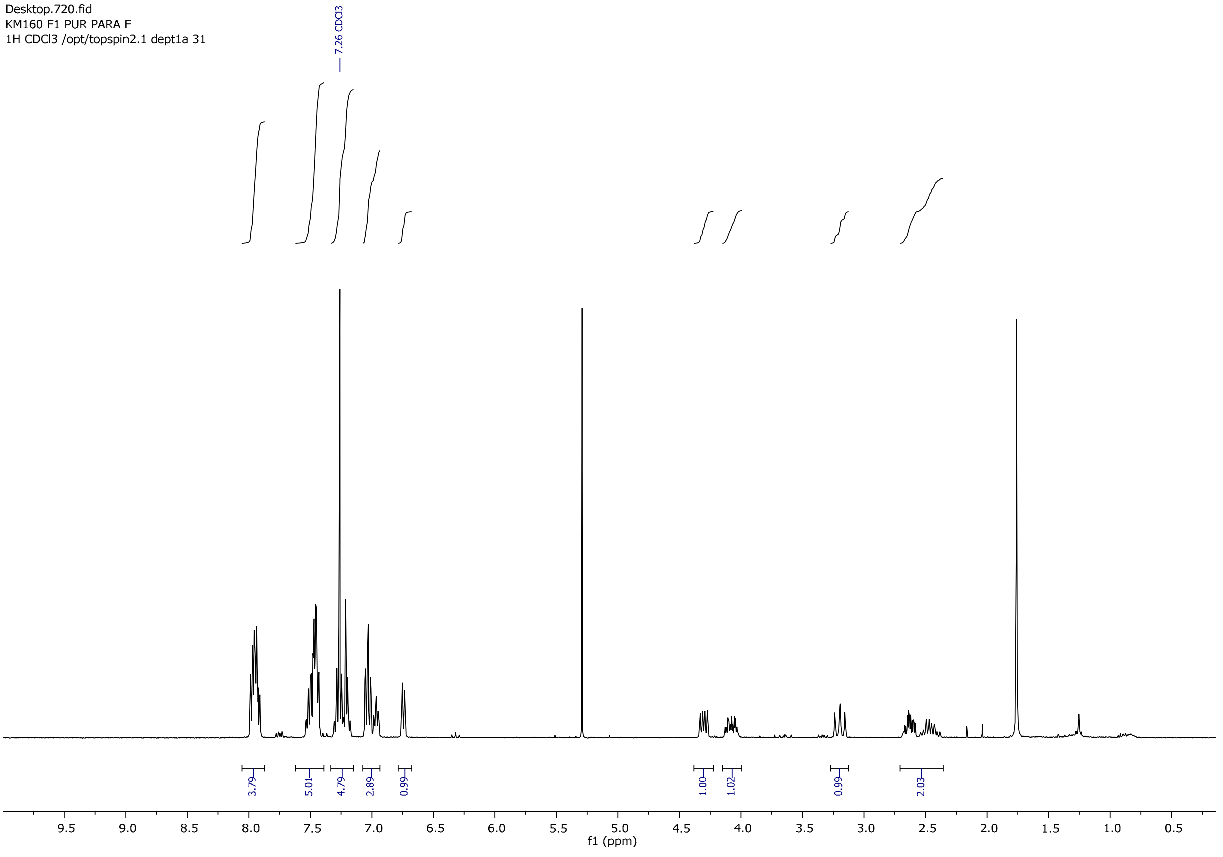

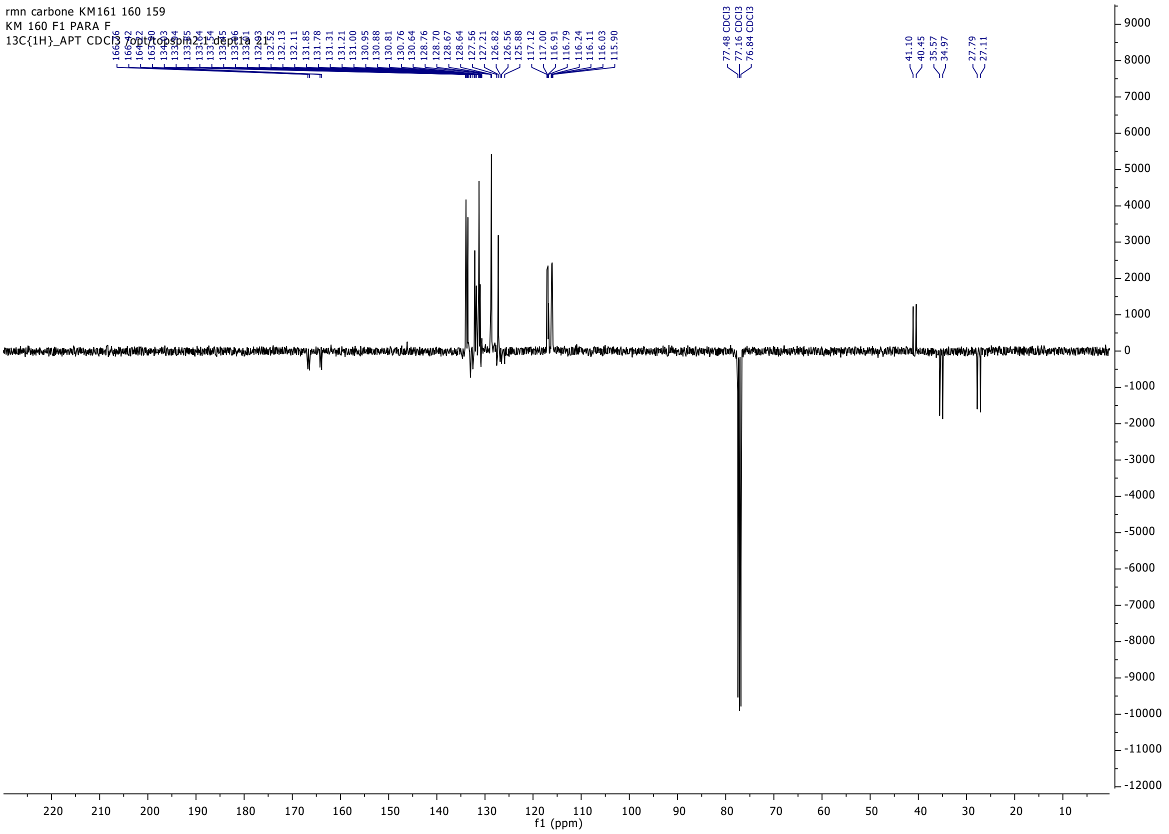

High Resolution Mass Result

Analysis Info

Sample Name	**MMM-1 q _ KM160 f1**	Acquisition Date	7/26/2022 12:19:00 PM
		Instrument / Ser#	micrOTOF-Q 228888.10300

Acquisition Parameter

Source Type	ESI	Ion Polarity	Positive	Scan Begin	50 m/z	Scan End	2200 m/z

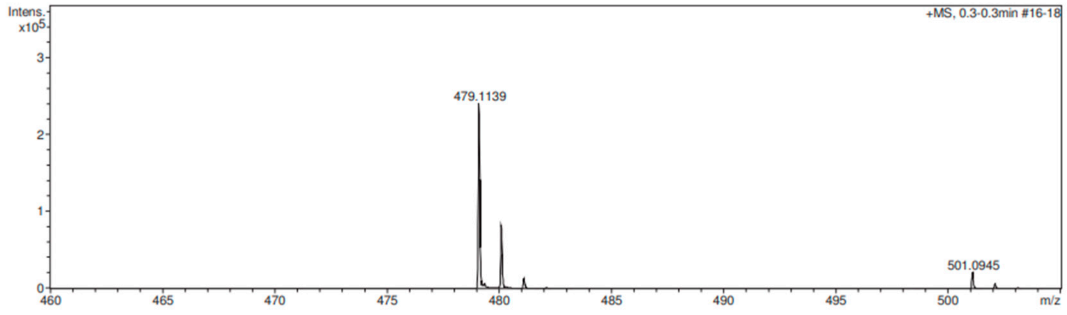

Meas. m/z	#	Ion Formula	m/z	err [ppm]	mSigma	# Sigma	Score	rdb	e⁻ Conf	N-Rule
479.1139	1	C27H23F2O2P2	479.1136	-0.7	24.8	1	100.00	16.5	even	ok

4-(bis(4-chloroxyphenyl) phosphoryl)-2-phenyl-1,3,4-trihydroisophosphinoline 2-oxide ISOP-pCla

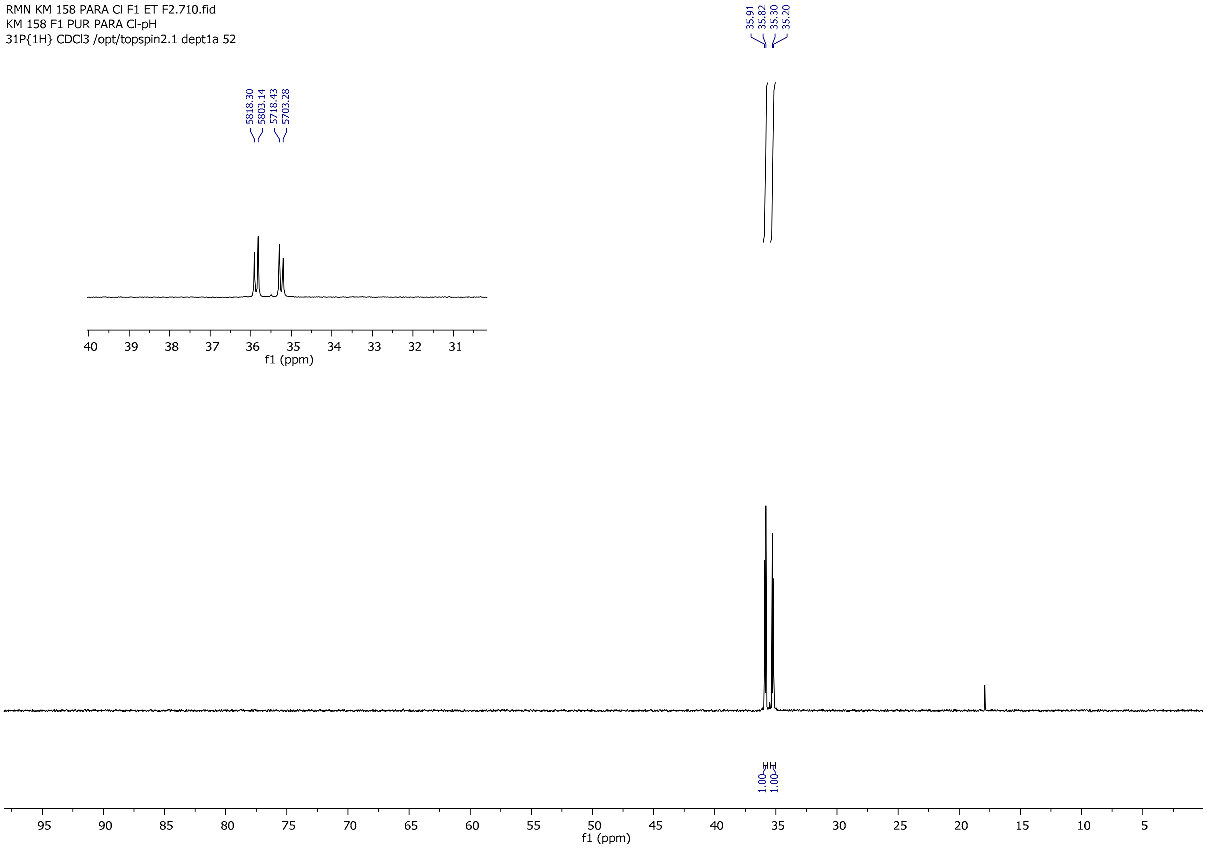

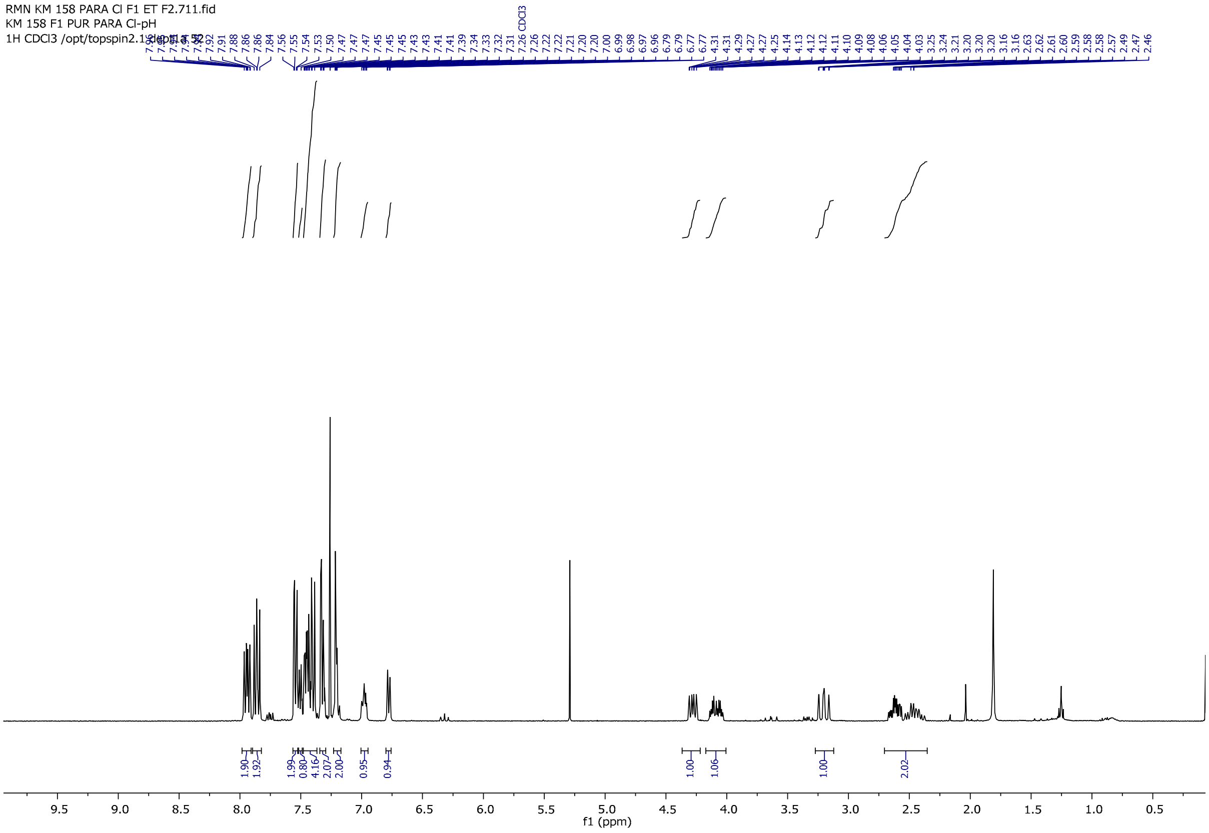

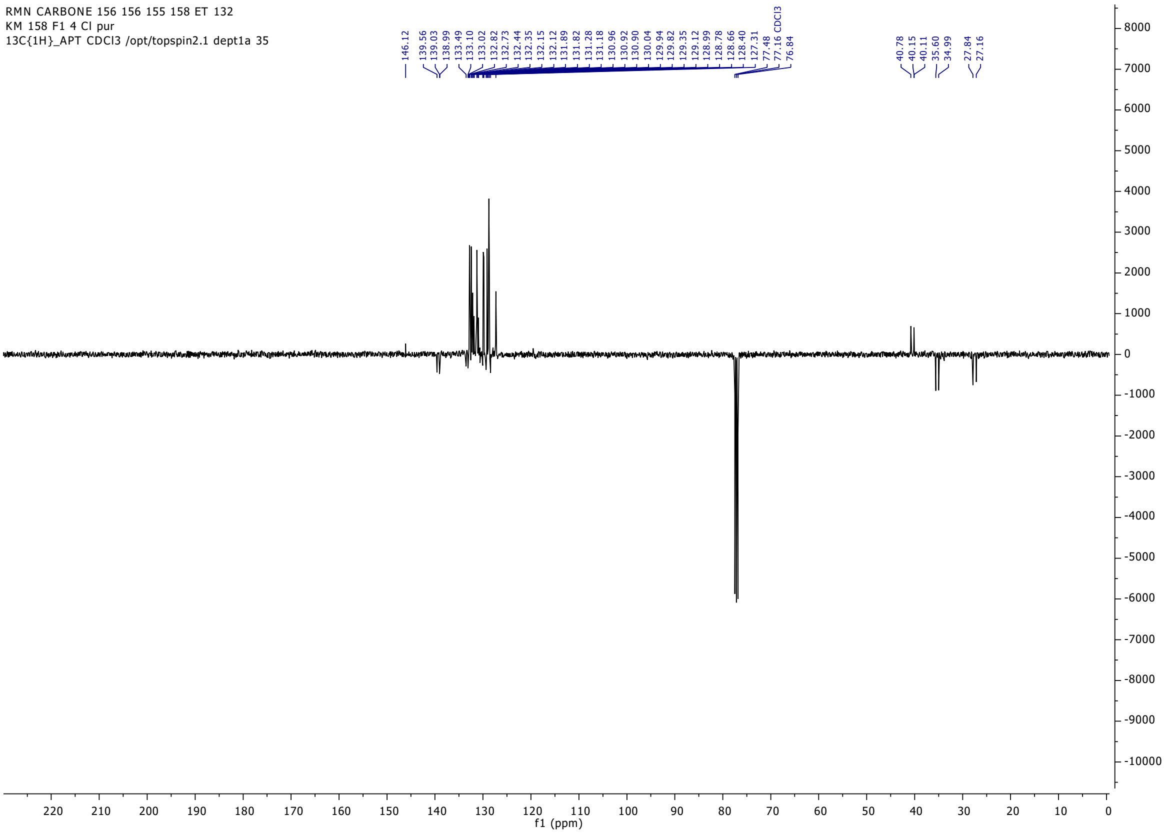

High Resolution Mass Result

Analysis Info

Sample Name **MMM-1 m _ KM158 f1**

Acquisition Date 7/26/2022 12:06:50 PM

Instrument / Ser# micrOTOF-Q 228888.10300

Acquisition Parameter

Source Type ESI Ion Polarity Positive Scan Begin 50 m/z Scan End 2200 m/z

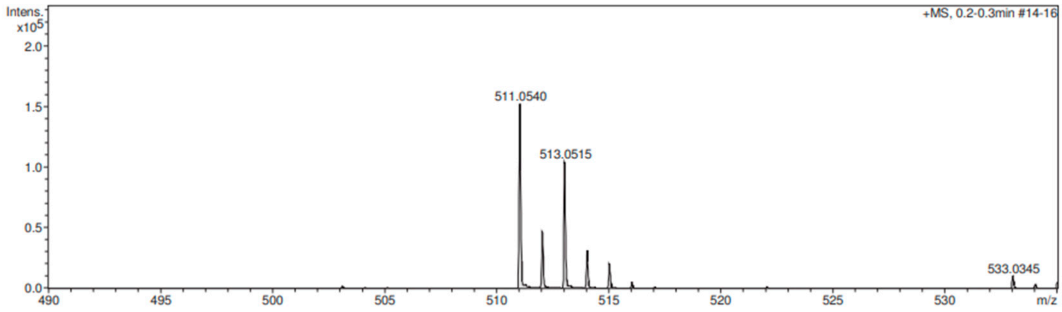

Meas. m/z	#	Ion Formula	m/z	err [ppm]	mSigma	# Sigma	Score	rdb	e⁻ Conf	N-Rule
511.0540	1	C27H23Cl2O2P2	511.0545	0.9	7.3	1	100.00	16.5	even	ok

4-(bis(2,3-dichloroxyphenyl)phosphoryl)-2-phenyl-1,3,4-trihydroisophosphinoline 2-oxide ISOP-o,mCla

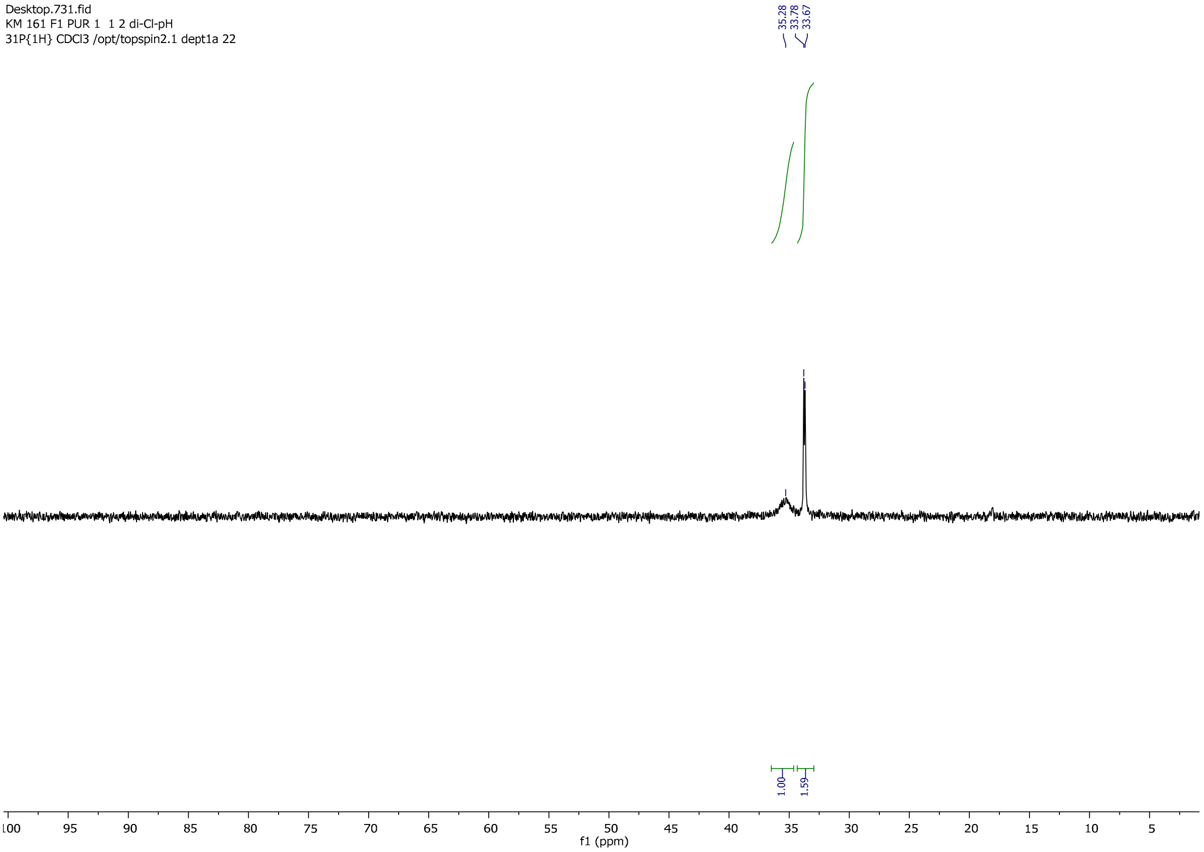

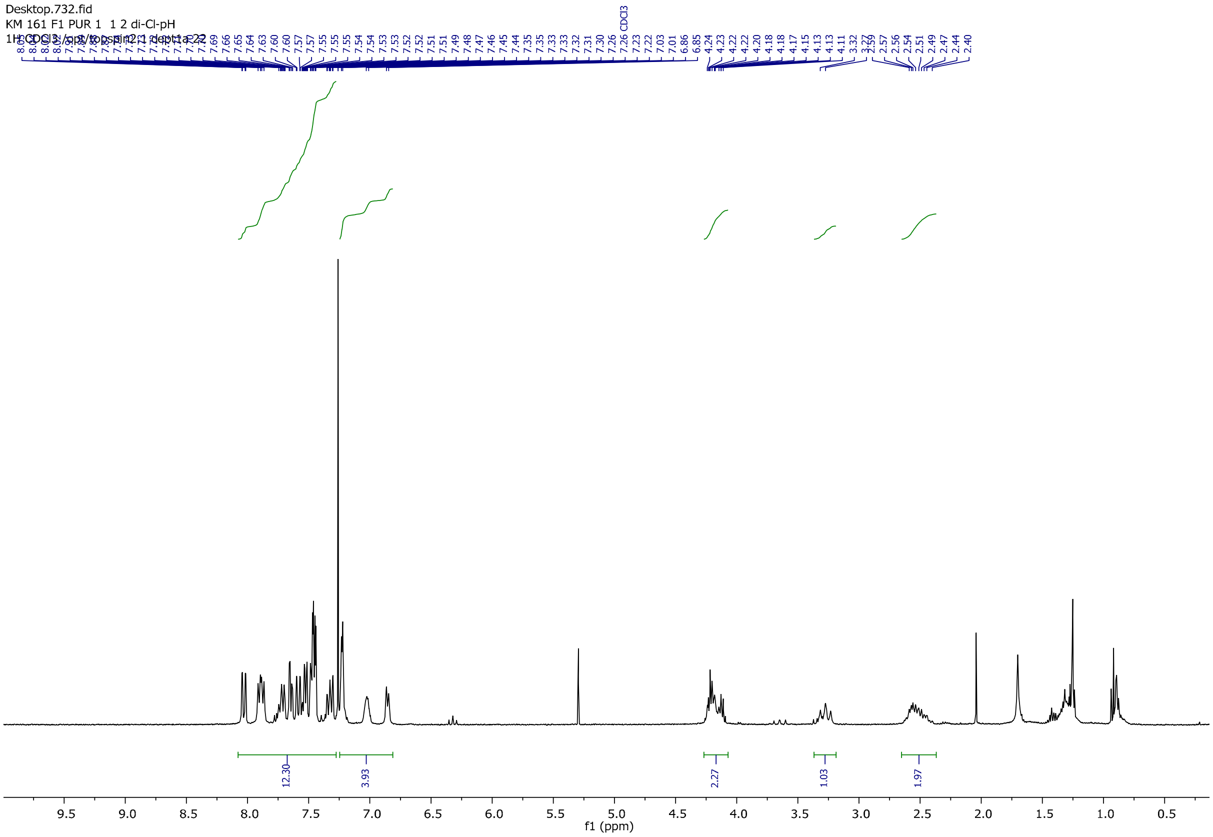

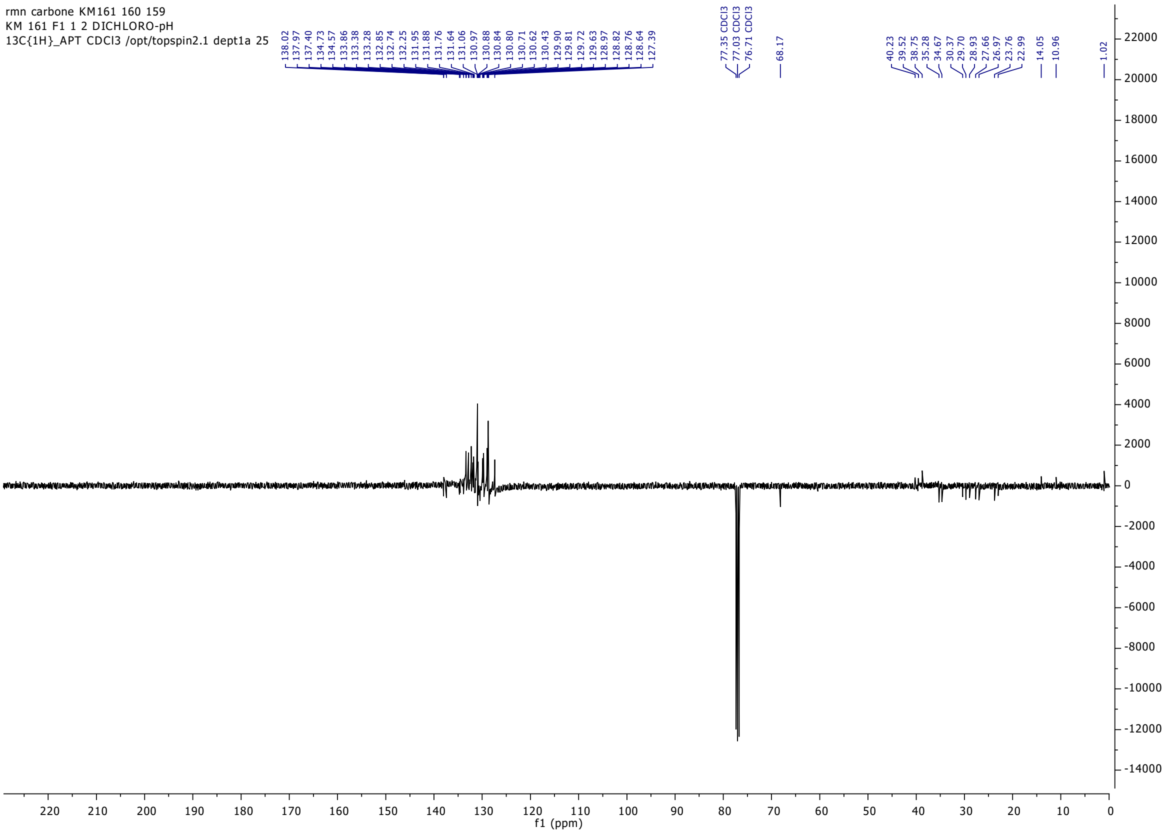

High Resolution Mass Result

Analysis Info

Sample Name **MMM-1 s _ KM161 f1**

Acquisition Date 7/26/2022 12:25:06 PM

Instrument / Ser# micrOTOF-Q 228888.10300

Acquisition Parameter

Source Type	ESI	Ion Polarity	Positive	Scan Begin	50 m/z	Scan End	2200 m/z

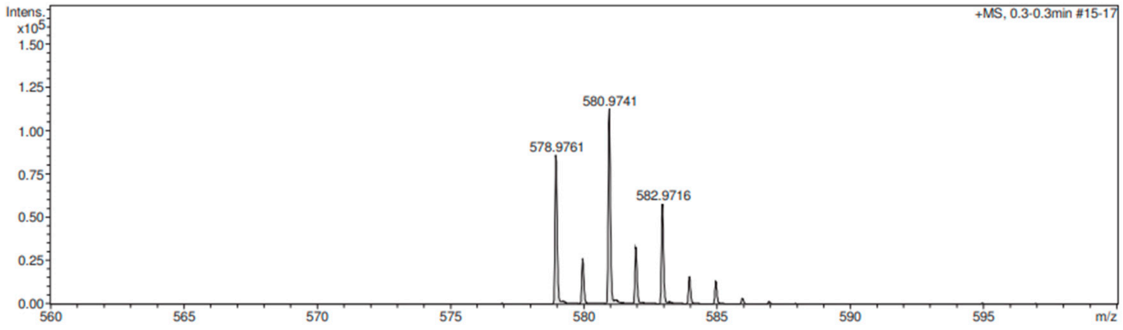

Meas. m/z	#	Ion Formula	m/z	err [ppm]	mSigma	# Sigma	Score	rdb	e⁻ Conf	N-Rule
578.9761	1	C27H21Cl4O2P2	578.9765	0.7	5.4	1	100.00	16.5	even	ok

4-(bis(4-chloroxyphenyl) phosphoryl)-2-phenyl-1,3,4-trihydroisophosphinoline 2-oxide Mixture ISOP-pCla + ISOP-pCla'

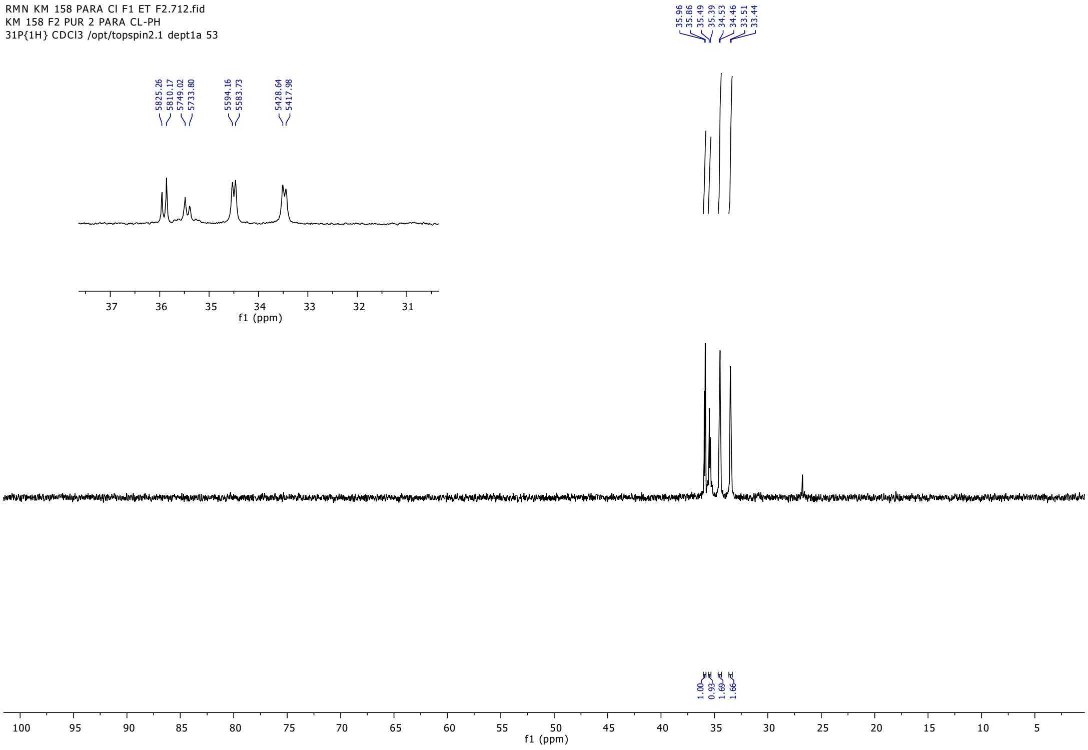

High Resolution Mass Result

Analysis Info

Sample Name **MMM-1 n _ KM158 f2**

Acquisition Date 7/26/2022 12:09:52 PM

Instrument / Ser# micrOTOF-Q 228888.10300

Acquisition Parameter

Source Type ESI Ion Polarity Positive Scan Begin 50 m/z Scan End 2200 m/z

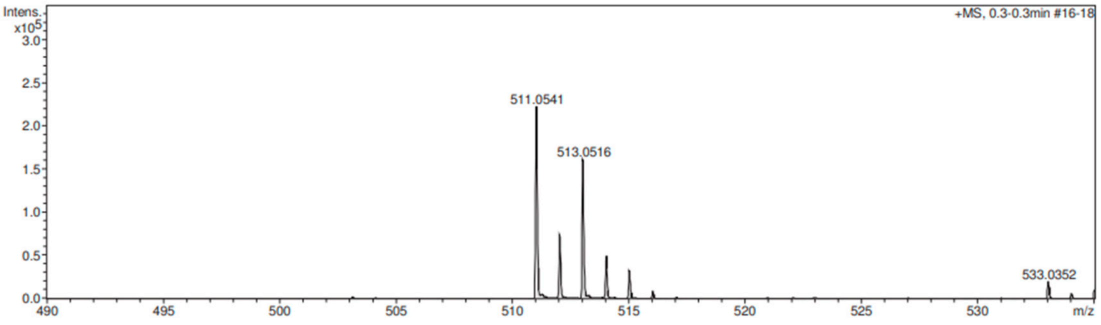

Meas. m/z	#	Ion Formula	m/z	err [ppm]	mSigma	# Sigma	Score	rdb	e⁻ Conf	N-Rule
511.0541	1	C27H23Cl2O2P2	511.0545	0.7	23.1	1	100.00	16.5	even	ok

4-(bis(2,3-dichloroxyphenyl)phosphoryl)-2-phenyl-1,3,4-trihydroisophosphinoline 2-oxide Mixture ISOP-o,mCla+ ISOP-o,mCla'

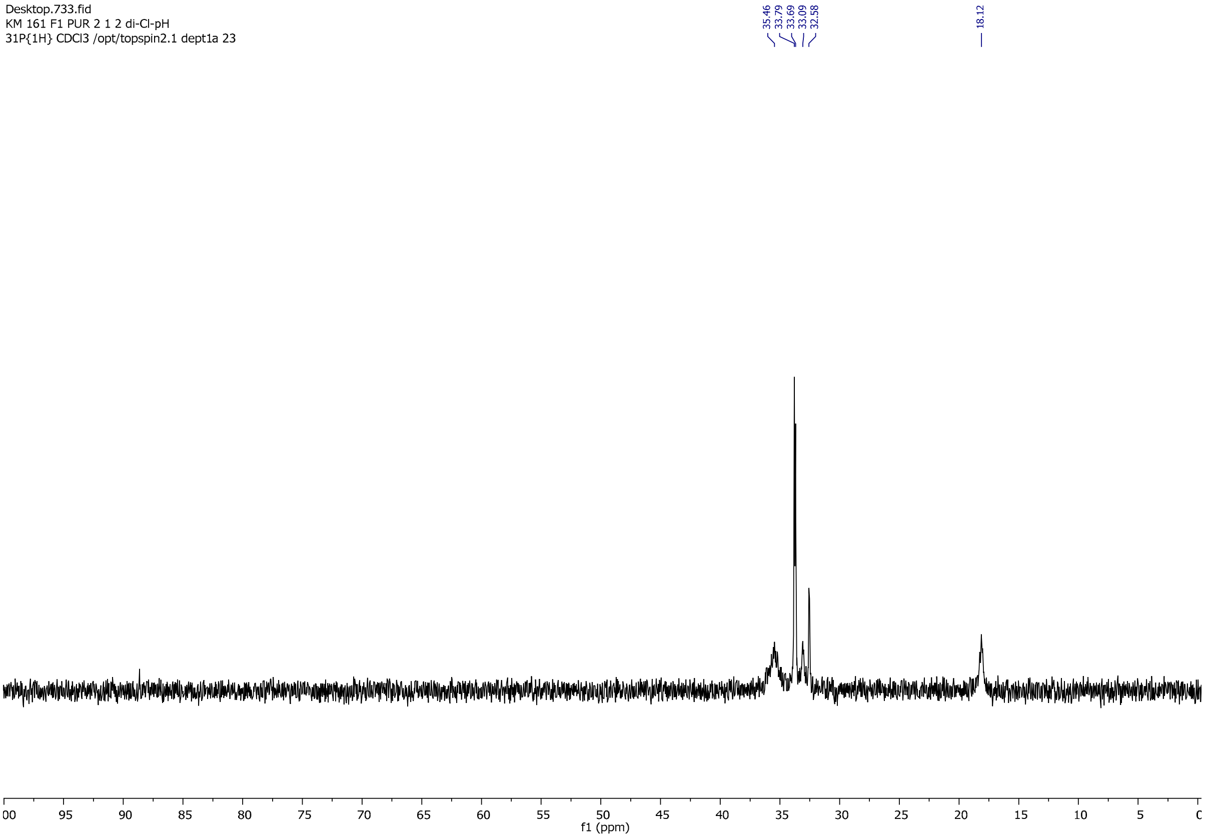

4-(bis(2,3-dichloroxyphenyl)phosphoryl)-2-phenyl-1,3,4-trihydroisophosphinoline 2-oxide ISOP-o,mCla’

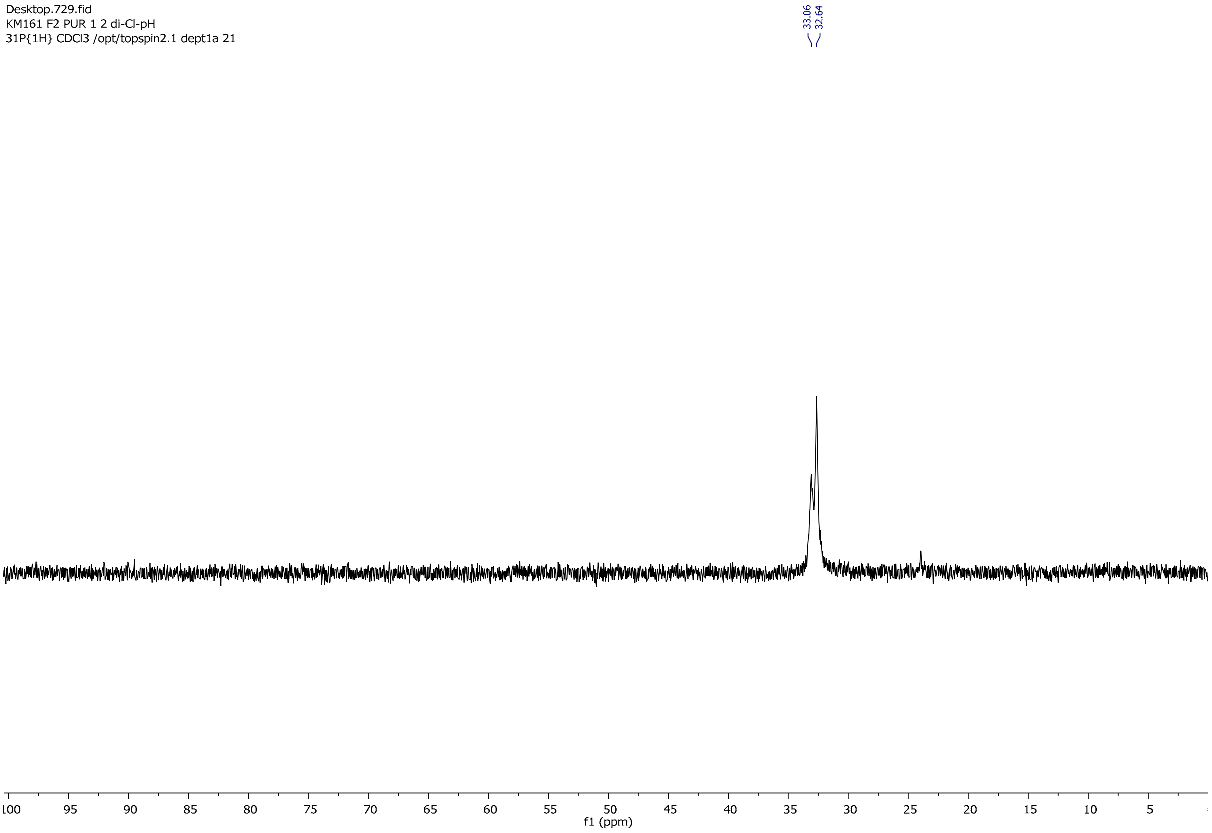

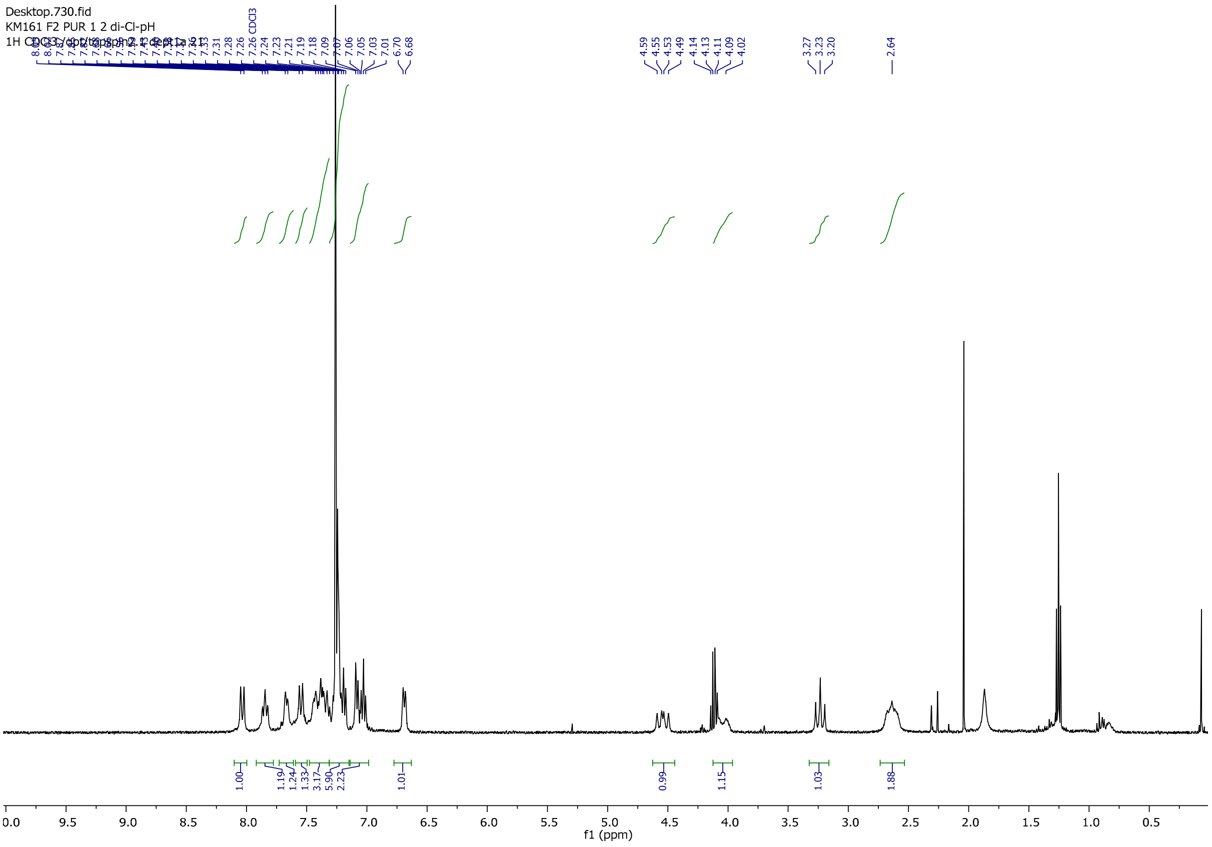

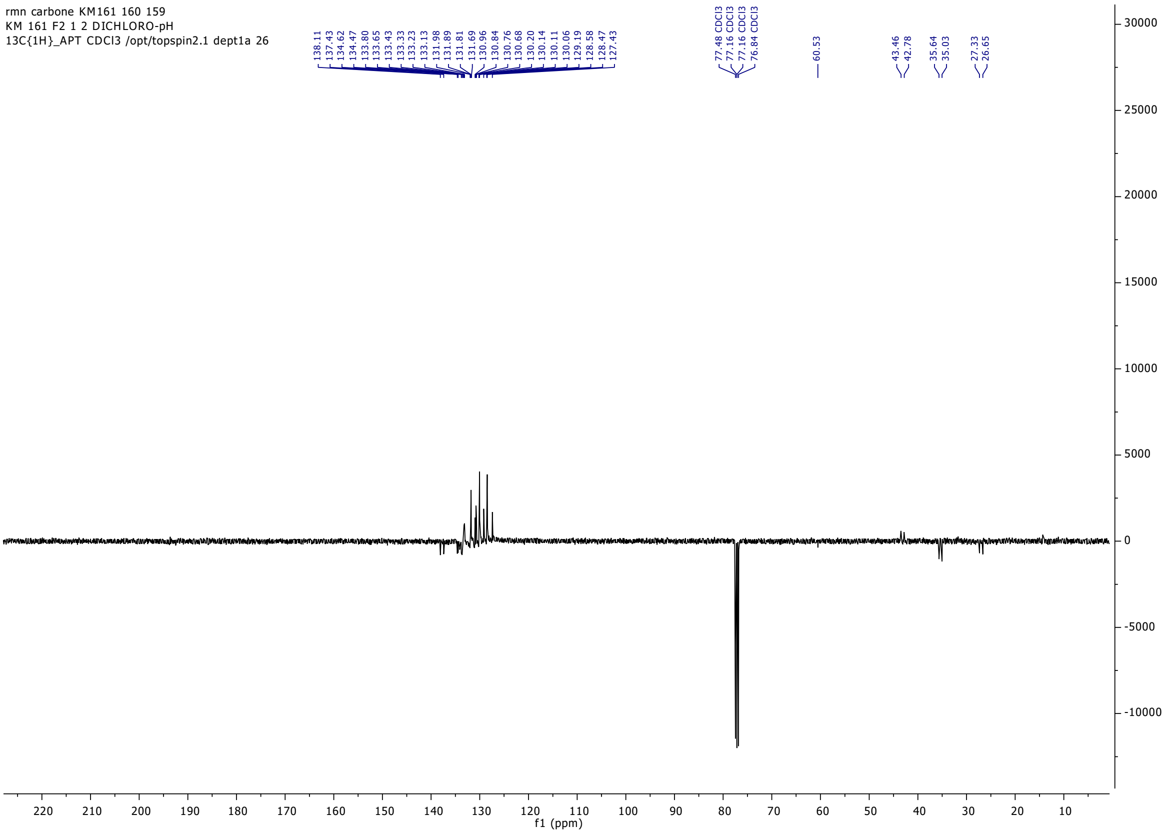

Acquisition Parameter

Source Type	ESI	Ion Polarity	Positive	Scan Begin	50 m/z	Scan End	2200 m/z

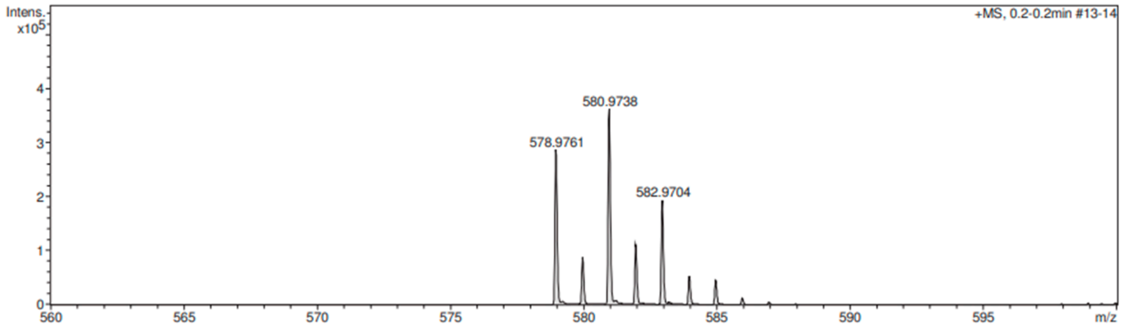

Meas. m/z	#	Ion Formula	m/z	err [ppm]	mSigma	# Sigma	Score	rdb	e⁻ Conf	N-Rule
578.9761	1	C27H21Cl4O2P2	578.9765	0.7	18.2	1	100.00	16.5	even	ok
	2	C23H17Cl4N6P2	578.9739	-3.9	24.8	2	27.07	17.5	even	ok

S3 - Comparison between TGA and PCFC

The comparison between PCFC and TGA was carried out to check inconsistencies in data.

Figure S3a plots the residue fraction in TGA and in PCFC. Residue fractions in both techniques are quite well correlated but it seems that its value is slightly higher in PCFC. This may be assigned to the heating rate (1 K/s in PCFC and 20 K/min in TGA).

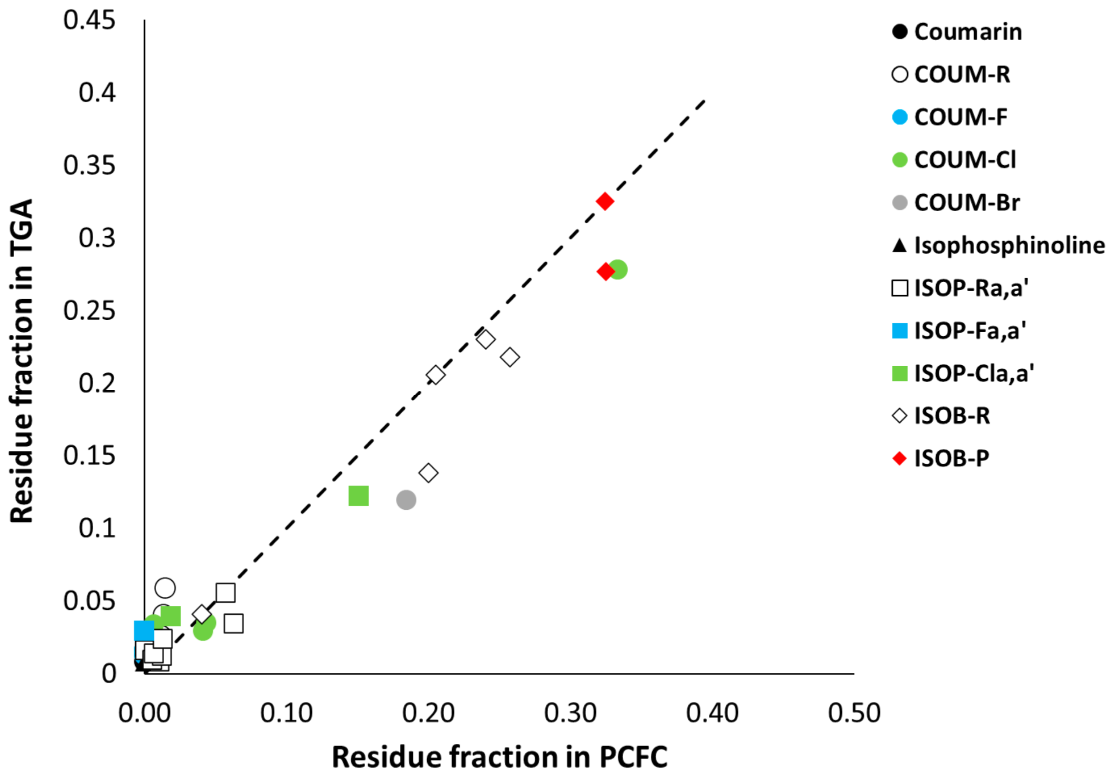

Figure S3a – Residue fraction in TGA versus residue fraction in PCFC for all molecules studied

Figure S3b plots the temperature of maximum decomposition rate in TGA and the temperature at pHRR in PCFC. Once again, there is a good correlation between both values for most of molecules because both peaks correspond usually to the same decomposition step. Tdmax is lower than TpHRR due to the different heating rates in both techniques (20 K/min in TGA and 1K/s in PCFC).

There are two molecules for which Tdmax and TpHRR are not correlated. Indeed, these two molecules decompose into two steps and the highest pMLR in TGA does not correspond to the highest pHRR in PCFC. This can be ascribed to a change in heat of combustion between both decomposition steps [11]. For example, in the case of **ISOB-3g** from group 3, the main pMLR corresponds to the first decomposition step but the heat release rate is the highest for the second step. It means that the heat of combustion is surely lower during the first step than during the second one.

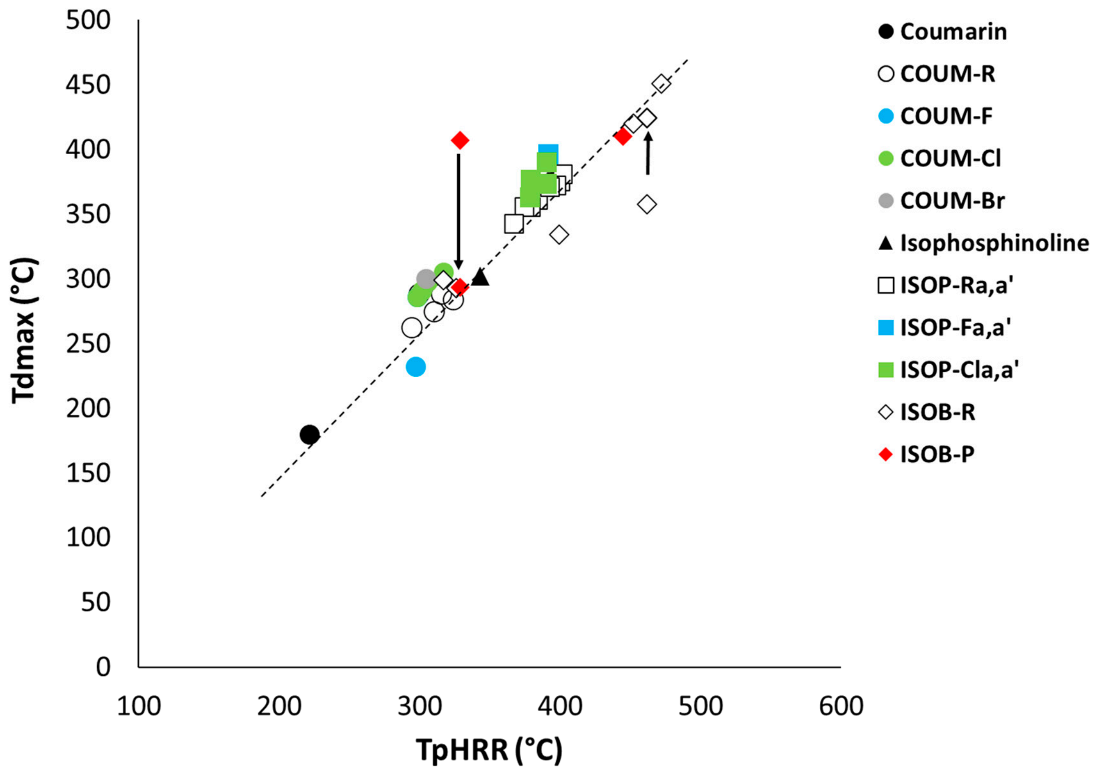

Figure S3b – Temperature at maximum decomposition rate in TGA versus temperature at pHRR in PCFC for all molecules studied

References

[1] Bruker (2012). *APEX3*, *XPREP* and *SAINT*. Bruker AXS Inc., Madison, Wisconsin, USA

[2] Van der Lee, A. Charge flipping for routine structure solution. *J. Appl. Crystallogr.* **2013**, *46*, 1306-1315. https://doi.org/10.1107/S0021889813020049

[3] Palatinus, L.; Chapuis, G.; SUPERFLIP—A Computer Program for the Solution of Crystal Structures by Charge Flipping in Arbitrary Dimensions. *J. Appl. Crystallogr.* **2007**, *40*, 786-790. https://doi.org/10.1107/S0021889807029238

[4] Betteridge, P.W.; Carruthers, J.R.; Cooper, R.I.; Prout, K.; Watkin, D. J. CRYSTALS version 12: software for guided crystal structure analysis. *J. Appl. Crystallogr.* **2003**, *36*, 1487-1487. https://doi.org/10.1107/S0021889803021800

[5] a) Tripolszky, A.; Keglevich, G. Synthesis of secondary phosphine oxides by substitution at phosphorus by grignard reagents, *Letters in Organic Chemistry*, **2018**, 15, 387-393. https://doi:10.2174/1570178615666171227144555.

[6] Yuan, Q.; Liu, H.-W.; Cai, Z.-J.; Ji, S.-J. Direct 1,1-bisphosphonation of isocyanides: atom- and step-economical access to bisphosphinoylaminomethanes. *ACS.Omega,* **2021**, 6, 8495. https://doi.org/10.1021/acsomega.1c00160

[7] Lhermet, R.; Moser, E.; Jeanneau, E.; Olivier-Bourbigou, H.; Breuil, P.-A.R.; Outer-sphere reactivity shift of secondary phosphine oxide-based nickel complexes: from ethylene hydrophosphinylation to oligomerization *Chem. Eur. J.* **2017**, 23, 7433–7437. https://doi10.1002/chem.201701414

[8] Duan, S.; Pan, A.; Du,Y.; Zhu, G.; Tian, X.; Zhang, H.; Walsh, P.J.; Yang, X. Nickel-Catalyzed Enantioselective Hydrophosphinylation of 2-Azadienes to Access Enantioenriched α-Aminophosphine Oxides. *ACS Catal.* **2023**, *13*, 10887−10894. https://doi.org/10.1021/acscatal.3c02870

[9] Dapeng Zhang, D.; Lian, M.; Liu, J.; Tang, S.; Liu, G.; Ma, C.; Meng, Q.; Peng, H.; Zhu, D. Preparation of O-Protected Cyanohydrins by Aerobic Oxidation of α-Substituted Malononitriles in the Presence of Diarylphosphine. Oxides. *Org. Lett.* **2019**, *21*, 2597−2601. https://DOI:10.1021/acs.orglett.9b00569

[10] A type triarylphosphine compound and synthetic method and application thereof Chinese Patent, 2019, CN 1149207774 A.

[11] Microscale forced combustion: Pyrolysis-combustion flow calorimetry (PCFC), R. Sonnier, chapter 3 from Analysis of Flame Retardancy in Polymer Science, Elsevier, 2022, Pages 91-116, editors H. Vahabi, M. Saeb, G. Malucelli
